# A comprehensive next generation sequencing-based virome assessment in brain tissue suggests no major virus - tumor association

**DOI:** 10.1186/s40478-016-0338-z

**Published:** 2016-07-11

**Authors:** Michael J. Strong, Eugene Blanchard, Zhen Lin, Cindy A. Morris, Melody Baddoo, Christopher M. Taylor, Marcus L. Ware, Erik K. Flemington

**Affiliations:** Department of Pathology, Tulane University, New Orleans, LA 70112 USA; Tulane Cancer Center, New Orleans, LA 70112 USA; Department of Microbiology, Immunology & Parasitology, Louisiana State University School of Medicine, New Orleans, LA 70112 USA; Department of Microbiology and Immunology, Tulane University School of Medicine, New Orleans, LA 70112 USA; Department of Neurological Surgery, Ochsner Clinic Foundation, New Orleans, LA 70112 USA

**Keywords:** Human cytomegalovirus, Brain tumors, Gliomas, Meningiomas, Transcriptome analysis

## Abstract

Next generation sequencing (NGS) can globally interrogate the genetic composition of biological samples in an unbiased yet sensitive manner. The objective of this study was to utilize the capabilities of NGS to investigate the reported association between glioblastoma multiforme (GBM) and human cytomegalovirus (HCMV). A large-scale comprehensive virome assessment was performed on publicly available sequencing datasets from the Cancer Genome Atlas (TCGA), including RNA-seq datasets from primary GBM (*n* = 157), recurrent GBM (*n* = 13), low-grade gliomas (*n* = 514), recurrent low-grade gliomas (*n* = 17), and normal brain (*n* = 5), and whole genome sequencing (WGS) datasets from primary GBM (*n* = 51), recurrent GBM (*n* = 10), and normal matched blood samples (*n* = 20). In addition, RNA-seq datasets from MRI-guided biopsies (*n* = 92) and glioma stem-like cell cultures (*n* = 9) were analyzed. Sixty-four DNA-seq datasets from 11 meningiomas and their corresponding blood control samples were also analyzed. Finally, three primary GBM tissue samples were obtained, sequenced using RNA-seq, and analyzed. After in-depth analysis, the most robust virus findings were the detection of papillomavirus (HPV) and hepatitis B reads in the occasional LGG sample (4 samples and 1 sample, respectively). In addition, low numbers of virus reads were detected in several datasets but detailed investigation of these reads suggest that these findings likely represent artifacts or non-pathological infections. For example, all of the sporadic low level HCMV reads were found to map to the immediate early promoter intimating that they likely originated from laboratory expression vector contamination. Despite the detection of low numbers of Epstein-Barr virus reads in some samples, these likely originated from infiltrating B-cells. Finally, human herpesvirus 6 and 7 aligned viral reads were identified in all DNA-seq and a few RNA-seq datasets but detailed analysis demonstrated that these were likely derived from the homologous human telomeric-like repeats. Other low abundance viral reads were detected in some samples but for most viruses, the reads likely represent artifacts or incidental infections. This analysis argues against associations between most known viruses and GBM or mengiomas. Nevertheless, there may be a low percentage association between HPV and/or hepatitis B and LGGs.

**Electronic supplementary material:**

The online version of this article (doi:10.1186/s40478-016-0338-z) contains supplementary material, which is available to authorized users.

## Introduction

Glioblastoma multiforme (GBM) is the most common malignant primary brain tumor in adults. An estimated 77,670 new cases of primary CNS tumors are expected to be diagnosed in the United States in 2016 [1]. Of these, 24,790 will be diagnosed as malignant [1]. Although the incidence of primary brain tumors is low compared to other cancer types, primary brain tumors give rise to a disproportionate amount of morbidity and mortality, often robbing patients of basic and critical functions such as movement and speech [2]. The median survival of newly diagnosed patients is only 12–15 months, making it one of the most devastating types of cancers [3]. In fact, the five-year survival rate for primary malignant brain and central nervous system tumors is the sixth lowest among all types of cancers after pancreatic, liver & intrahepatic bile duct, lung, esophageal, and stomach [2]. Unfortunately, despite substantial investigations into disease mechanisms and at least some advances in currently available treatment options, the outcomes for GBM patients remain dismal [3].

Although an association between human cytomegalovirus (HCMV) and GBM was first observed in 2002 [4], there is still a high degree of discordance in the literature regarding the detection of viral agents in CNS tumors [4–28]. These discrepancies have been attributed to a number of issues including the use of different cohorts, differences in sensitivities of different PCR assays for low levels of viral gene expression, and the exquisite sensitivities of assays such as IHC to slight differences in experimental conditions.

In an attempt to remedy the high degree of discordance regarding the detection of HCMV in CNS tumors, an HCMV and glioma symposium was convened in Washington, DC on April 17, 2011. At the conclusion of this workshop, a summary paper was published reporting the consensus position in 4 major areas: 1) the existence of HCMV in gliomas, 2) the role of HCMV in gliomas, 3) HCMV as a therapeutic target, and 4) key future investigative directions [29]. Based on the evidence presented at the workshop, it was concluded that HCMV sequences and viral gene expression exist in many malignant gliomas and that in vitro studies support the idea that HCMV can modulate key signaling pathways in glioblastomas [29].

Next generation sequencing (NGS) has the ability to globally interrogate the genetic composition of biological samples in an unbiased manner and with relatively high sensitivity. Applying this technology to pathogen discovery has already shown promise, resulting in the discovery of a novel Merkel cell polyomarvirus in Merkel cell carcinoma [30], for example. In our laboratory, we have utilized NGS technology in the interrogation of Epstein-Barr virus (EBV) in diffuse large B-cell lymphomas [31] and gastric carcinoma [32]. The goal for the study presented here was to help resolve the lingering controversy pertaining to the presence of HCMV in GBM while at the same time providing a comprehensive and unbiased assessment of the viral genetic composition of brain tumor biopsies. This analysis failed to find convincing evidence for an association between HCMV or other known viruses and GBM or mengiomas. Nevertheless, we detected human papillomavirus (HPV) and hepatitis B in some low-grade gliomas (LGG). In addition, we expand on our previous reporting of potential contamination and/or interpretational artifacts that need to be considered in next generation sequencing based metagenomic and metatranscriptomic studies [33, 34].

## Materials and methods

### Clinical tumor sample and sequence data acquisition

All human specimens were de-identified prior to acquisition. Fresh frozen tissue from 1 GBM sample was obtained from the Louisiana Cancer Research Consortium (LCRC) Biospecimen Core. Additionally, RNA isolated from 2 GBM samples were obtained from BioServe. An RNA-seq dataset from a lymphoblastoid cell line immortalized with EBV (JY) was used as a control for downstream analysis [35].

Publically available sequence datasets were obtained from various sources. Next generation sequencing datasets from The Cancer Genome Atlas (TCGA) initiative were downloaded from the Cancer Genomics Hub (CGHub; https://cghub.ucsc.edu) and included RNA-seq datasets (unaligned fastq files) from primary GBM tumors [36–38] (*n* = 157), recurrent GBM tumors (*n* = 13), low grade gliomas [39] (LGG; *n* = 514), recurrent low grade gliomas (*n* = 17), and normal brain (*n* = 5), and TCGA whole genome sequencing datasets (aligned bam files) from primary GBM tumors (*n* = 51), recurrent GBM tumors (*n* = 10), and normal matched blood samples (*n* = 20) (Additional file 1: Table S1). The aligned bam files were converted to fastq files using bam2fastq (https://gsl.hudsonalpha.org/information/software/bam2fastq, default parameters).

Additional brain tissue sequencing datasets were obtained from the NCBI Sequence Read Archive (Additional file 1: Table S1). RNA-seq datasets from tumor and peripheral brain tissue of a GBM patient were obtained using accession number SRP009144 [40]. Normal brain tissue RNA-seq dataset from the Illumina Human Body Map 2.0 project was obtained using (NCBI GEO accession GE30611). RNA-seq datasets from a cohort of short-term cultures of glioma stem-like cells freshly isolated from nine patients diagnosed with primary GBM were downloaded using accession number SRP016798 [41, 42]. A cohort of RNA-seq datasets from MRI-localized biopsies of the tumor core and margins from multiple glioma patients and non-neoplastic brain tissue specimens were downloaded using accession number SRP044668 analyzed [43]. Non-neoplastic brain tissue samples were obtained from multiple patients undergoing procedures to alleviate epilepsy symptoms or to place shunts to treat normal pressure hydrocephalus. In total, RNA-seq datasets from 39 contrast-enhancing glioma core samples, 36 non-enhancing FLAIR glioma margin samples, and 17 non-neoplastic brain tissue samples were analyzed. A cohort of 64 whole genome sequencing datasets from 11 Grade I meningiomas and 11 matched blood samples were obtained using accession number SRP016129 [44]. Finally, RNA-seq datasets from HCMV infected fibroblasts were downloaded using accession number SRP016143 [45].

### Sample preparation and next generation RNA sequencing

Total RNA was extracted from 3 primary GBM biopsies (00RTS3 – from the LCRC biospecimen core; and CAURPRVE and H8CPFRSJ – from Bioserve) using Trizol (Invitrogen, Carlsbad, CA) according to manufacturer’s instructions. Total RNA from sample 00RTS3 was subjected to polyA selection, and the library was prepared using the ScriptSeq Protocol (Epicentre, Madison, WI) and subjected to 2x101 base paired-end sequencing on an Illumina Hi-seq 2000 machine. Total RNA from samples CAURPRVE, H8CPFRSJ, and JY were subjected to ribosomal RNA depletion using the Ribo-Zero kit (Epicentre, Madison, WI) and cDNA libraries were prepared using the Illumina Truseq Stranded Total RNA Sample Prep Kit and subjected to 1x101 base single-end multiplexed sequencing on an Illumina Hi-seq 2000 machine. The RNA-seq data used in this publication is available through GEO Series accession number (in process).

### Metatranscriptomic analysis using RNA CoMPASS

Metatranscriptome analysis was performed by running single-end or one pair from paired-end sequencing data through our automated RNA-seq exogenous organism analysis software, RNA CoMPASS [46]. Within RNA CoMPASS, reads were aligned to the human reference genome, hg19 (UCSC), plus a splice junction database (which was generated using the make transcriptome application from Useq [47]; splice junction radius set to the read length minus 4) using Novoalign V3.00.05 (www.novocraft.com) [−o SAM, default options]. Non-mapped reads were subjected to a BLAST V2.2.30 search against the Human RefSeq RNA database to identify and remove human reads that fail to be identified through the Novoalignment. Remaining non-human reads were then subjected to a BLAST V2.2.30 search against the NCBI NT database to identify reads corresponding to known exogenous organisms [48]. Results from the NT BLAST searches were filtered to eliminate matches with an E-value of greater than 10e^−6^. The results were then fed into MEGAN 4 V4.70.4 for visualization of taxonomic classifications [49]. RNA CoMPASS was run in parallel on three 2x2.66 GHz 12 core Intel Xeon Mac Pro computers with 64-96GBs of memory each.

### Virome analysis

Raw sequence data from RNA-seq and DNA-seq were aligned to a reference containing a human genome (hg19; Genome Reference Consortium GRCH37) plus a library of 740 virus genomes (including sequences from all known human viruses documented by NCBI) [50]. The alignments were performed using Spliced Transcripts Alignment to a Reference (STAR) aligner version 2.3.0 [−−clip5pNbases 6 (only used if reads were longer than 36 base pairs), default options] [51]. Uniquely mapped viral and human reads were quantified using in-house computational pipelines. Signal maps (i.e. the total number of reads covering each nucleotide position) from viruses of interest were generated using IGV tools and were subjected to manual visual inspection using the IGV genome browser [52].

### Quantitative RT-PCR

Total RNA was reverse-transcribed using the SuperScript III First-Strand Synthesis System for RT-PCR (Invitrogen, Carlsbad, CA). Random hexamers were used with 1 ug total RNA in a 20 μl reaction volume according to manufacturer’s instructions. For the incubation steps (25 °C for 10 min followed by 50 °C for 50 min) a Mastercycler ep (Eppendorf, Hamburg, Germany) was used. For real-time PCR, 1 μl of the resulting cDNA (diluted to 10 ng/ul) was used in a 10 μl reaction mixture that included 5 μl of 2x iQ SYBR Green Supermix (Bio-Rad, Hercules, CA), 1 μl of 10 μM forward and reverse primer mix (Integrated DNA Technologies, Coralville, IA), and 3 μl of PCR grade water.

A list of PCR primer oligos can be found in Additional file 2: Table S2. Each sample was PCR’ed in triplicate. No-template controls and no-reverse transcription controls were also included in each PCR run. Thermal cycling was performed on a CFX96 Real Time System (Bio-Rad, Hercules, CA) and data analysis was performed using the CFX Manager 3.0 software. Cycling conditions included an initial incubation at 95 °C for 3 min followed by 40 cycles consisting of 95 °C for 15 s, and 60 °C for 60 s. Melting curve analysis was performed at the end of every qRT-PCR run.

## Results

### Lack of virus association in the cancer genome atlas GBM RNA-seq datasets

**Fig. 1:**
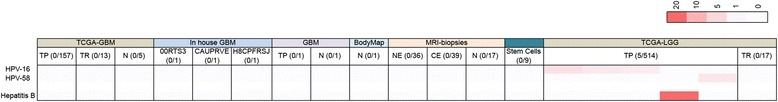
Heat map showing the number of viral reads per million human mapped (RPHM) reads for brain tissue RNA-seq datasets. Color intensity represents relative viral RPHM across all datasets

Previous studies have used human read subtraction-based approaches to assess the metatranscriptomic profile of primary and recurrent glioma RNA-seq datasets from The Cancer Genome Atlas (TCGA) where a lack of association with HCMV was reported [10, 14, 26, 27]. To investigate this issue further, we first performed a global virome analysis on these samples (as well as 5 normal brain tissue samples), using a more directed, non-subtraction based approach that we have reported previously [33, 50, 53]. For this analysis, we directly aligned all reads from these datasets to an alignment index containing the human genome plus a library of 740 virus genomes (including sequences from all known human viruses documented by NCBI) using the Spliced Transcripts Alignment to a Reference (STAR) aligner. Running this virome pipeline on a known EBV-associated gastric cancer tissue biopsy cohort from TCGA [54] showed EBV read levels which ranged from 7–400 viral Reads Per Mapped Human reads (RPMH) [53]. Like PCR, next generation sequencing is susceptible to low level contamination issues [34]. Nevertheless, in an attempt to capture potential low abundance infections, we set a viral read threshold that was 10 times lower than the lowest viral RPMH value for EBV in the gastric cancer cohort [53, 54]. Based on this requirement of at least 0.7 viral RPMH, no virus associations were called in 157 primary and 13 recurrent gliomas or in 5 normal brain RNA-seq datasets analyzed except for the finding of 2.1 human herpesvirus (HHV) -6 or HHV-7 RPMH (note: HHV-6 and HHV-7 reads are considered together here due to all reads mapping to a homologous region of these two viruses) in one recurrent glioma (Fig. 1, Additional file 3: File S1). Notably, all HHV6/7 reads in this sample were found to contain the simple sequence, TAACCC, a repeat found in both of these virus genomes and in human telomeric repeats. Since no HHV6/7 assigned reads mapped outside of the TAACCC repeat region of the viral genomes, we conclude that these reads likely originated from contaminating genomic sequences from human telomeric repeats.

Analysis of RNA-seq data from a cohort of time course HCMV infected fibroblasts [45] demonstrated robust numbers of HCMV reads (ranging from 68,875 – 578,635 RPMH) that escalated roughly proportionally to the length of infection (Additional file 4: File S2). Since our detection cutoff of 0.7 RPMH for TCGA cohort is about 5 to 6 orders of magnitude less than these read numbers, this data supports a lack of association between HCMV and GBMs.

#### Assessment of sequence library preparation strategies

Although the bulk of viral RNAs are polyadenylated, some viral transcripts are not. To assess whether we were missing viral infections because of a lack of detecting non-polyadenylated viral transcripts, we wanted to test whether sequencing of ribodepleted RNAs (versus polyA selected RNAs as per the TCGA cohort) might yield the detection of viral reads. HCMV has been reported to have a high penetrance in GBM with some studies reporting as high as 90–100 % positivity [4, 17, 19, 24, 29]. We sequenced two of our own GBM samples using ribosomal depleted RNA as well as an additional sample using poly-A selected RNA. Because virome analysis was not previously performed on these samples, we implemented two virus detection approaches. First we analyzed each of these samples through our custom subtraction based pathogen detection pipeline, RNA CoMPASS, which analyzes the entire metatranscriptome. For a more focused virome analysis, we then implemented the STAR aligner approach.

No associations were found with any known viruses in the polyA selected RNA sample (00RTS3) (Fig. 1) using either RNA CoMPASS (Additional file 5: Figure S1) or the STAR/virome analysis approach (Additional file 6: File S3). Additional publically available poly-A selected RNA datasets from a GBM study, in which they sequenced one primary GBM and the matched normal brain, and a normal brain sample from the BodyMap project were obtained and analyzed. Assessing these public poly-A selected RNA datasets similarly showed no association with any known virus (Fig. 1, Additional file 5: Figures S2, S3 and S4).

Analysis of the ribosomal depleted RNA samples (CAUPRVE and H8CPFRSJ) revealed reads from the murine leukemia virus (MuLV) family (2.4 and 4.3 RPMH, respectively) and a low abundance of EBV reads (0.8 and 1.9 RPMH, respectively) (Additional file 6: File S3 and Additional file 5: Figures S5 and S6). Further analysis of the viral read coverage for MuLV and EBV demonstrated near identical coverage patterns to another sample (JY – an EBV-immortalized B cell lymphoblastoid cell line, which we have previously shown to be infected with MuLV [35]) that was sequenced in the same sequencing lane (Additional file 5: Figure S7). Our suspicion of contamination across barcodes was confirmed by real-time PCR for samples CAUPRVE and H8CPFRSJ, in which neither MuLV nor EBV transcripts were detected (Additional file 5: Figure S8). Real-time RT-PCR for HCMV transcripts were similarly negative in these samples, thus confirming the lack of RNA-seq based HCMV findings in these datasets.

#### Analysis of tumor tissue sampling

Since GBM is a very heterogeneous solid tumor, differential sampling of the tumor mass may result in different transcriptomic and metatranscriptomic profiles. To take this issue into account for the detection of HCMV, we took advantage of a well-designed study in which the authors sequenced MRI-localized biopsies of the tumor core and margins from multiple GBM patients. The authors also sequenced several non-neoplastic brain tissue samples [43]. Virome assessment of this cohort using RNA CoMPASS detected no viruses (Additional file 5: Figures S9 – S102). Assessment of this cohort using the STAR/virome approach detected Human Immunodeficiency virus type 1 (HIV-1) at levels greater than 0.7 viral RPMH in 2 glioma samples (5.3 and 5.5 RPMH) taken from the non-enhancing FLAIR portion (margins) of the tumor and 2 glioma samples (3.1 and 4.2 RPMH) taken from the contrast enhancing portion (core) of the tumor (Additional file 6: File S3). To investigate these findings further, we analyzed the genome coverage of HIV reads in these samples plus three samples that showed HIV read levels that are below our 0.7 RPMH threshold (1 non-neoplastic sample (0.37 RPMH) and 2 additional contrast enhancing core samples (0.04 and 0.16 RPMH)). Inspection of the HIV-1 read coverage from all seven glioma samples revealed that the majority of the HIV-1 reads aligned to the long terminal repeat regions and additional homologous regions of the expression vector pH1TO, as shown in Additional file 5: Figure S103. MuLV reads were also detected in 33 out of the 36 glioma samples (0.03 – 5.74 RPMH) taken from the non-enhancing FLAIR portion of the tumor, 35 out of the 39 glioma samples (0.03 – 12.72 RPMH) taken from the contrast enhancing portion of the tumor, and 15 out of the 17 non-neoplastic samples (0.03 – 3.33 RPMH) (Additional file 6: File S3). Inspection of the MuLV read coverage showed a sporadic genomic coverage pattern that was similar across the tumor samples (Additional file 5: Figures S104-S106). The observed similar coverage profiles, the finding of low read numbers, and our previous observations of sample cross contamination of human samples with MuLV, lead us to suspect that the MuLV read findings here are most likely due to cross contamination. Finally, although the level of HCMV reads did not exceed 0.7 viral RPMH in any given sample, for completeness, all detected HCMV reads including those from 14 glioma samples from non-enhancing FLAIR portions (0.03 – 0.21 RPMH) and 12 glioma samples from contrast enhancing portions (0.04 – 0.25 RPMH) were analyzed further. All HCMV reads were found to align to the HCMV immediate early promoter (Additional file 5: Figures S107 and S108). As mentioned above for the HCMV findings in the TCGA cohort, this is suggestive of sample contamination with laboratory expression plasmids bearing the HCMV promoter. After accounting for the artifactual viral reads, we conclude that this cohort shows no likely association with any known viruses (Fig. 1).

#### Analysis of GBM stem cell subpopulations

Due to the low abundance and limited detection of HCMV in GBMs reported in the literature, some groups have proposed that HCMV is harbored in only a small number of tumor cells, specifically the CD133+ tumor stem cells [55–57]. To address this possibility, we analyzed RNA-seq datasets generated from a cohort of short-term glioma stem-like cell cultures freshly isolated from nine patients diagnosed with primary GBM. Analysis of these datasets using our STAR/virome pipeline showed the detection of human Adenovirus C (HAdV-C) in 9 out of the 24 samples with the remaining 15 samples showing low abundance HAdV-C read levels Additional file 6: File S3 and Additional file 5: Figures S109-S132. HAdV-C was also detected in the same 9 samples using RNA CoMPASS (Additional file 5: Figures S112-S132). Despite detecting HAdV-C in these samples, viral read coverage was primarily limited to the same three small regions of the genome in the majority of samples (Additional file 5: Figure S133). Further manual blast analysis of the HAdV-C reads demonstrated homology with laboratory adenovirus vectors (data not shown). Although we detected only low level HCMV reads in 3 glioma stem-like cell culture samples (0.03 – 0.1 RPMH), we further analyzed these reads due to the historical significance of this virus with GBM. Similar to our previous HCMV findings discussed above, viral read coverage analysis again showed that all reads aligned with the HCMV IE1 promoter (Additional file 5: Figure S134). RNA CoMPASS analysis did not identify HCMV reads in these samples (Additional file 5: Figures S112-S113 and S131).

#### Virome analysis of TCGA low-grade gliomas

To study the virome of low-grade gliomas (LGG), RNA-seq datasets from 514 primary and 17 recurrent LGGs from the TCGA were analyzed [39]. Due to the magnitude of the sample number, we exclusively used the sensitive yet rapid approach of our STAR/virome method. Based on this analysis, Human Papillomavirus (HPV) 16 was detected in 3 out of the 514 samples (1.5 – 2.4 RPMH), although lower HPV-16 read numbers were observed in 22 additional samples (Fig. 1 and Additional file 7: File S4). Inspection of HPV-16 read coverage for the 3 positive samples showed expression of the HPV-16 E6 and E7 oncogenes with lower coverage of the E1, E2 and E4 regions (Additional file 5: Figure S135). The lack of any coverage of the right half of the HPV-16 genome is consistent with deletion of this region which is frequently observed in oncogenic HPV genome integrations (where viral integration and the concomitant deletion of these negative regulatory genes results in increased expression of the oncogenic E6 and E7 genes while at the same time preventing productive viral infection and host cell destruction). We also detected HPV-58 in 1 sample (1.9 RPMH) with lower HPV-58 reads in 2 additional samples (Fig. 1 and Additional file 7: File S4). Inspection of viral read coverage for the HPV-58 positive samples showed that the single sample with read levels above the 0.7 RPMH threshold had good read coverage of the E6, E7, and the E2/E4/E5 region (Additional file 5: Figure S136). Finally, Hepatitis B reads were detected in 1 sample (13 RPMH), although below threshold Hepatitis B reads were detected in 2 additional samples (Fig. 1 and Additional file 7: File S4). Inspection of Hepatitis B read coverage for the above threshold sample showed good read coverage within the HBVgp1/HBVgp2/HBVgp3 region where coverage abruptly stops. Furthermore, the majority of HBV reads align to the HBVgp3 gene, which encodes the regulatory HBx protein (Additional file 5: Figure S137). Like the above findings of read mapping being primarily limited to viral HPV oncogenes, the HBV mapping data are suggestive of oncogenic viral integration. No virus associations were observed with the 17 recurrent LGGs (Fig. 1 and Additional file 7: File S4).

#### Lack of virus association in the cancer genome atlas GBM whole genome datasets

To explore the possibility that viruses infecting brain tissue become transcriptionally dormant, resulting in the lack of virus detection in RNA-seq datasets, we subjected the TCGA GBM whole genome sequencing (WGS) datasets to our virome analysis pipeline.

**Fig. 2:**
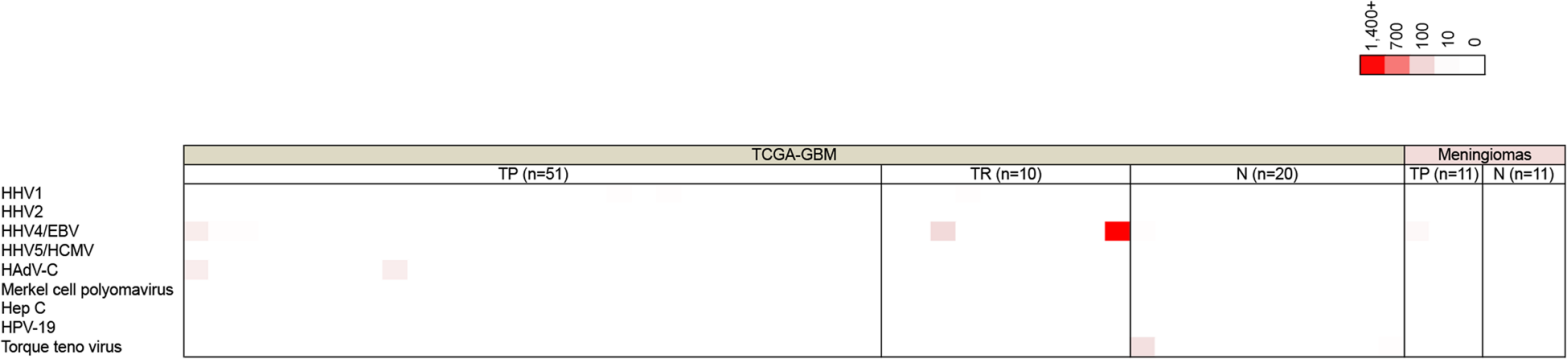
Heat map showing the total reads for brain tissue whole genome sequencing datasets. Color intensity represents relative viral reads across all datasets

Analysis of viruses at the DNA level was relatively unremarkable with the highest read numbers in the primary (TP) GBM WGS datasets being derived from EBV and HAdV-C (Fig. 2 and Additional file 8: File S5). The highest viral read numbers identified in a recurrent (TR) GBM WGS datasets was derived from EBV with 1,454 total viral reads (Fig. 2). Two HCMV viral reads were detected in 1 TP GBM WGS dataset (TCGA-14-1823) but not the corresponding blood matched normal (N) control. Manual blast of these HCMV reads from the tumor demonstrated homology to HCMV laboratory expression vector sequences (Additional file 9: File S6), which was also demonstrated by Tang et al. [27].

EBV reads were detected in 9 TP GBM WGS datasets for which 6 out of 9 samples analyzed had corresponding blood matched samples (Additional file 8: File S5). We detected EBV in 4 additional normal blood samples and 3 TR GBM WGS datasets (Additional file 8: File S5). Upon further analysis of the raw EBV reads from the TP GBM datasets, the viral reads were found to be homologous to the EBV genome based on blasting analysis (Additional file 9: File S6). EBV read coverage analysis for the 3 TR GBM samples displayed viral genome coverage across the entire genome for 2 of the samples (Additional file 5: Figure S138). Torque Teno Virus reads were incidentally identified in three normal blood samples. Finally, although a high level of HHV- 6 and 7 reads were detected in all WGS samples, manual inspection of the raw sequence reads showed that they are likely derived from human chromosomal telomeric-like repeats, TAACCC (data not shown).

#### Lack of virus association in meningioma whole genome datasets

To determine if other brain tumors are associated with viruses, we analyzed 64 WGS datasets from 11 patients with grade I meningiomas and their matched blood control samples. Analysis of the WGS datasets from the meningioma samples demonstrated no confirmed association with viruses (Fig. 2 and Additional file 10: File S7). Similar to our analysis of the TCGA GBM WGS datasets, a low abundance of EBV reads were detected in 3 tumor samples and 4 normal blood samples, raising the possibility that these reads came from infiltrating B-cells. Furthermore, HHV-6 and HHV-7 reads were detected in all 22 samples but consisted of the simple sequence repeat, TAACCC suggesting that these reads likely originated from human telomeric repeats (data not shown).

## Discussion

Although there was an agreement reached from the HCMV/GBM symposium in 2011, emerging studies using NGS to assess the viral association with GBM has been unable to recapitulate this association [9, 10, 14, 26, 27]. In line with these previous studies, our data further supports no direct viral association with GBM. There may be a possible association of HPV-16, HPV-58, and Hepatitis B with LGGs, however additional validation studies are required before any conclusions can be drawn from our initial assessment. Furthermore, based on the low abundance of viral reads that were identified in these cases, whether these viruses are truly associated with LGGs and not derived from sequencing contamination is unclear. Finally, although the viral detection threshold that we set for the RNA-seq datasets is relatively low (0.07 RPMH), all HCMV read findings were analyzed further irrespective of how low the HCMV read level and were found to be likely derived from laboratory plasmid contamination.

The hallmark of herpesviruses, and their key to persistence within their host, is their ability to switch to highly restricted gene expression patterns that allow avoidance of the immune system. To overcome the potential problem of missing viral infections due to this type of viral adaptation, WGS datasets were analyzed. Nevertheless, this approach also failed to identify any meaningful virus associations in the analyzed samples. This is in contrast to a report by Amirian et al. in which they identified HHV-6A and HHV-6B in the WGS datasets from TCGA [5]. Another study conducted by Cimino et al. also identified HHV-6 and EBV DNA when they analyzed unmapped reads from a NGS-based comprehensive oncology panel [9]. Although our initial investigation detected the presence of HHV-6 and HHV-7 viruses, further analysis of these viral reads revealed all reads consisted of human chromosomal telomeric-like repeats, TAACCC. Although HHV 6 and 7 have sequences homologous to this region, no other regions of the viral genome were represented in the sequence datasets. This is highly suggestive that these reads originated from the telomeric region of human chromosomes rather than representing *bona fide* HHV6 or HHV7 infection.

EBV DNA reads were identified in a number of the TCGA DNA-seq datasets including 9 TP GBM WGS samples, 6 normal matched blood WGS samples plus 4 additional normal blood WGS samples, and 3 TR GBM WGS samples. In addition, we identified EBV DNA reads in 3 grade I meningioma samples and 4 normal blood samples. All EBV DNA reads identified were low in abundance with 1 – 39 reads detected in primary GBM samples, 1–5 reads detected in normal blood samples, and 1 – 15 reads detected in grade 1 meningiomas, a result similar to the findings of Cimino et al. in which they identified 1 – 18 EBV reads in 5 GBM samples [9]. We identified 3 TR GBM samples using WGS datasets that were EBV positive, with 1 of these datasets showing moderate EBV levels (1454 viral reads), another showing minimal EBV levels (80 viral reads), and the last dataset had 1 EBV viral read. Although these three TR GBM WGS datasets were positive for EBV, the corresponding RNA-seq datasets for these samples failed to validate these findings. Without tissue to confirm these findings, it is impossible to determine the origin of these viral reads and we do not feel confident in associating EBV with these TR GBM samples. In addition, based on our past experience in the field of EBV, if EBV was truly associated, we would likely see greater than 10 viral RPMH for RNA-seq and thousands of viral reads for DNA-seq [32, 53]. Finally, given the ubiquitous nature of EBV, the low viral read counts, and the presence of EBV in both tumor and blood samples in relatively equal proportions, we postulate that the EBV reads that were detected likely originated from EBV infected B-cells localized in the tumor stroma and/or from library or sequencing sample cross-contamination.

Due to the nature of GBM, there is a possibility for a preponderance of necrotic tissue within the tumor bulk, resulting in the effective dilution of tumor cells and tumor associated viruses; which could be argued as an explanation for the lack of strong viral detection. However, given the large number of samples analyzed and the careful procurement protocols utilized by TCGA, it is unlikely that the majority of samples fall within this scenario. Further supporting this contention, our analysis of the MRI-localized GBM biopsies from Gill et al. [43] did not detect any known viruses and there were no differences between samples obtained from the core (presumably more necrotic) and those samples obtained from the tumor margin (presumably less necrotic, with active tumor growth and neoangiogenesis).

The identification of HPV-16, HPV-58 and HBV in a small portion of LGG RNA-seq datasets is a potentially interesting finding. Analysis of the clinical data from these patients using cBioPortal [58, 59] demonstrated that the majority of virus positive samples were oligodendrogliomas (3 out of the 5 samples) from White males with an average age of 42 (Additional file 11: File S8). The demographics are relatively consistent with the whole LGG cohort (55 % males, 92 % White, and average age 43). Tumor type varied slightly from the whole cohort, which consisted of 193 astrocytomas (38), 130 oligoastrocytomas (25), and 191 oligodendrogliomas (37 %). In addition, although the genetic profile of these patients demonstrates a variety of alterations, some of the more common alterations observed in the entire cohort (e.g., IDH1, IDH2, ATRX, and TP53) were not observed in these patients with HPV or HBV reads (see reference [39] for additional details regarding LGG samples). The lack of mutation of one or more of these in tumors with detected virus could be due to viral subversion of the corresponding pathways, obviating the need for somatic mutations (for example, through HPV E6 mediated inhibition of the p53 pathway). Nevertheless, further investigation into the association between viruses, HPV and HBV and LGGs is warranted.

Both HPV-16 and HPV-58 are considered high-risk HPV types, which are causative agents in the development of cervical carcinoma. The likely mechanism of action for both HPV-16 and HPV-58 is viral integration into the host genome [60, 61]. Coverage analysis of the HPV positive LGG datasets indicate that some of the samples display evidence of integration with disruption of the viral E1 gene (Additional file 5: Figures S135-136) with all samples with HPV reads showing the majority of read coverage mapping to viral E6 and E7 oncogenes. Due to the low viral read numbers detected in our study, additional validation experiments are warranted to determine if there is truly an association between LGGs and HPV or whether these findings represent sample cross-contamination with true HPV associated samples.

Like HPV, the mechanism of action for HBV is also integration into the host genome. Visual analysis of the HBV positive LGG datasets demonstrated robust gene coverage within the HBVgp1/HBVgp2/HBVgp3 region with an abrupt termination of gene coverage after HBVgp3 (Additional file 5: Figure S137). Further, the majority of reads align within the HBVgp3 gene, which encodes the regulatory HBx protein. Previous studies have shown that HBx plays a critical role in the pathogenesis of hepatocellular carcinoma [62, 63]. While this observation is also of potential interest, given the fact that adequate HBV reads were detected in only 1 sample out of 514 LGG datasets (0.19 %), further analysis is necessary to validate this observation.

The RNA CoMPASS analysis of the auxiliary brain tissue sequencing datasets provided a full metatranscriptomic profile including bacterial, fungal, and viral reads. Although we only presented data on the virome in this study, a complete metatranscriptomic analysis was performed. Although reads for several bacterial species were identified in the datasets, it has been our experience that the source of many of these reads are from environmental contamination [33, 34, 64] and do not represent true associations.

Due to reports of an association between HCMV and GBM, immunotherapy treatments against HCMV were considered a logical next step as an exhilarating new avenue for cancer therapy. There are several clinical trials in the United States in various stages of completion focused on targeted HCMV therapy in GBM patients. While we await the results of these clinical trials, the results from the valganciclovir treatment of glioblastoma patients in Sweden (VIGAS) study, a randomized, double-blinded, placebo-controlled trial was recently published showing trends but no significant differences in tumor volumes between the valganciclovir (an anti-CMV drug) and placebo groups at 3 and 6 months [65]. However, in a retrospective analysis of the same cohort with additional patients taking valganciclovir for compassionate reasons, the rate of survival of treated patients at 2 years was 62 % as compared with 18 % of contemporary controls with a similar disease stage, surgical-resection grade, and baseline treatment [66]. Although these are remarkable results, questions have been raised as to the interpretation of the data and whether this survival rate is misleading [67].

## Conclusions

Several recent publications have highlighted the lack of association between viruses, specifically HCMV, and GBM [6, 10, 14, 15, 18, 26–28, 68]. Based on our comprehensive analysis, we substantiate these claims. Given the austerity of recent evidence against a HCMV etiology for GBM, moving forward, we caution against the use of anti-CMV therapy for GBM patients until this issue is completely resolved.

## Additional files

Additional file 1: Table S1. Sequencing Datasets. (DOC 45 kb) Additional file 2: Table S2. List of PCR primers. (DOC 36 kb) Additional file 3: File S1. TCGA GBM RNA. (XLS 374 kb) Additional file 4: File S2. HCMV analysis. (XLSX 28 kb) Additional file 5: Supplementary Figures 1-138. (PDF 17.9 mb) Additional file 6: File S3 Other studies. (XLS 3154 kb) Additional file 7: File S4 TCGA LGG RNA. (XLSX 546 kb) Additional file 8: File S5 TCGA GBM DNA. (XLS 2545 kb) Additional file 9: File S6 Viral Blast analysis. (XLS 110 kb) Additional file 10: File S7 WGS meningioma. (XLS 61 kb) Additional file 11: File S8 LGG clinical data. (XLSX 38 kb)
