# Supplementary material for: A comprehensive next generation sequencing-based virome assessment in brain tissue suggests no major virus - tumor association
Source: Acta Neuropathol Commun. 2016 Jul 11;4:71. doi: 10.1186/s40478-016-0338-z (PMC4940872; doi:10.1186/s40478-016-0338-z)
Supplement: Additional file 1: Table S1. — Sequencing Datasets. (DOC 45 kb) [file 40478_2016_338_MOESM1_ESM.doc]

**Additional file 1: Table S1.** Sequencing Datasets

| **Dataset** | **Disease** | **Analyte Type** | **Library Type** | **Number of Samples** | **Accession Number** |
| --- | --- | --- | --- | --- | --- |
| **TCGA-GBM** | GBM | Total RNA (polyA) | RNA-seq | 157 |  |
| **TCGA-GBM** | GBM (recurrence) | Total RNA (polyA) | RNA-seq | 13 |  |
| **TCGA-GBM** | Normal Brain | Total RNA (polyA) | RNA-seq | 5 |  |
| **TCGA-GBM** | GBM | DNA | WGS | 51 |  |
| **TCGA-GBM** | GBM  (recurrence) | DNA | WGS | 10 |  |
| **TCGA-GBM** | Normal Blood | DNA | WGS | 20 |  |
| **TCGA-LGG** | LGG | Total RNA (polyA) | RNA-seq | 514 |  |
| **TCGA-LGG** | LGG (recurrence) | Total RNA (polyA) | RNA-seq | 17 |  |
| **00RTS3** | GBM | Total RNA (polyA) | RNA-seq | 1 |  |
| **CAURPRVE** | GBM | Total RNA (ribodepleted) | RNA-seq | 1 |  |
| **H8CPFRSJ** | GBM | Total RNA (ribodepleted) | RNA-seq | 1 |  |
| **GBM** | GBM | Total RNA (polyA) | RNA-seq | 1 GBM  1 Normal | SRP009144 |
| **BodyMap** | Normal Brain | Total RNA (polyA) | RNA-seq | 1 |  |
| **Glioma stem cells** | GBM |  | RNA-seq | 9 | SRP016798 |
| **MRI-localized biopsy** | GBM | Total RNA  (polyA) | RNA-seq | 39 CE  36 NE  17 NB | SRP044668 |
| **Meningioma** | Meningioma | DNA | WGS | 11 tumor  11 blood | SRP016129 |
| **HCMV** | Fibroblast cell line | Total RNA  (polyA) | RNA-seq | 3 | SRP016143 |

CE: contrast-enhancing glioma core samples, NE: non-enhancing FLAIR glioma margin samples, NB: non-neoplastic brain tissue, HCMV: human cytomegalovirus
