# Supplementary material for: A comprehensive next generation sequencing-based virome assessment in brain tissue suggests no major virus - tumor association
Source: Acta Neuropathol Commun. 2016 Jul 11;4:71. doi: 10.1186/s40478-016-0338-z (PMC4940872; doi:10.1186/s40478-016-0338-z)
Supplement: Additional file 2: Table S2. — List of PCR primers. (DOC 36 kb) [file 40478_2016_338_MOESM2_ESM.doc]

**Additional file 2: Table S**2. List of PCR primers

| **Primer** | **Forward Primer** | **Reverse Primer** |
| --- | --- | --- |
| **EBV (EBER1)^1^** | 5’- GGACCTACGCTGCCCTAGA -3’ | 5’- CAGCTGGTACTTGACCGAAGA -3’ |
| **HCMV^1^** | 5’- taatacaagccatccaca -3’ | 5’- tagataaggttcatgagcct -3’ |
| **MuLV^2^** | 5’- CTGAGAGAAGTCAACAAGCG -3’ | 5’- CTGGATCTCTCCACTCAAAGGC -3’ |
| **GAPDH** | 5’- gccaaaagggtcatcatctc -3’ | 5’- ggggccatccacagtcttct -3’ |

**References**

**1.** Bergallo M, Costa C, Terlizzi M, et al. Evaluation of Two Set of Primers for Detection of Immediate Early Gene UL123 of Human Cytomegalovirus (HCMV). *Mol Biotechnol.* 2008; 38(1):65-70.

**2.** Lin Z, Puetter A, Coco J, et al. Detection of Murine Leukemia Virus in the Epstein-Barr Virus-Positive Human B-Cell Line JY, Using a Computational RNA-Seq-Based Exogenous Agent Detection Pipeline, PARSES. *Journal of Virology.* 2012; 86(6):2970-2977.
