# Supplementary material for: A comprehensive next generation sequencing-based virome assessment in brain tissue suggests no major virus - tumor association
Source: Acta Neuropathol Commun. 2016 Jul 11;4:71. doi: 10.1186/s40478-016-0338-z (PMC4940872; doi:10.1186/s40478-016-0338-z)

**Supplemental Fig S1.** Representative graphical of taxonomic analysis of sample, 00RTS3.

**Supplemental Fig S2.** Representative graphical of taxonomic analysis of sample, SRR359291.

**Supplemental Fig S3.** Representative graphical of taxonomic analysis of sample, SRR359290.

**Supplemental Fig S4.** Representative graphical of taxonomic analysis of a normal brain sample from the BodyMap initiative.

**Supplemental Fig S5.** Representative graphical of taxonomic analysis of sample, CAUPRVE.

**Supplemental Fig S6.** Representative graphical of taxonomic analysis of sample, H8CPFRSJ.

**Supplemental Fig S7.** EBV and MuLV gene coverage analysis on RNA-seq datasets from GBM samples and JY cell line

**Supplemental Fig S8.** Gene expression profile of several tissue samples. These included a fibroblast-derived cell line, fibroblast-derived cell line infected with HCMV, JY cell line, and 2 primary GBMs. Relative viral mRNA expression (-fold) is represented as red (HCMV), purple (MuLV), and blue (EBV) columns and are the fold difference compared to the fibroblast-derived cell line values.

**Supplemental Fig S9.** Representative graphical of taxonomic analysis of sample, SRR1521352.

**Supplemental Fig S10.** Representative graphical of taxonomic analysis of sample, SRR1521353.

**Supplemental Fig S11.** Representative graphical of taxonomic analysis of sample, SRR1521354.

**Supplemental Fig S12.** Representative graphical of taxonomic analysis of sample, SRR1521355.

**Supplemental Fig S13.** Representative graphical of taxonomic analysis of sample, SRR1521356.

**Supplemental Fig S14.** Representative graphical of taxonomic analysis of sample, SRR1521357.

**Supplemental Fig S15.** Representative graphical of taxonomic analysis of sample, SRR1521358.

**Supplemental Fig S16.** Representative graphical of taxonomic analysis of sample, SRR1521359.

**Supplemental Fig S17.** Representative graphical of taxonomic analysis of sample, SRR1521360.

**Supplemental Fig S18.** Representative graphical of taxonomic analysis of sample, SRR1521361.

**Supplemental Fig S19.** Representative graphical of taxonomic analysis of sample, SRR1521362.

**Supplemental Fig S20.** Representative graphical of taxonomic analysis of sample, SRR1521363.

**Supplemental Fig S21.** Representative graphical of taxonomic analysis of sample, SRR1521364.

**Supplemental Fig S22.** Representative graphical of taxonomic analysis of sample,

SRR1521365.

**Supplemental Fig S23.** Representative graphical of taxonomic analysis of sample, SRR1521366.

**Supplemental Fig S24.** Representative graphical of taxonomic analysis of sample, SRR1521367.

**Supplemental Fig S25.** Representative graphical of taxonomic analysis of sample, SRR1521368.

**Supplemental Fig S26.** Representative graphical of taxonomic analysis of sample, SRR1521369.

**Supplemental Fig S27.** Representative graphical of taxonomic analysis of sample, SRR1521370.

**Supplemental Fig S28.** Representative graphical of taxonomic analysis of sample, SRR1521371.

**Supplemental Fig S29.** Representative graphical of taxonomic analysis of sample, SRR1521372.

**Supplemental Fig S30.** Representative graphical of taxonomic analysis of sample, SRR1521373.

**Supplemental Fig S31.** Representative graphical of taxonomic analysis of sample, SRR1521374.

**Supplemental Fig S32.** Representative graphical of taxonomic analysis of sample, SRR1521375.

**Supplemental Fig S33.** Representative graphical of taxonomic analysis of sample, SRR1521376.

**Supplemental Fig S34.** Representative graphical of taxonomic analysis of sample, SRR1521377.

**Supplemental Fig S35.** Representative graphical of taxonomic analysis of sample, SRR1521378.

**Supplemental Fig S36.** Representative graphical of taxonomic analysis of sample, SRR1521379.

**Supplemental Fig S37.** Representative graphical of taxonomic analysis of sample, SRR1521380.

**Supplemental Fig S38.** Representative graphical of taxonomic analysis of sample, SRR1521381.

**Supplemental Fig S39.** Representative graphical of taxonomic analysis of sample, SRR1521382.

**Supplemental Fig S40.** Representative graphical of taxonomic analysis of sample, SRR1521383.

**Supplemental Fig S41.** Representative graphical of taxonomic analysis of sample, SRR1521384.

**Supplemental Fig S42.** Representative graphical of taxonomic analysis of sample, SRR1521385.

**Supplemental Fig S43.** Representative graphical of taxonomic analysis of sample, SRR1521386.

**Supplemental Fig S44.** Representative graphical of taxonomic analysis of sample, SRR1521387.

**Supplemental Fig S45.** Representative graphical of taxonomic analysis of sample,

SRR1521388.

**Supplemental Fig S46.** Representative graphical of taxonomic analysis of sample, SRR1521389.

**Supplemental Fig S47.** Representative graphical of taxonomic analysis of sample, SRR1521390.

**Supplemental Fig S48.** Representative graphical of taxonomic analysis of sample, SRR1521391.

**Supplemental Fig S49.** Representative graphical of taxonomic analysis of sample, SRR1521392.

**Supplemental Fig S50.** Representative graphical of taxonomic analysis of sample, SRR1521393.

**Supplemental Fig S51.** Representative graphical of taxonomic analysis of sample, SRR1521394.

**Supplemental Fig S52.** Representative graphical of taxonomic analysis of sample, SRR1521395.

**Supplemental Fig S53.** Representative graphical of taxonomic analysis of sample, SRR1521396.

**Supplemental Fig S54.** Representative graphical of taxonomic analysis of sample, SRR1521397.

**Supplemental Fig S55.** Representative graphical of taxonomic analysis of sample, SRR1521398.

**Supplemental Fig S56.** Representative graphical of taxonomic analysis of sample, SRR1521399.

**Supplemental Fig S57.** Representative graphical of taxonomic analysis of sample, SRR1521400.

**Supplemental Fig S58.** Representative graphical of taxonomic analysis of sample, SRR1521401.

**Supplemental Fig S59.** Representative graphical of taxonomic analysis of sample, SRR1521402.

**Supplemental Fig S60.** Representative graphical of taxonomic analysis of sample, SRR1521403.

**Supplemental Fig S61.** Representative graphical of taxonomic analysis of sample, SRR1521404.

**Supplemental Fig S62.** Representative graphical of taxonomic analysis of sample, SRR1521405.

**Supplemental Fig S63.** Representative graphical of taxonomic analysis of sample, SRR1521406.

**Supplemental Fig S64.** Representative graphical of taxonomic analysis of sample, SRR1521407.

**Supplemental Fig S65.** Representative graphical of taxonomic analysis of sample, SRR1521408.

**Supplemental Fig S66.** Representative graphical of taxonomic analysis of sample, SRR1521409.

**Supplemental Fig S67.** Representative graphical of taxonomic analysis of sample, SRR1521410.

**Supplemental Fig S68.** Representative graphical of taxonomic analysis of sample,

SRR1521411.

**Supplemental Fig S69.** Representative graphical of taxonomic analysis of sample, SRR1521412.

**Supplemental Fig S70.** Representative graphical of taxonomic analysis of sample, SRR1521413.

**Supplemental Fig S71.** Representative graphical of taxonomic analysis of sample, SRR1521414.

**Supplemental Fig S72.** Representative graphical of taxonomic analysis of sample, SRR1521415.

**Supplemental Fig S73.** Representative graphical of taxonomic analysis of sample, SRR1521416.

**Supplemental Fig S74.** Representative graphical of taxonomic analysis of sample, SRR1521417.

**Supplemental Fig S75.** Representative graphical of taxonomic analysis of sample, SRR1521418.

**Supplemental Fig S76.** Representative graphical of taxonomic analysis of sample, SRR1521419.

**Supplemental Fig S77.** Representative graphical of taxonomic analysis of sample, SRR1521420.

**Supplemental Fig S78.** Representative graphical of taxonomic analysis of sample, SRR1521421.

**Supplemental Fig S79.** Representative graphical of taxonomic analysis of sample, SRR1521422.

**Supplemental Fig S80.** Representative graphical of taxonomic analysis of sample, SRR1521423.

**Supplemental Fig S81.** Representative graphical of taxonomic analysis of sample, SRR1521424.

**Supplemental Fig S82.** Representative graphical of taxonomic analysis of sample, SRR1521425.

**Supplemental Fig S83.** Representative graphical of taxonomic analysis of sample, SRR1521426.

**Supplemental Fig S84.** Representative graphical of taxonomic analysis of sample, SRR1521427.

**Supplemental Fig S85.** Representative graphical of taxonomic analysis of sample, SRR1521428.

**Supplemental Fig S86.** Representative graphical of taxonomic analysis of sample, SRR1521429.

**Supplemental Fig S87.** Representative graphical of taxonomic analysis of sample, SRR1521430.

**Supplemental Fig S88.** Representative graphical of taxonomic analysis of sample, SRR1521431.

**Supplemental Fig S89.** Representative graphical of taxonomic analysis of sample, SRR1521432.

**Supplemental Fig S90.** Representative graphical of taxonomic analysis of sample, SRR1521433.

**Supplemental Fig S91.** Representative graphical of taxonomic analysis of sample,

SRR1521434.

**Supplemental Fig S92.** Representative graphical of taxonomic analysis of sample, SRR1521435.

**Supplemental Fig S93.** Representative graphical of taxonomic analysis of sample, SRR1521436.

**Supplemental Fig S94.** Representative graphical of taxonomic analysis of sample, SRR1521437.

**Supplemental Fig S95.** Representative graphical of taxonomic analysis of sample, SRR1521438.

**Supplemental Fig S96.** Representative graphical of taxonomic analysis of sample, SRR1521439.

**Supplemental Fig S97.** Representative graphical of taxonomic analysis of sample, SRR1521440.

**Supplemental Fig S98.** Representative graphical of taxonomic analysis of sample, SRR1521441.

**Supplemental Fig S99.** Representative graphical of taxonomic analysis of sample, SRR1521442.

**Supplemental Fig S100.** Representative graphical of taxonomic analysis of sample, SRR1521443.

**Supplemental Fig S101.** Representative graphical of taxonomic analysis of sample, SRR1521444.

**Supplemental Fig S102.** Representative graphical of taxonomic analysis of sample, SRR1521445.

**Supplemental Fig S103.** HIV gene coverage analysis for MRI-localized biopsies

**Supplemental Fig S104.** MuLV gene coverage analysis for MRI-localized non-enhancing biopsies

**Supplemental Fig S105.** MuLV gene coverage analysis for MRI-localized contrast biopsies

**Supplemental Fig S106.** MuLV gene coverage analysis for MRI-localized non-neoplasm biopsies

**Supplemental Fig S107.** HCMV gene coverage analysis for MRI-localized non-enhancing biopsies

**Supplemental Fig S108.** HCMV gene coverage analysis for MRI-localized contrast biopsies

**Supplemental Fig S109.** Representative graphical of taxonomic analysis of sample, SRR608893.

**Supplemental Fig S110.** Representative graphical of taxonomic analysis of sample, SRR608894.

**Supplemental Fig S111.** Representative graphical of taxonomic analysis of sample, SRR608895.

**Supplemental Fig S112.** Representative graphical of taxonomic analysis of sample, SRR608896.

**Supplemental Fig S113.** Representative graphical of taxonomic analysis of sample, SRR608897.

**Supplemental Fig S114.** Representative graphical of taxonomic analysis of sample, SRR608898.

**Supplemental Fig S115.** Representative graphical of taxonomic analysis of sample, SRR608899.

**Supplemental Fig S116.** Representative graphical of taxonomic analysis of sample, SRR608900.

**Supplemental Fig S117.** Representative graphical of taxonomic analysis of sample, SRR608901.

**Supplemental Fig S118.** Representative graphical of taxonomic analysis of sample, SRR942849.

**Supplemental Fig S119.** Representative graphical of taxonomic analysis of sample, SRR942850.

**Supplemental Fig S120.** Representative graphical of taxonomic analysis of sample, SRR942851.

**Supplemental Fig S121.** Representative graphical of taxonomic analysis of sample, SRR942852.

**Supplemental Fig S122.** Representative graphical of taxonomic analysis of sample, SRR942853.

**Supplemental Fig S123.** Representative graphical of taxonomic analysis of sample, SRR942854.

**Supplemental Fig S124.** Representative graphical of taxonomic analysis of sample, SRR942855.

**Supplemental Fig S125.** Representative graphical of taxonomic analysis of sample, SRR942856.

**Supplemental Fig S126.** Representative graphical of taxonomic analysis of sample,

SRR942857.

**Supplemental Fig S127.** Representative graphical of taxonomic analysis of sample, SRR942858.

**Supplemental Fig S128.** Representative graphical of taxonomic analysis of sample, SRR942859.

**Supplemental Fig S129.** Representative graphical of taxonomic analysis of sample, SRR942860.

**Supplemental Fig S130.** Representative graphical of taxonomic analysis of sample, SRR942861.

**Supplemental Fig S131.** Representative graphical of taxonomic analysis of sample, SRR942862.

**Supplemental Fig S132.** Representative graphical of taxonomic analysis of sample, SRR942863.

**Supplemental Fig S133.** Human Adenovirus C gene coverage analysis for gliomas stem cell samples

**Supplemental Fig S134.** HCMV gene coverage analysis for gliomas stem cell samples

**Supplemental Fig S135.** HPV-16 gene coverage analysis for low grade gliomas

**Supplemental Fig S136.** HPV-58 gene coverage analysis for low grade gliomas

**Supplemental Fig S137.** Hepatitis B gene coverage analysis for low grade gliomas

**Supplemental Fig S138.** WGS of EBV gene coverage for recurrent GBM samples

Supplemental Figure S1: 00RTS3

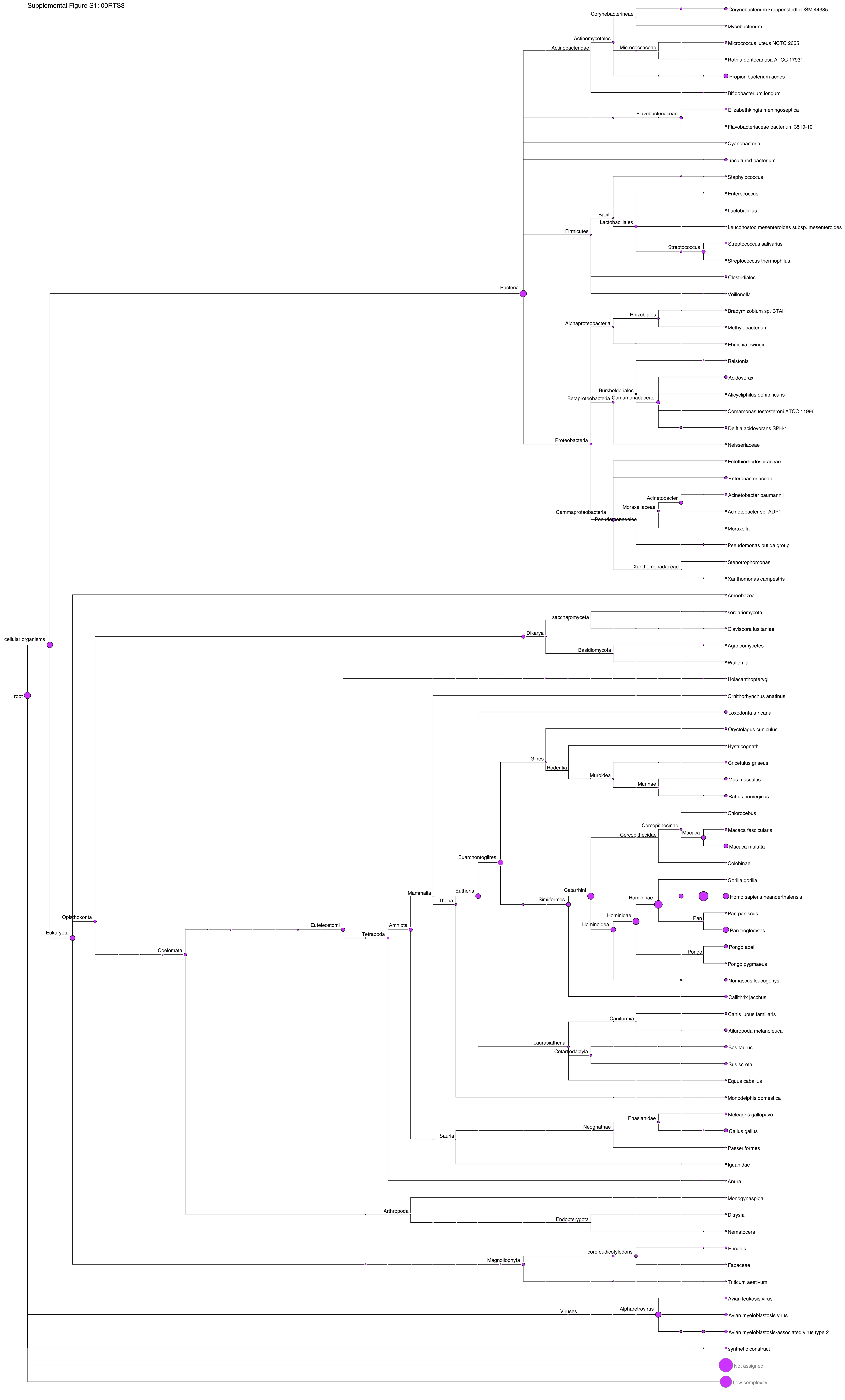

Supplemental Figure S2: SRR359291

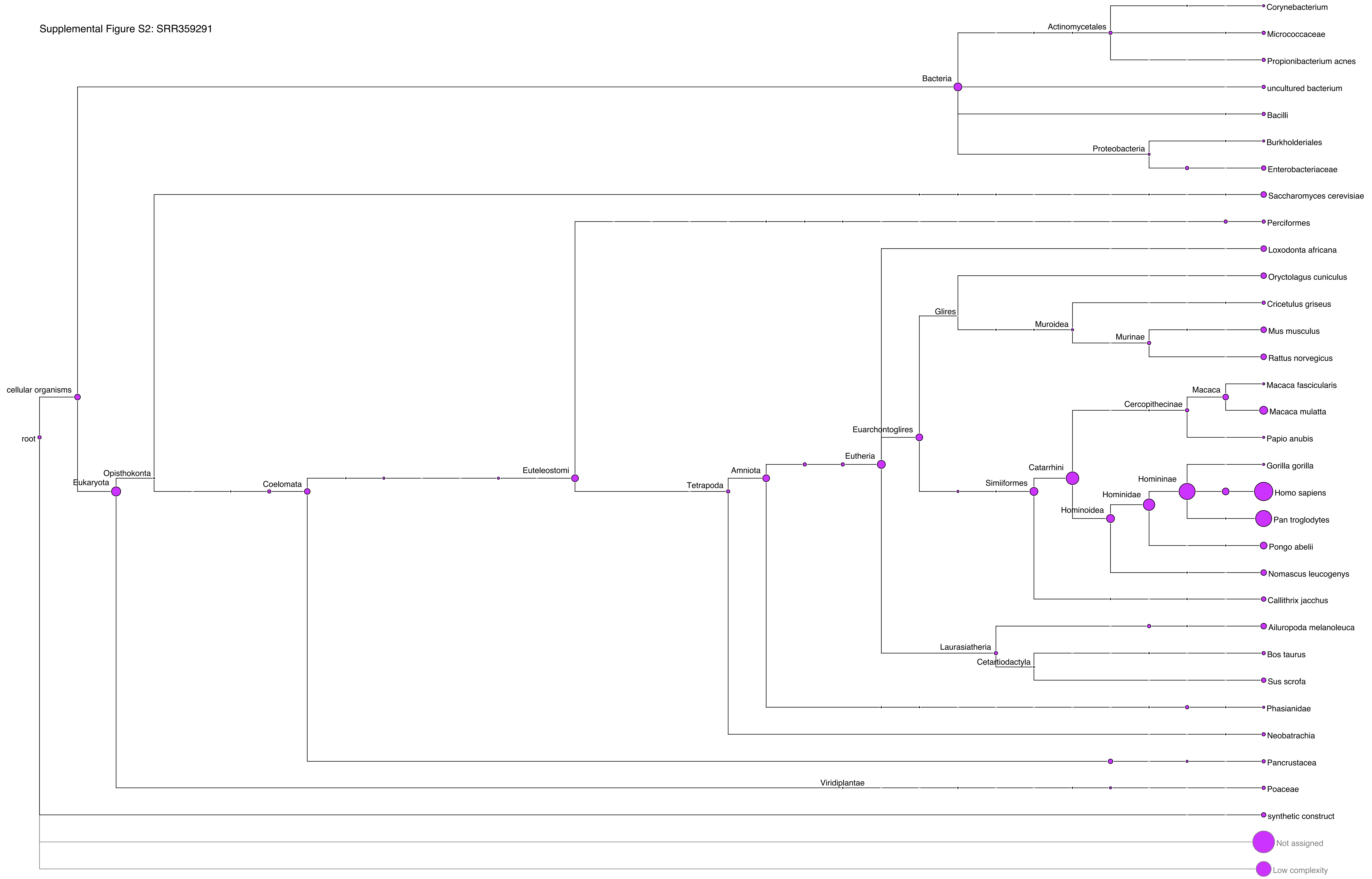

Supplemental Figure S3: SRR359290

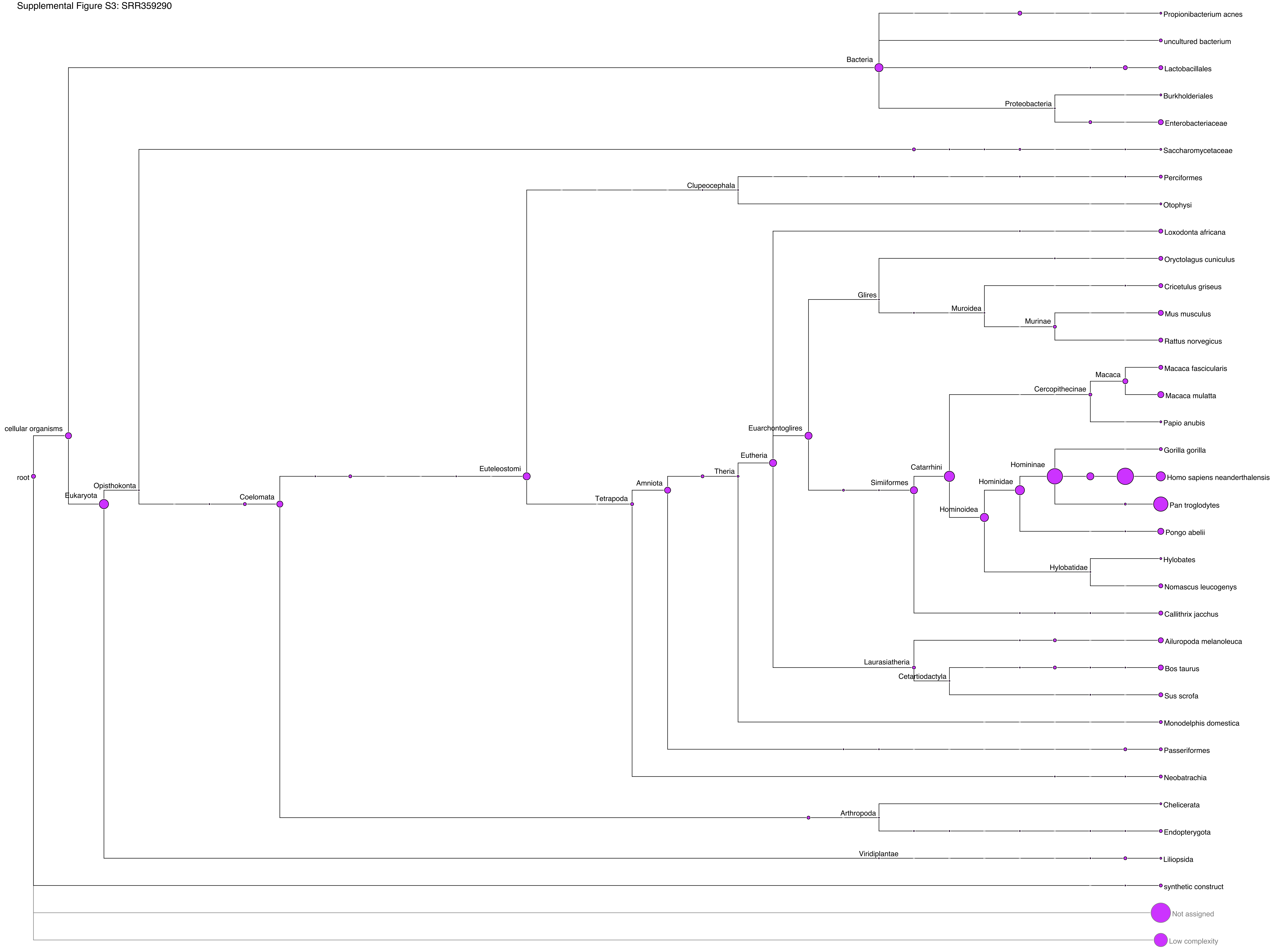

Supplemental Figure S4: Normal Brain Tissue BodyMap

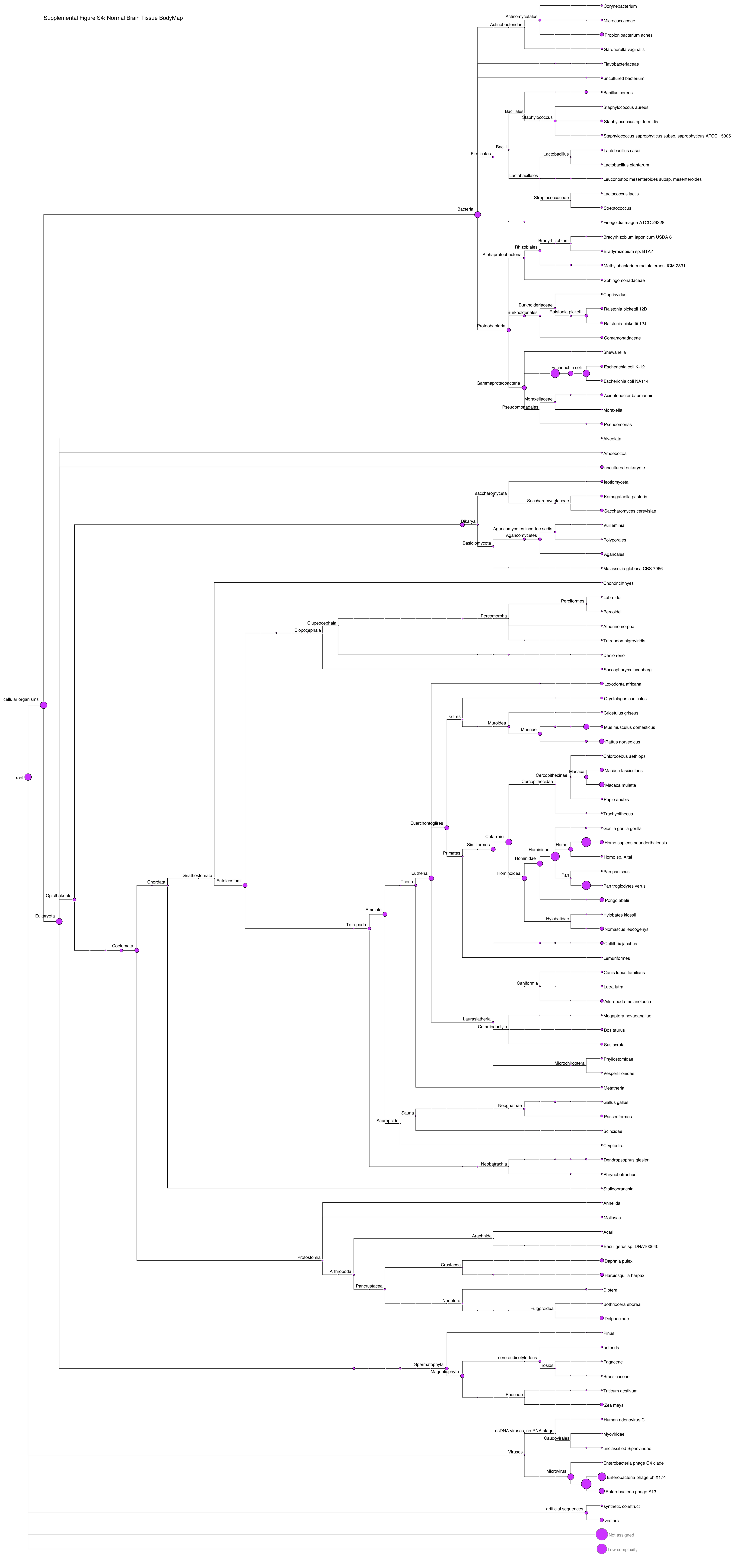

Supplemental Figure S5: CAUPRVE

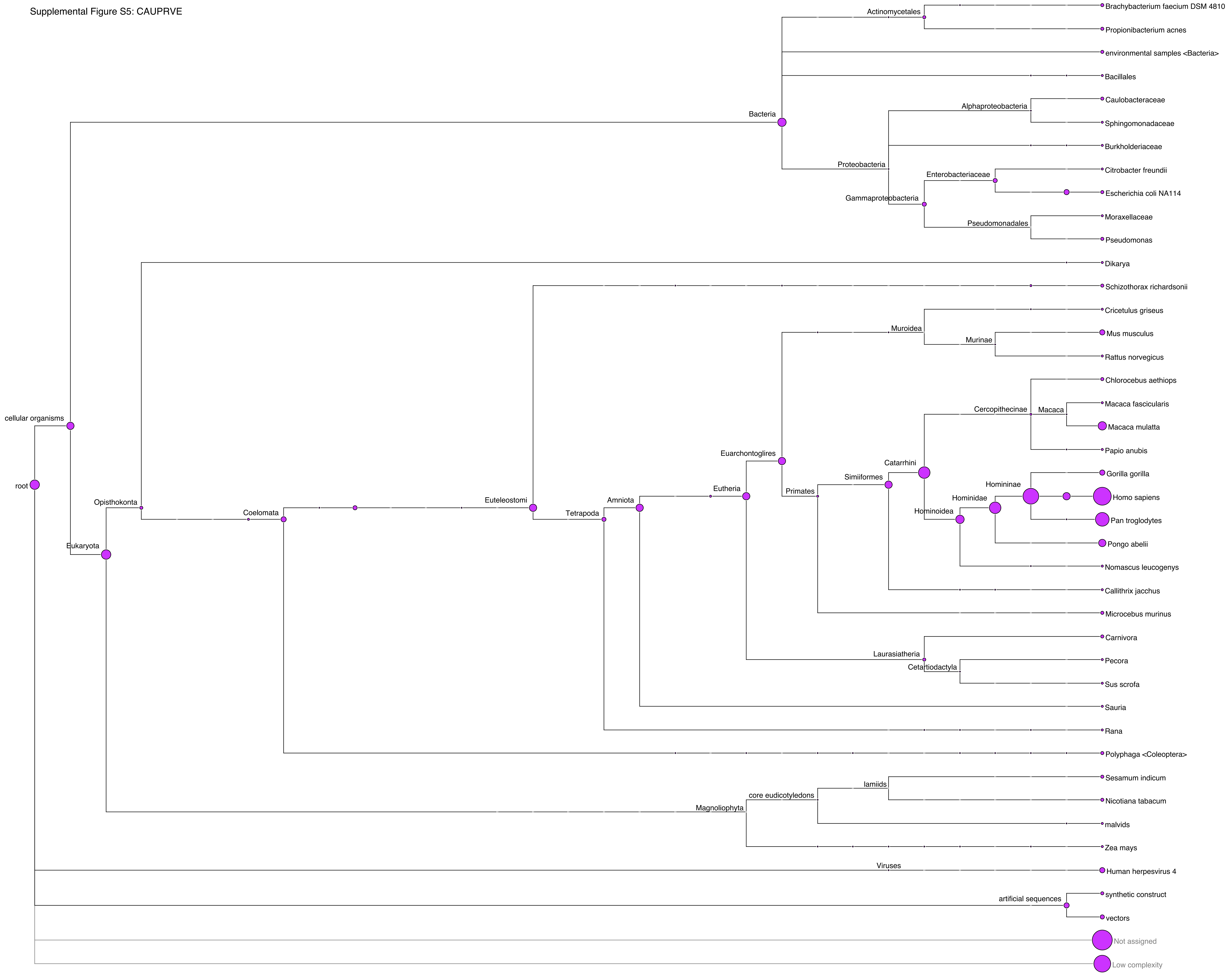

Supplemental Figure S6: H8CPFRSJ

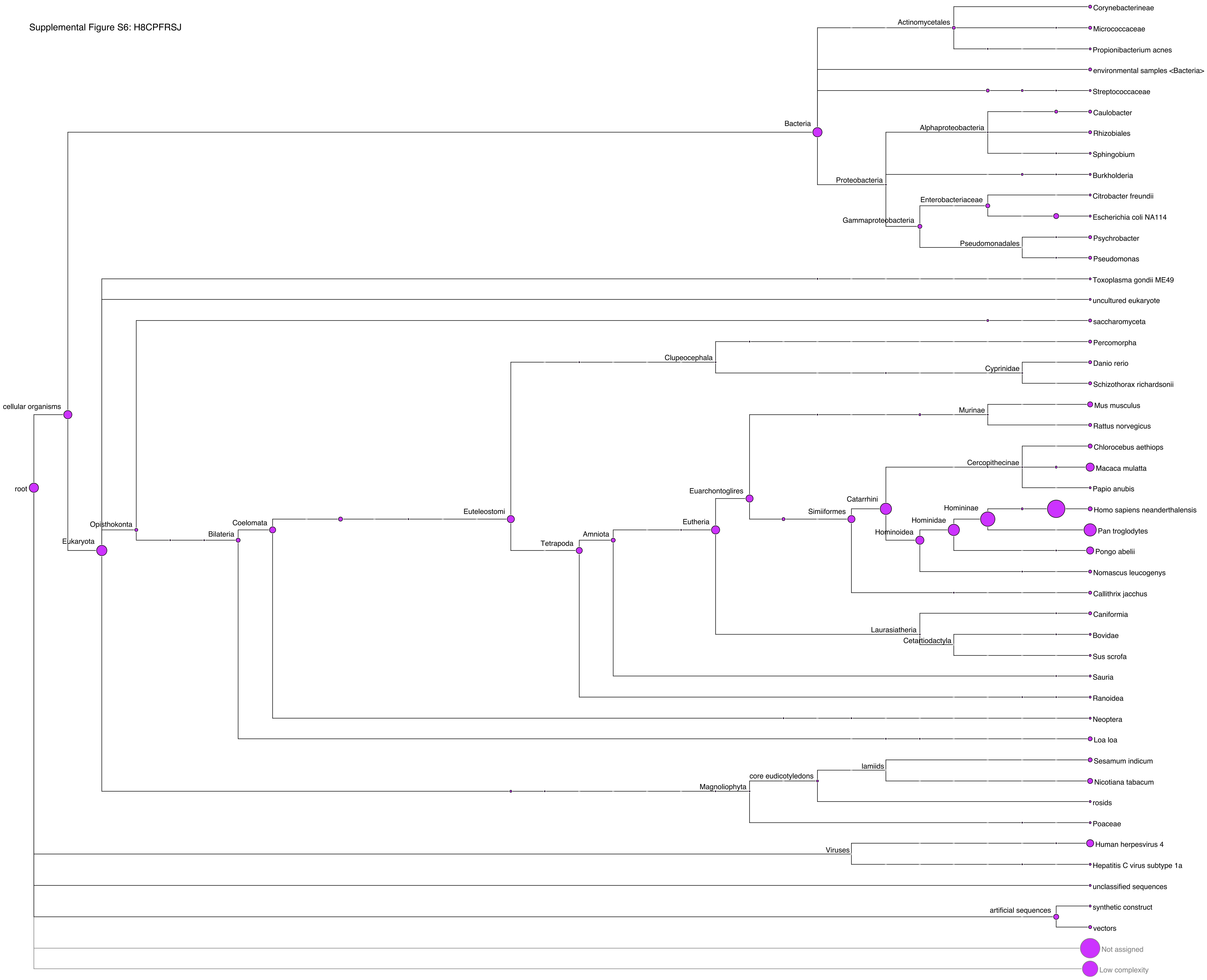

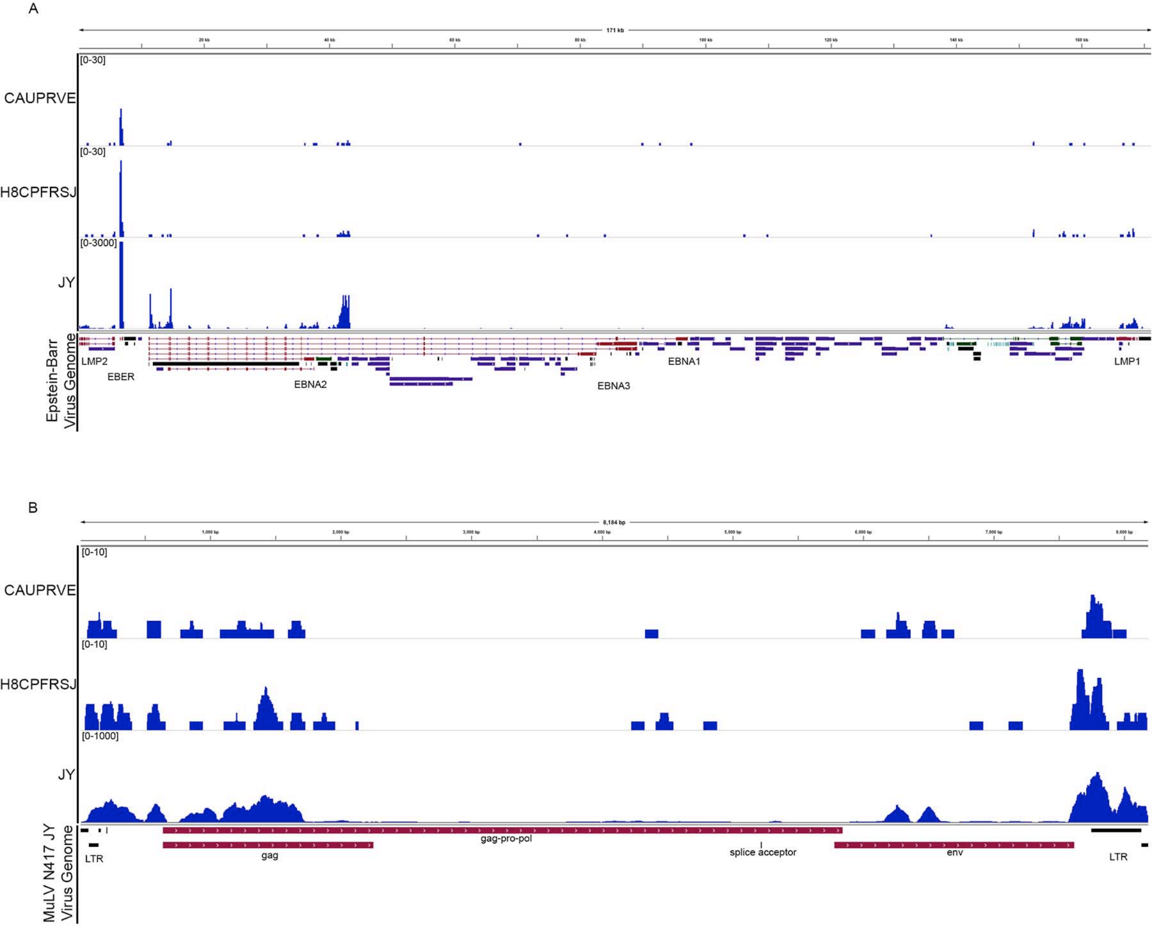

A
171 kb
CAUPRVE
[0-30]
H8CPFRSJ
[0-30]
JY
[0-3000]
Epstein-Barr Virus Genome
LMP2
EBER
EBNA2
EBNA3
EBNA1
LMP1
B
8,184 bp
CAUPRVE
[0-10]
H8CPFRSJ
[0-10]
JY
[0-1000]
MuLV N417 JY Virus Genome
LTR
gag-pro-pol
gag
splice acceptor
env
LTR

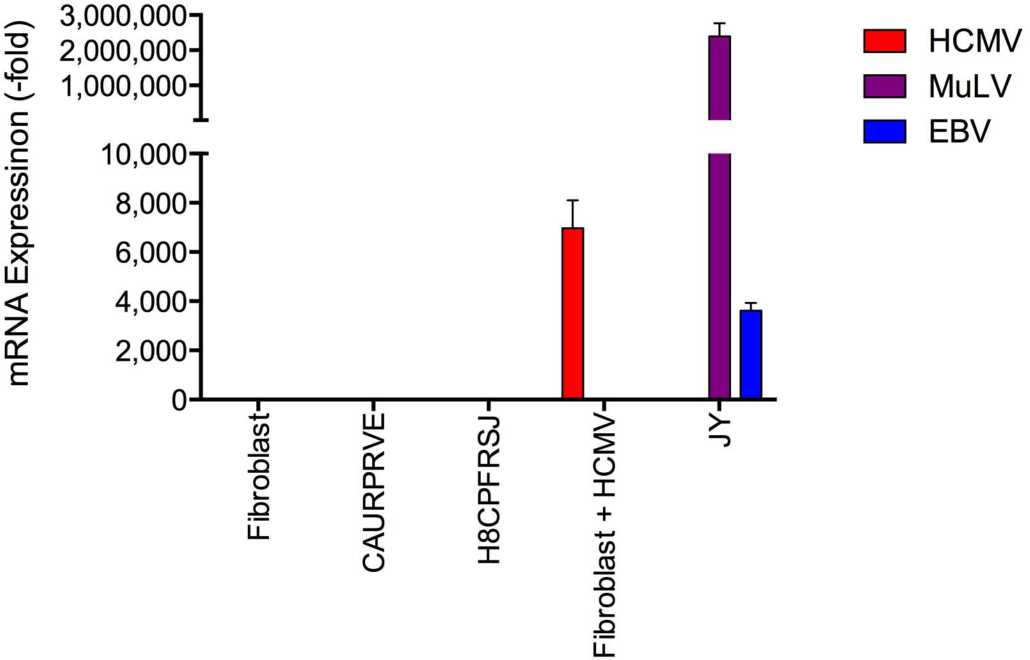

mRNA Expressinon (-fold)
3,000,000
2,000,000
1,000,000
10,000
8,000
6,000
4,000
2,000
0
Fibroblast
CAURPRVE
H8CPFRSJ
Fibroblast + HCMV
JY
HCMV
MuLV
EBV

Supplemental Figure S9: SRR1521352

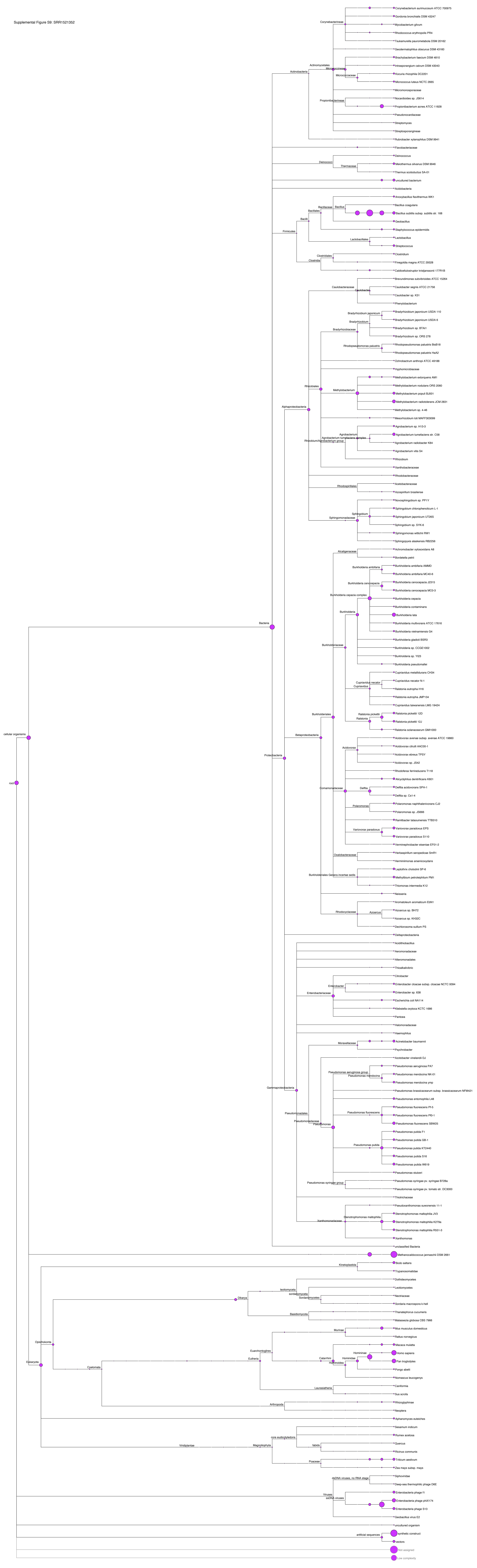

Supplemental Figure S10: SRR1521353

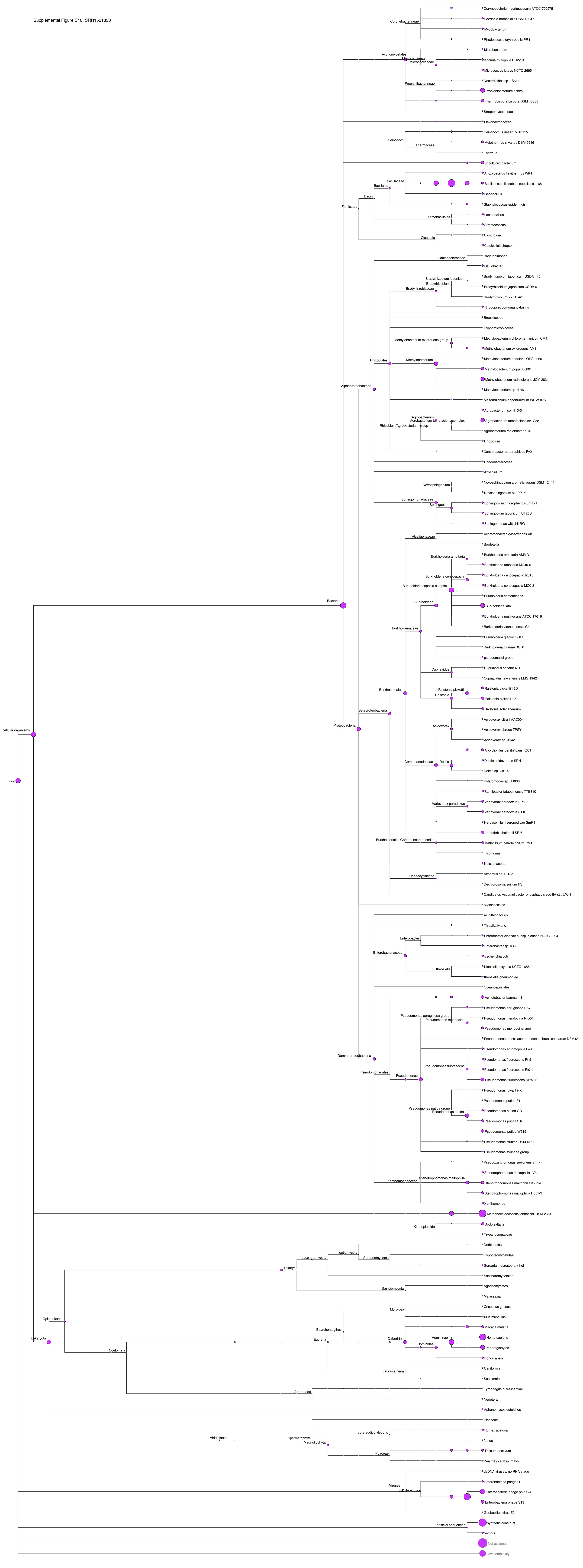

Supplemental Figure S11 SRR1021354

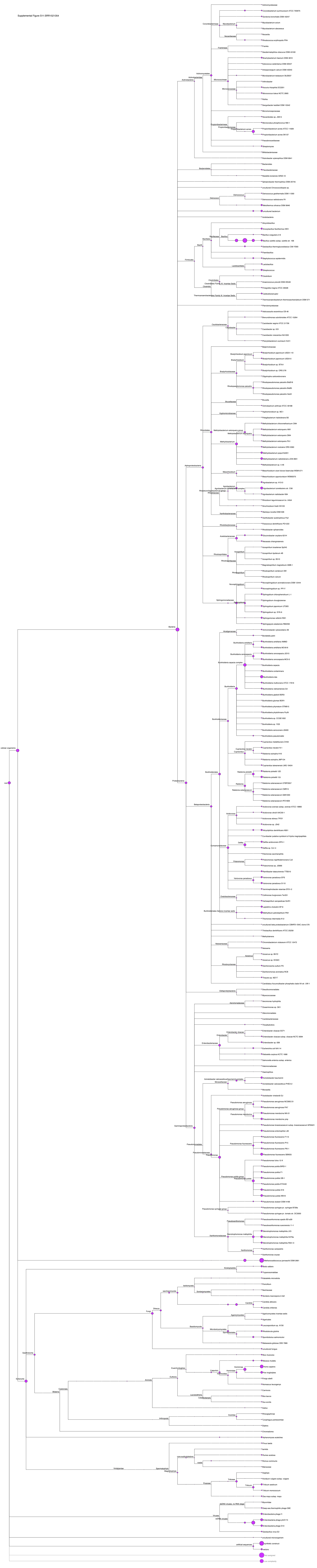

Supplemental Figure S12: SRR1521355

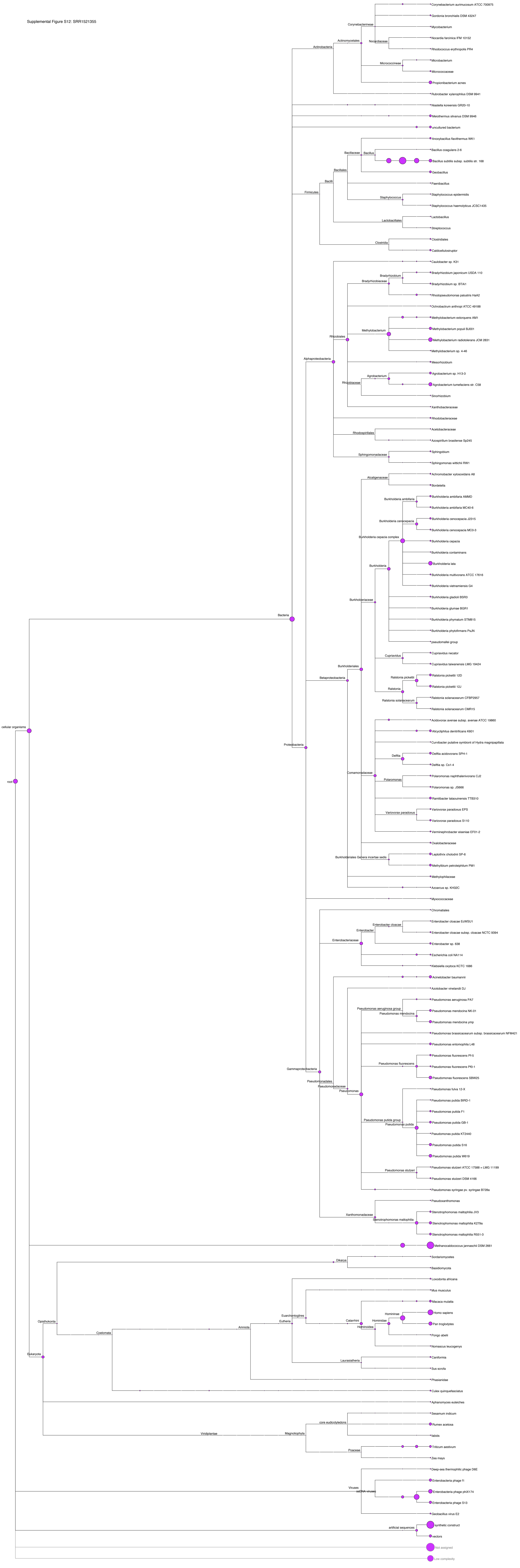

Supplemental Figure S13: SRR1521356

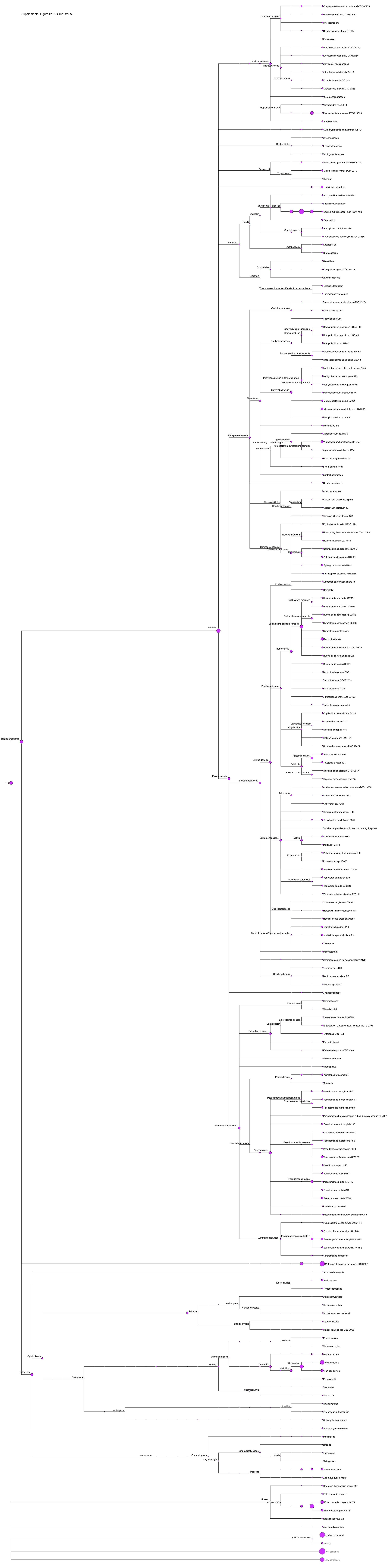

Supplemental Figure S14: SRR1521357

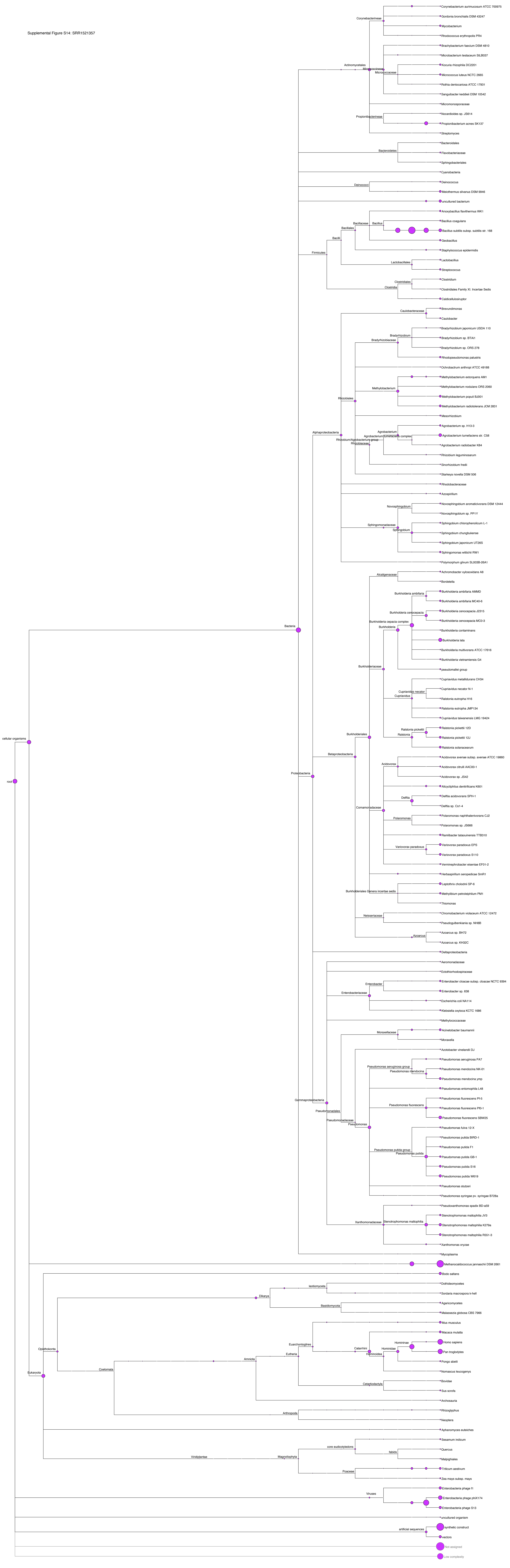

Supplemental Figure S15: SRR1521358

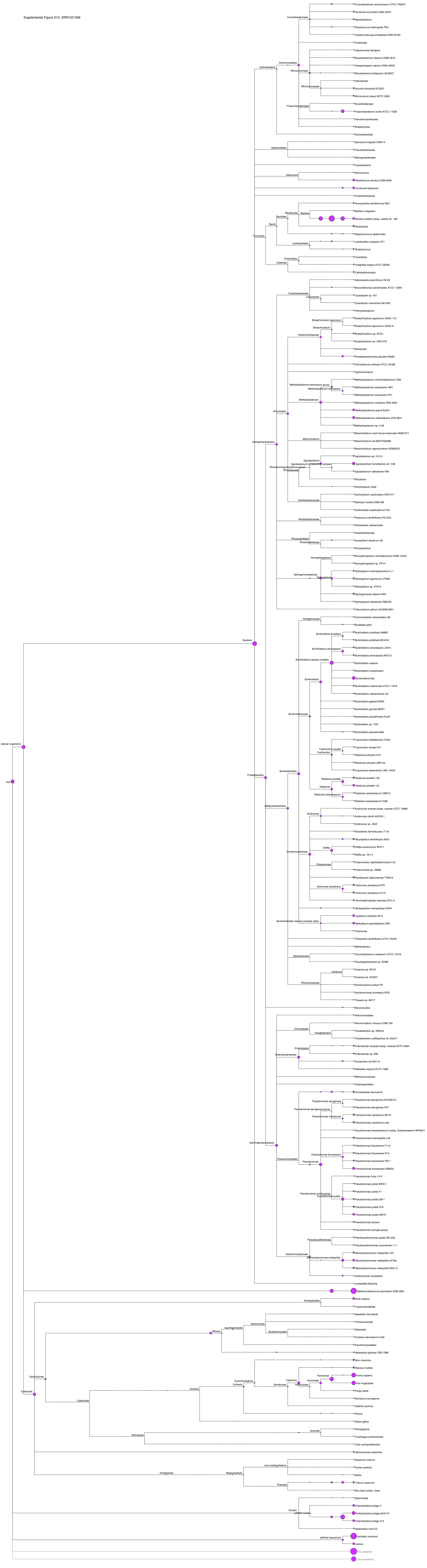

Supplemental Figure S16: SRR1021308

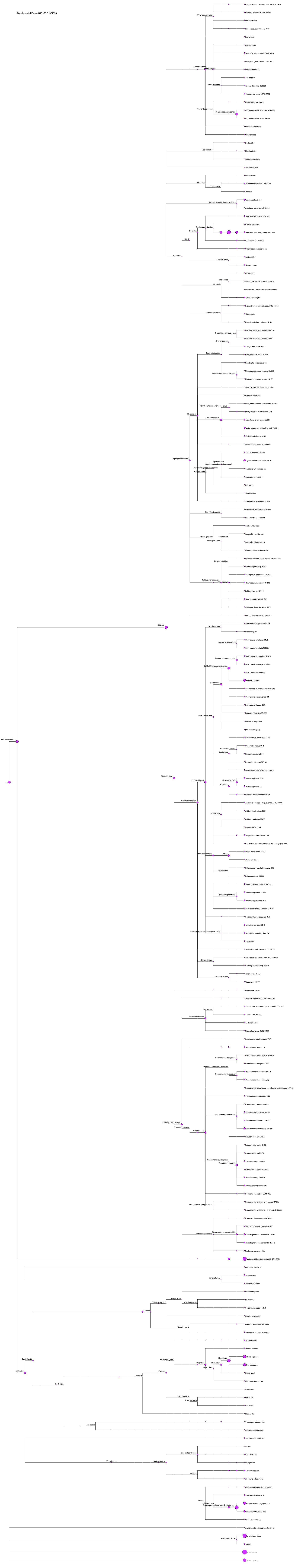

Supplemental Figure S17: SRR1521360

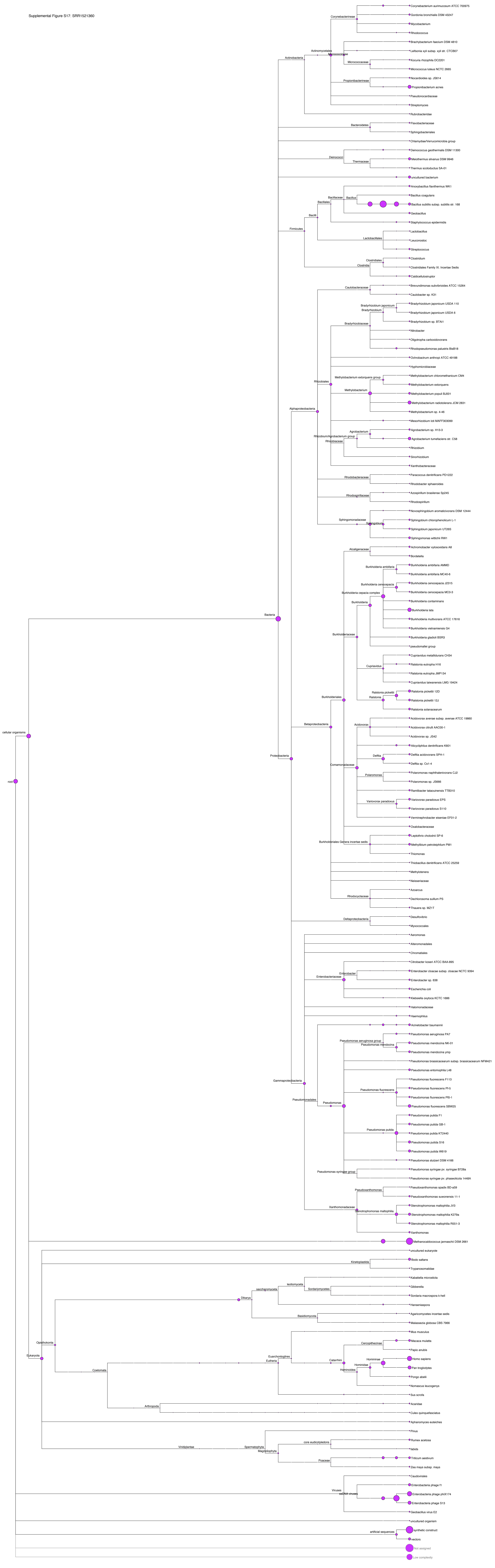

Supplemental Figure S18: SRR1521361

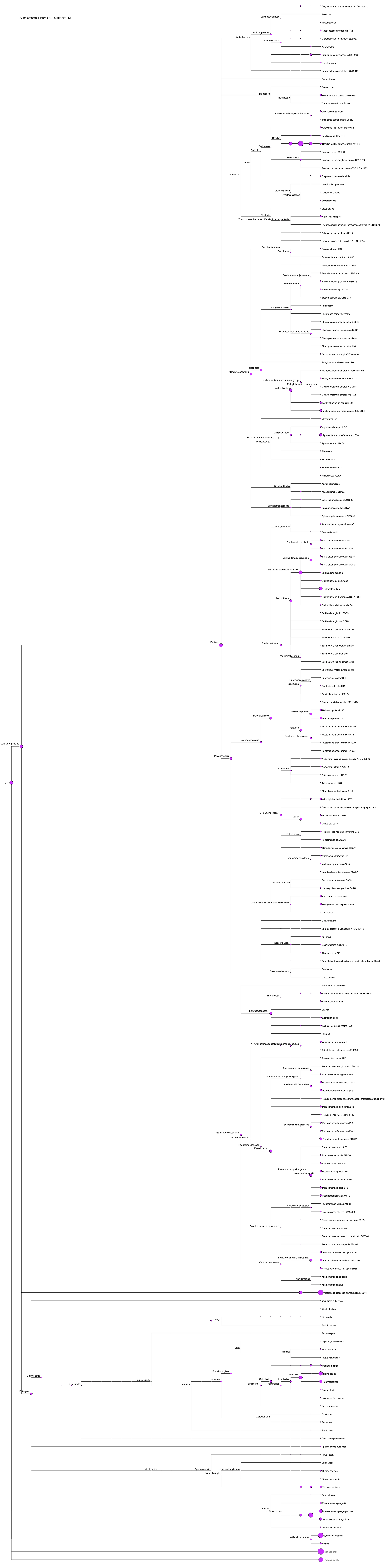

Supplemental Figure S19: SRR1521362

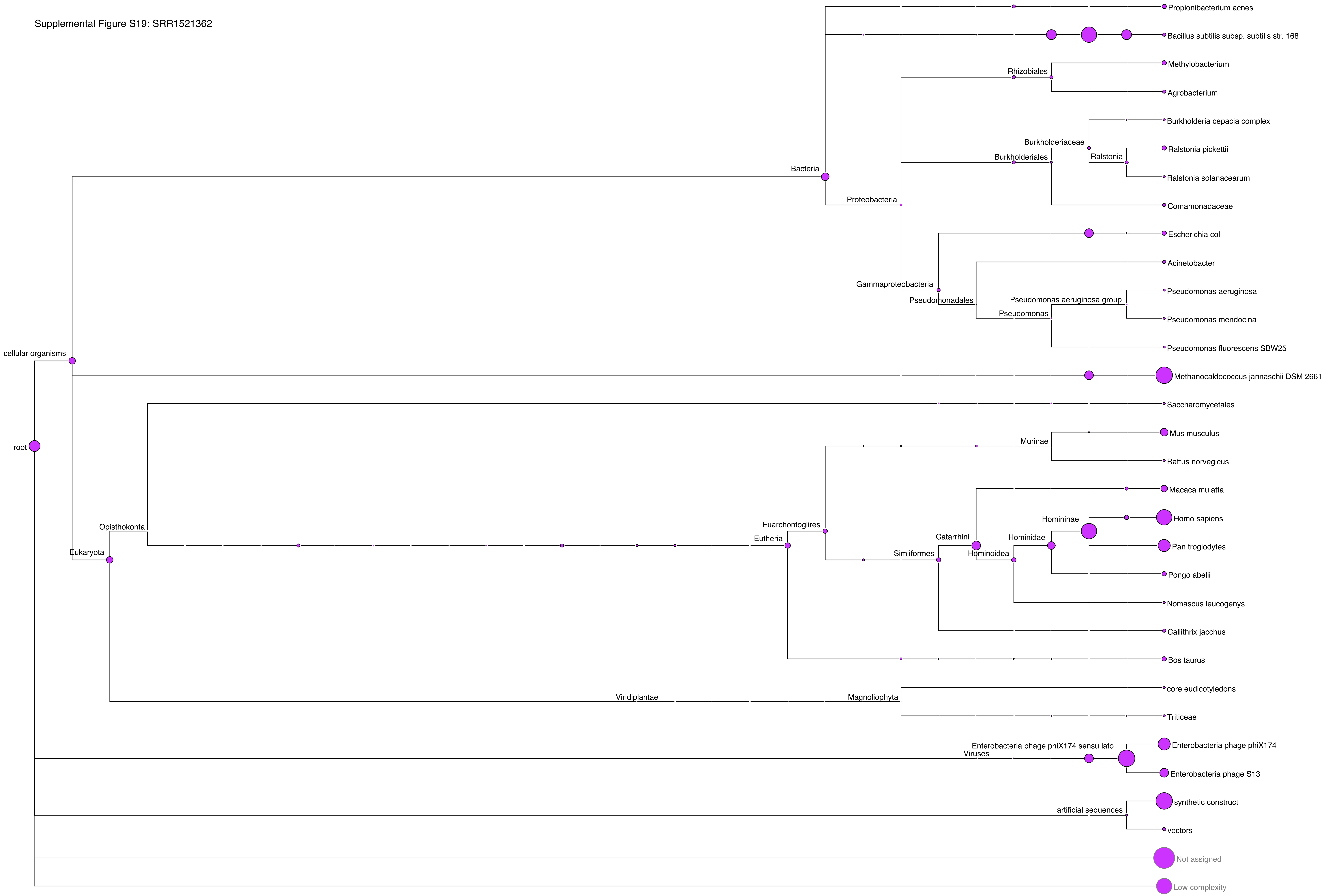

Supplemental Figure S20: SRR1521363

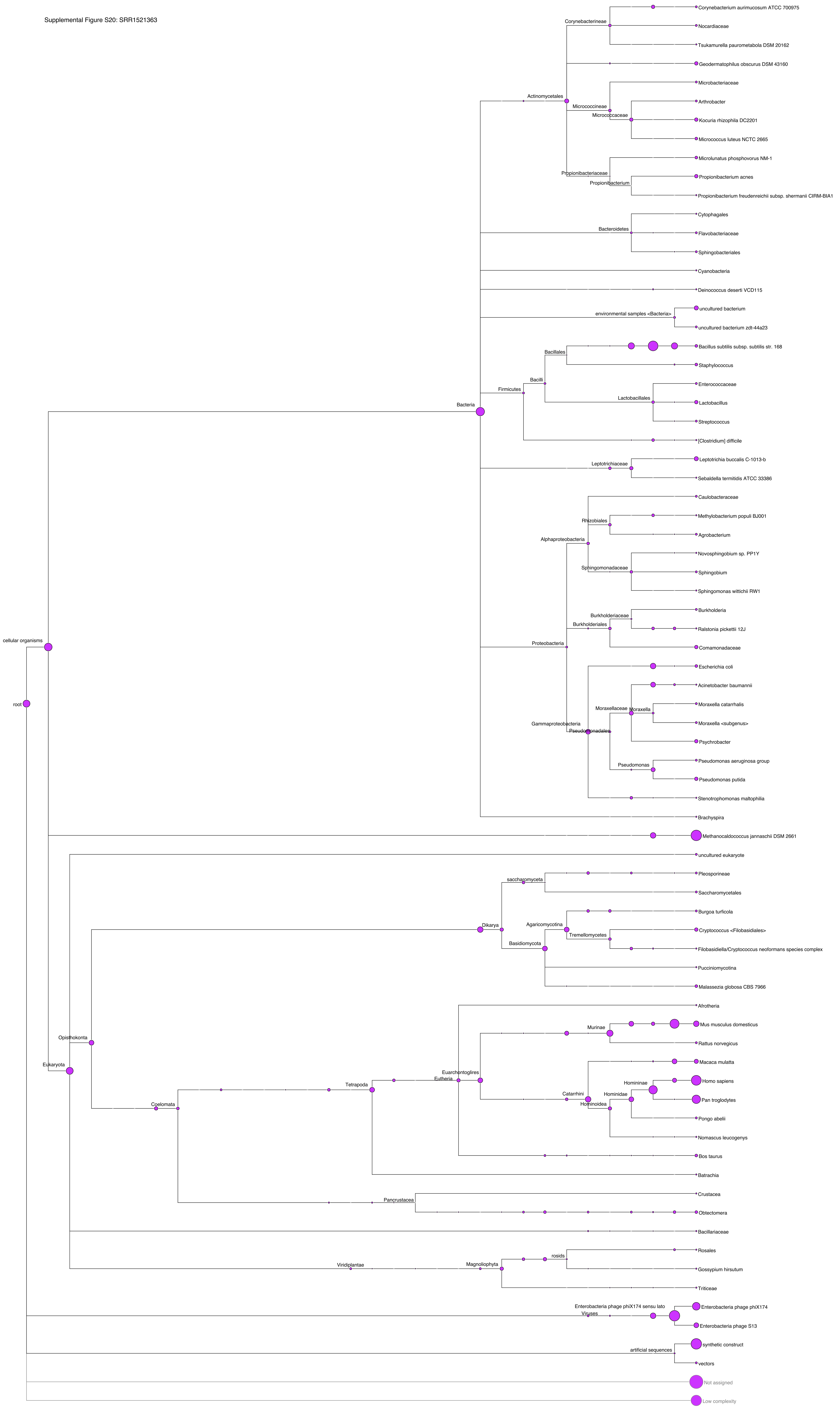

Supplemental Figure S21: SRR1521364

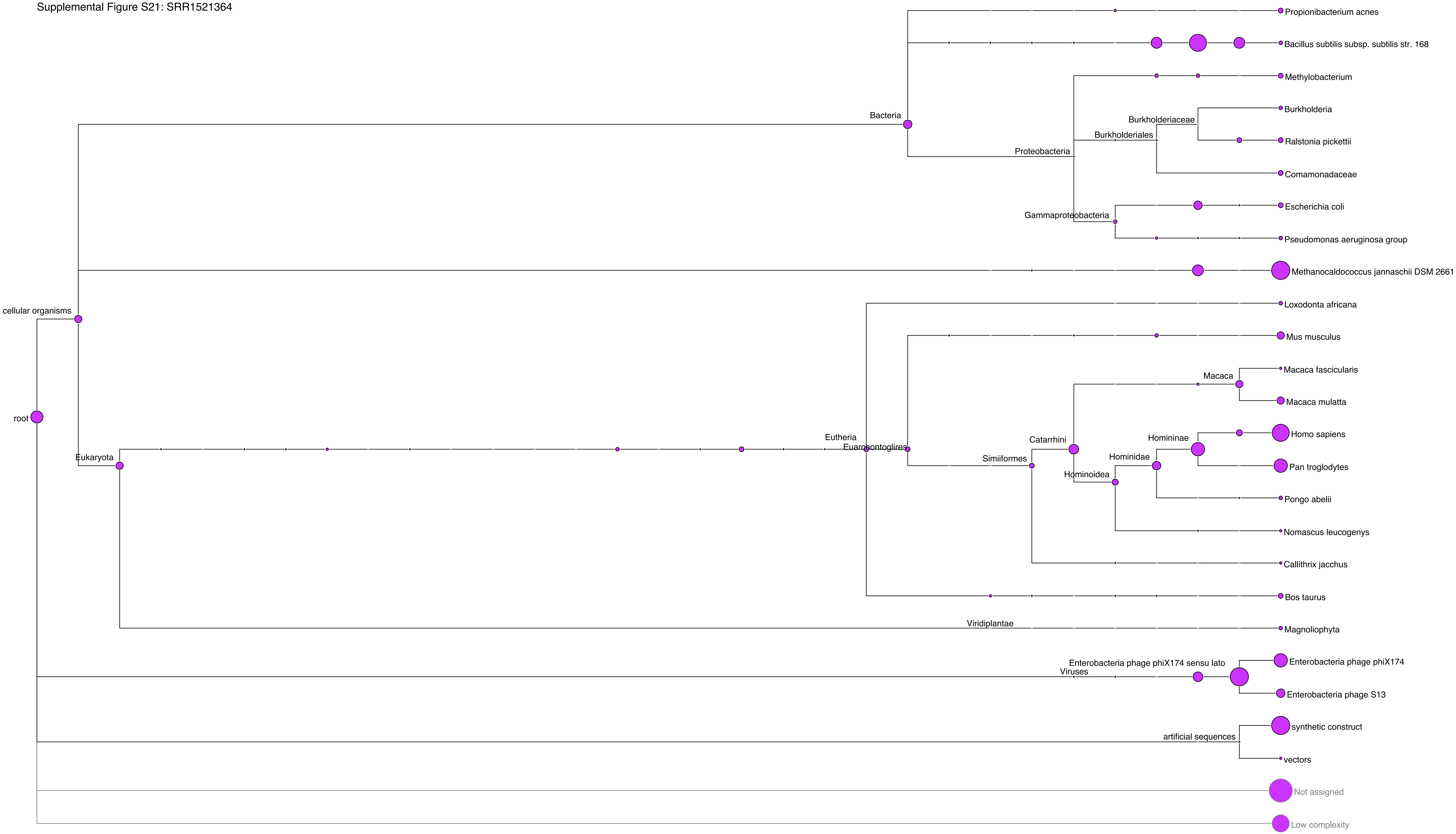

Supplemental Figure S22: SRR1521365

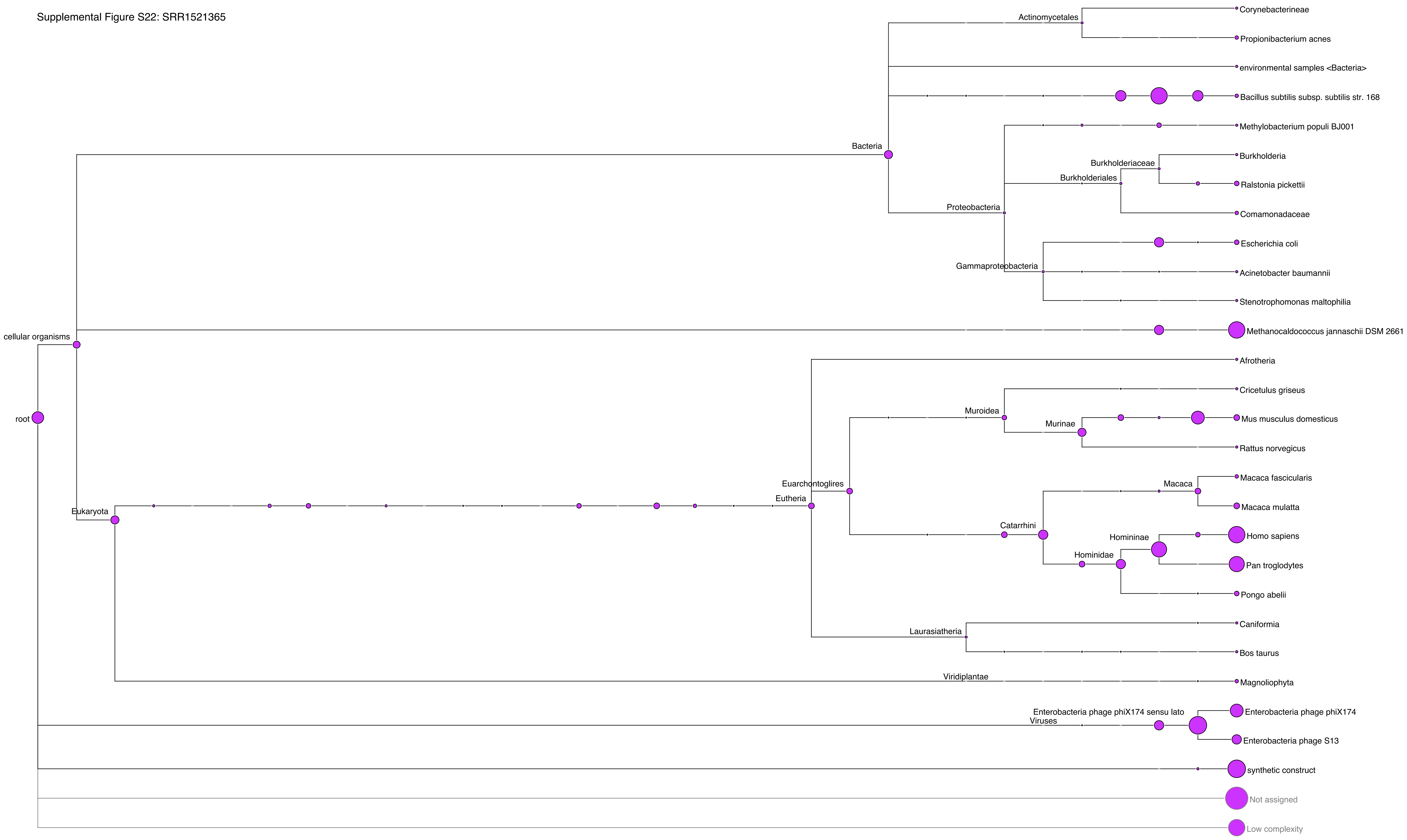

Supplemental Figure S23: SRR1521366

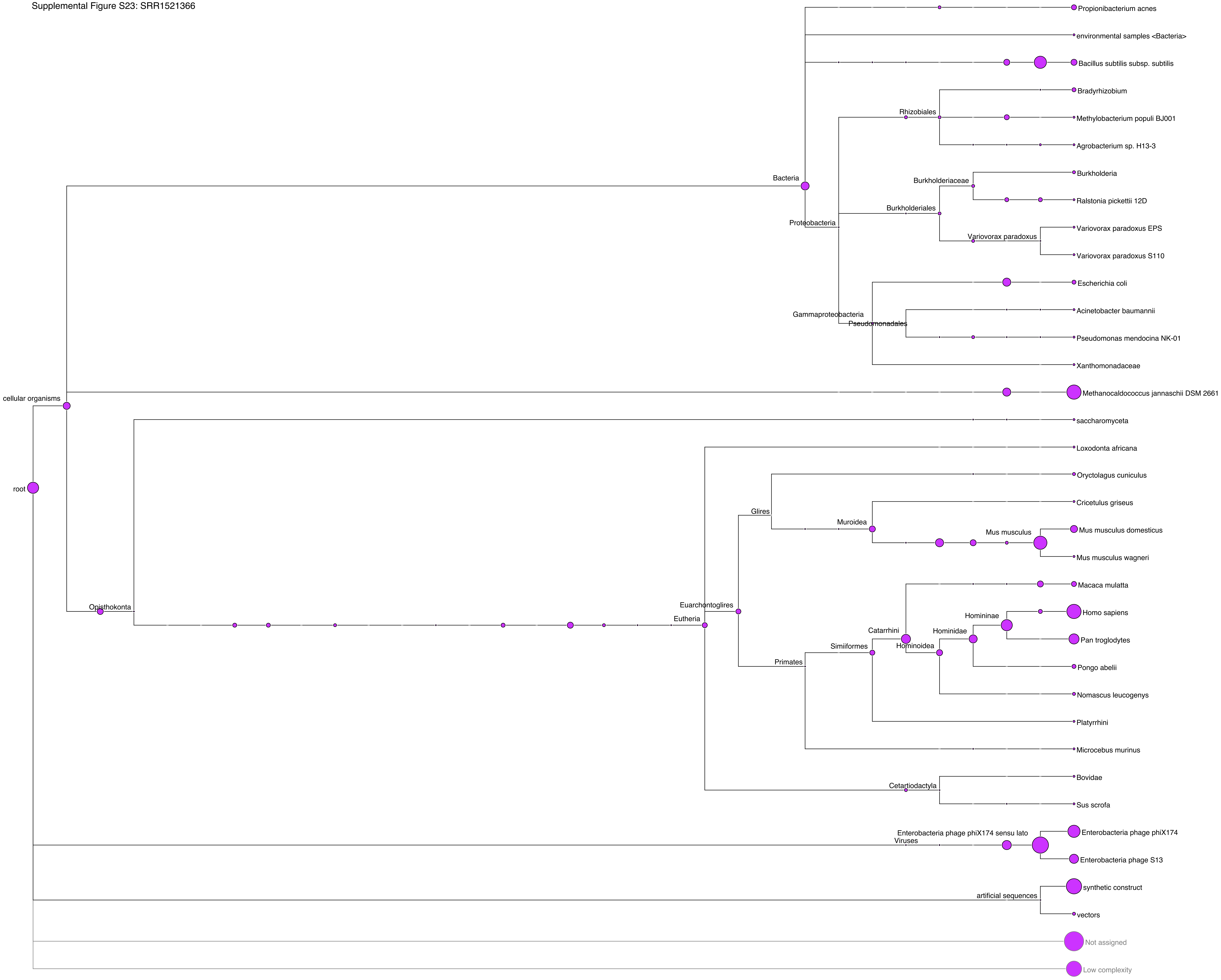

Supplemental Figure S24: SRR1521367

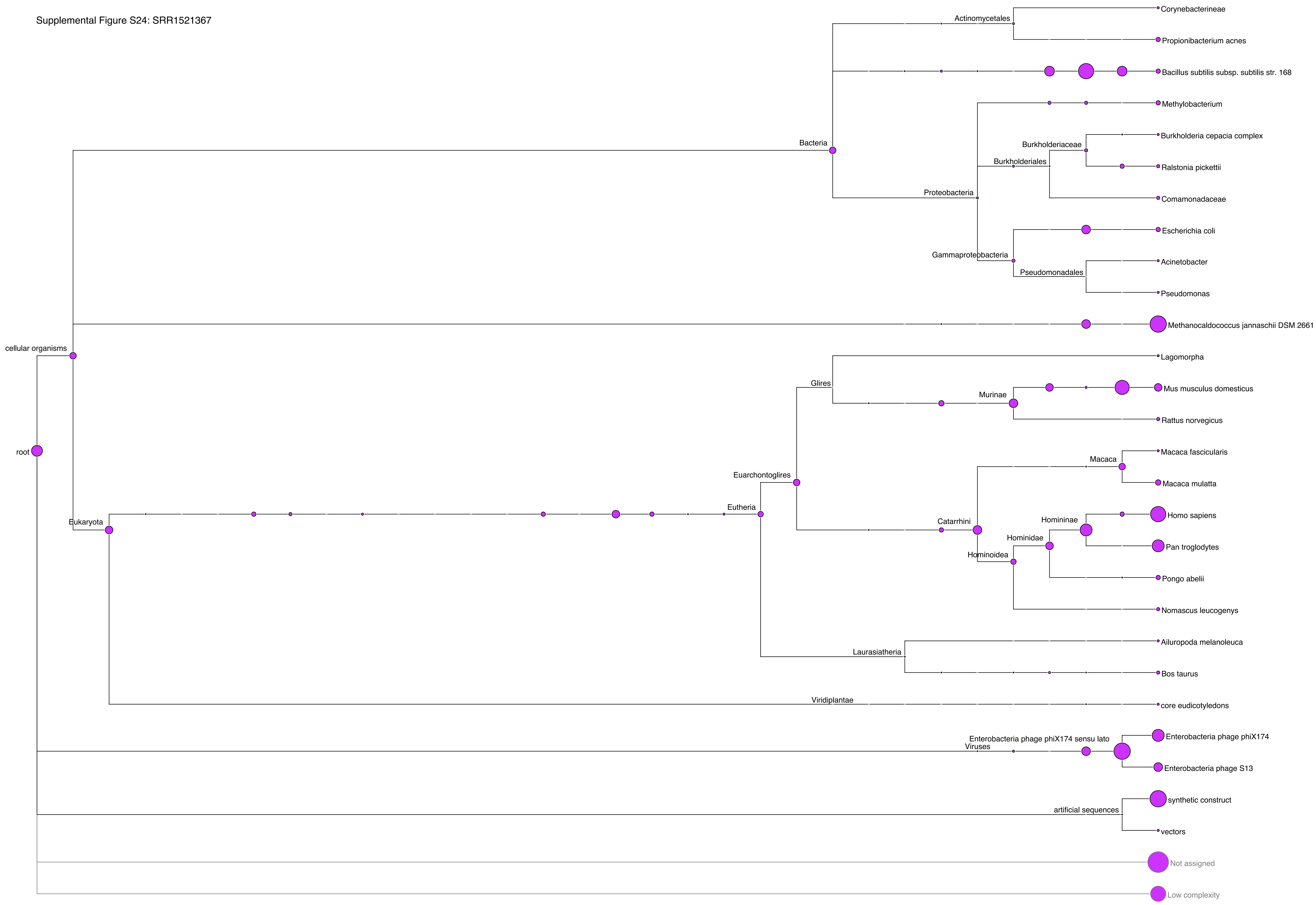

Supplemental Figure S25: SRR1521368

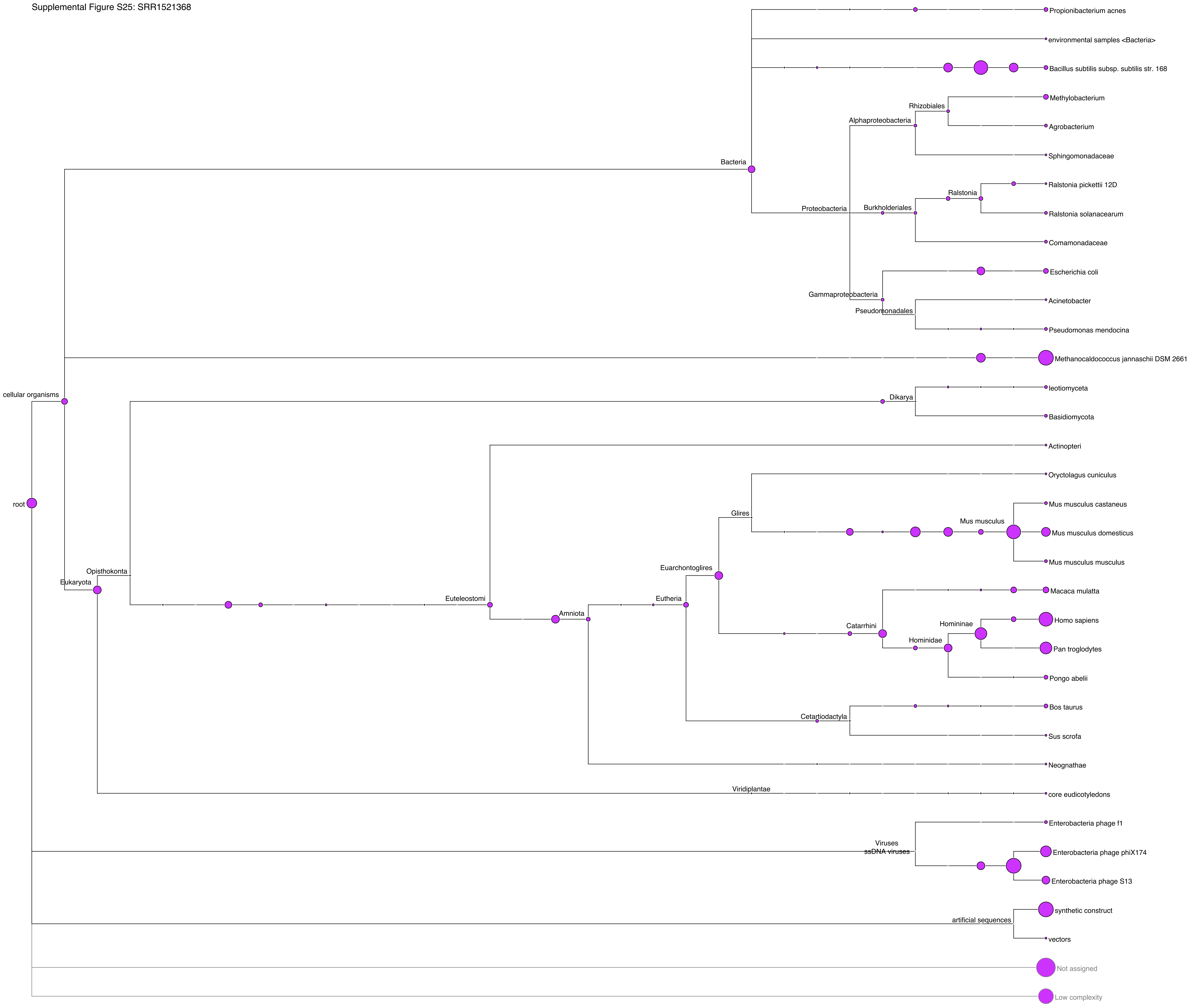

Supplemental Figure S26: SRR1521369

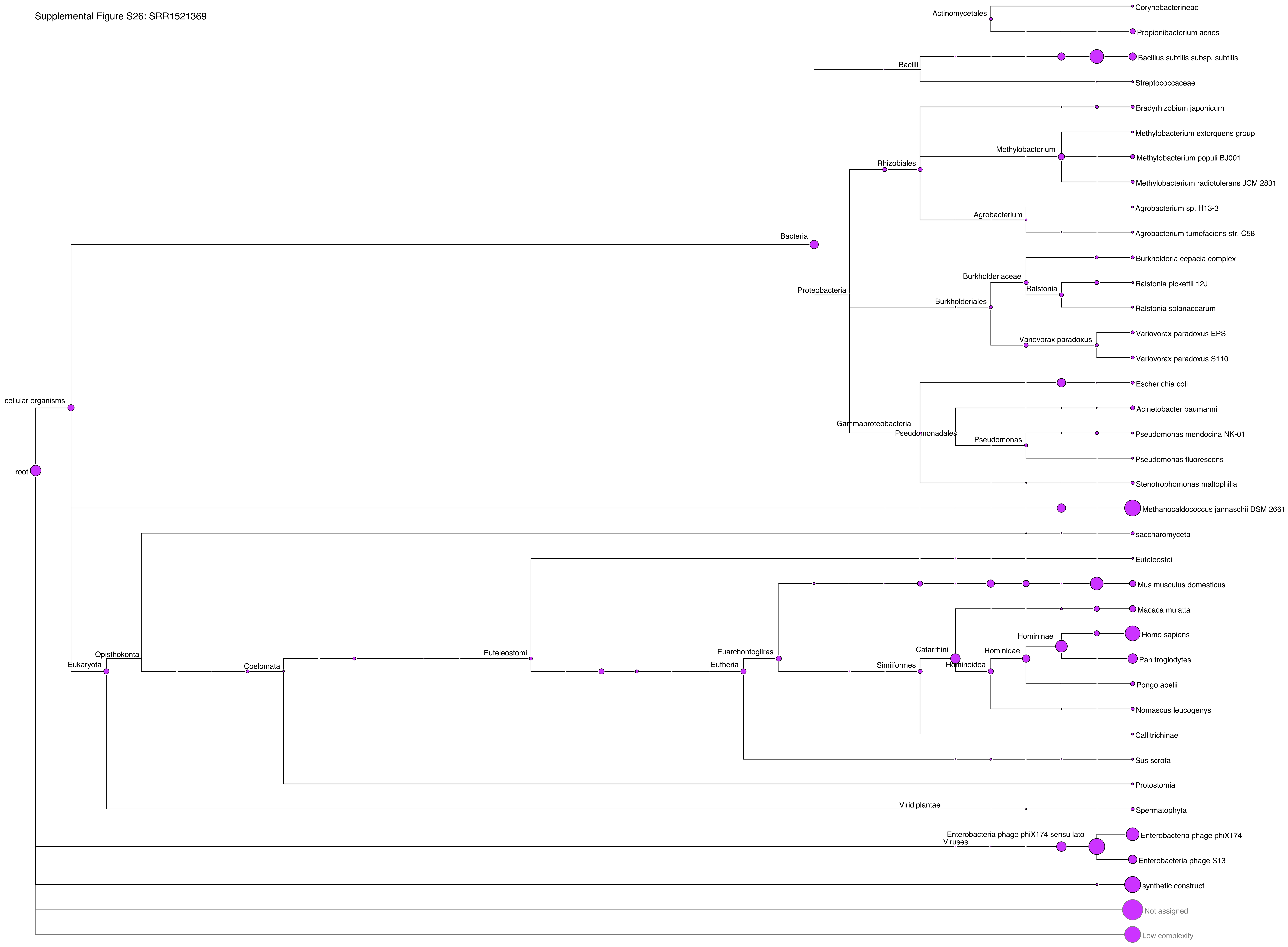

Supplemental Figure S27: SRR1521370

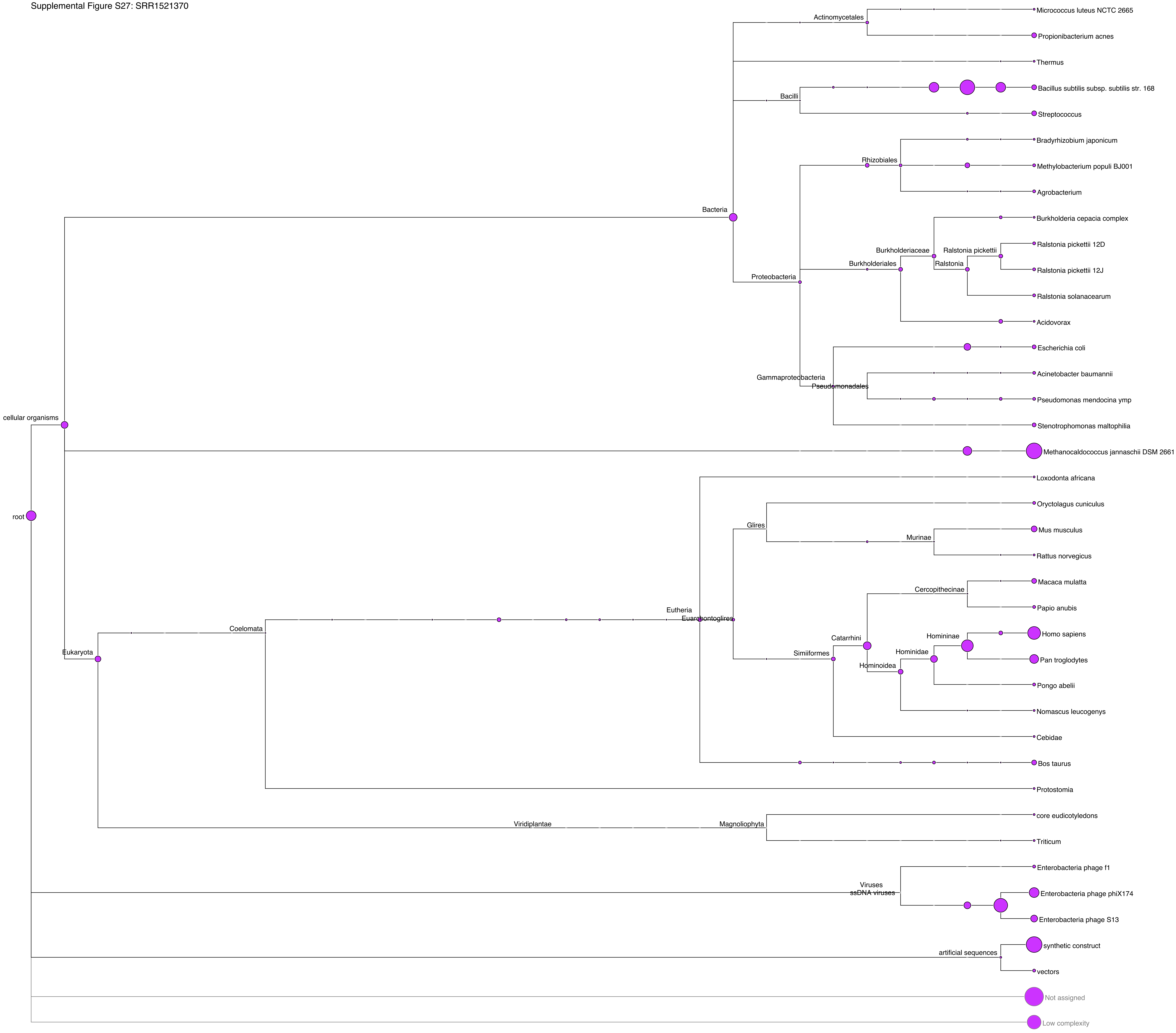

Supplemental Figure S28: SRR1521371

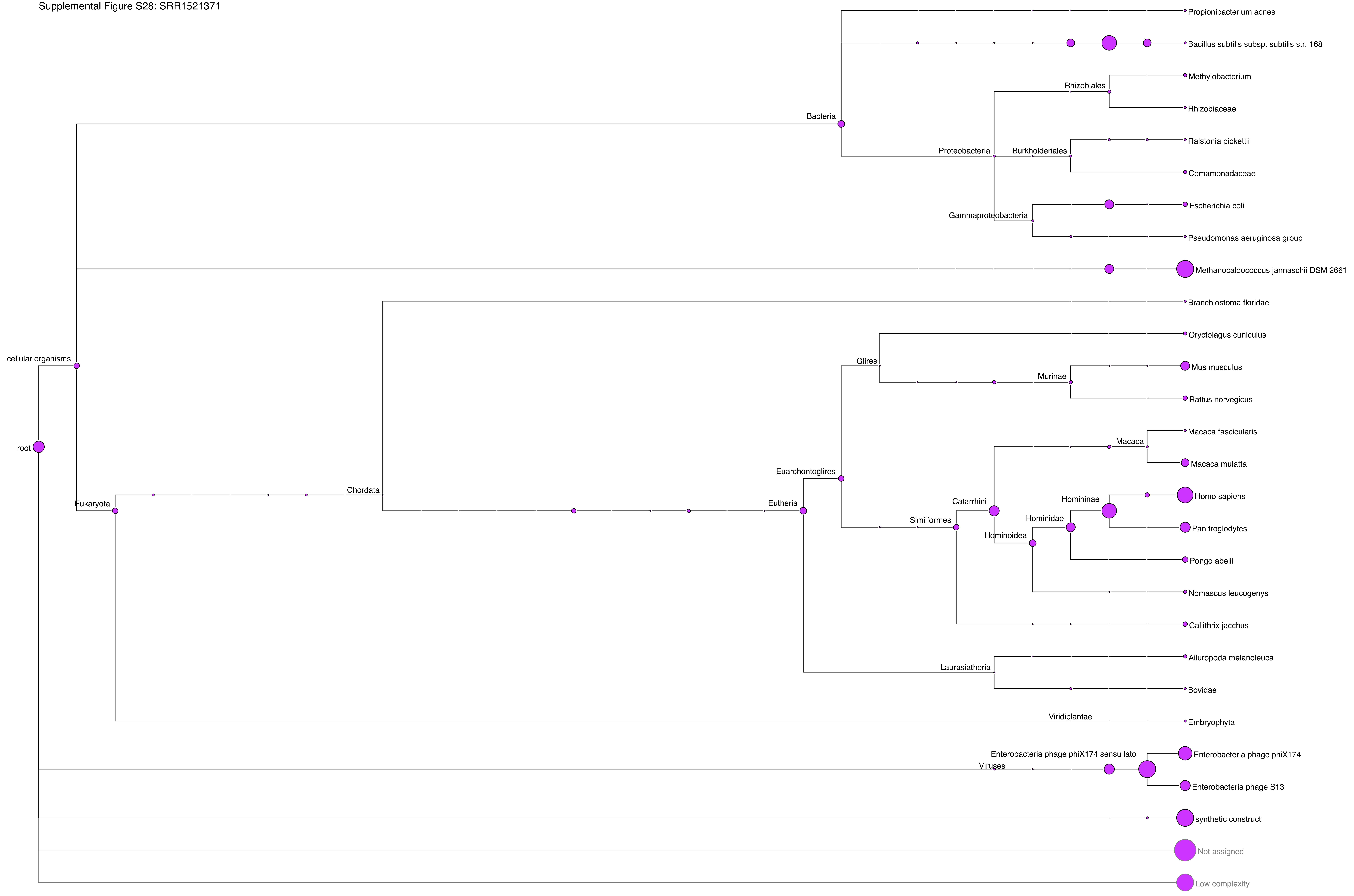

Supplemental Figure S29: SRR1521372

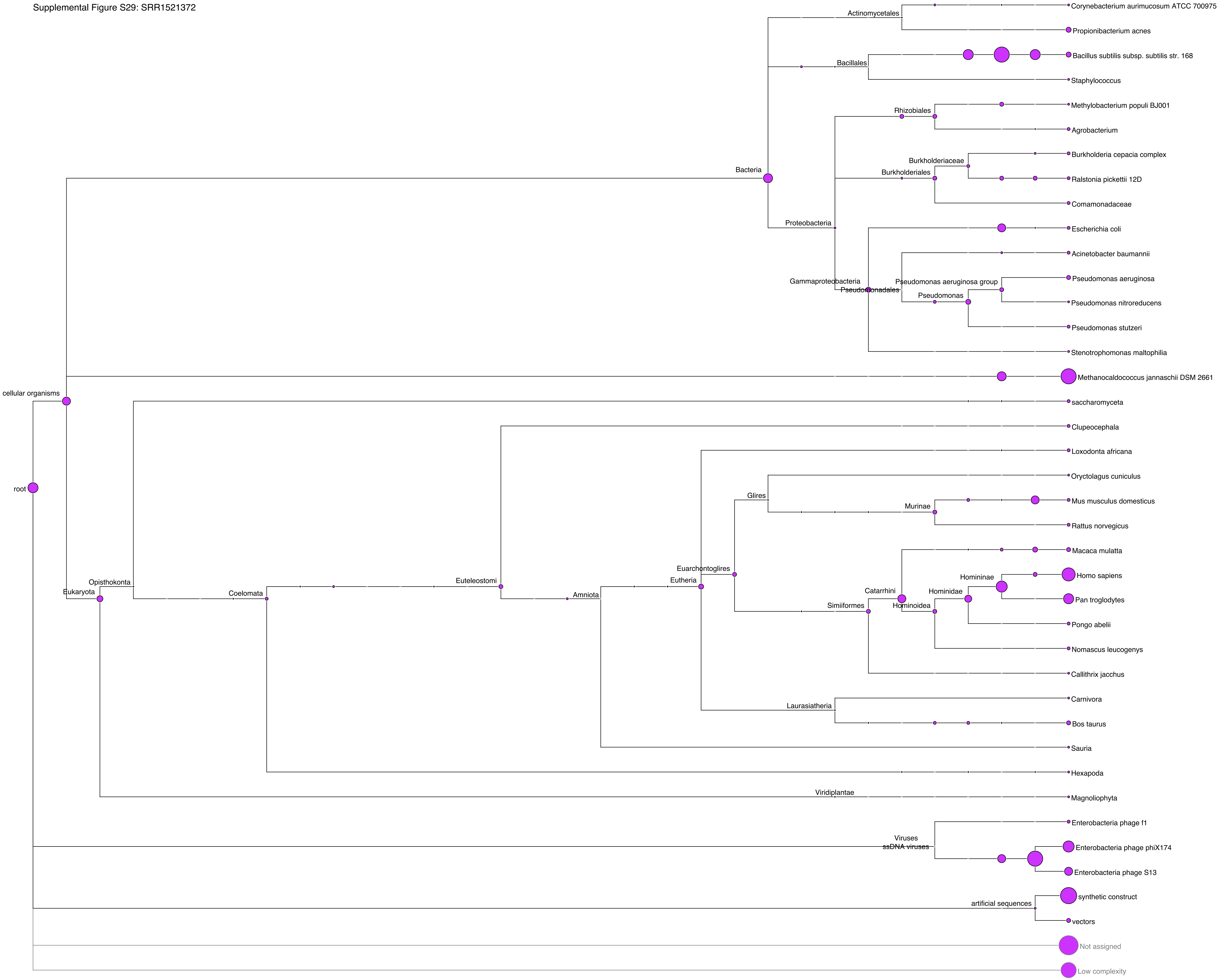

Supplemental Figure S30: SRR1521373

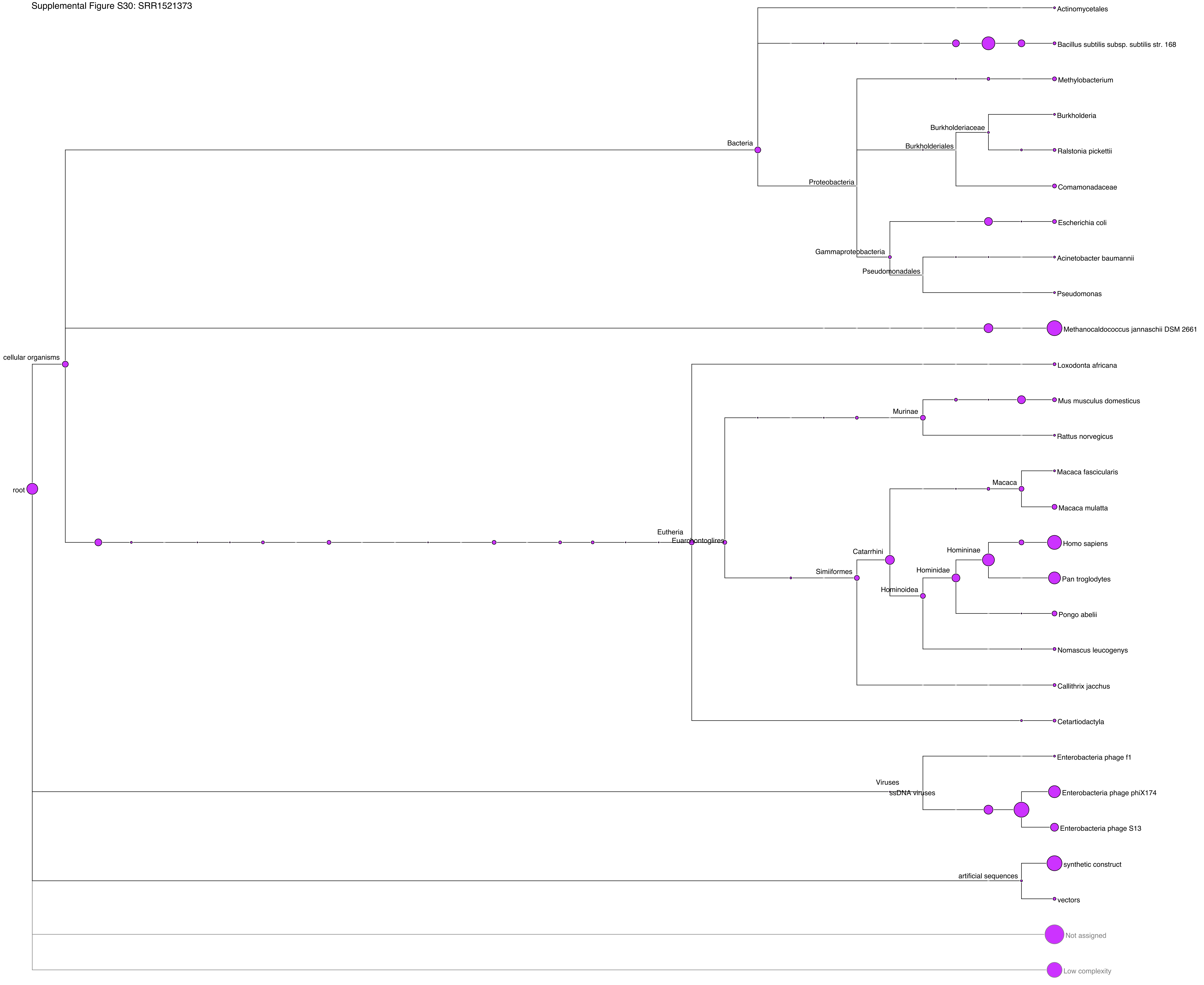

Supplemental Figure S31: SRR1521374

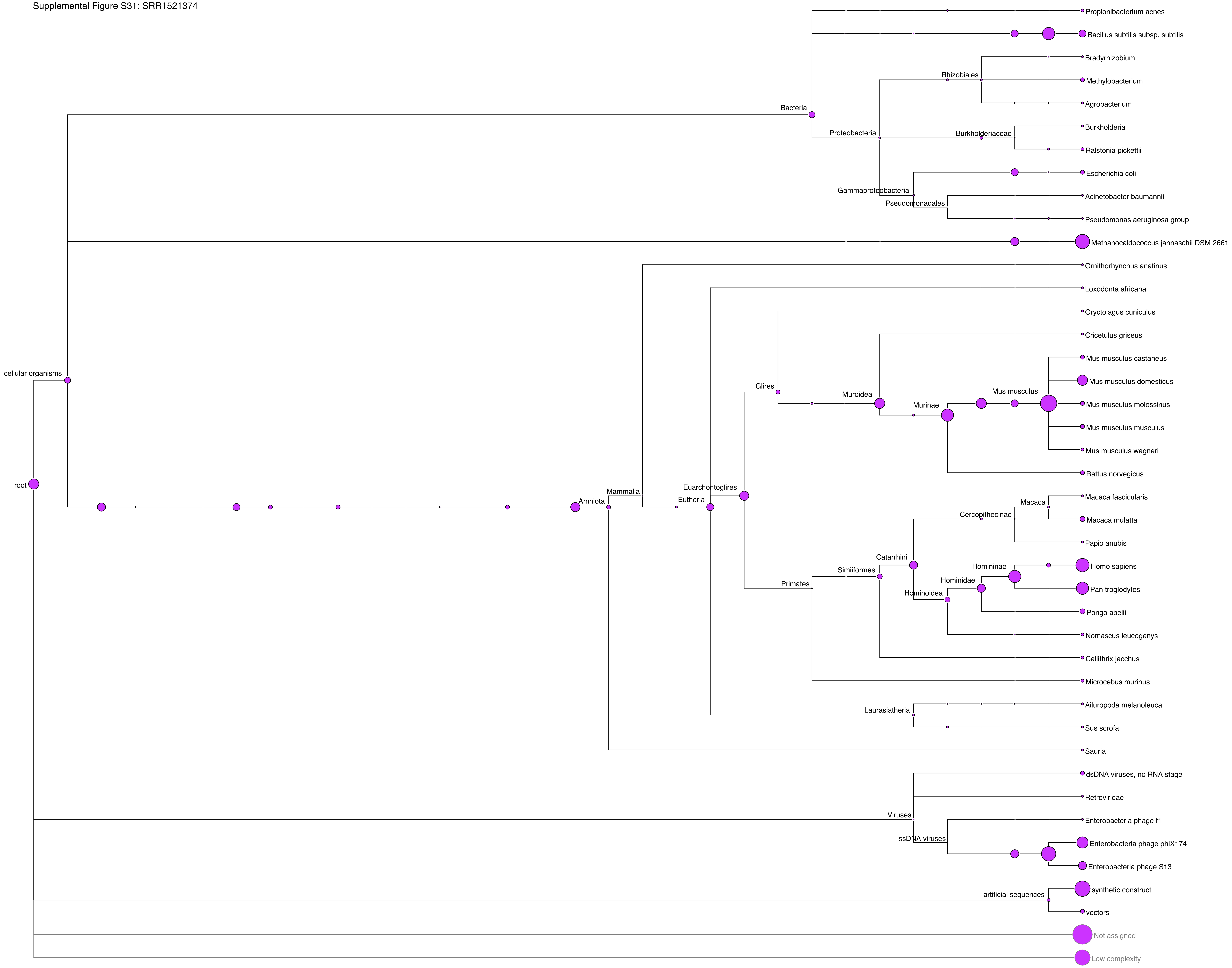

Supplemental Figure S32: SRR1521375

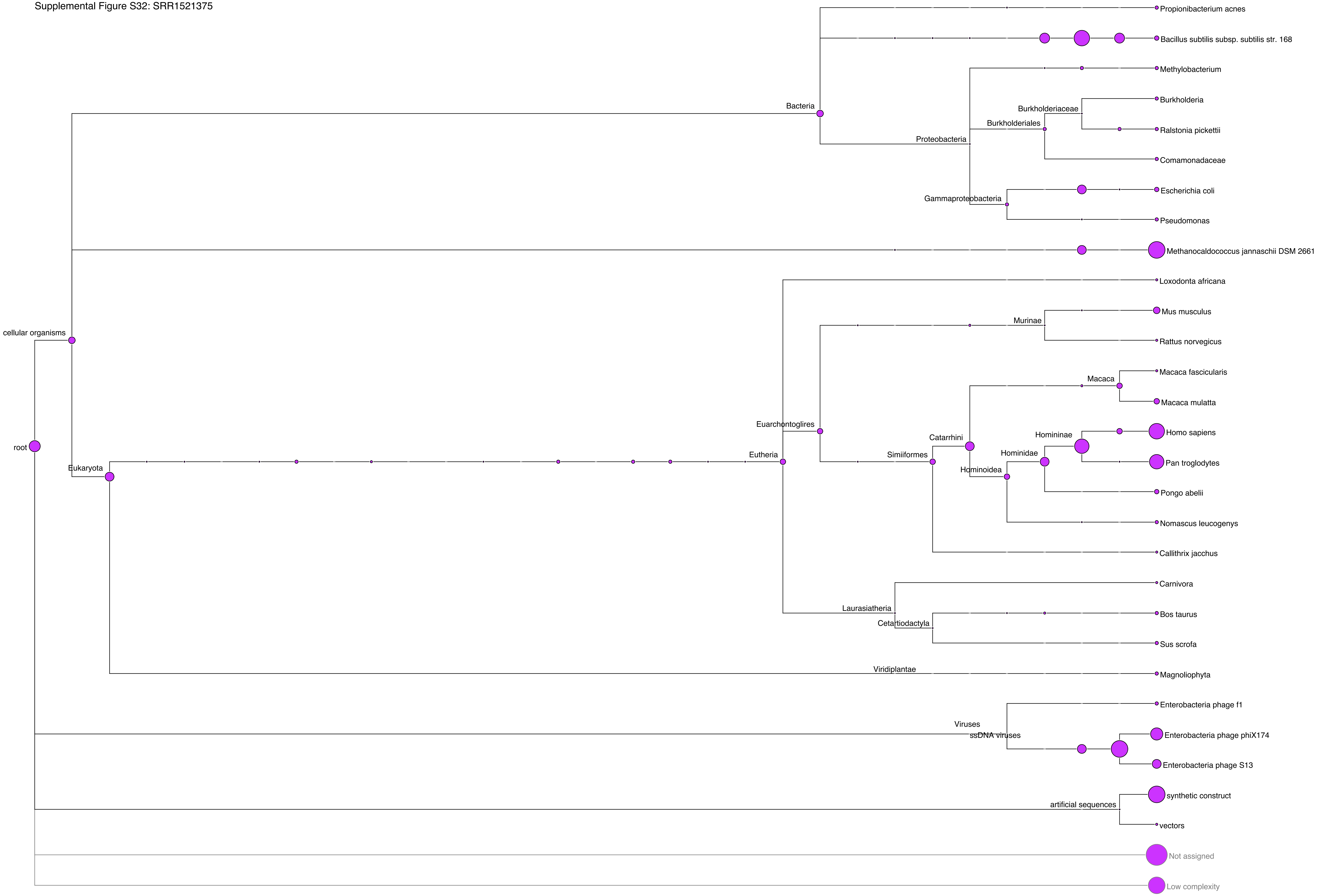

Supplemental Figure S33: SRR1521376

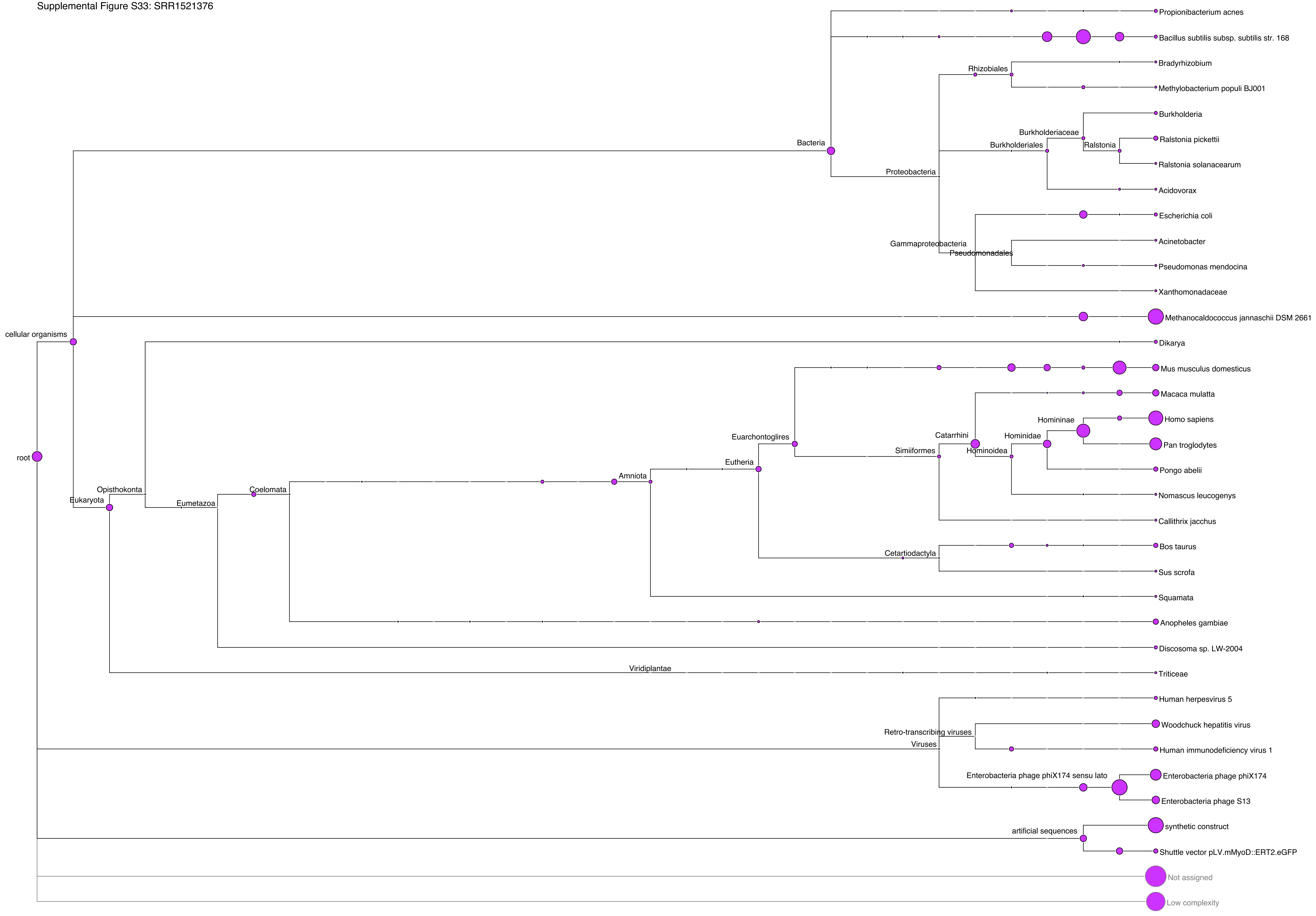

Supplemental Figure S34: SRR1521377

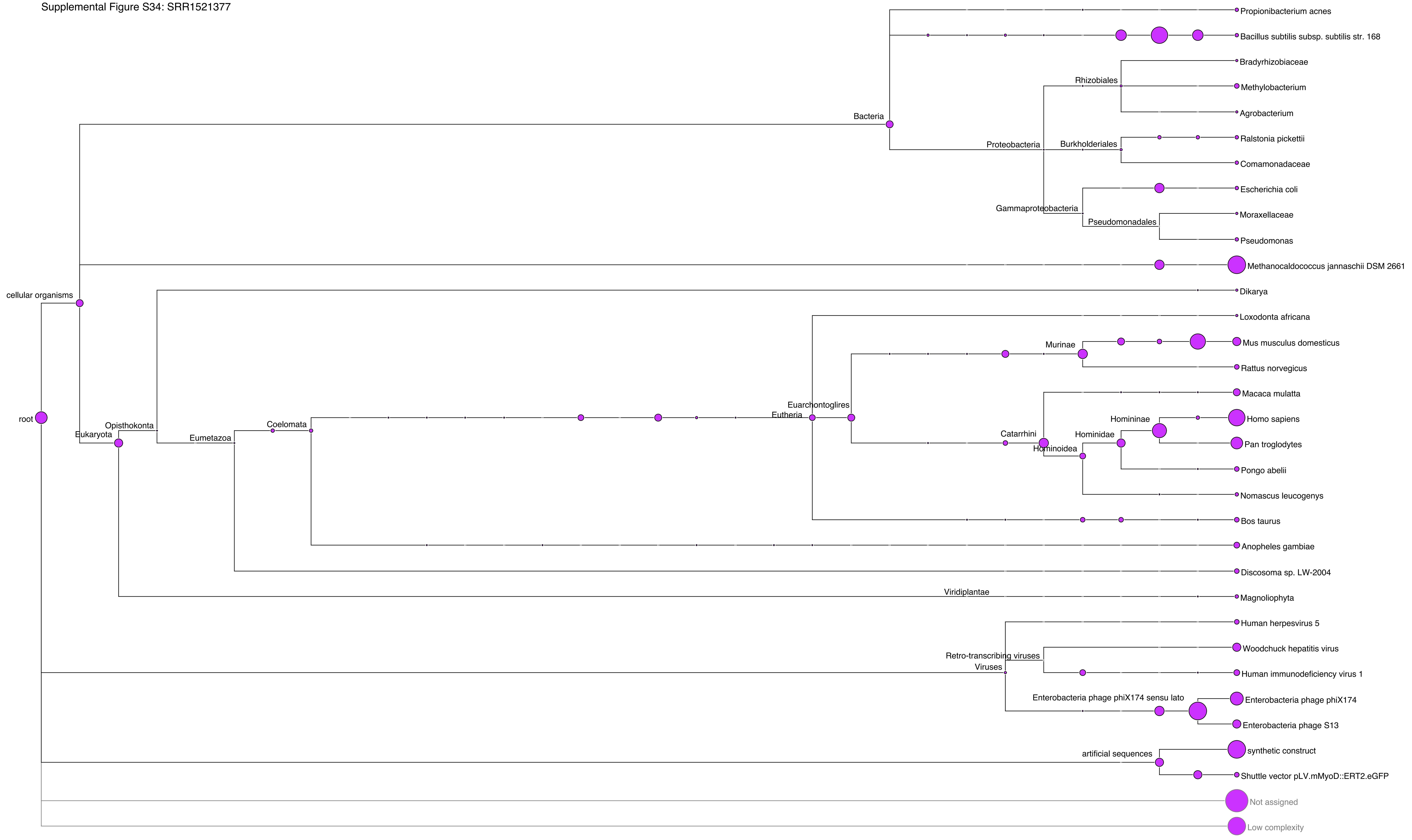

Supplemental Figure S35: SRR1521378

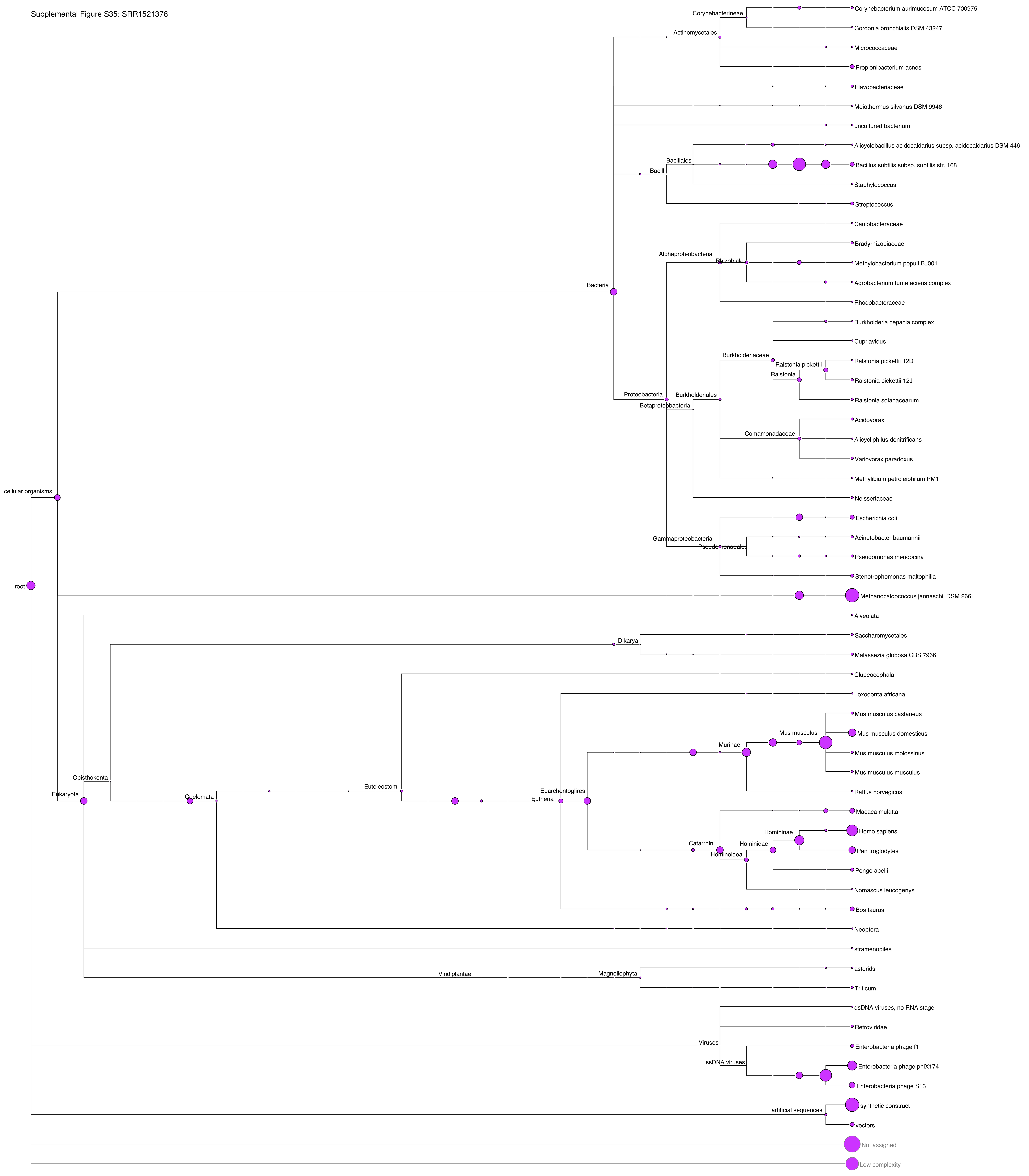

Supplemental Figure S36: SRR1521379

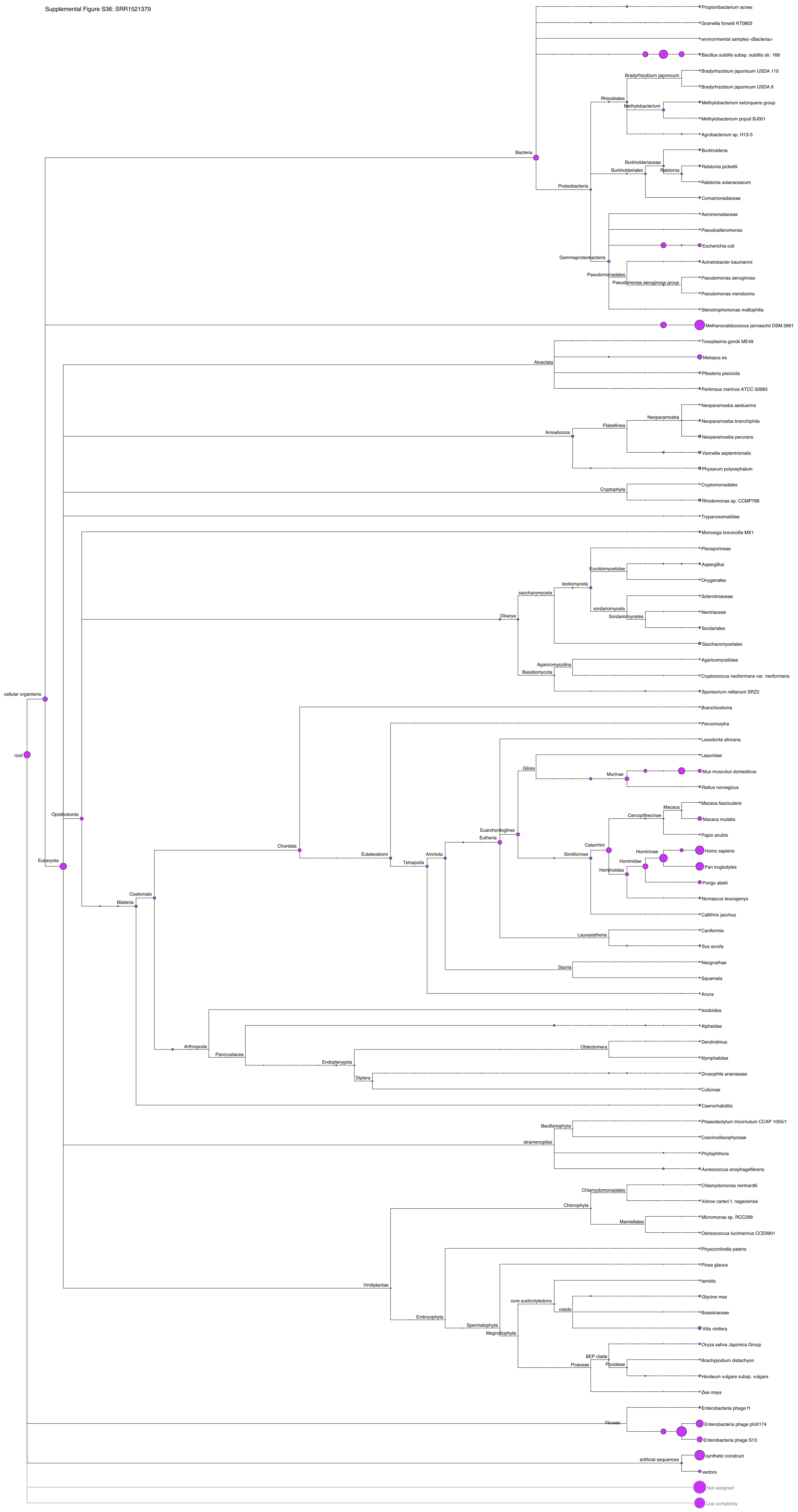

Supplemental Figure S37: SRR1521380

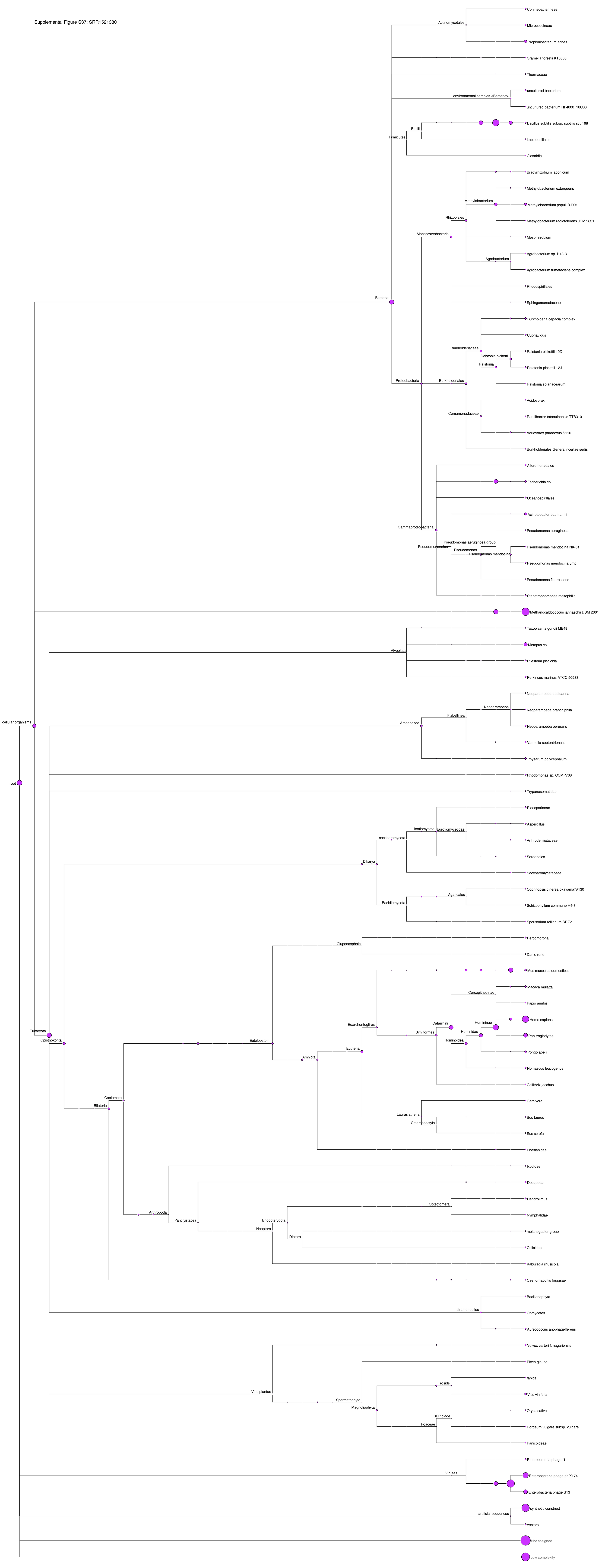

Supplemental Figure S38: SRR1521381

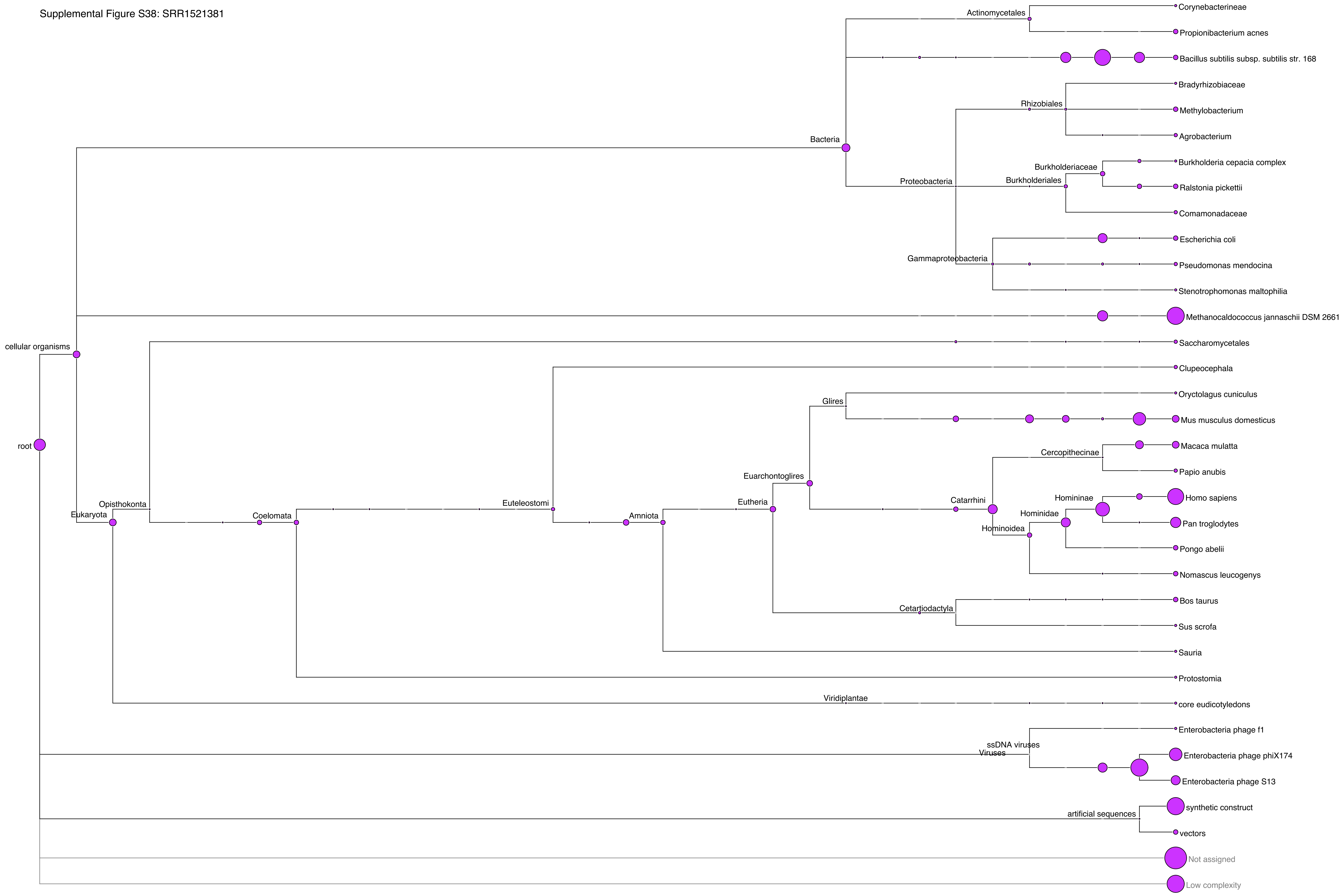

Supplemental Figure S39: SRR1521382

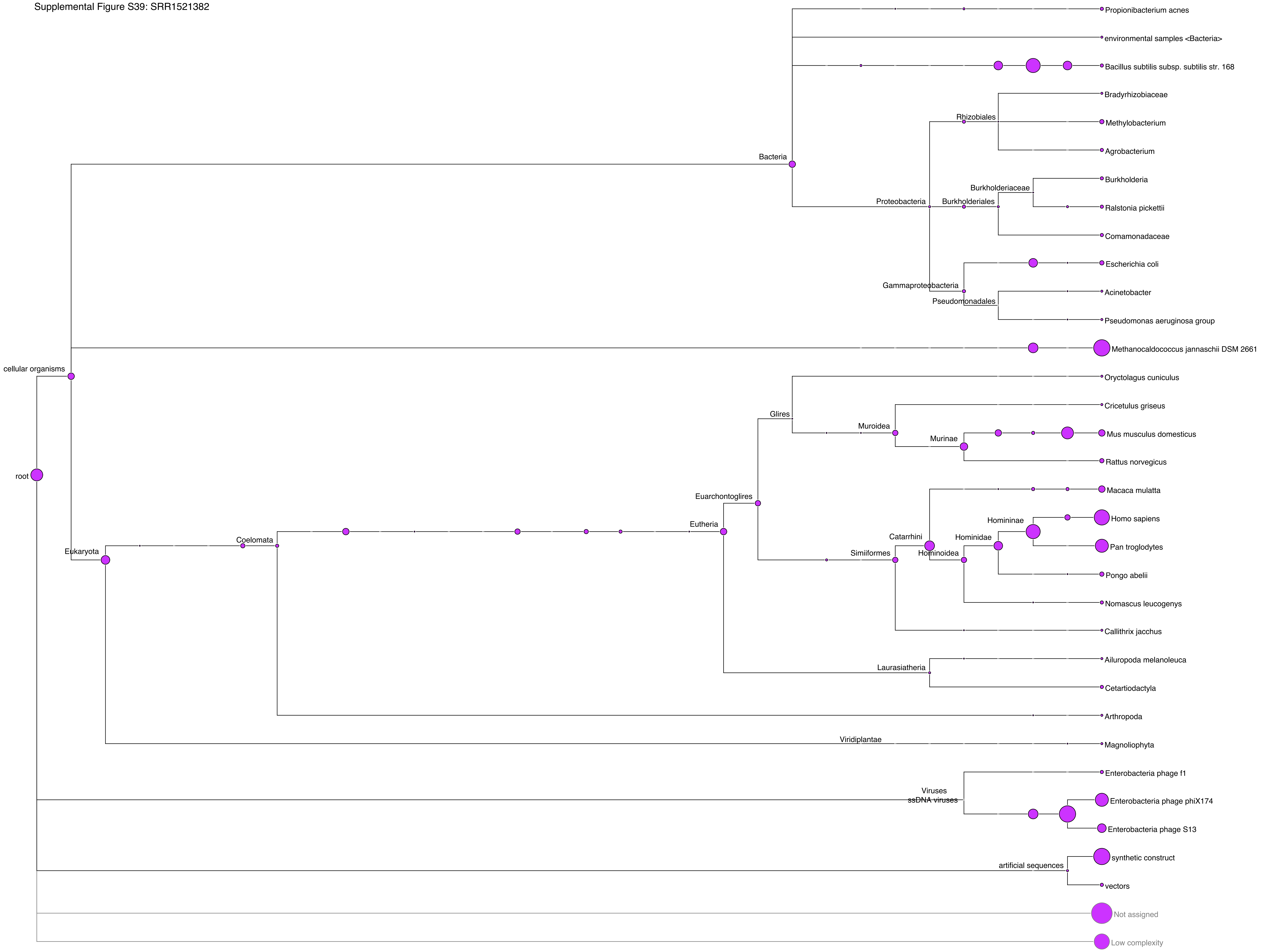

Supplemental Figure S40: SRR1521383

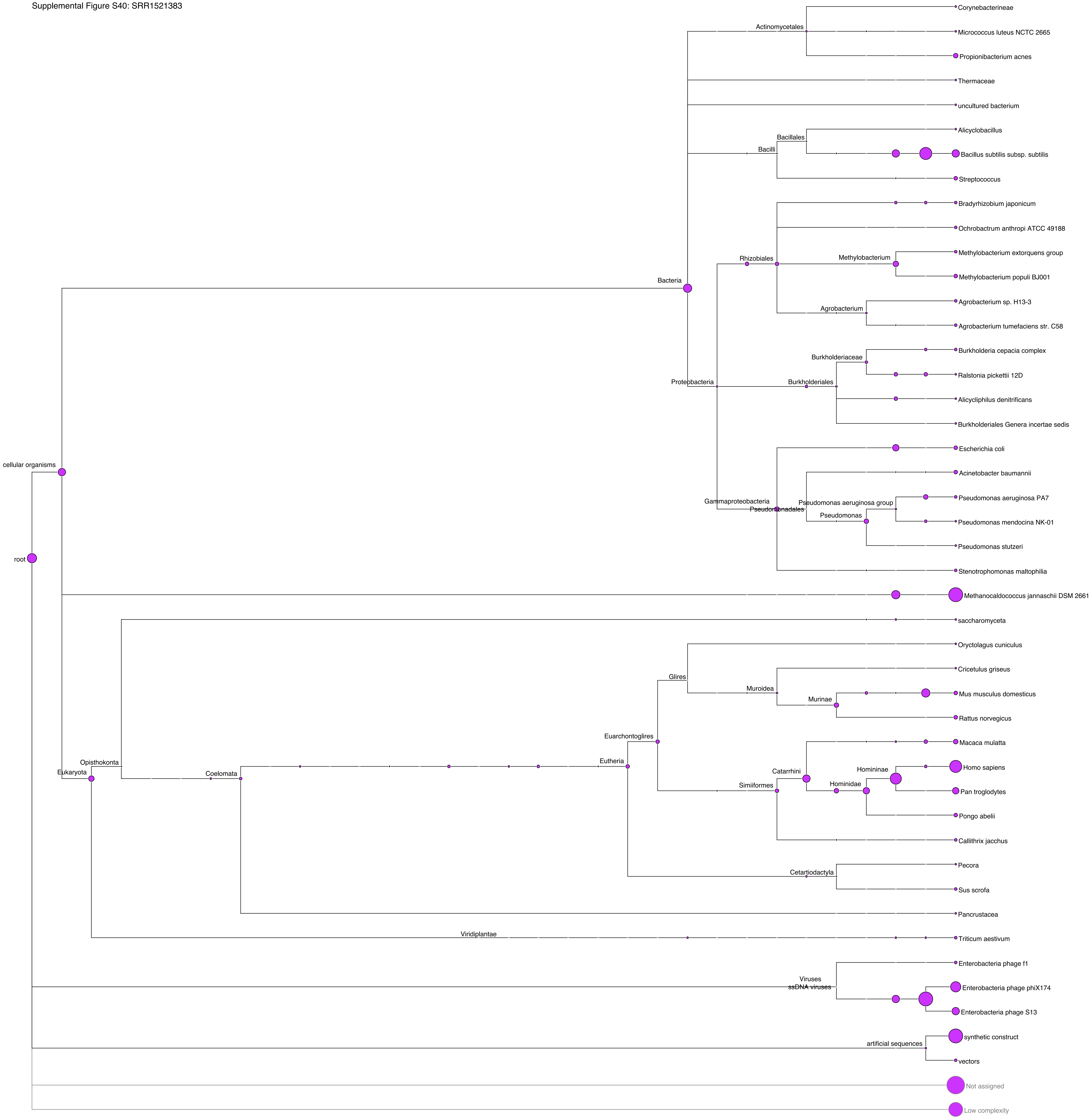

Supplemental Figure S41: SRR1521384

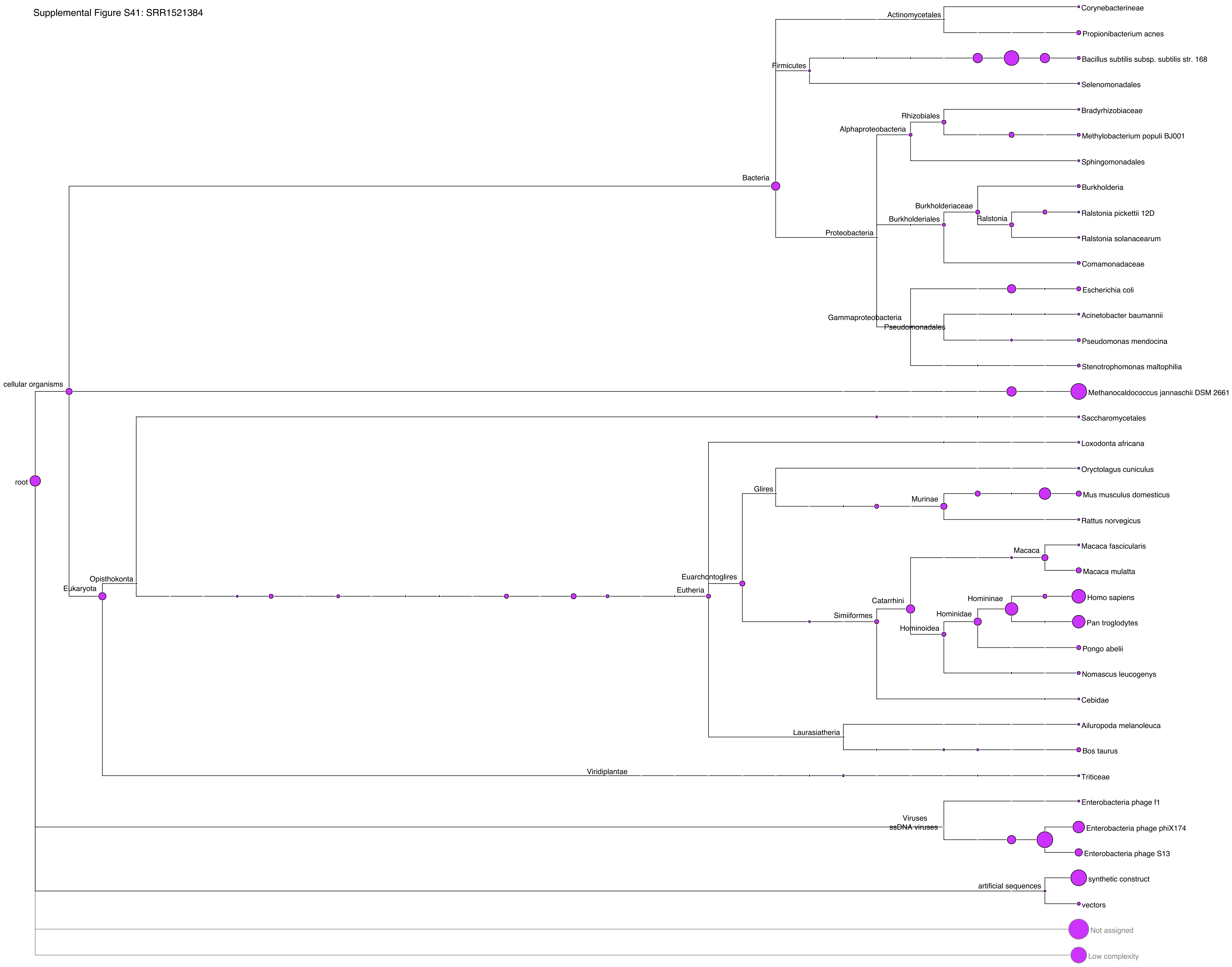

Supplemental Figure S42: SRR1521385

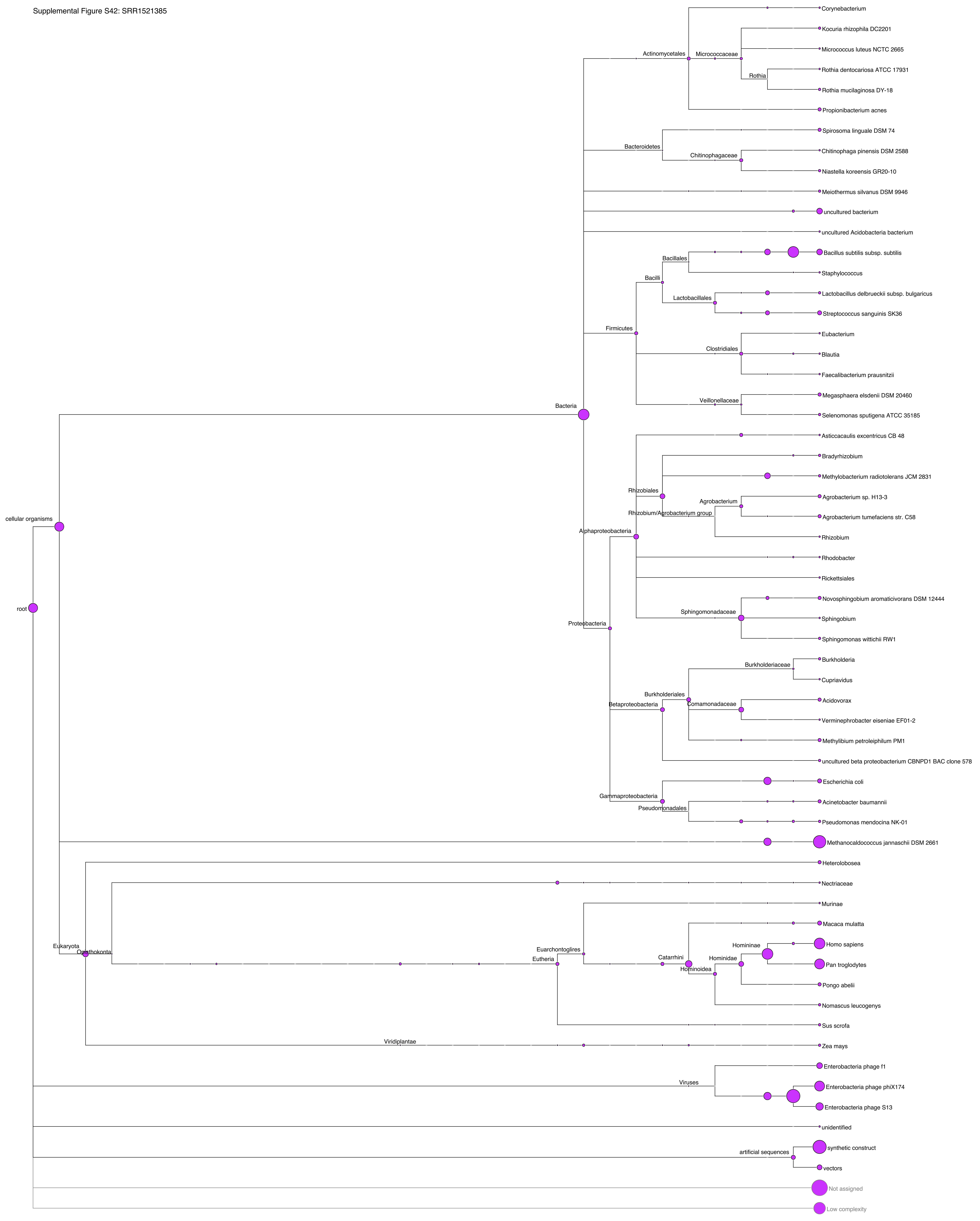

Supplemental Figure S43: SRR1521386

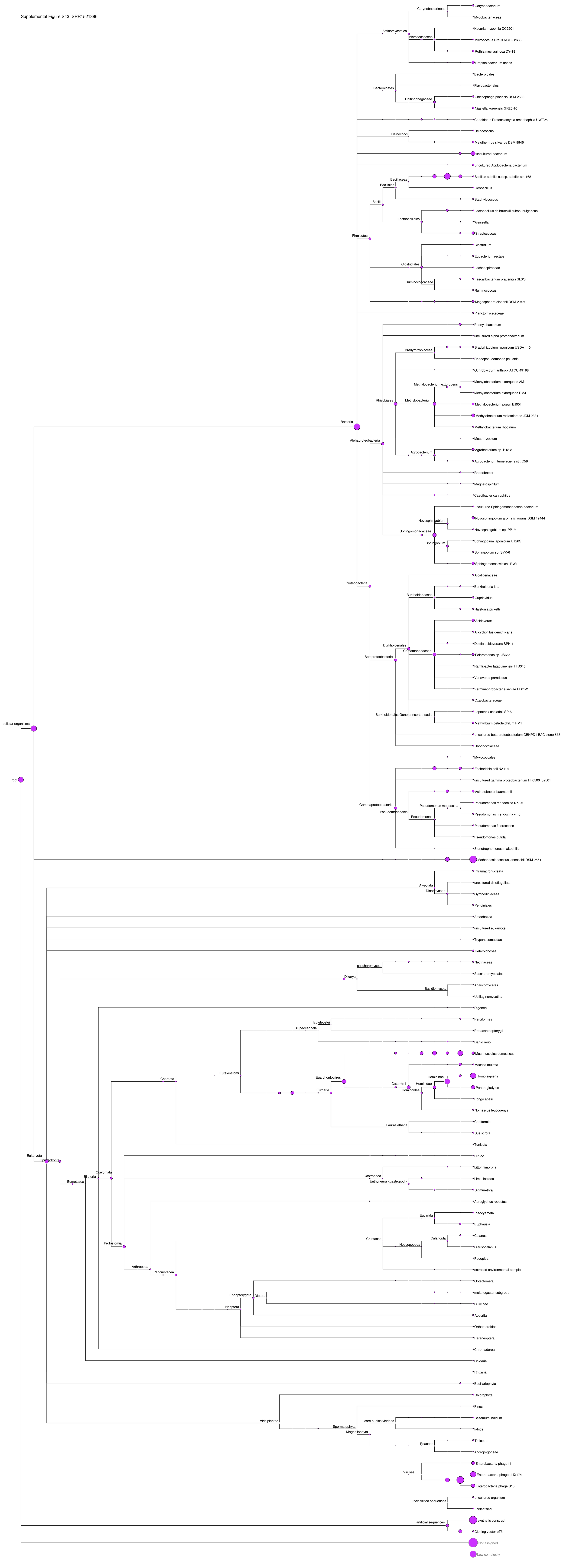

Supplemental Figure S44: SRR1521387

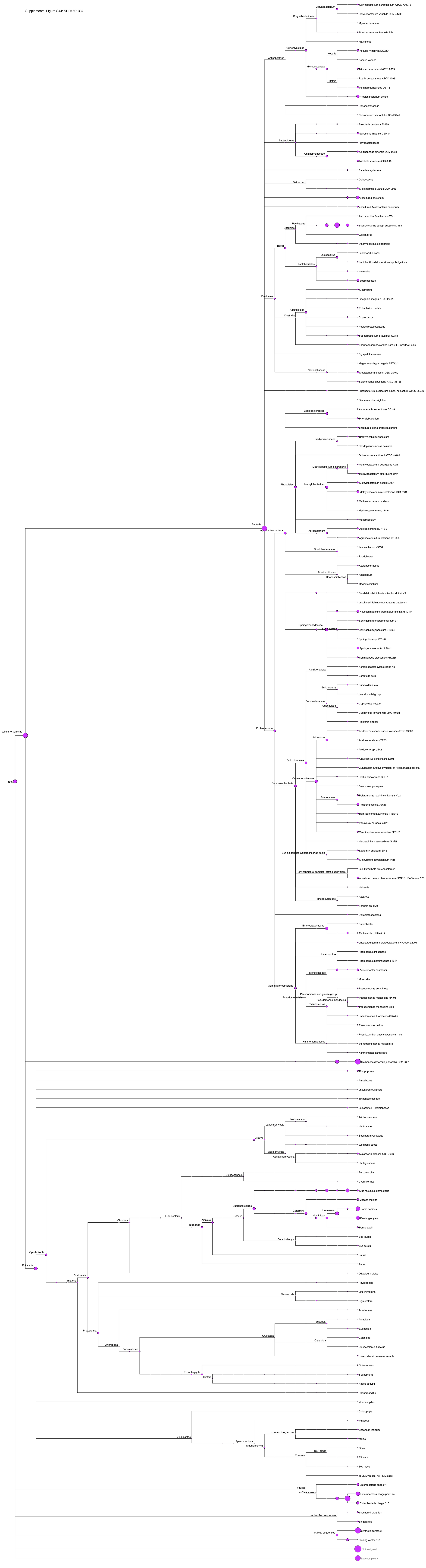

Supplemental Figure S45: SRR1521388

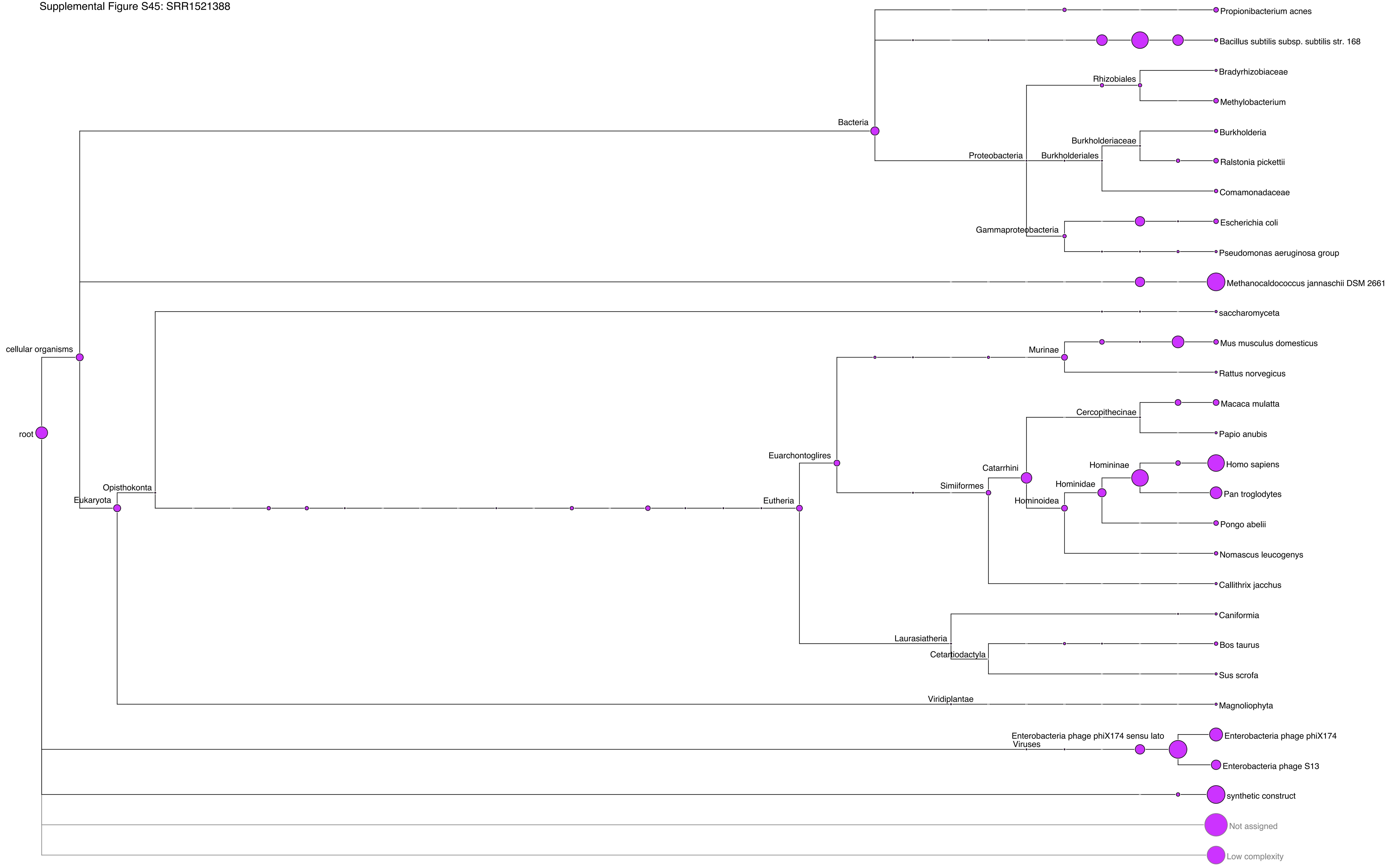

Supplemental Figure S46: SRR1521389

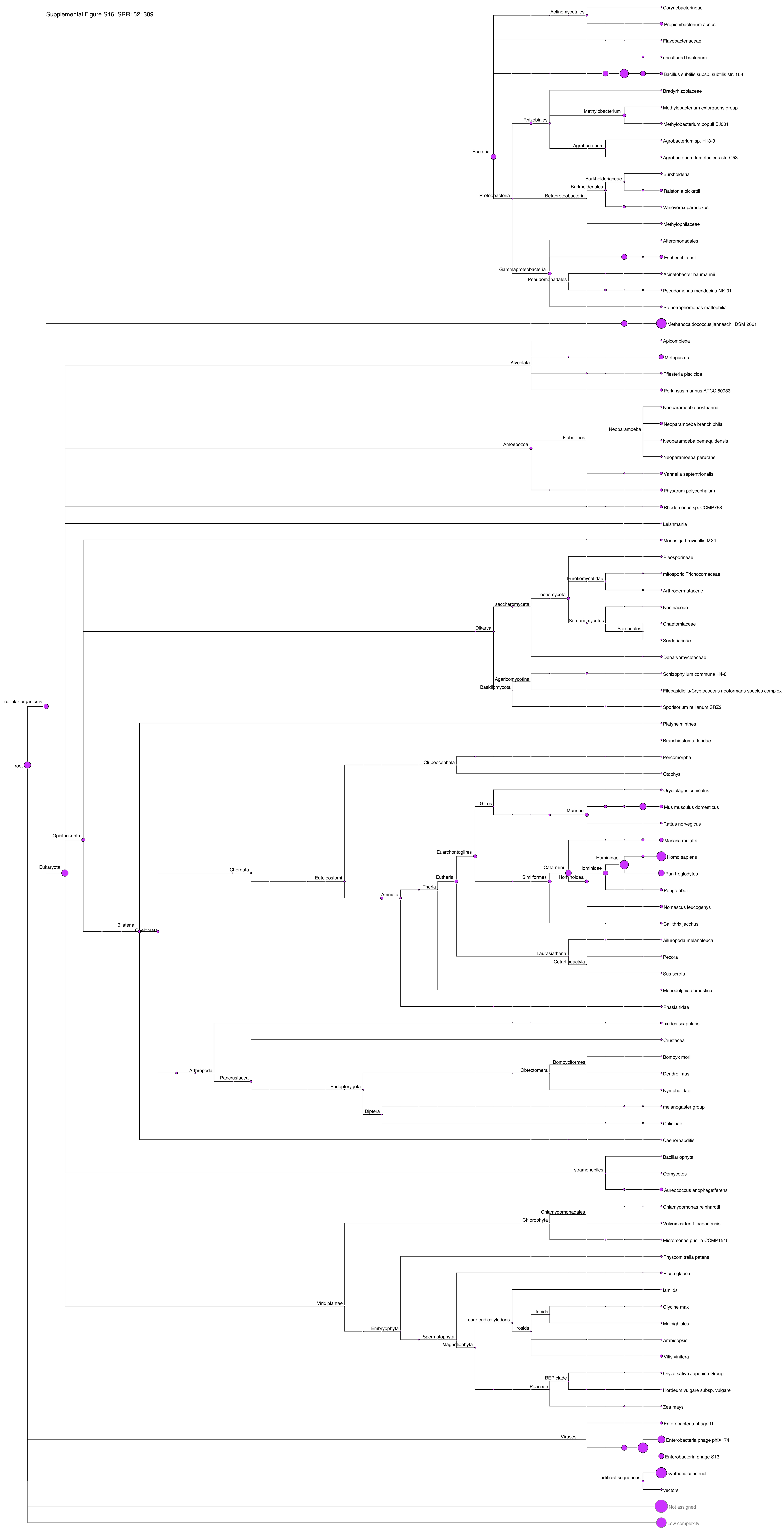

Supplemental Figure S47: SRR1521390

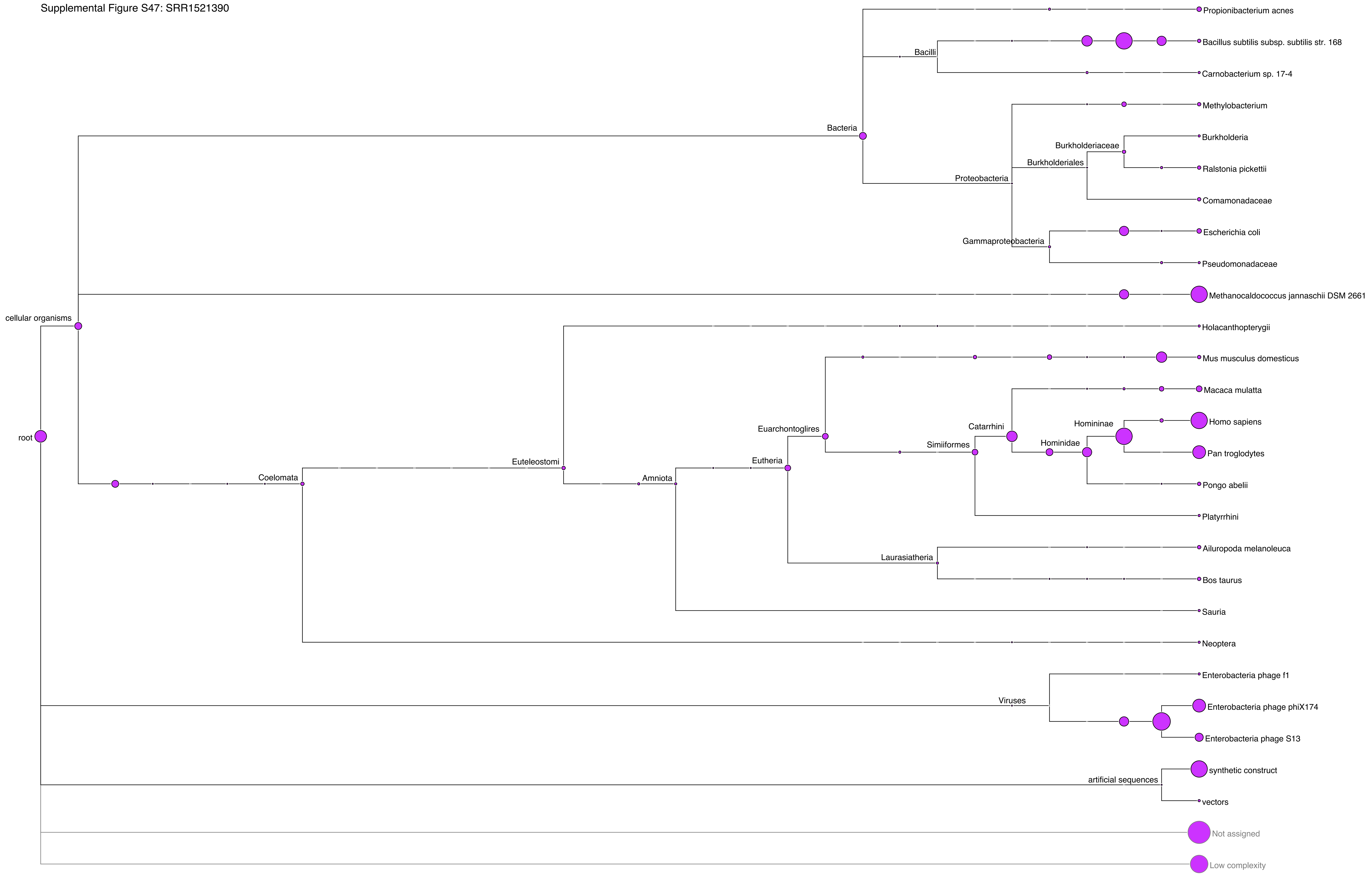

Supplemental Figure S48: SRR1521391

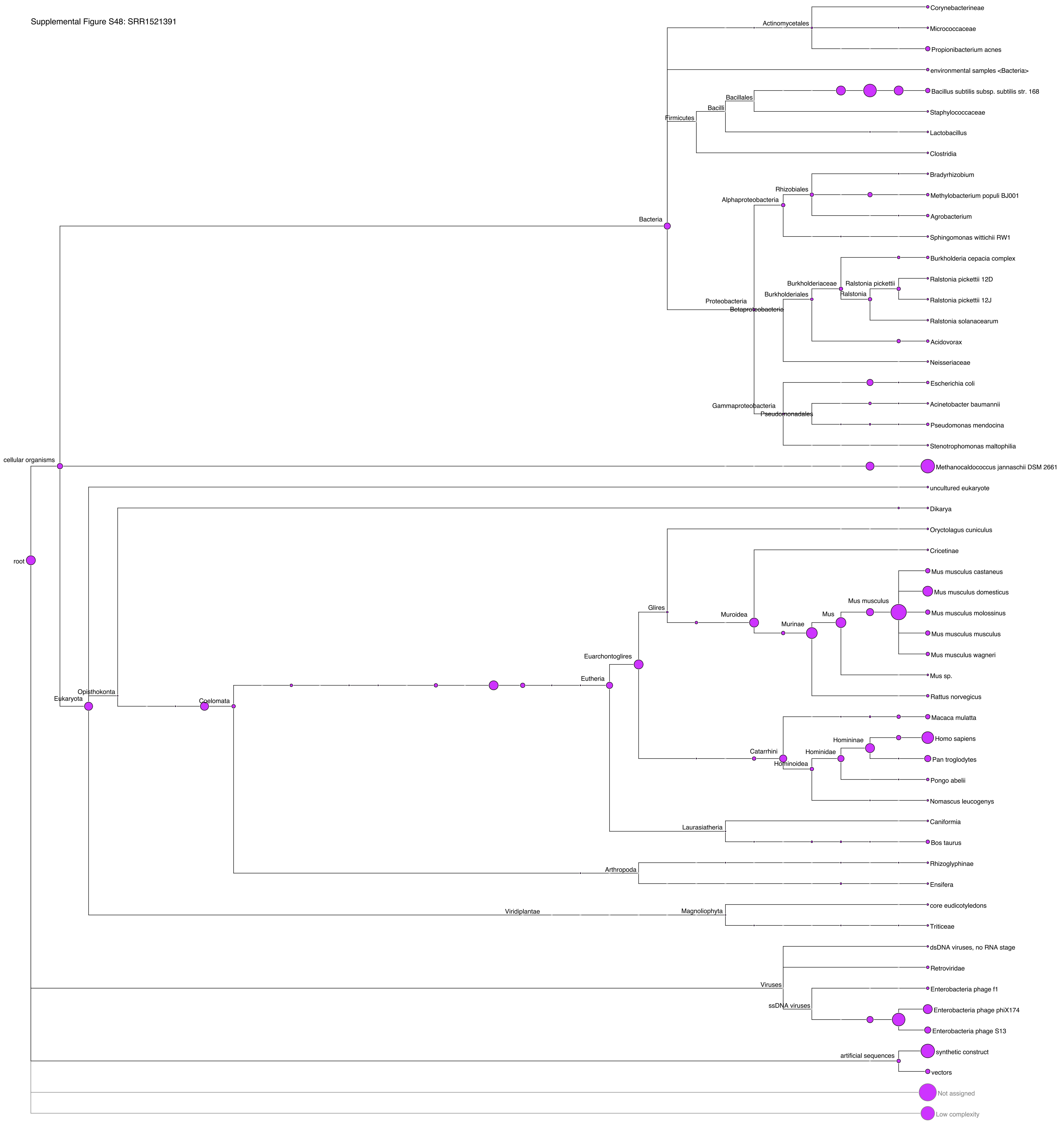

Supplemental Figure S49: SRR1521392

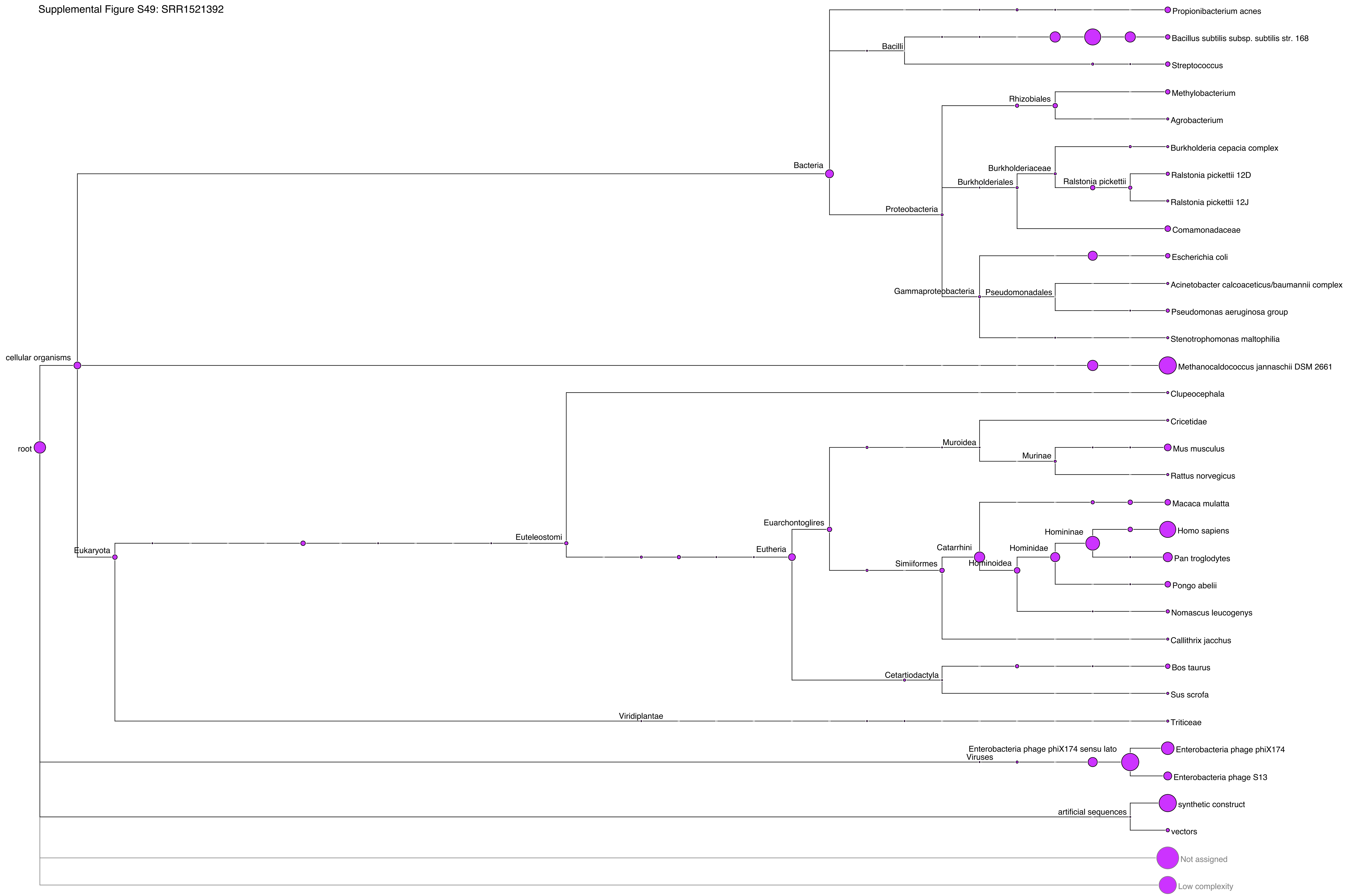

Supplemental Figure S50: SRR1521393

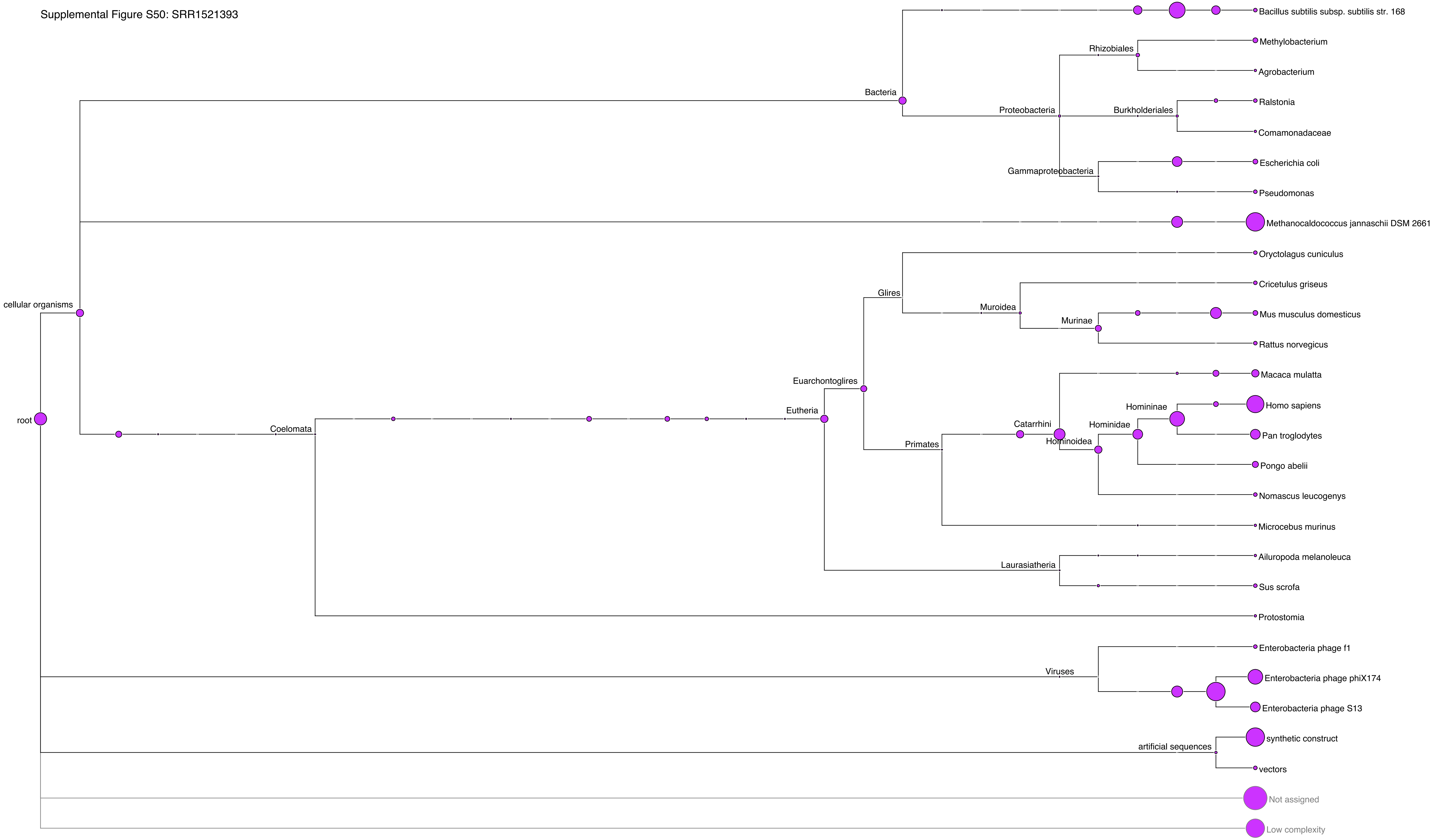

Supplemental Figure S51: SRR1521394

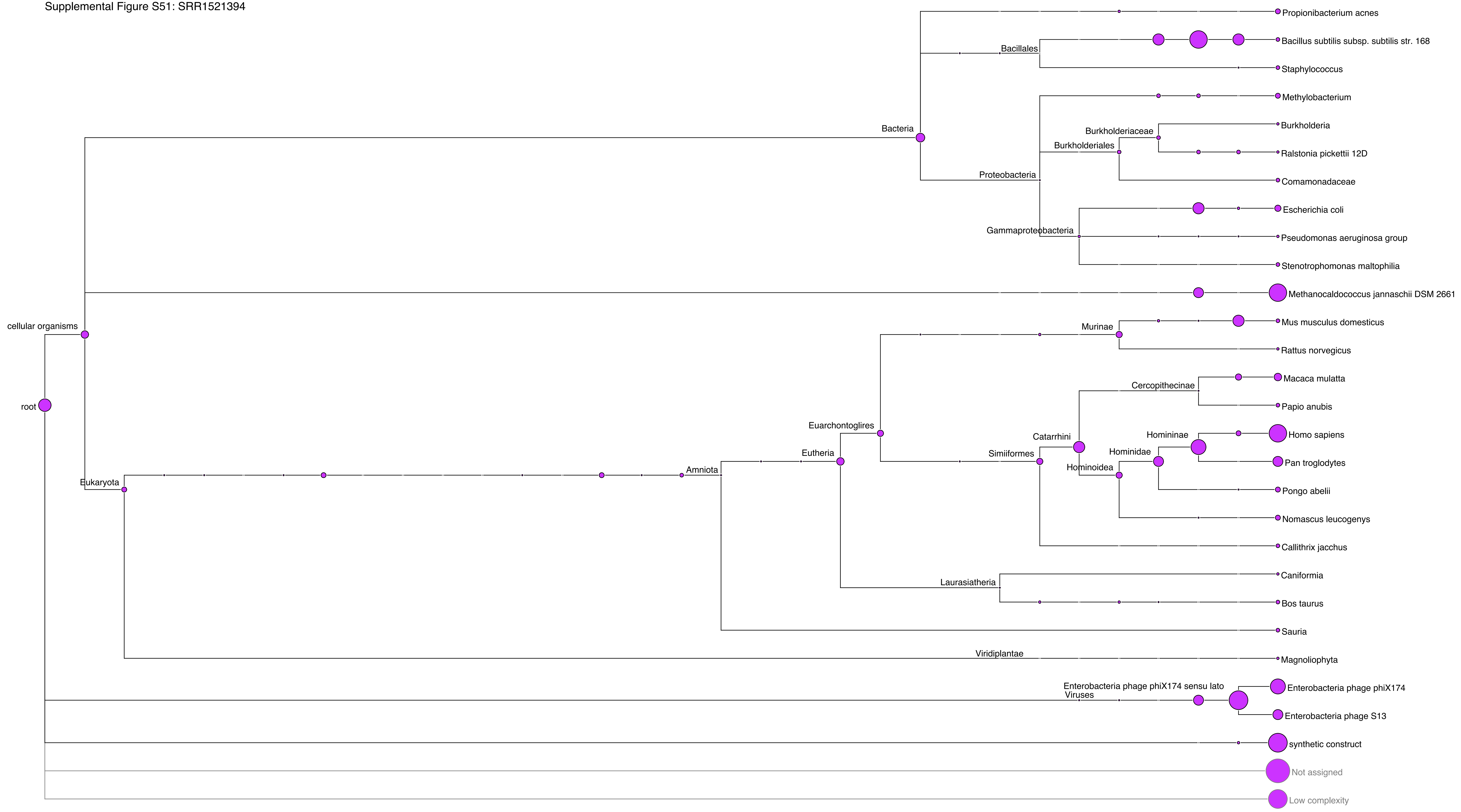

Supplemental Figure S52: SRR1521395

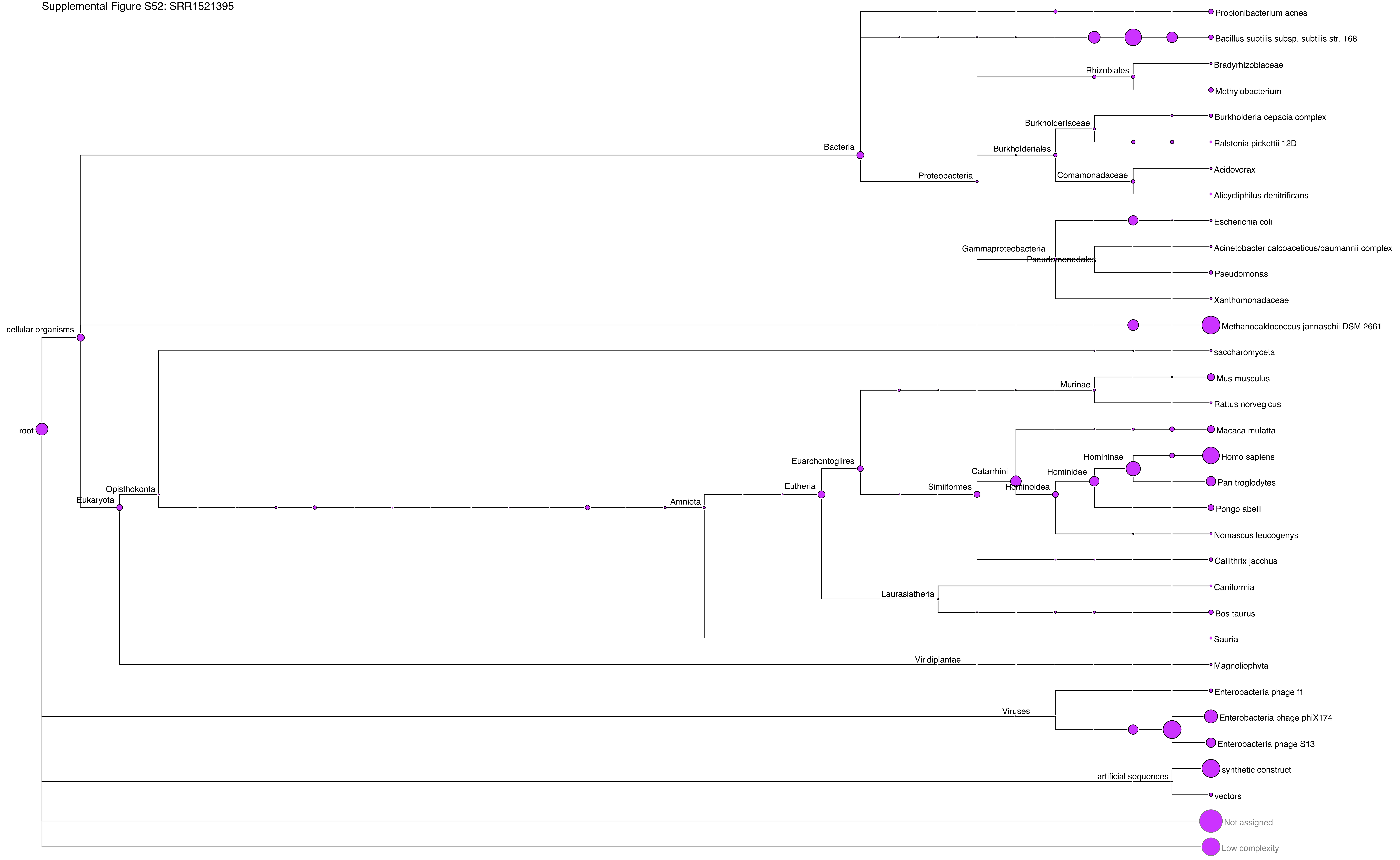

Supplemental Figure S53: SRR1521396

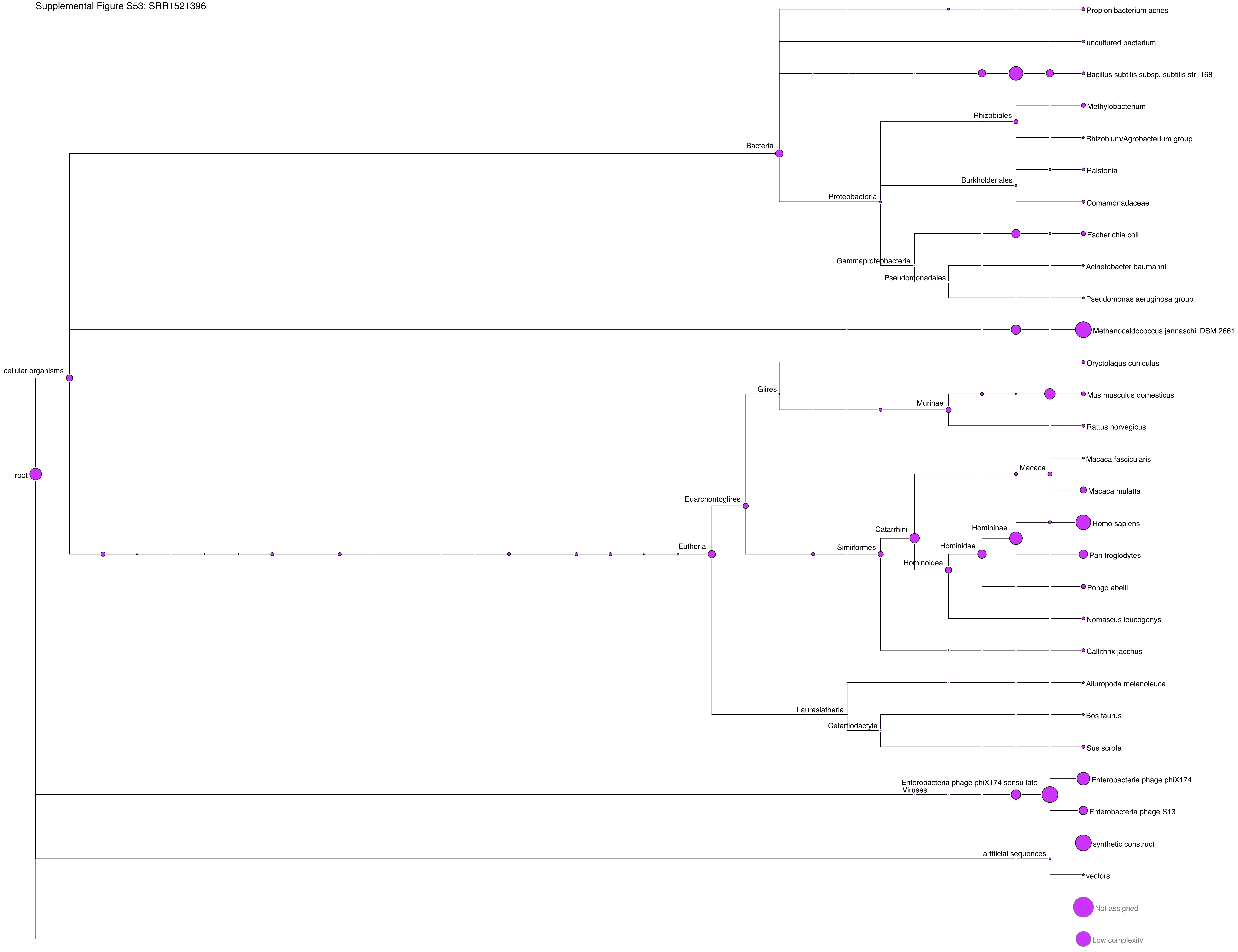

Supplemental Figure S54: SRR1521397

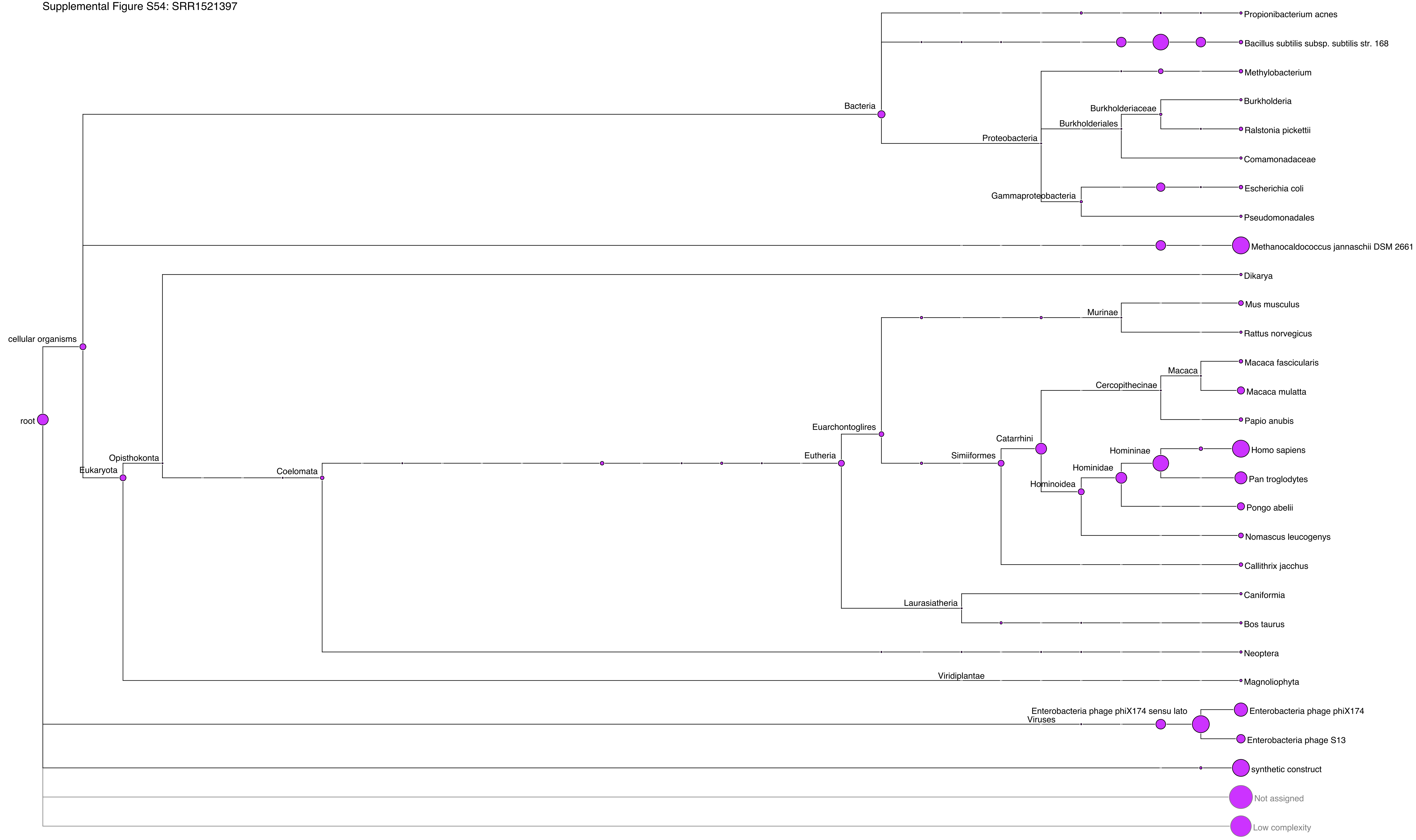

Supplemental Figure S55: SRR1521398

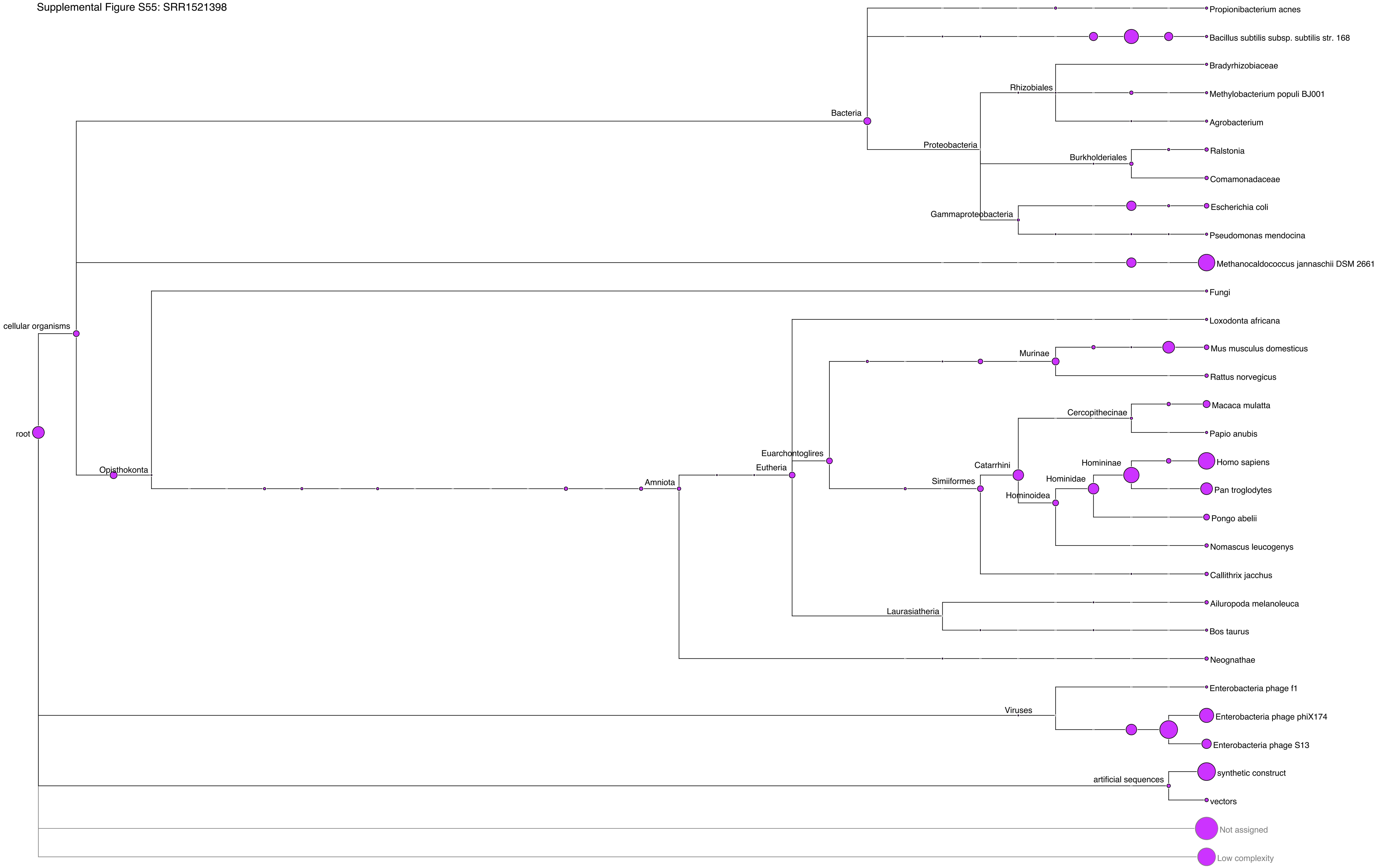

Supplemental Figure S56: SRR1521399

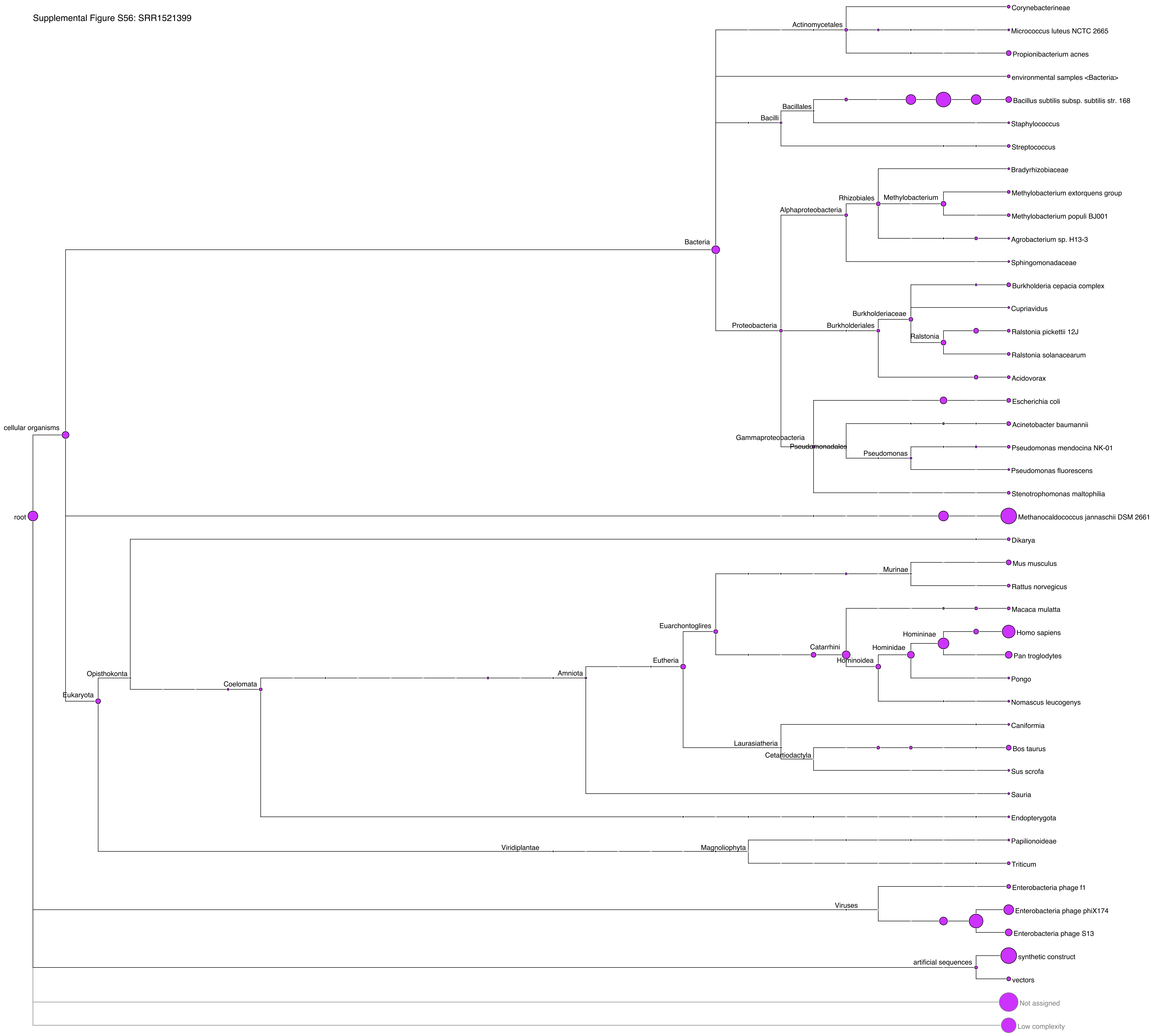

Supplemental Figure S57: SRR1521400

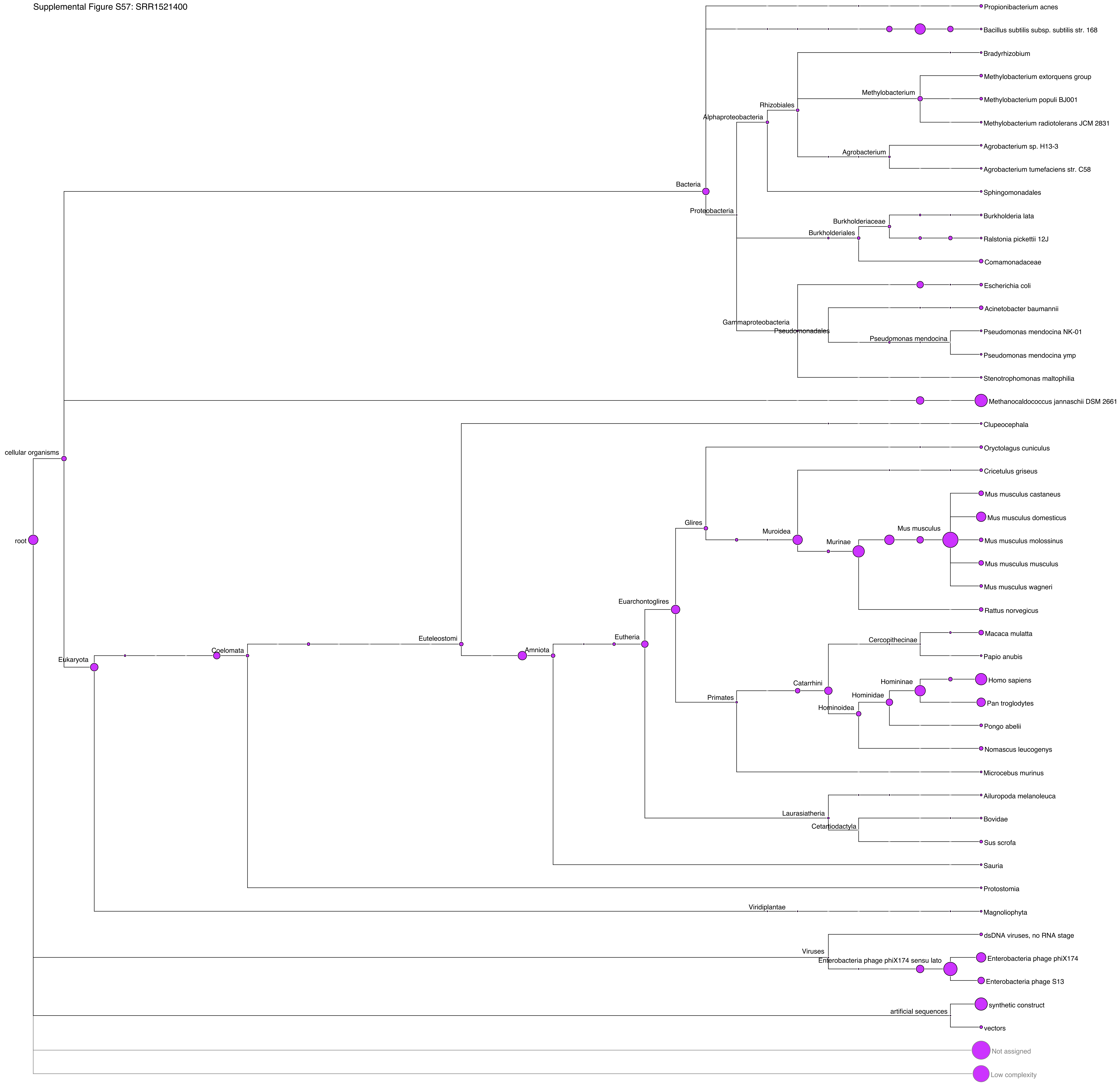

Supplemental Figure S58: SRR1521401

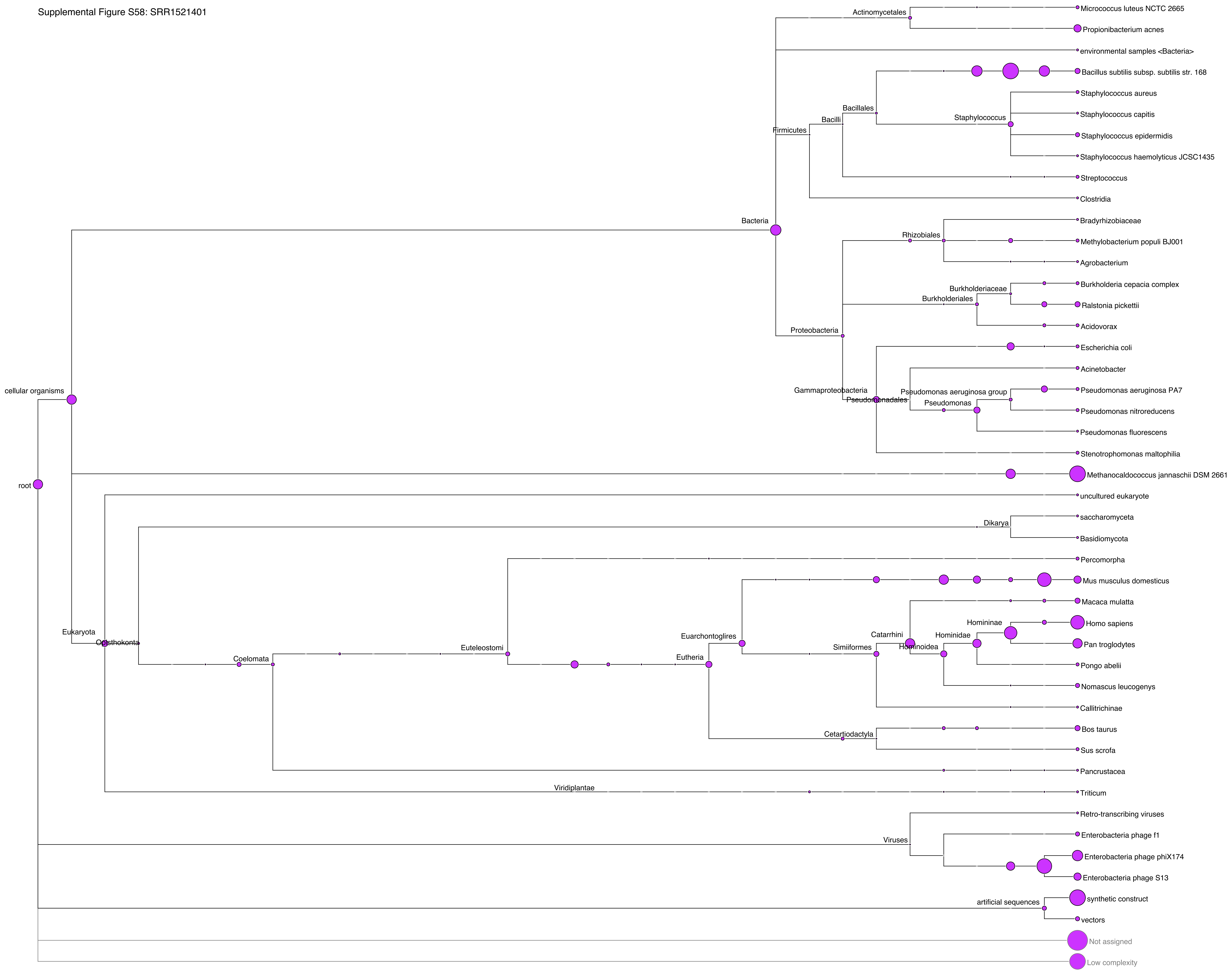

Supplemental Figure S59: SRR1521402

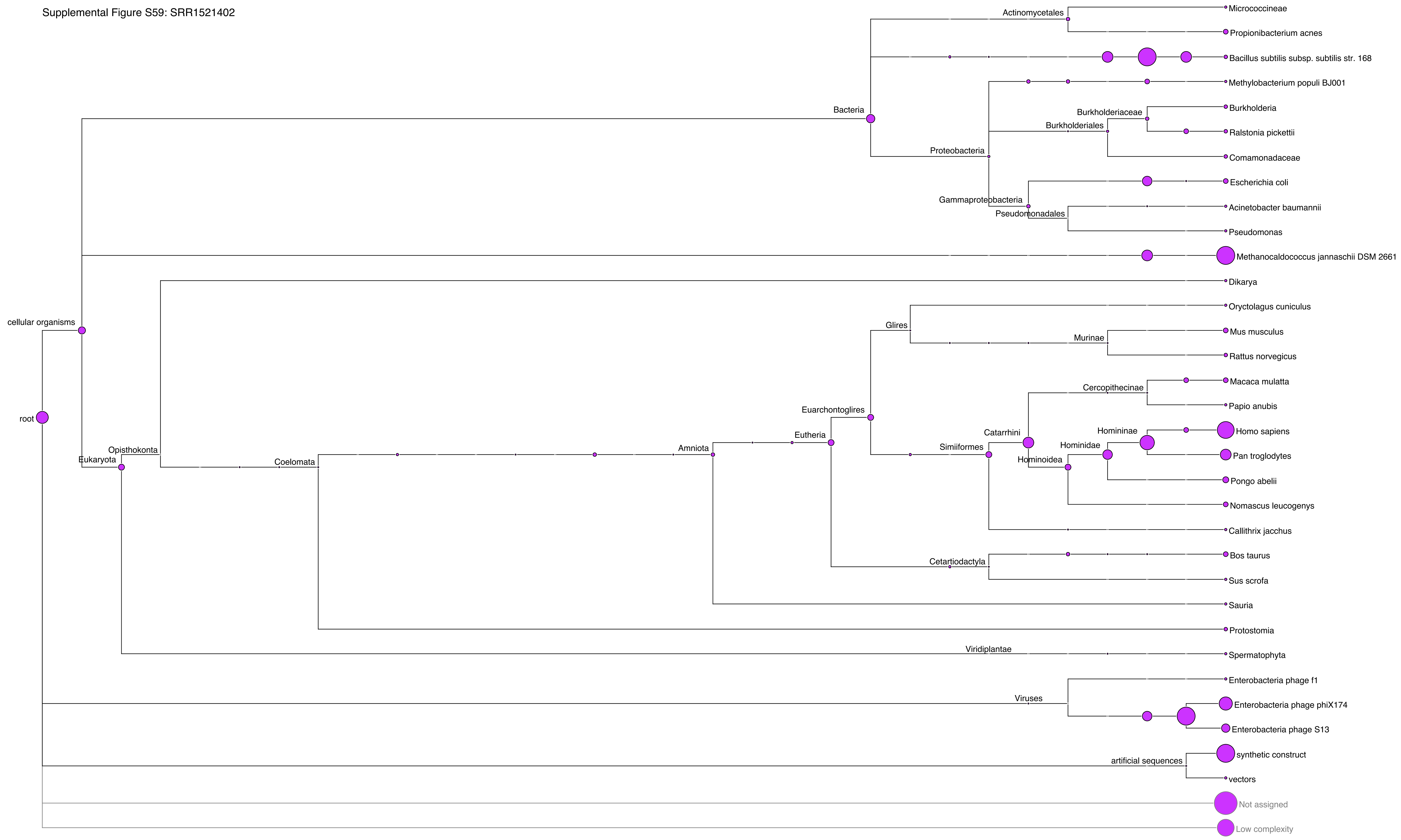

Supplemental Figure S60: SRR1521403

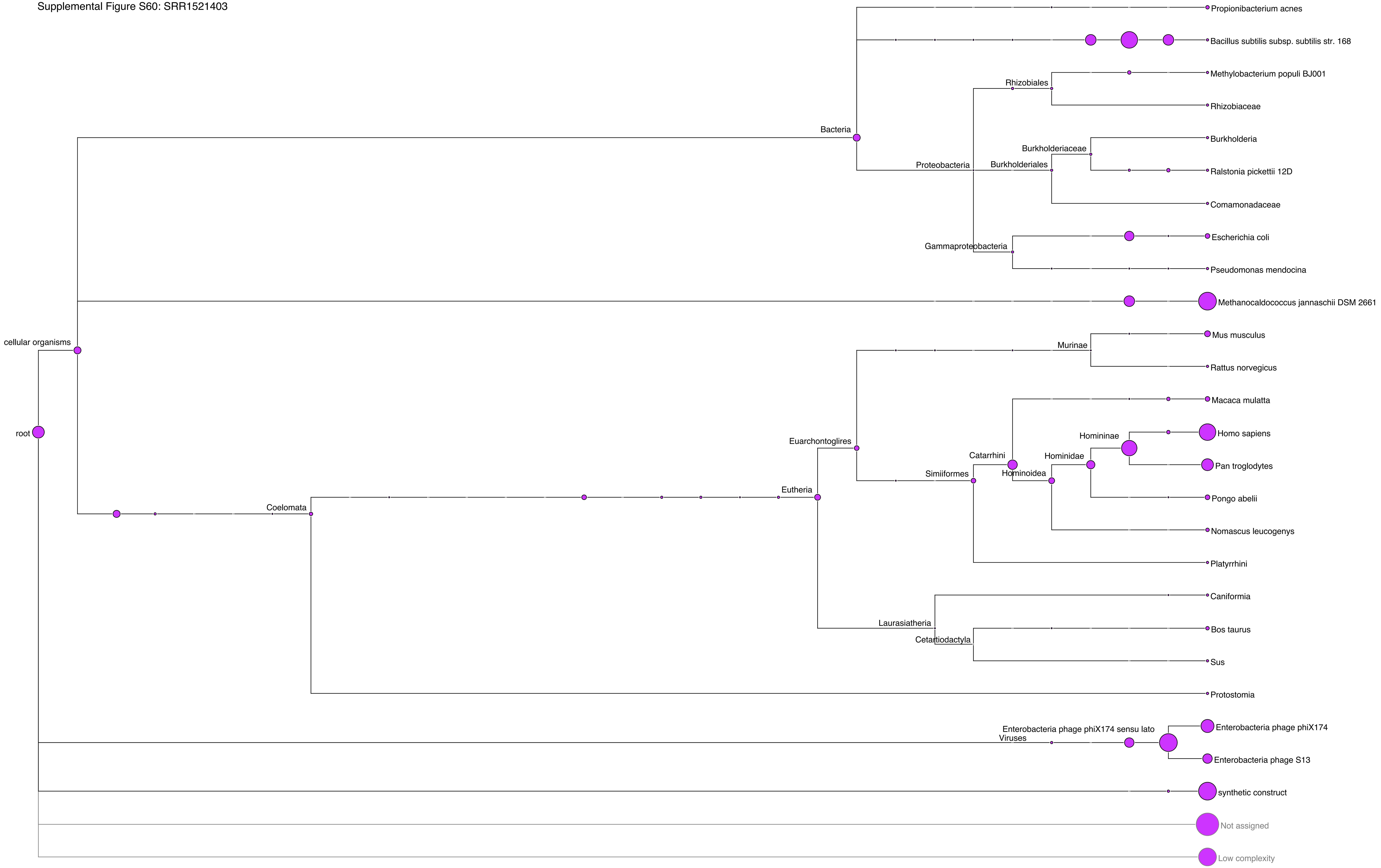

Supplemental Figure S61: SRR1521404

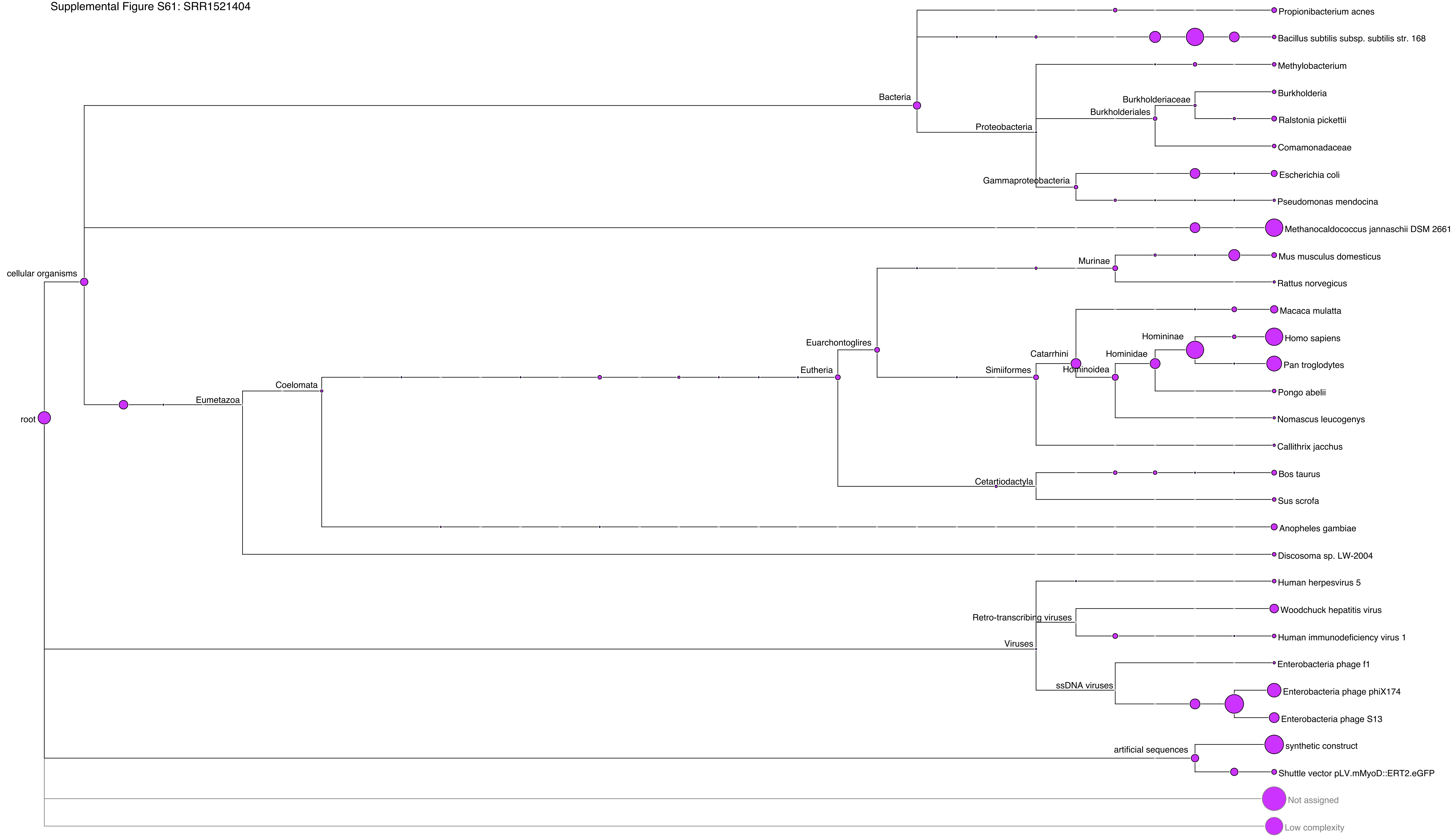

Supplemental Figure S62: SRR1521405

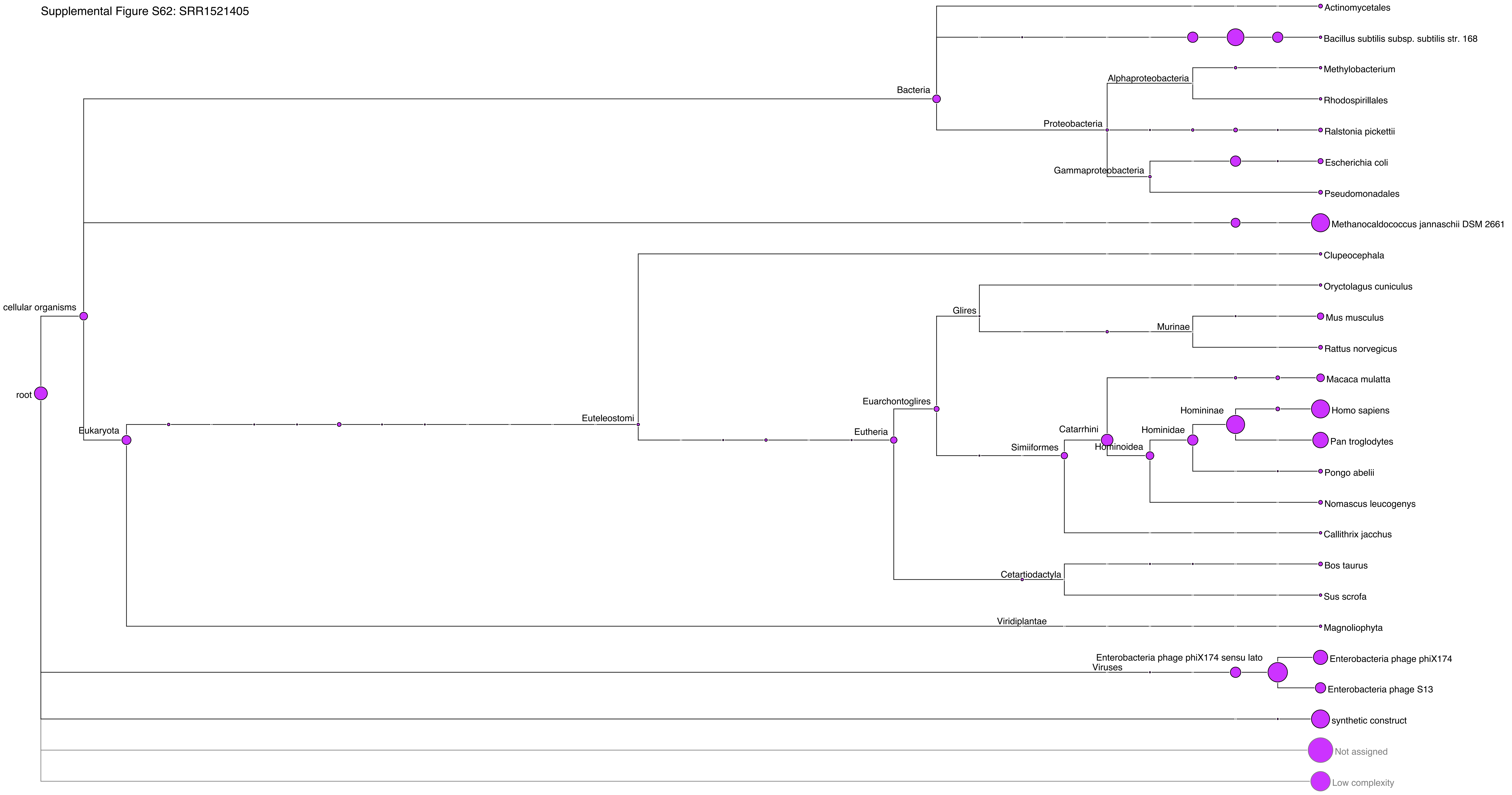

Supplemental Figure S63: SRR1521406

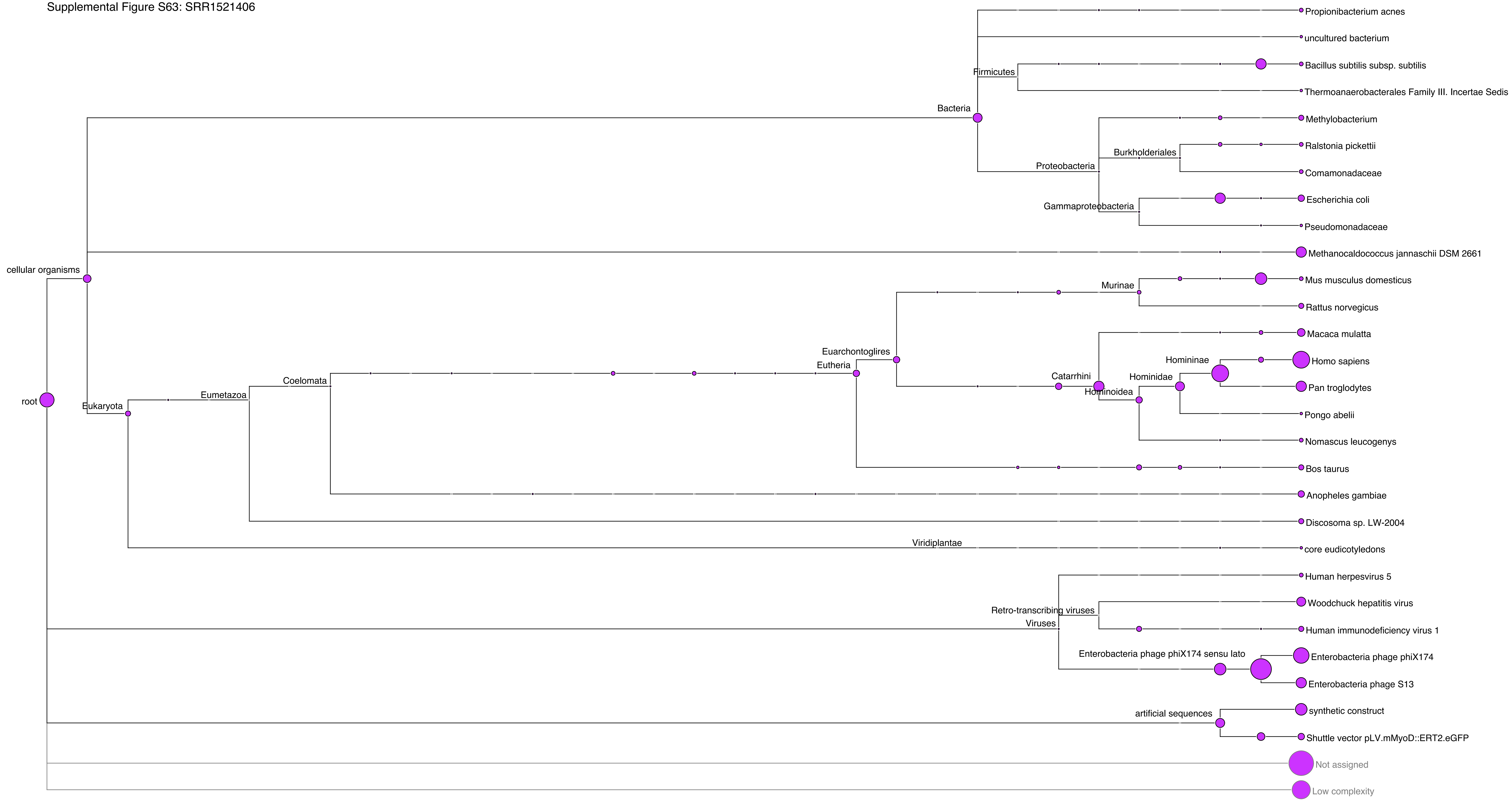

Supplemental Figure S64: SRR1521407

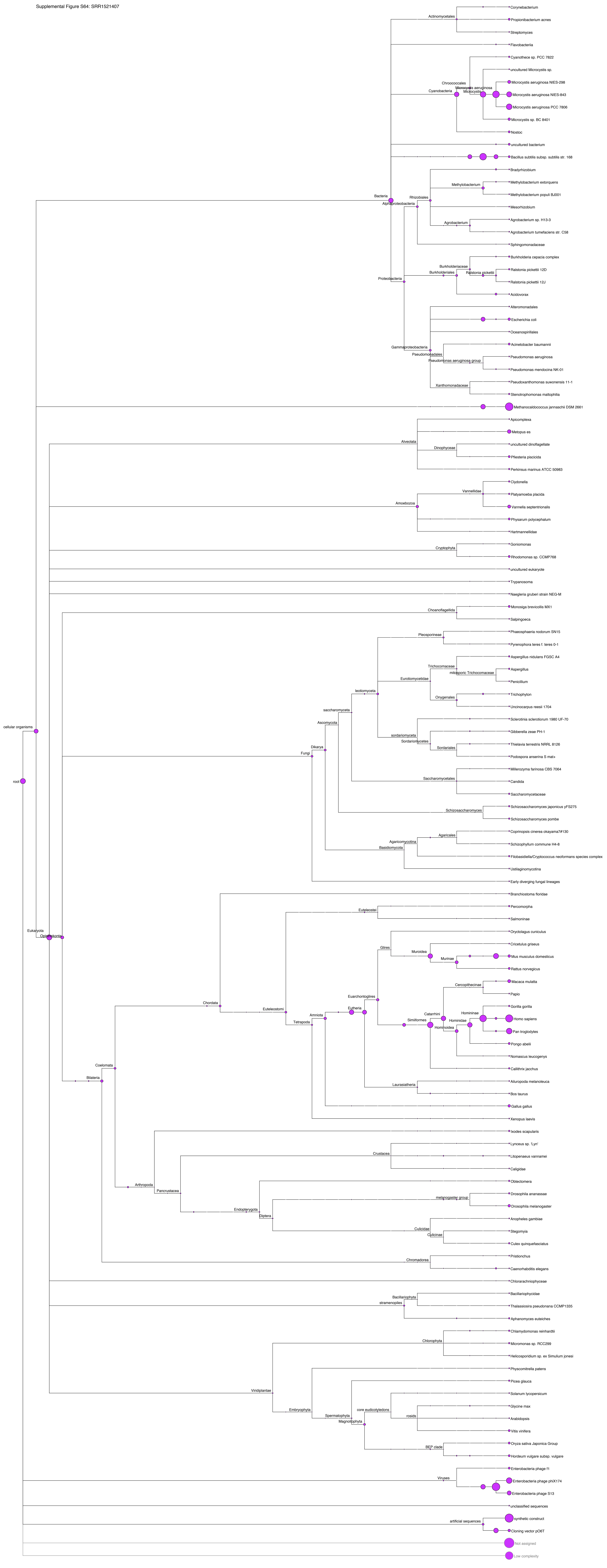

Supplemental Figure S65: SRR1521408

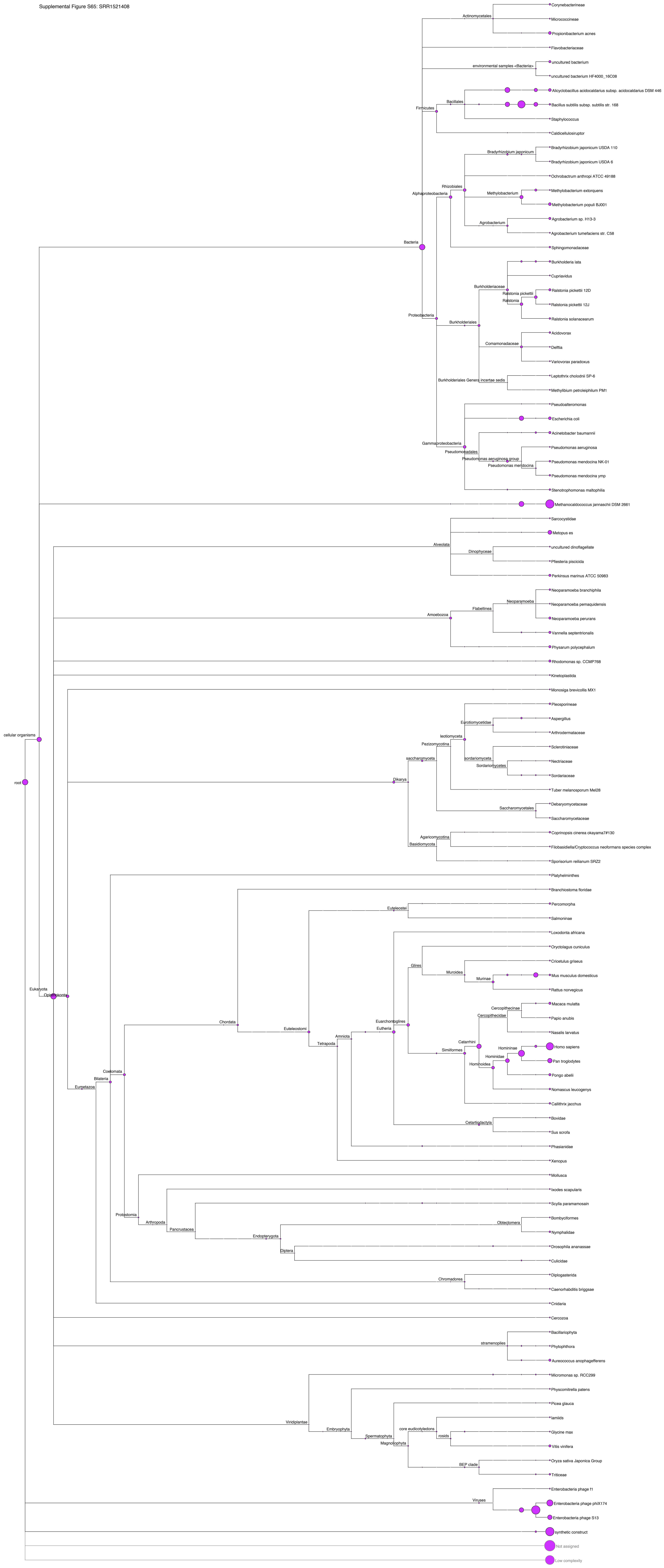

Supplemental Figure S66: SRR1521409

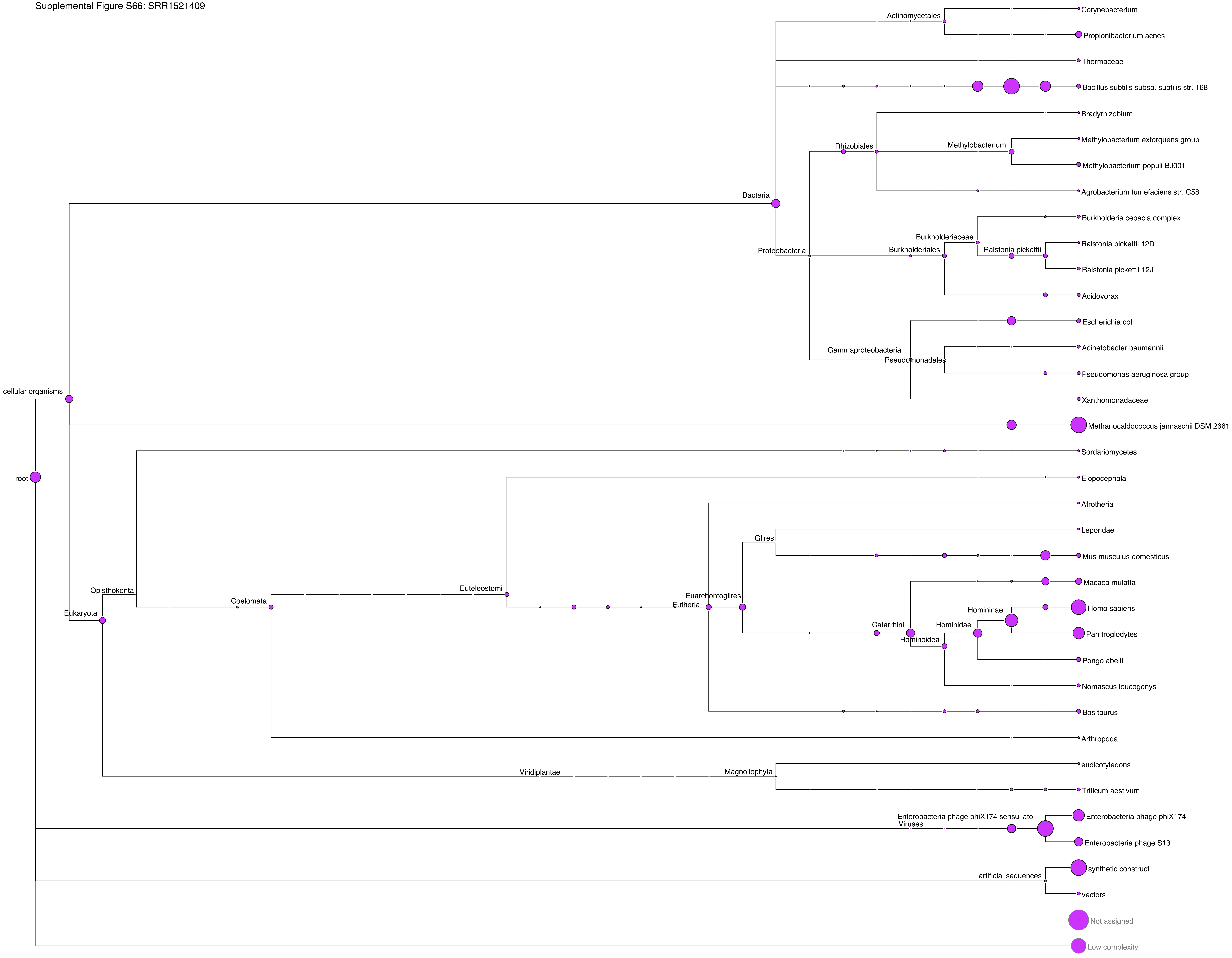

Supplemental Figure S67: SRR1521410

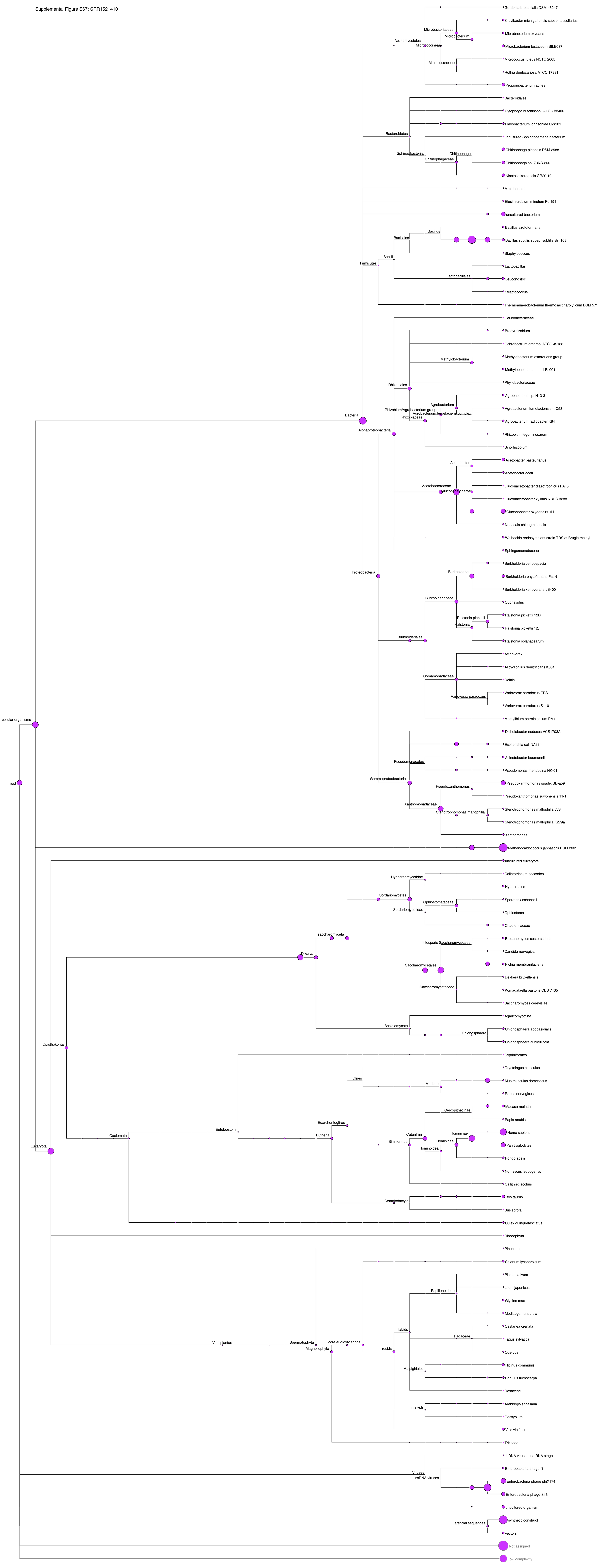

Supplemental Figure S66: SRR1521411

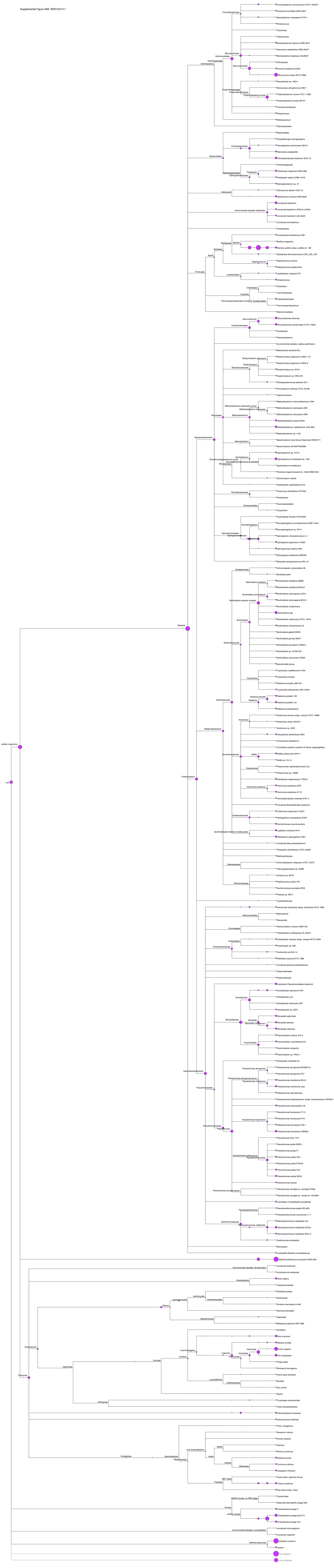

Supplemental Figure S69: SRR1521412

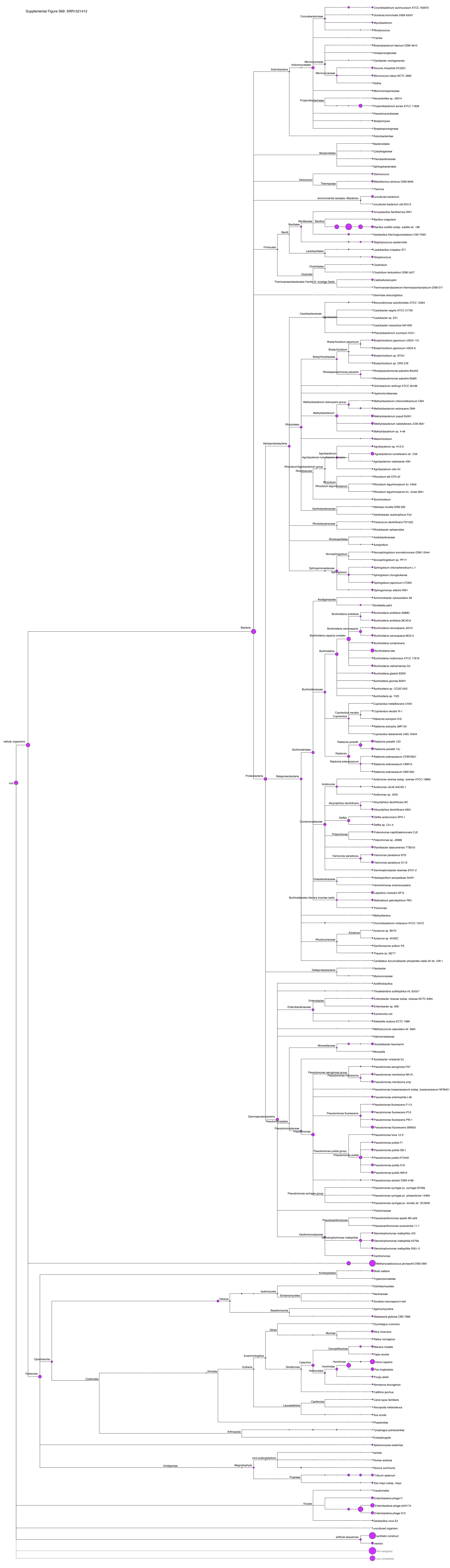

Supplemental Figure S70: SRR1521413

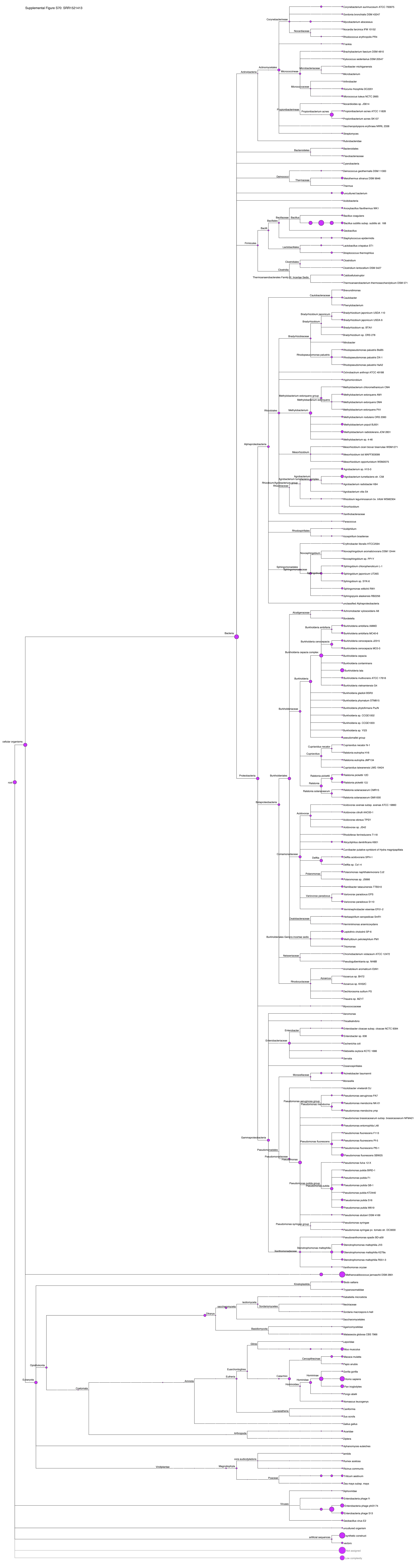

Supplemental Figure S71: SRR1521414

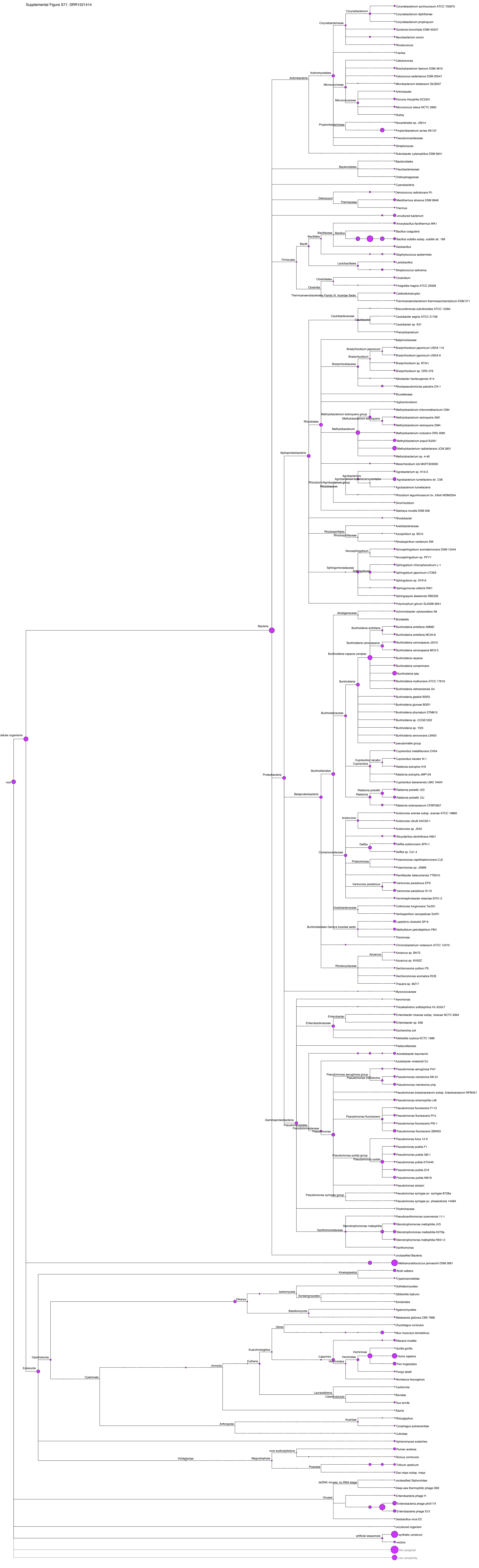

Supplemental Figure S72: SRR1521415

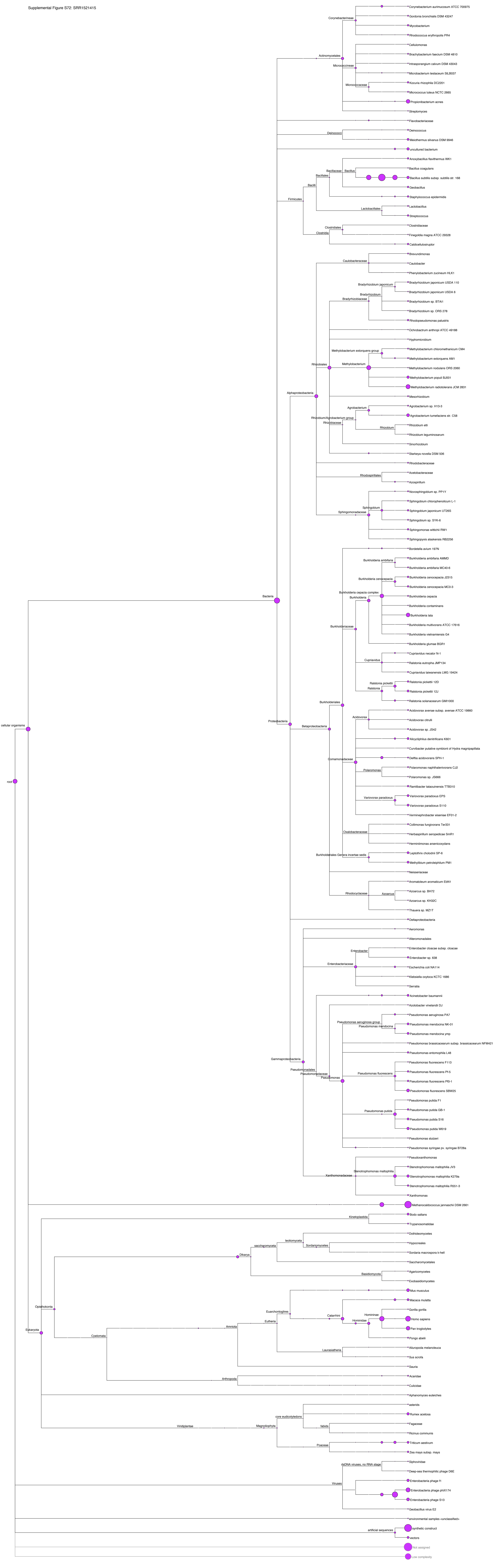

Supplemental Figure S73: SRR1521416

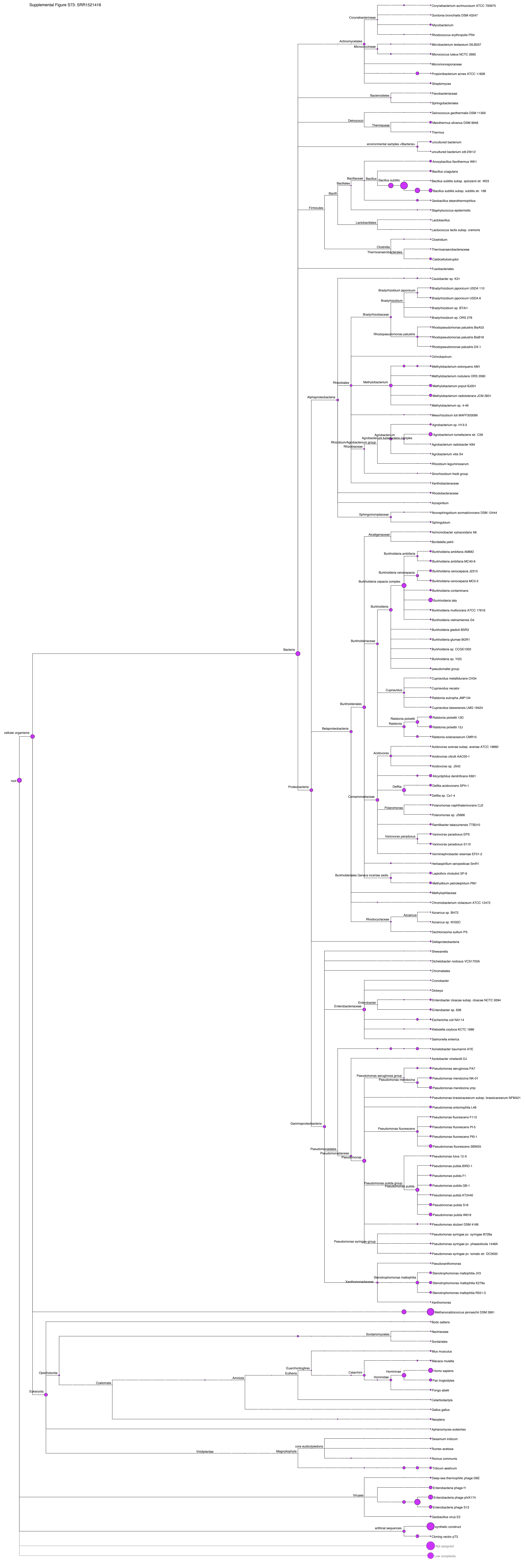

Supplemental Figure S74: SRR1521417

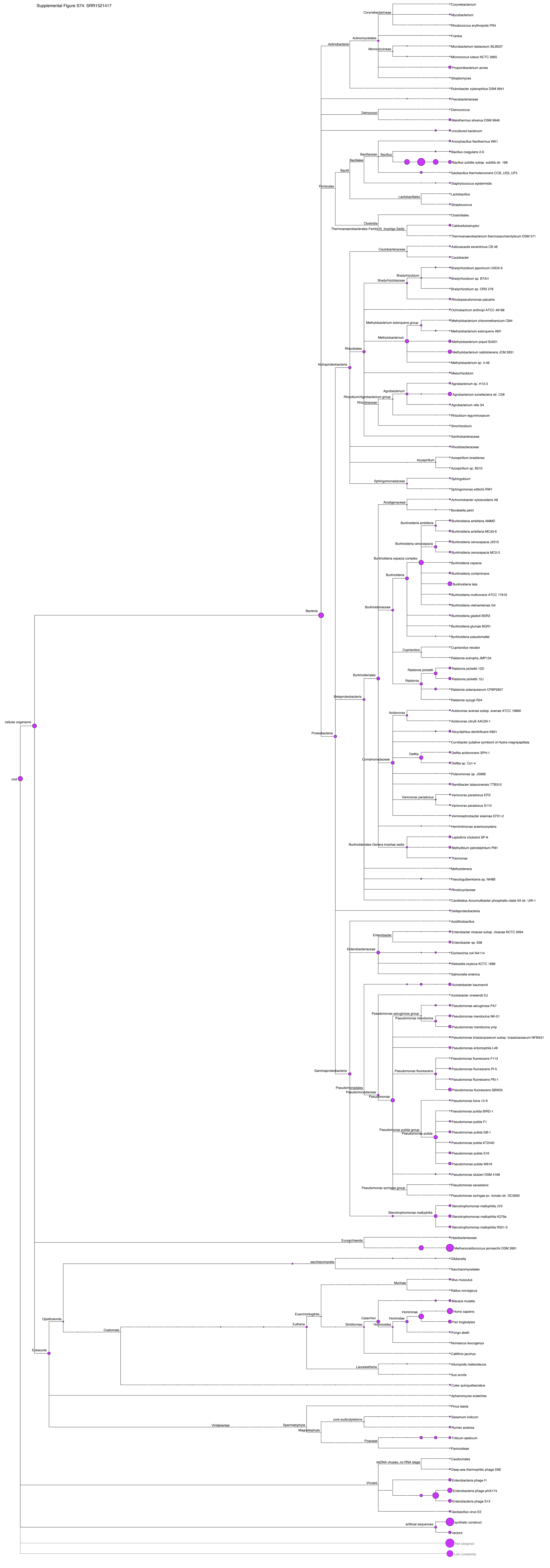

Supplemental Figure S75: SRR1521418

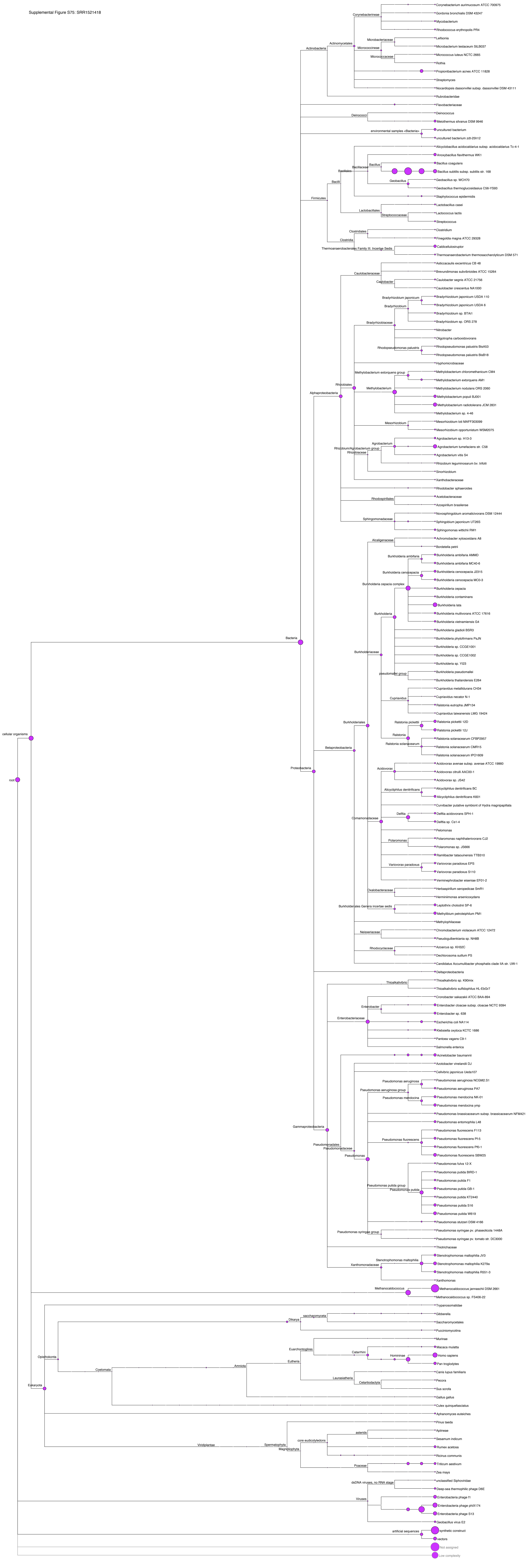

Supplemental Figure S76: SRR1521415

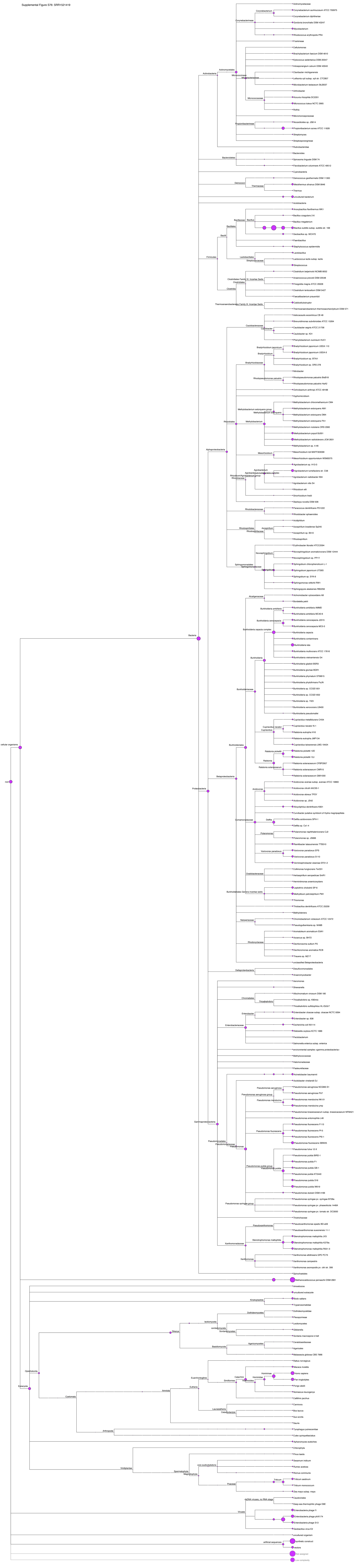

Supplemental Figure S77: SRR1521420

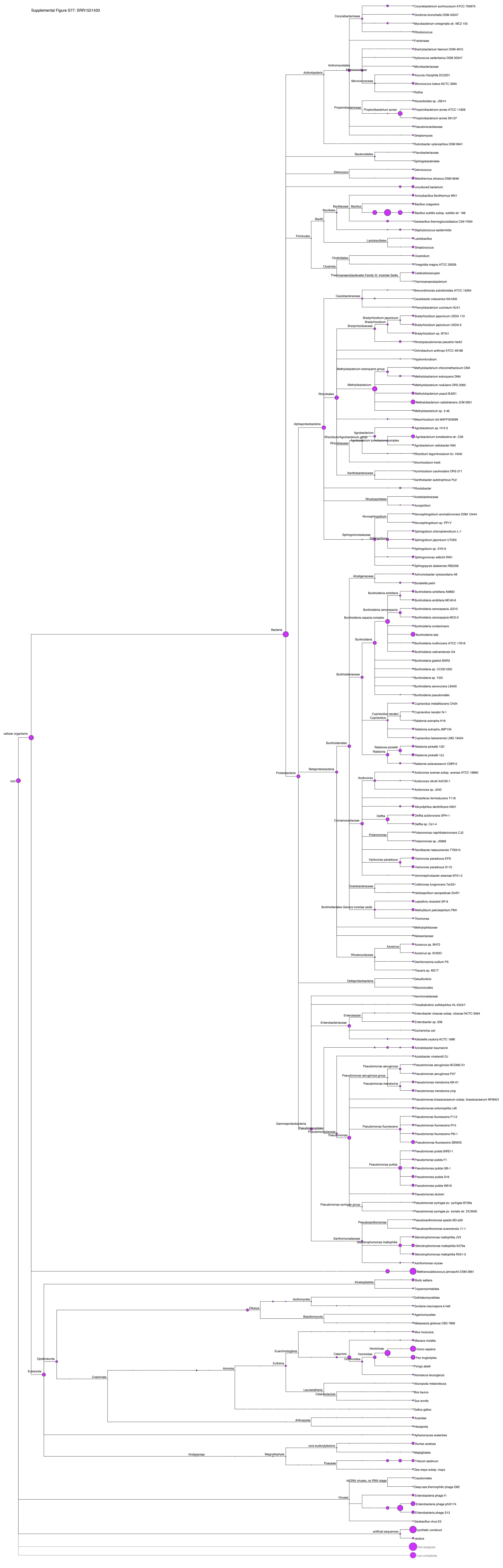

Supplemental Figure S78: SRR1521421

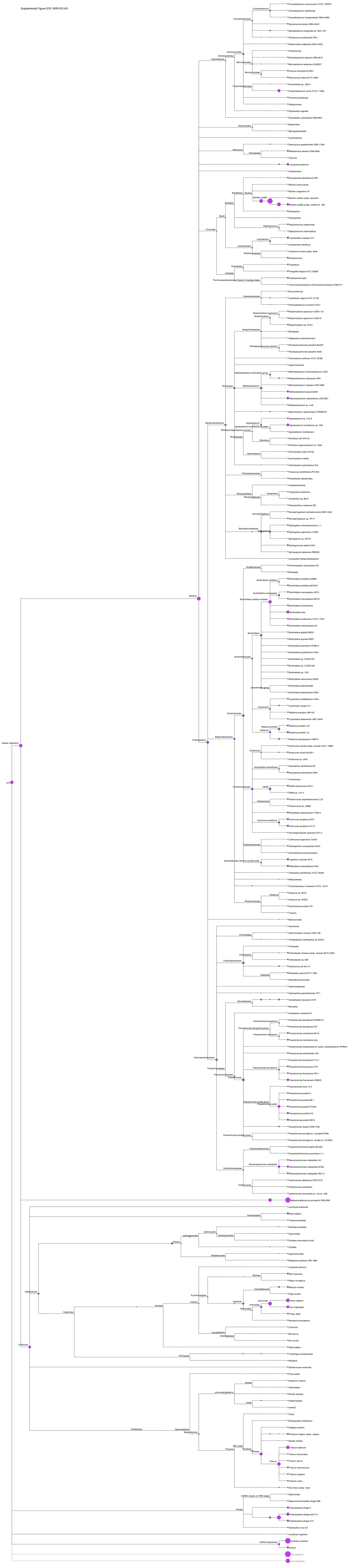

Supplemental Figure S79: SRR1521422

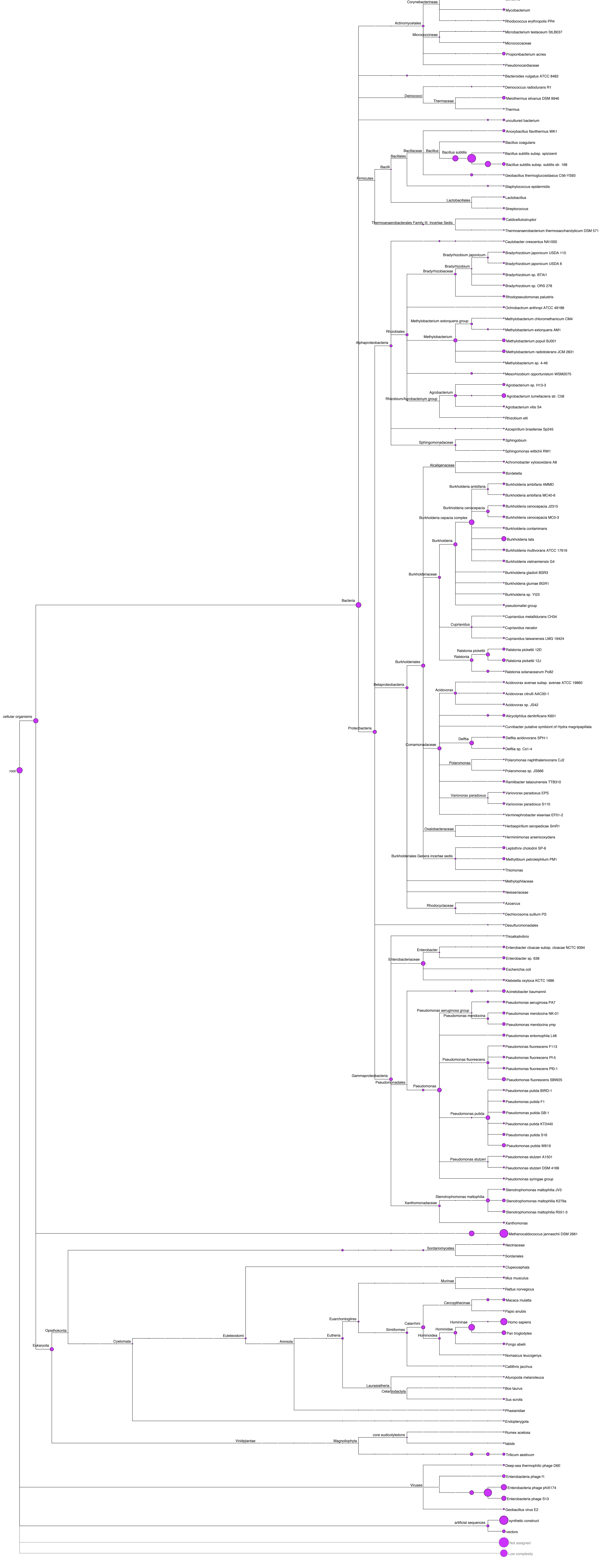

Supplemental Figure S80: SRR1521423

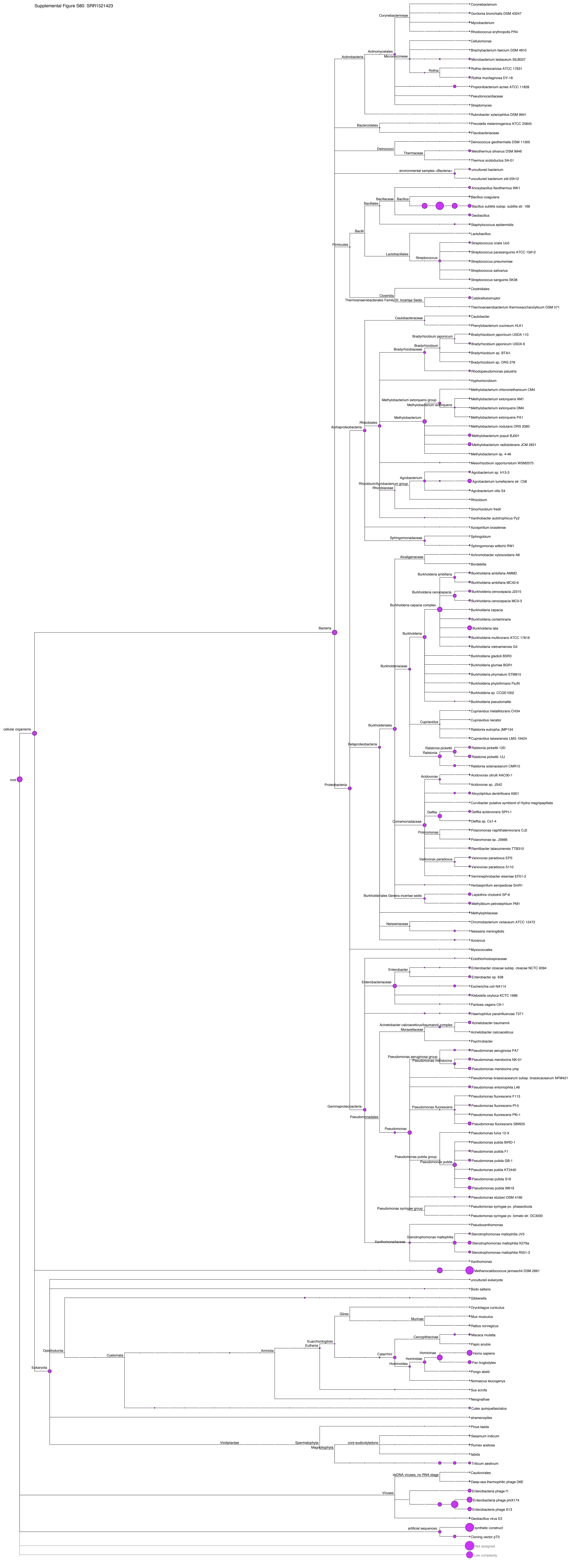

Supplemental Figure S81: SRR1521424

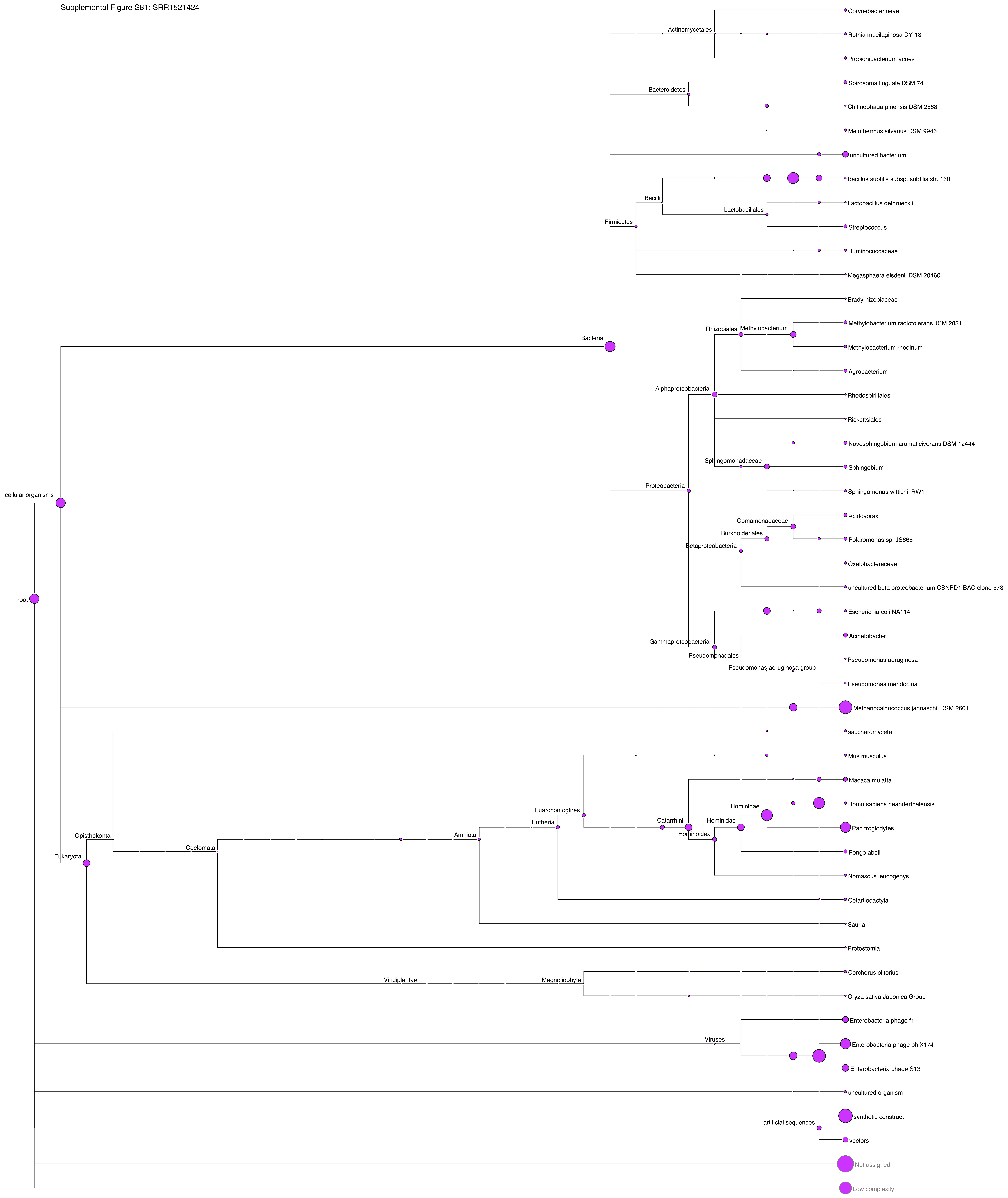

Supplemental Figure S82: SRR1521425

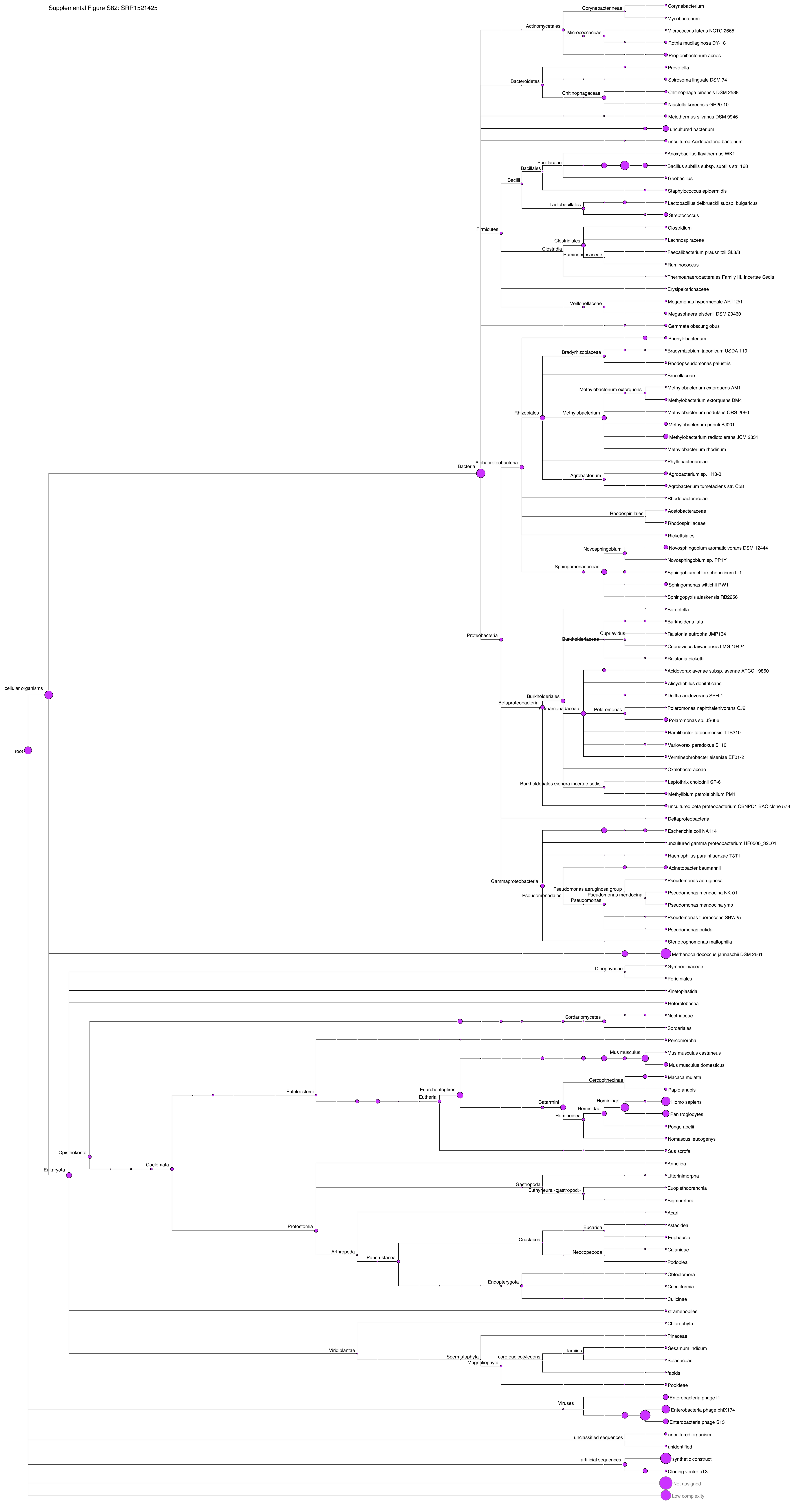

Supplemental Figure S83: SRR1521426

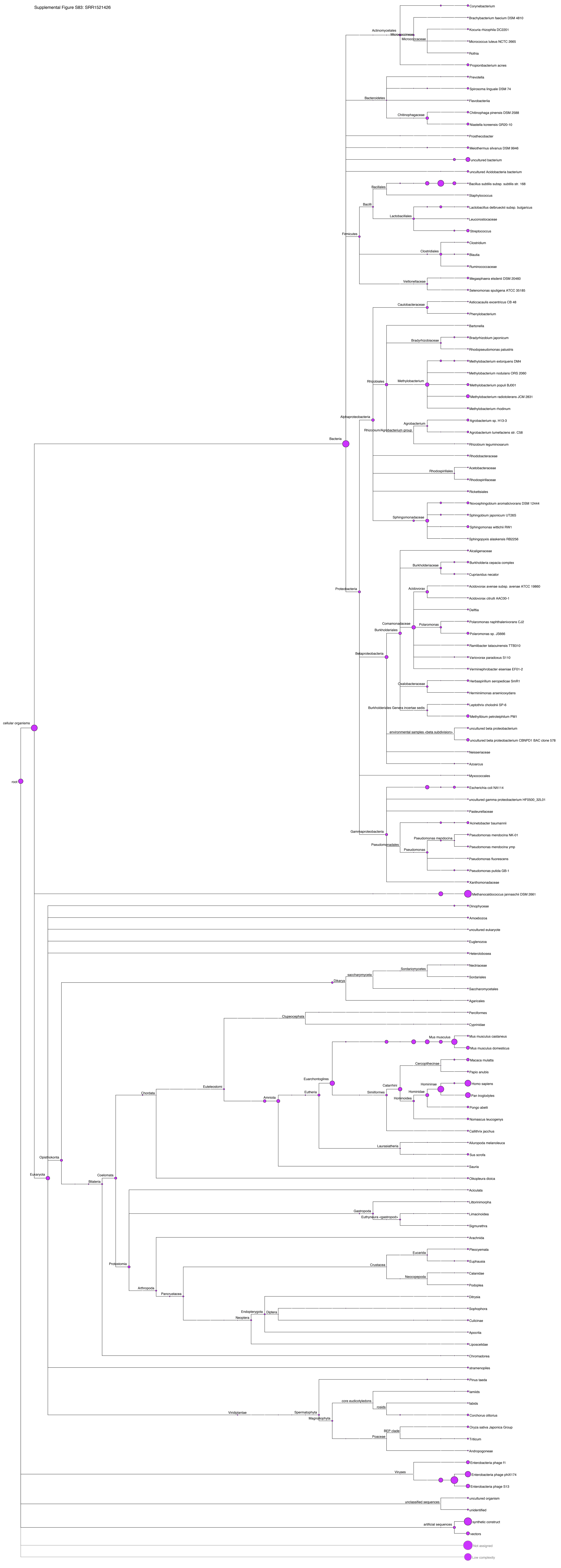

Supplemental Figure S84: SRR1521427

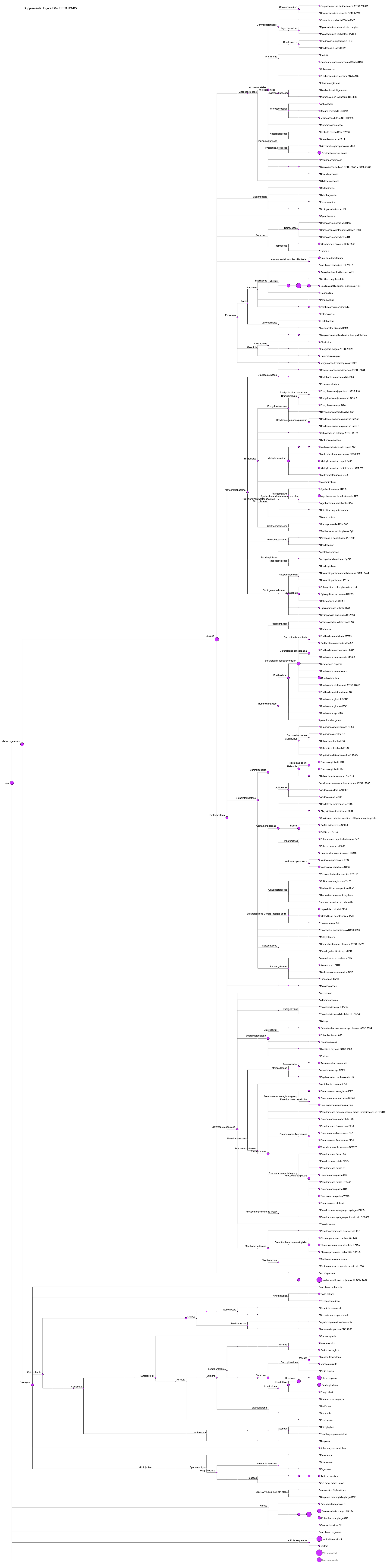

Supplemental Figure S85: SRR1521428

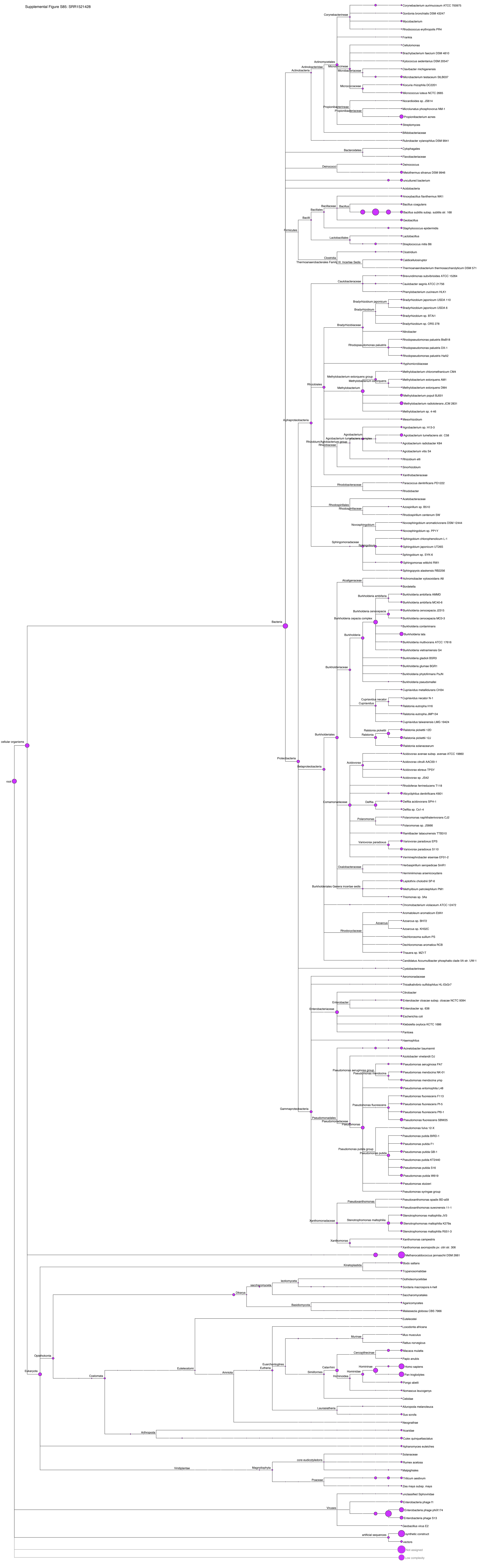

Supplemental Figure S86: SRR1521429

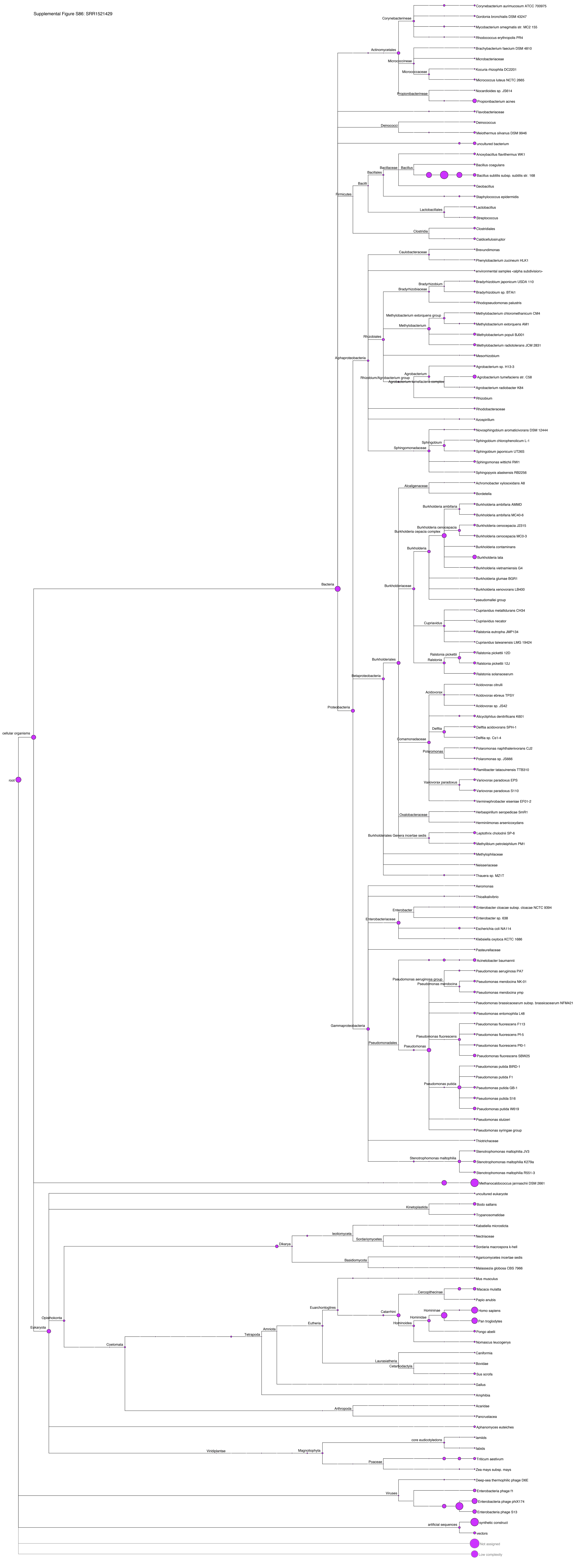

Supplemental Figure S87: SRR1521430

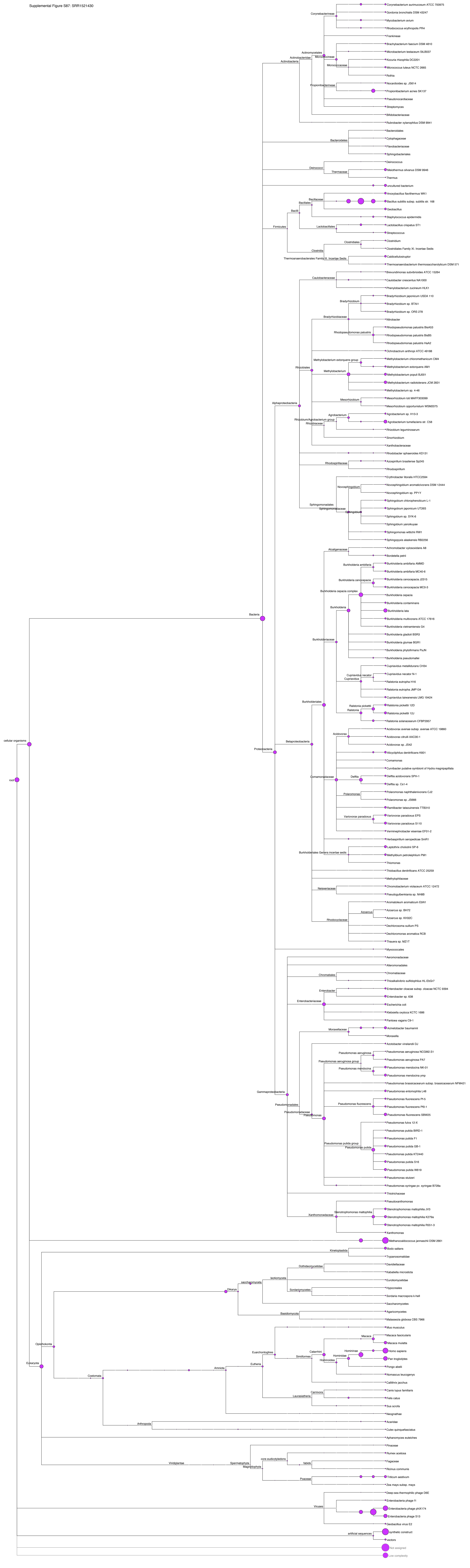

Supplemental Figure S88: SRR1521431

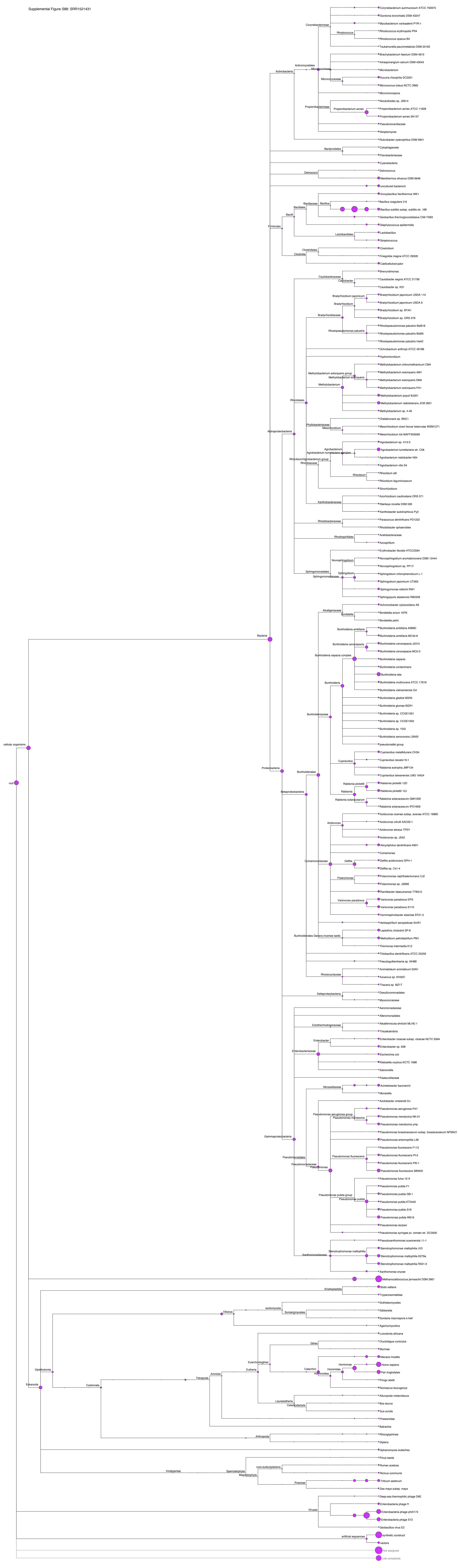

Supplemental Figure 589: SRR1521432

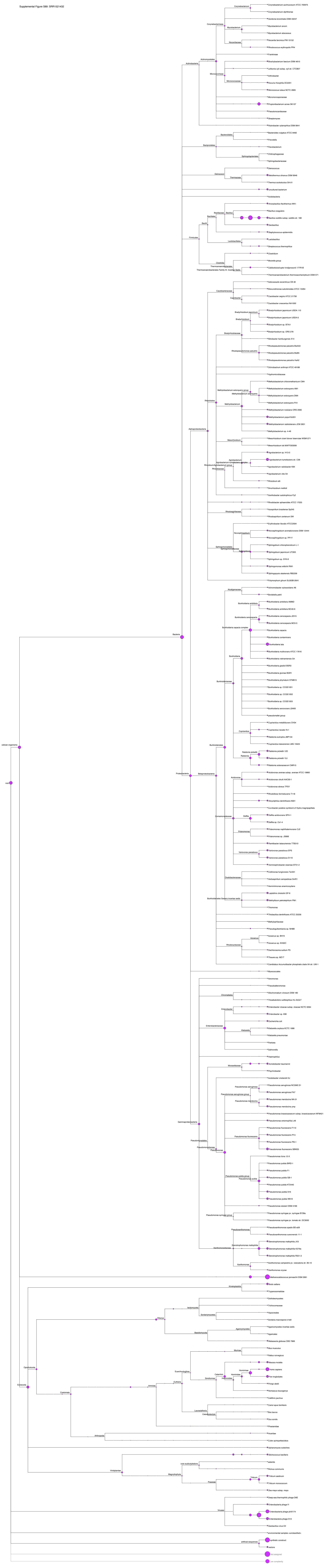

Supplemental Figure S90: SRR1521433

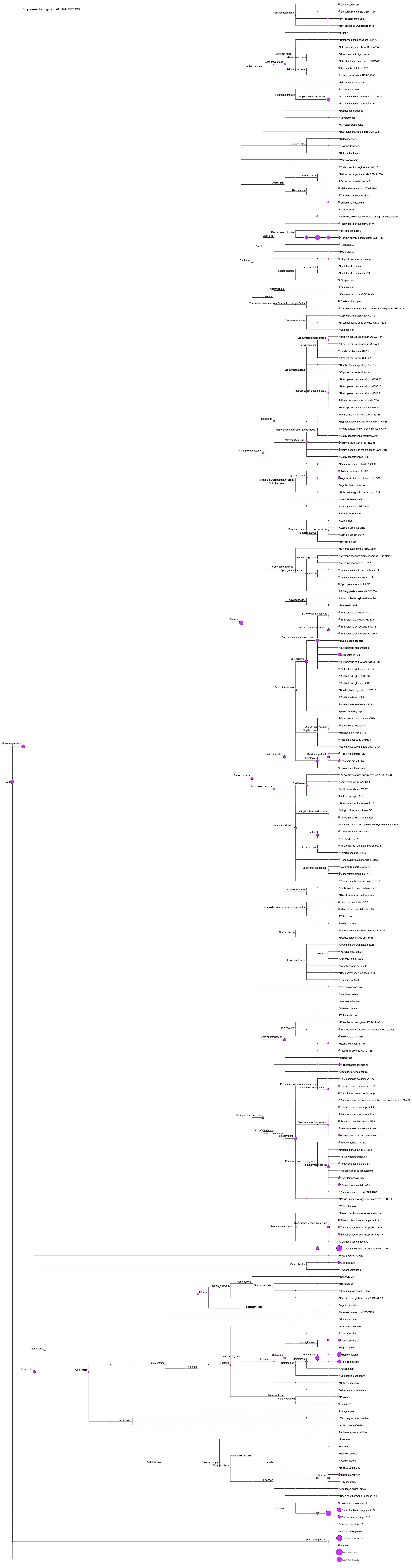

Supplemental Figure S91: SRR1521434

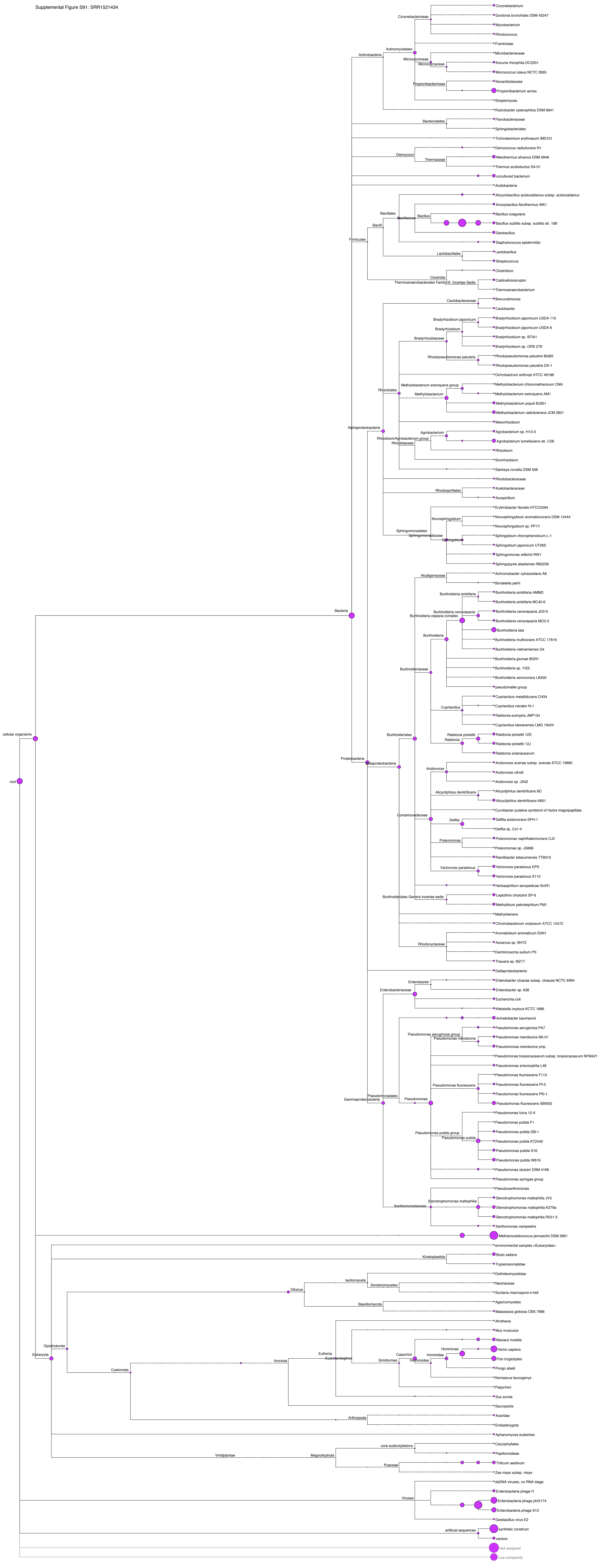

Supplemental Figure S92: SRR1521435

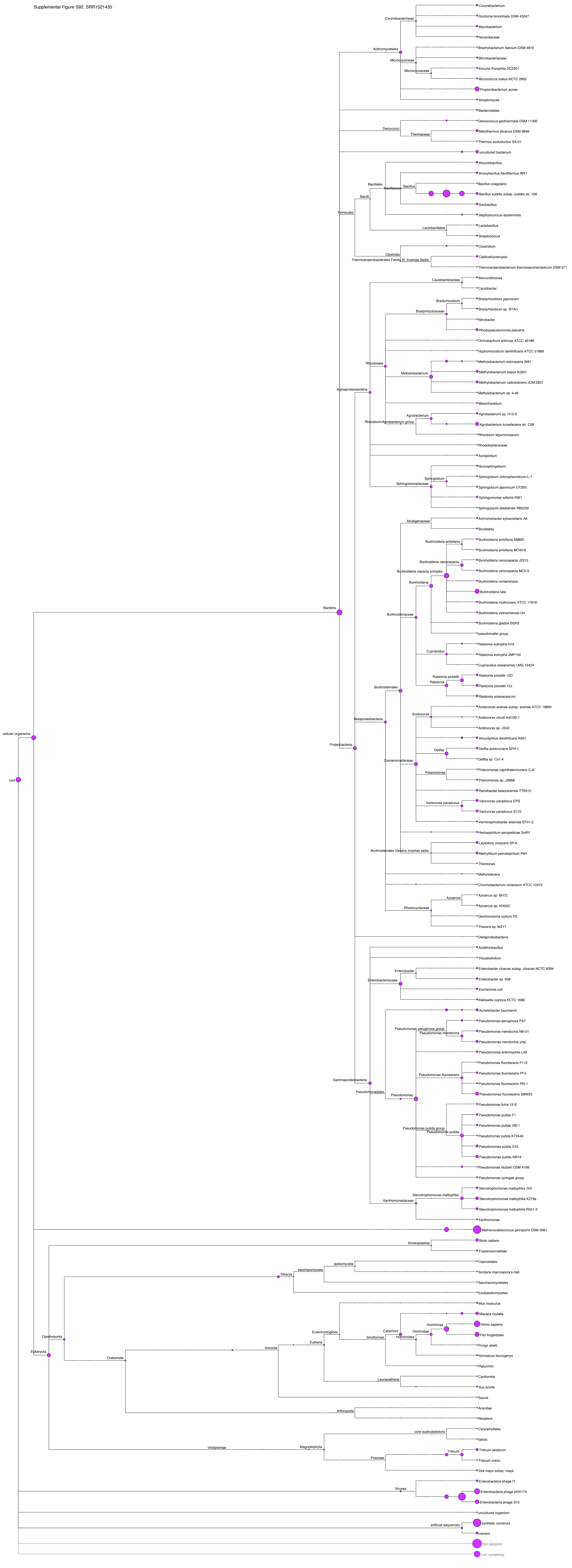

Supplemental Figure S93: SRR1521436

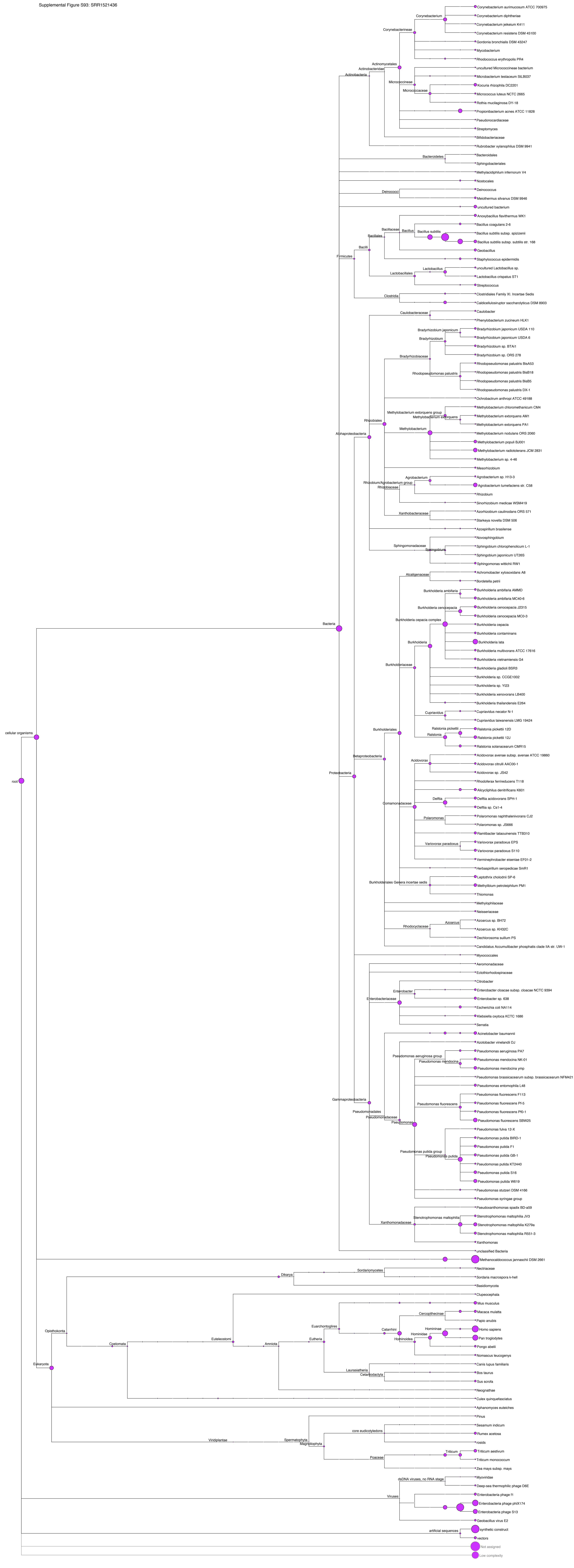

Supplemental Figure S94: SRR1521437

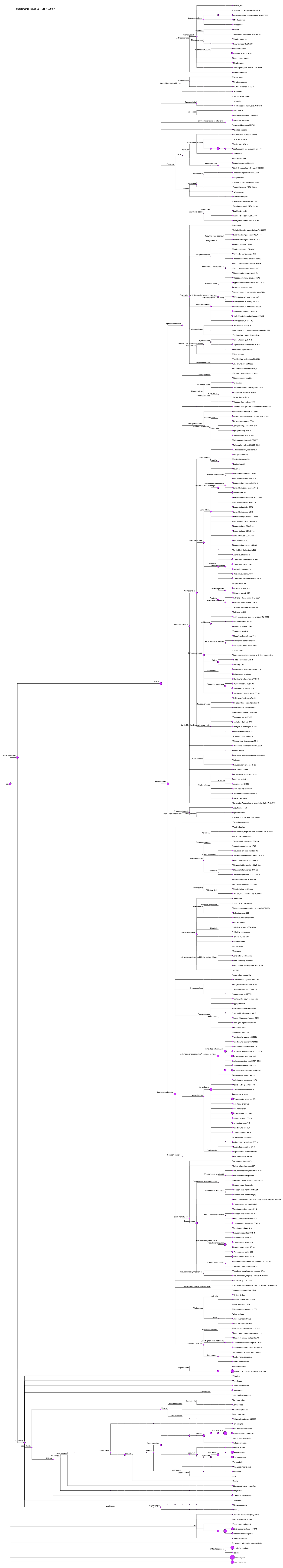

Supplemental Figure S95: SRR1521438

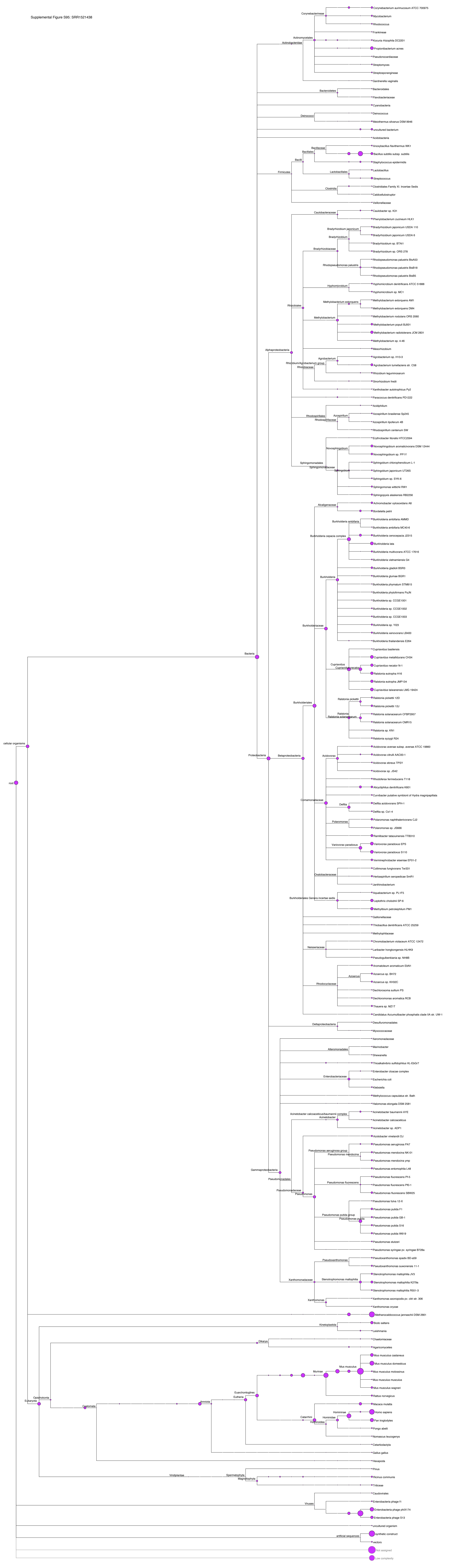

Supplemental Figure S96: SRR1521439

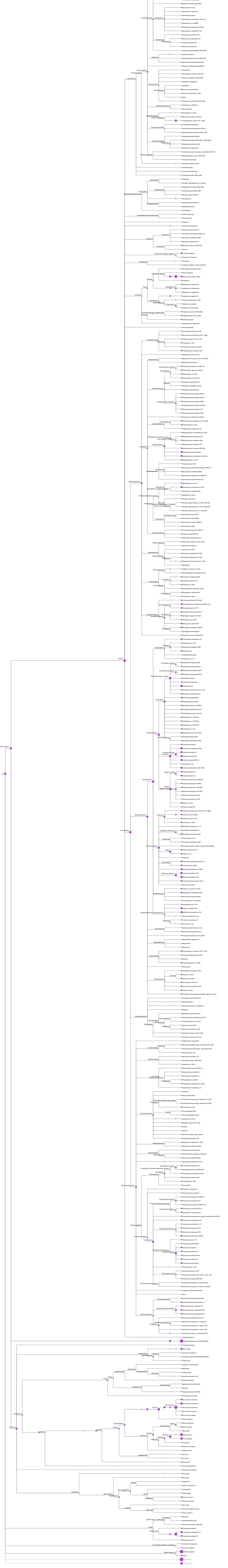

Supplemental Figure S97: SRR1521440

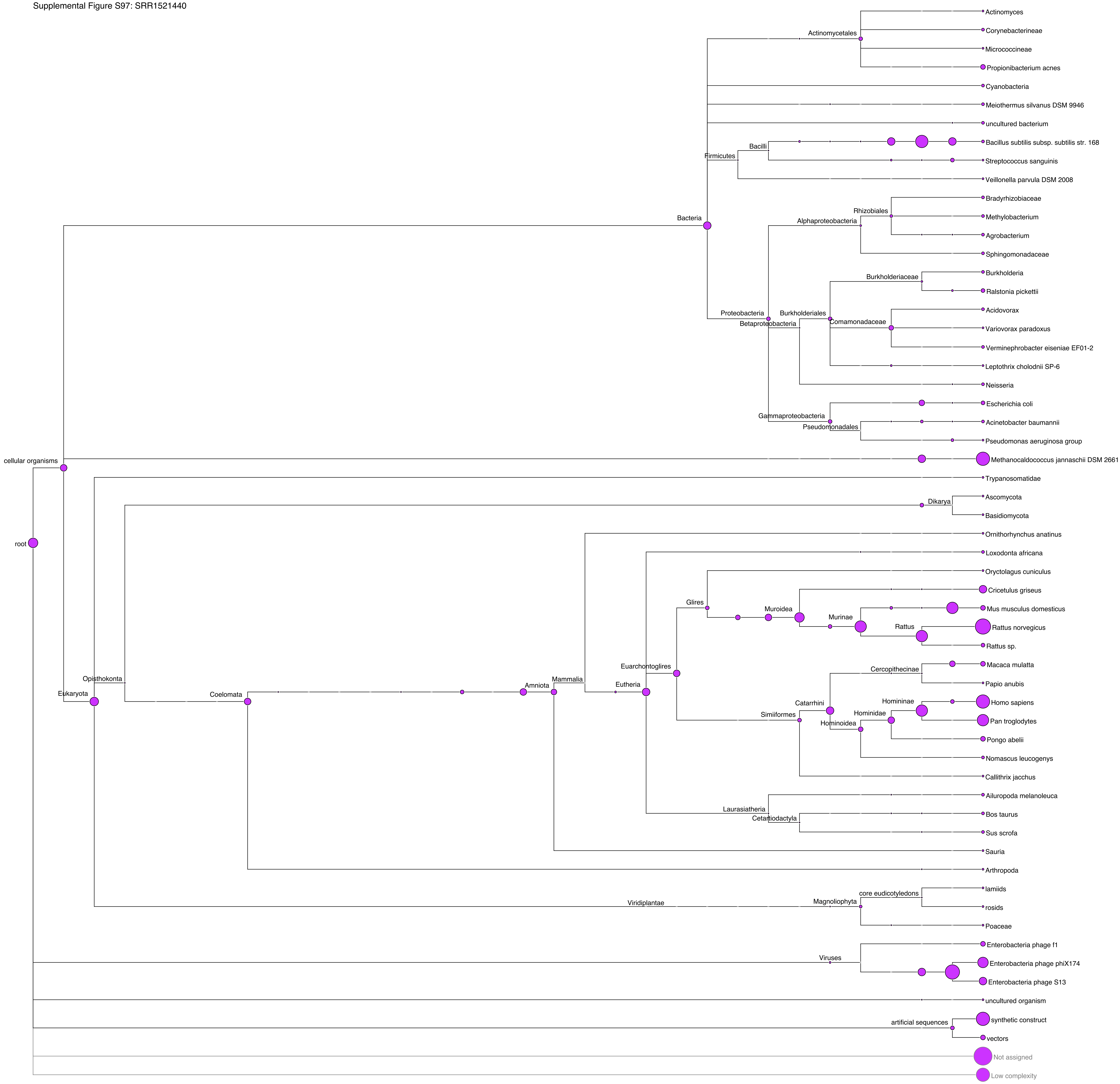

Supplemental Figure S98: SRR1521441

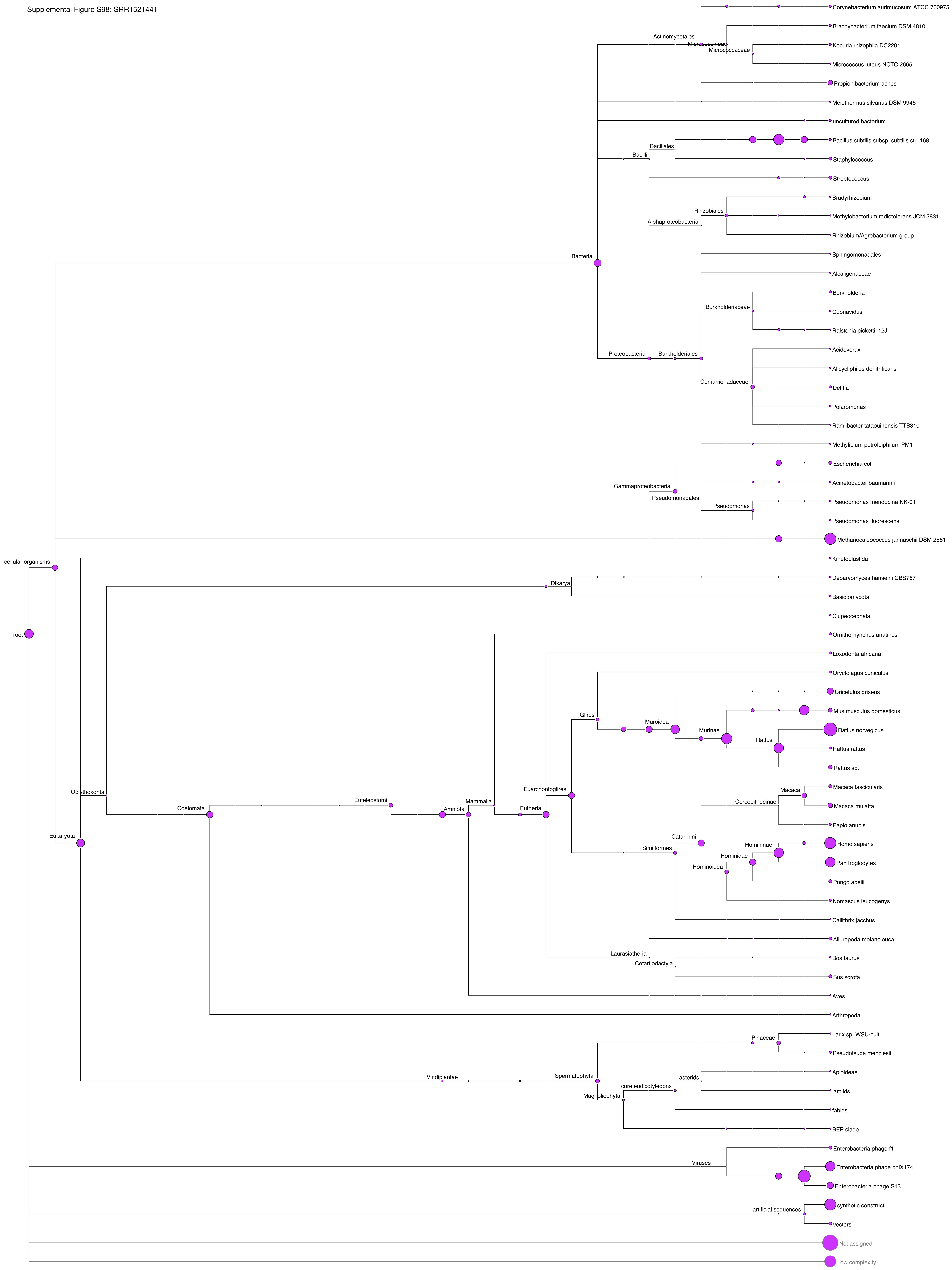

Supplemental Figure S99: SRR1521442

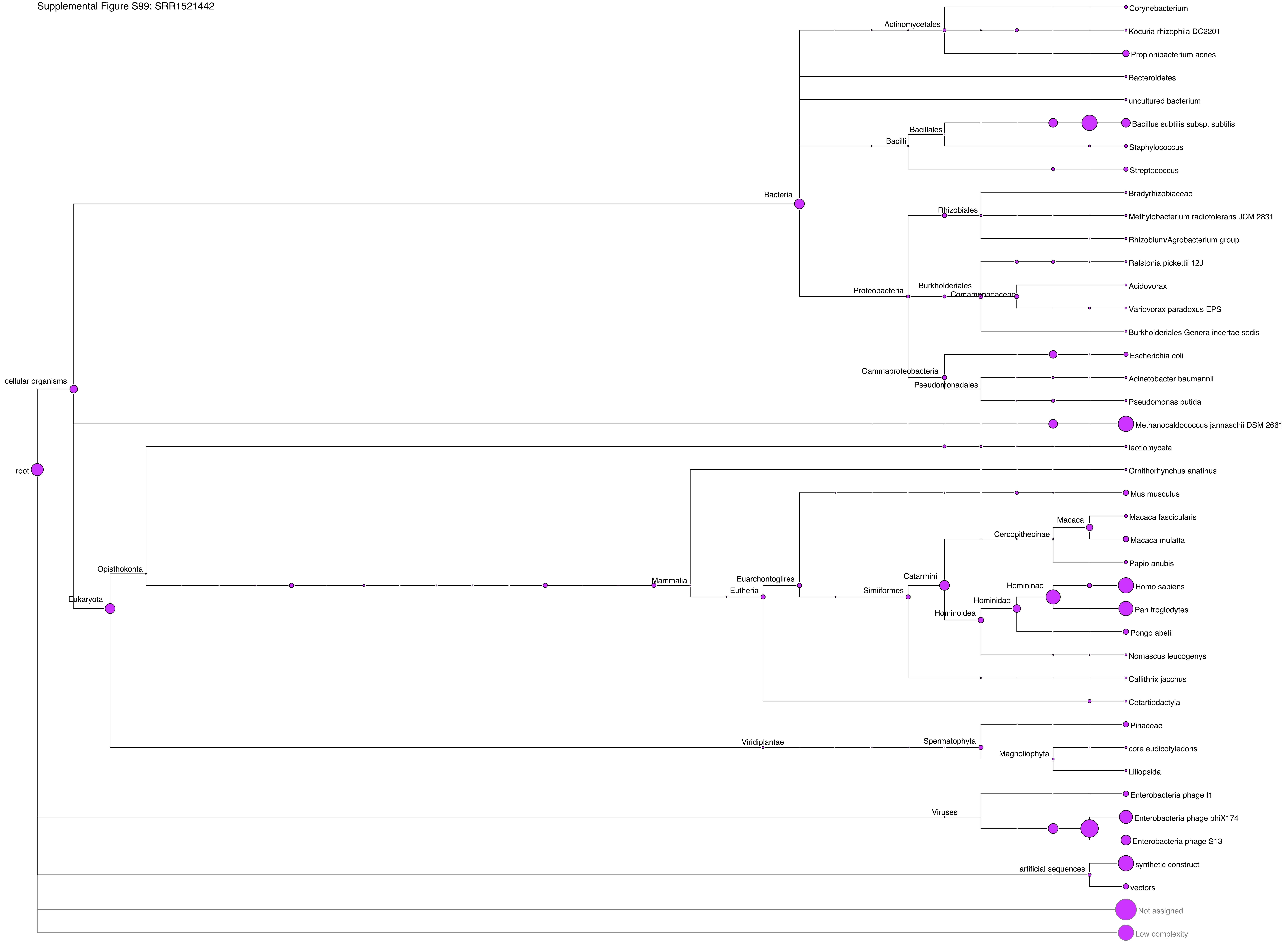

Supplemental Figure S100: SRR1521443

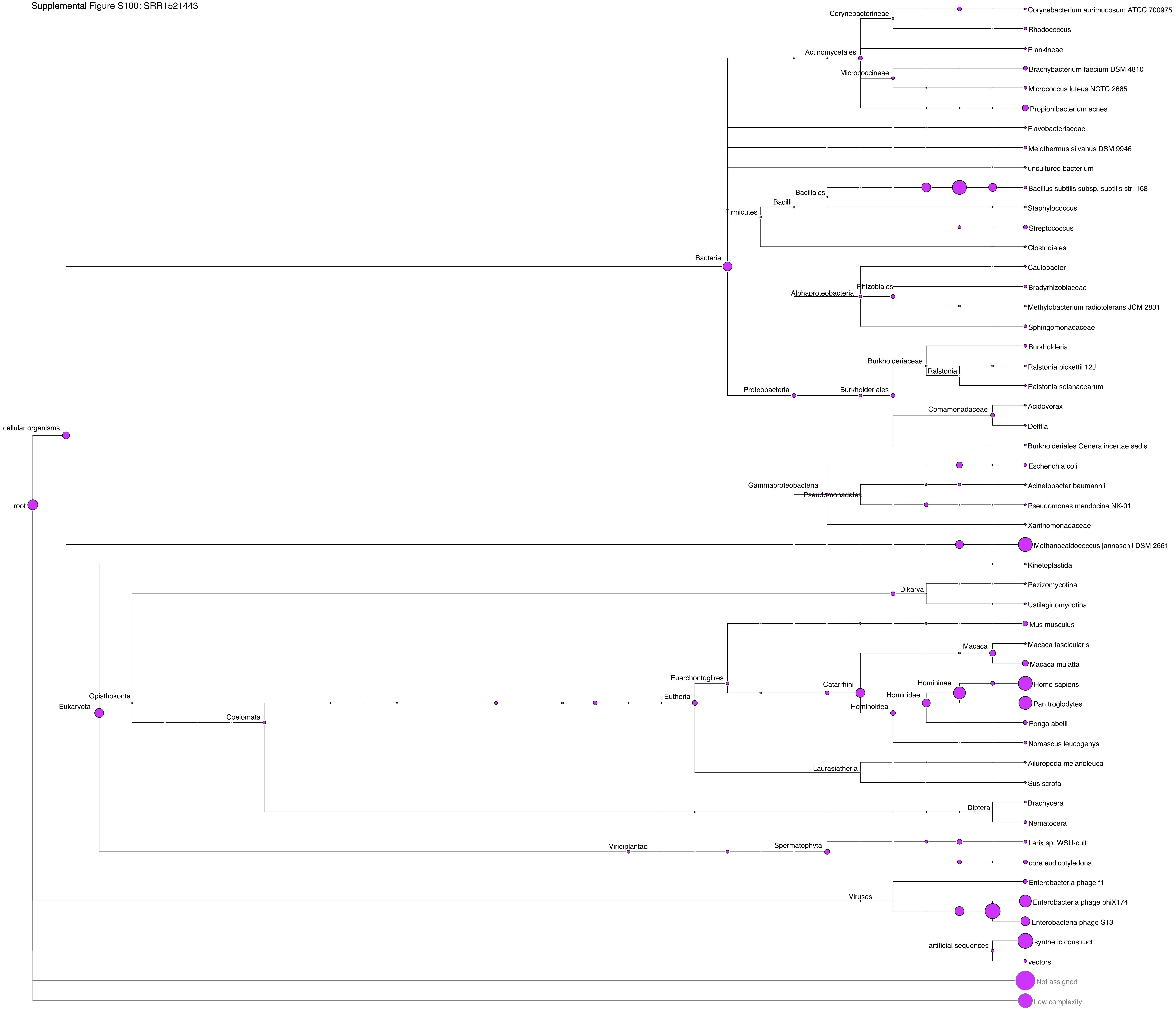

Supplemental Figure S101: SRR1521444

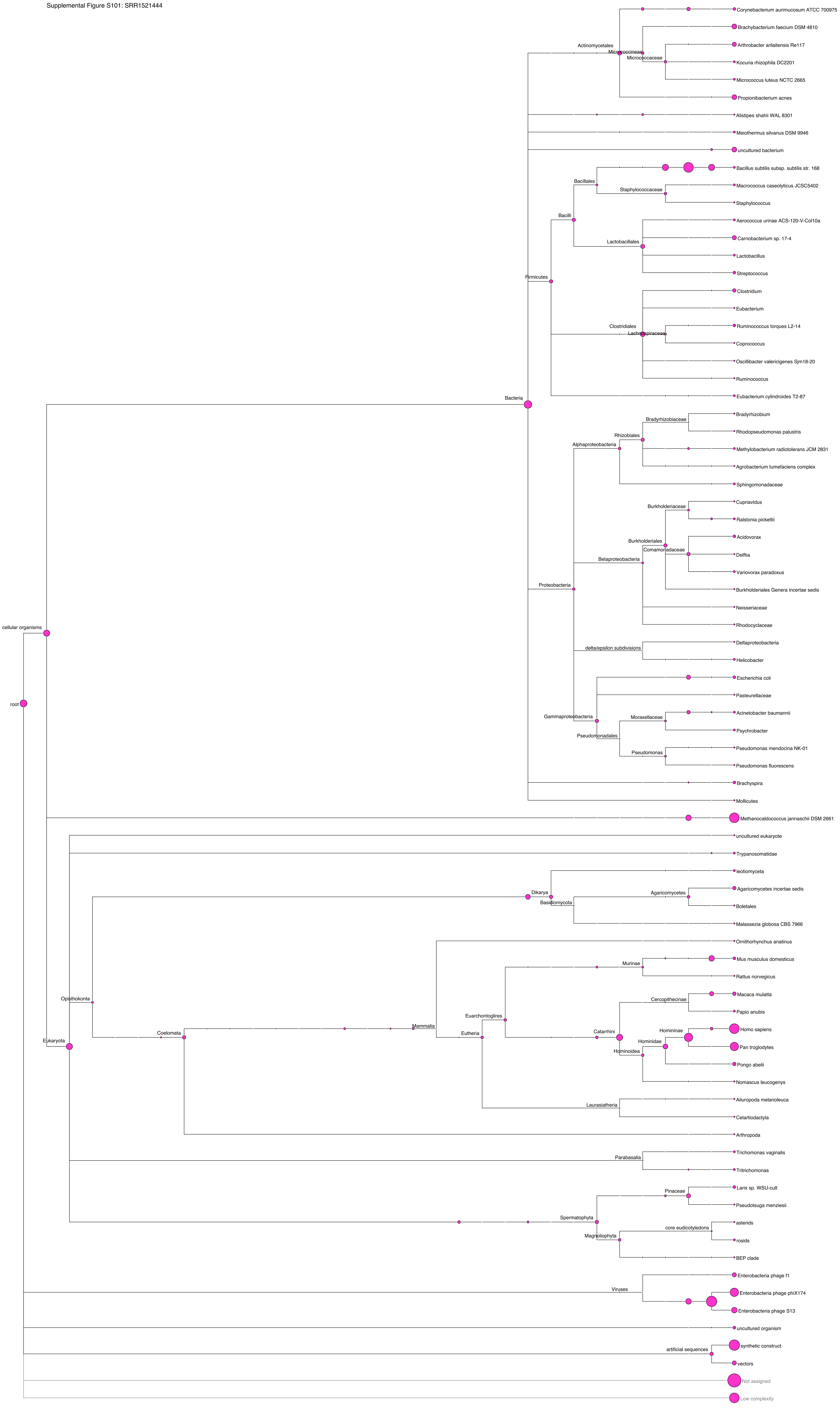

Supplemental Figure S102: SRR1521445

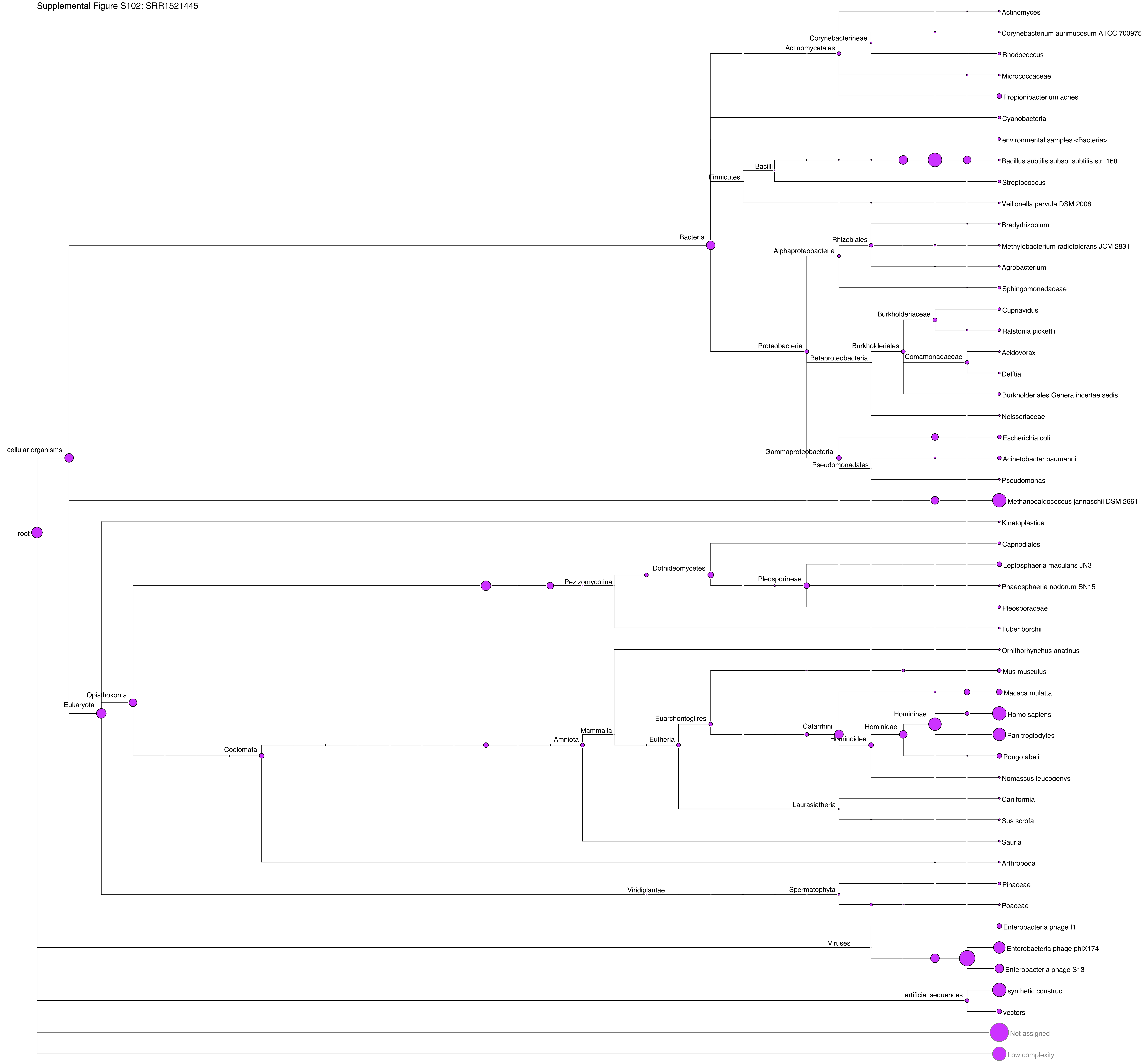

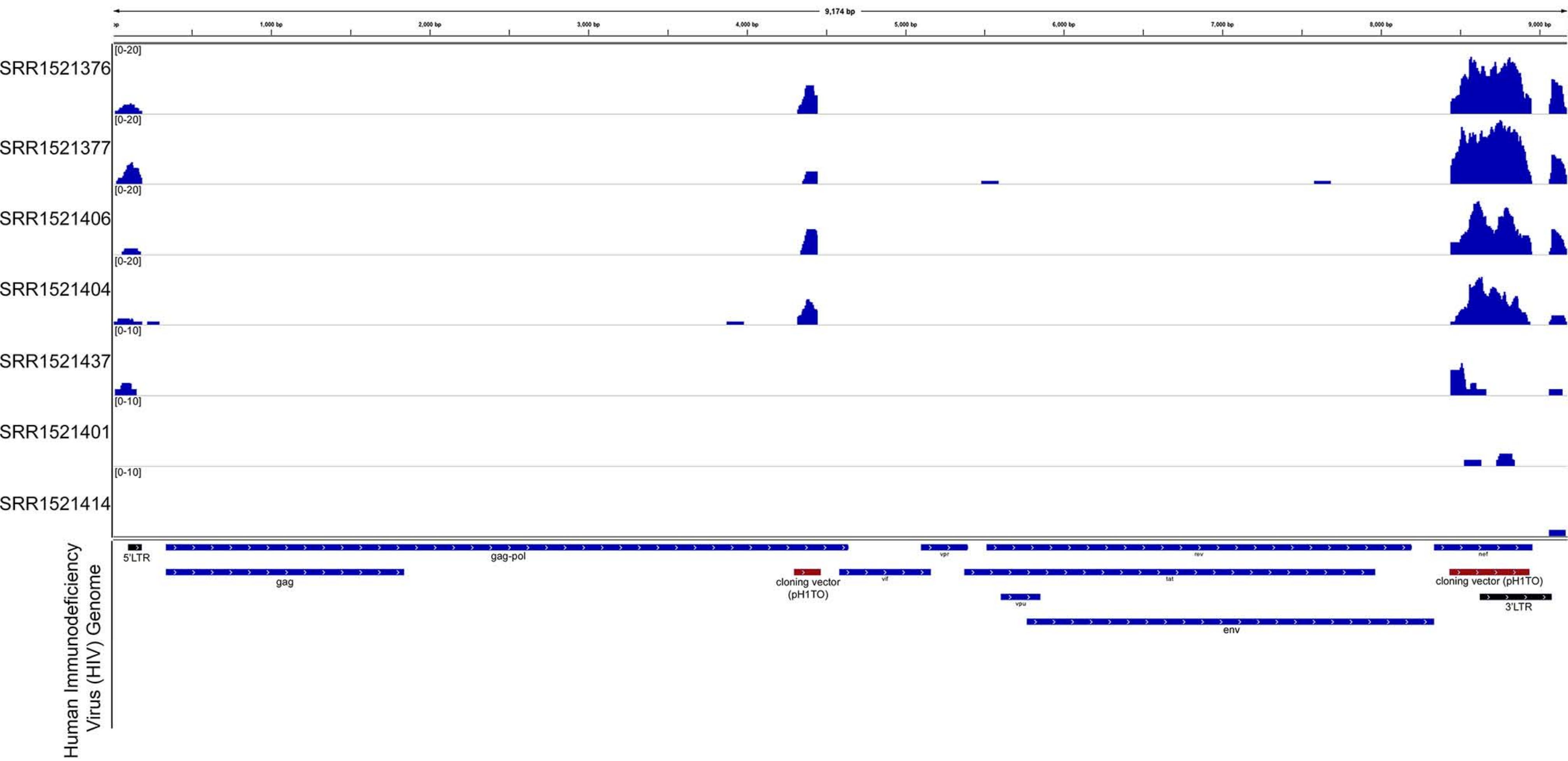
9,174 bp
1,000 bp
2,000 bp
3,000 bp
4,000 bp
5,000 bp
6,000 bp
7,000 bp
8,000 bp
9,000 bp
SRR1521376
[0-20]
SRR1521377
[0-20]
SRR1521406
[0-20]
SRR1521404
[0-20]
SRR1521437
[0-10]
SRR1521401
[0-10]
SRR1521414
[0-10]
Human Immunodeficiency Virus (HIV) Genome
5'LTR
gag-pol
gag
vpr
rev
nef
cloning vector (pH1TO)
vif
tat
cloning vector (pH1TO)
vpu
3'LTR
env

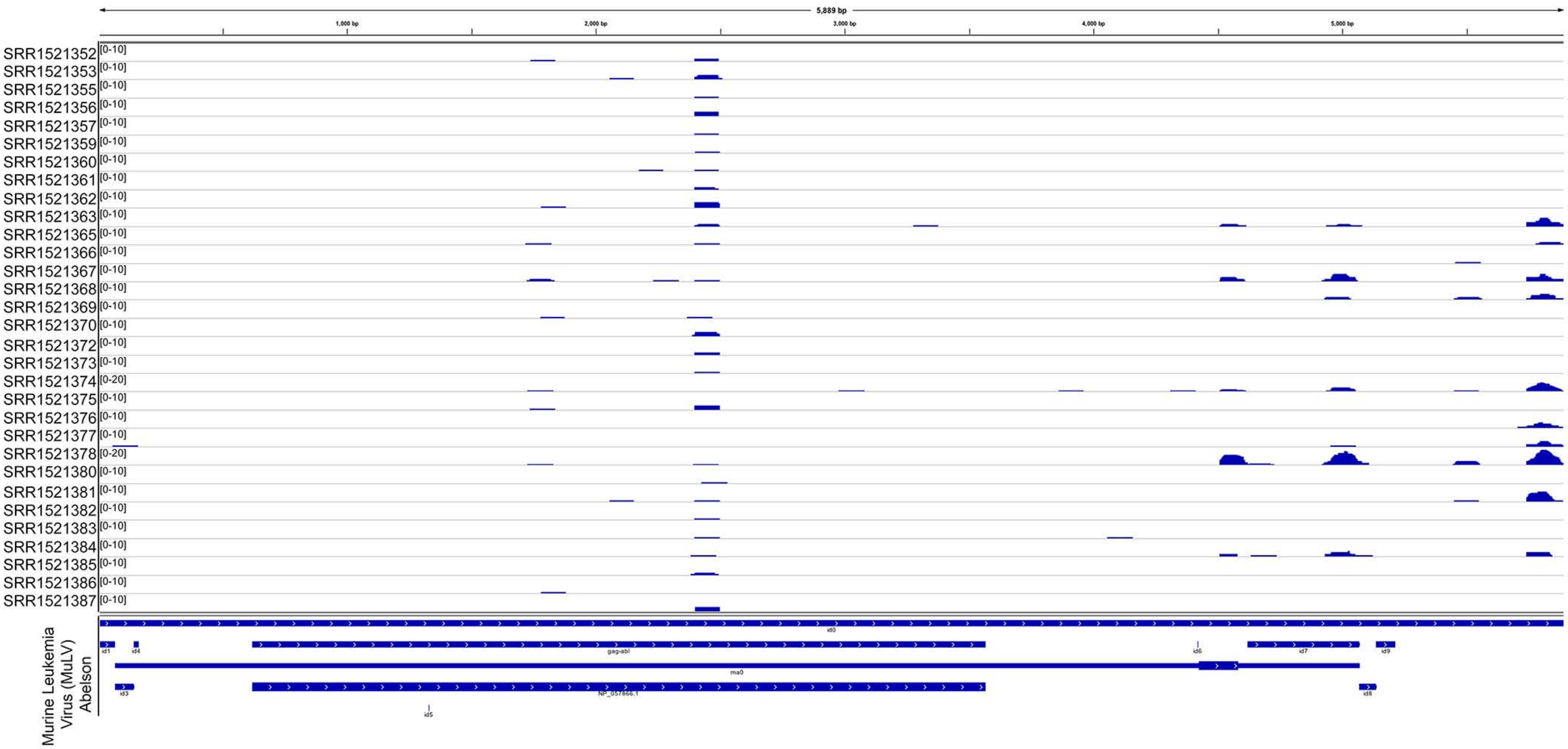

5,889 bp
1,000 bp
2,000 bp
3,000 bp
4,000 bp
5,000 bp
SRR1521352 [0-10]
SRR1521353 [0-10]
SRR1521355 [0-10]
SRR1521356 [0-10]
SRR1521357 [0-10]
SRR1521359 [0-10]
SRR1521360 [0-10]
SRR1521361 [0-10]
SRR1521362 [0-10]
SRR1521363 [0-10]
SRR1521365 [0-10]
SRR1521366 [0-10]
SRR1521367 [0-10]
SRR1521368 [0-10]
SRR1521369 [0-10]
SRR1521370 [0-10]
SRR1521372 [0-10]
SRR1521373 [0-10]
SRR1521374 [0-20]
SRR1521375 [0-10]
SRR1521376 [0-10]
SRR1521377 [0-10]
SRR1521378 [0-20]
SRR1521380 [0-10]
SRR1521381 [0-10]
SRR1521382 [0-10]
SRR1521383 [0-10]
SRR1521384 [0-10]
SRR1521385 [0-10]
SRR1521386 [0-10]
SRR1521387 [0-10]
Murine Leukemia Virus (MuLV) Abelson
id0
id1
id4
gag-abl
id6
id7
id9
ma0
id3
id5
NP_057866.1
id8

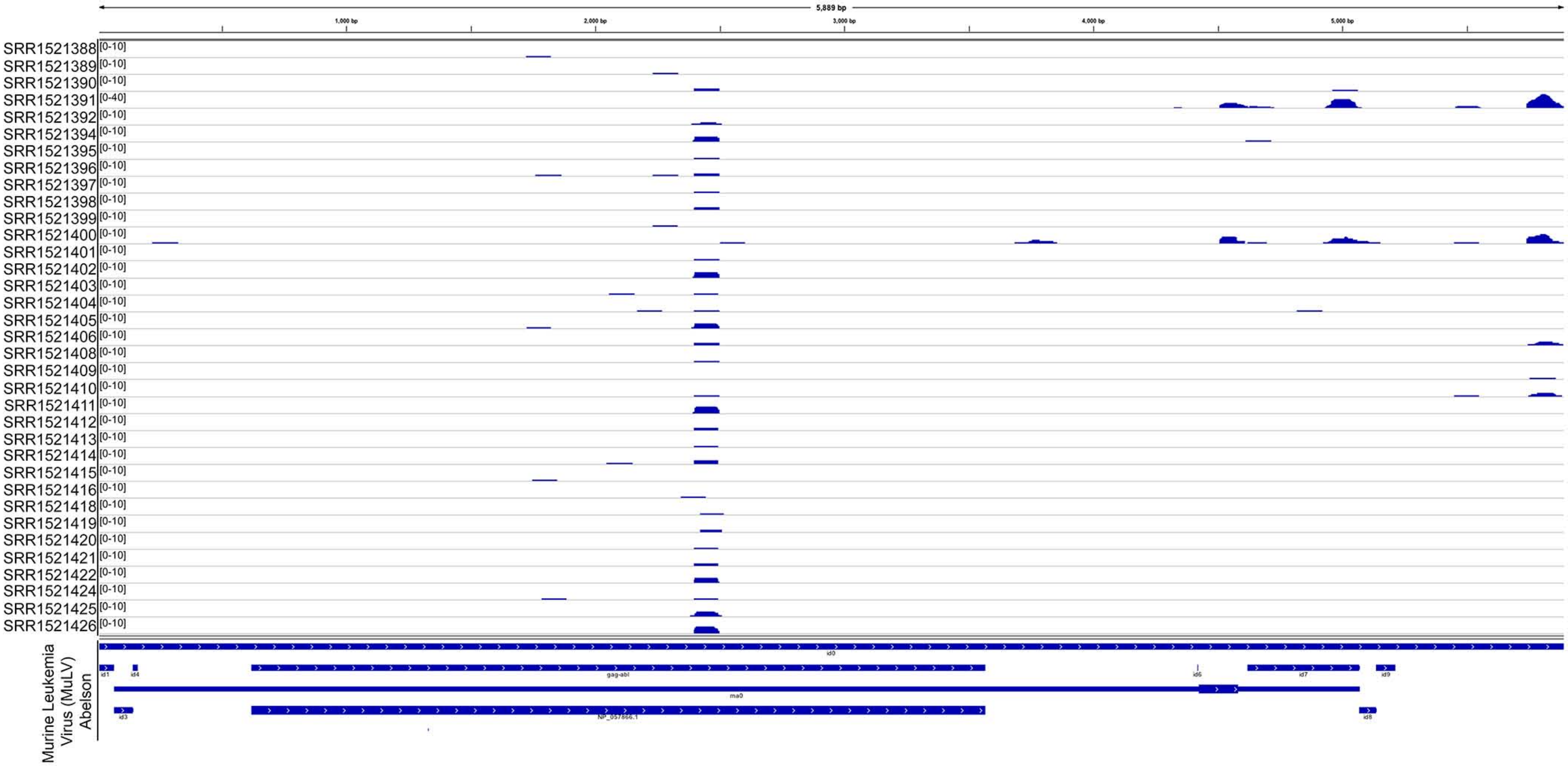

5,889 bp
1,000 bp
2,000 bp
3,000 bp
4,000 bp
5,000 bp
SRR1521388 [0-10]
SRR1521389 [0-10]
SRR1521390 [0-10]
SRR1521391 [0-40]
SRR1521392 [0-10]
SRR1521394 [0-10]
SRR1521395 [0-10]
SRR1521396 [0-10]
SRR1521397 [0-10]
SRR1521398 [0-10]
SRR1521399 [0-10]
SRR1521400 [0-10]
SRR1521401 [0-10]
SRR1521402 [0-10]
SRR1521403 [0-10]
SRR1521404 [0-10]
SRR1521405 [0-10]
SRR1521406 [0-10]
SRR1521408 [0-10]
SRR1521409 [0-10]
SRR1521410 [0-10]
SRR1521411 [0-10]
SRR1521412 [0-10]
SRR1521413 [0-10]
SRR1521414 [0-10]
SRR1521415 [0-10]
SRR1521416 [0-10]
SRR1521418 [0-10]
SRR1521419 [0-10]
SRR1521420 [0-10]
SRR1521421 [0-10]
SRR1521422 [0-10]
SRR1521424 [0-10]
SRR1521425 [0-10]
SRR1521426 [0-10]
Murine Leukemia Virus (MuLV) Abelson
id0
id1
id4
gag-abl
id6
id7
id9
ma0
id3
NP_057866.1
id8

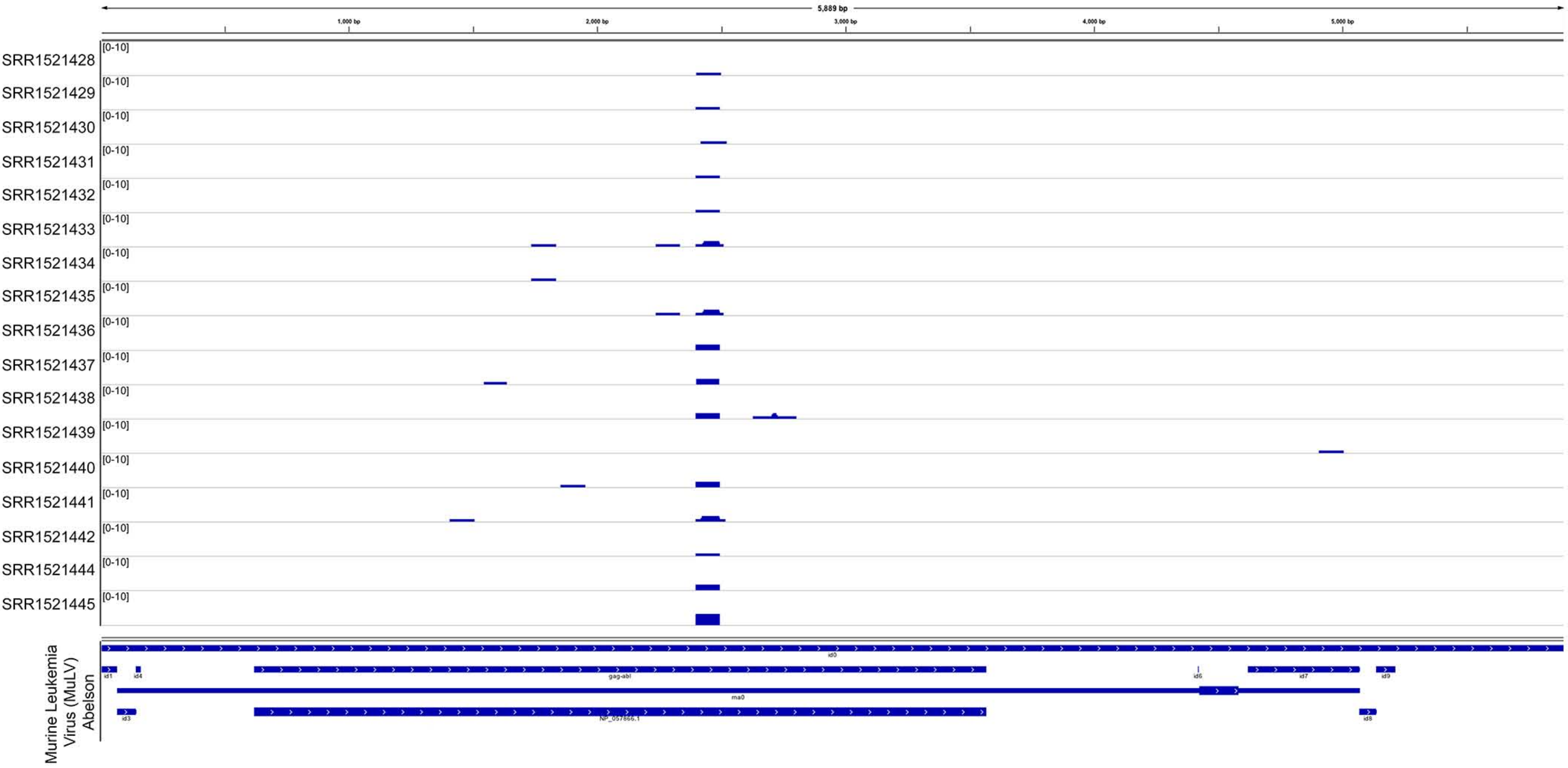

5,889 bp
1,000 bp
2,000 bp
3,000 bp
4,000 bp
5,000 bp
SRR1521428
SRR1521429
SRR1521430
SRR1521431
SRR1521432
SRR1521433
SRR1521434
SRR1521435
SRR1521436
SRR1521437
SRR1521438
SRR1521439
SRR1521440
SRR1521441
SRR1521442
SRR1521444
SRR1521445
[0-10]
Murine Leukemia Virus (MuLV) Abelson
id0
id1
id4
gag-abl
id6
id7
id9
ma0
id3
NP_057866.1
id8

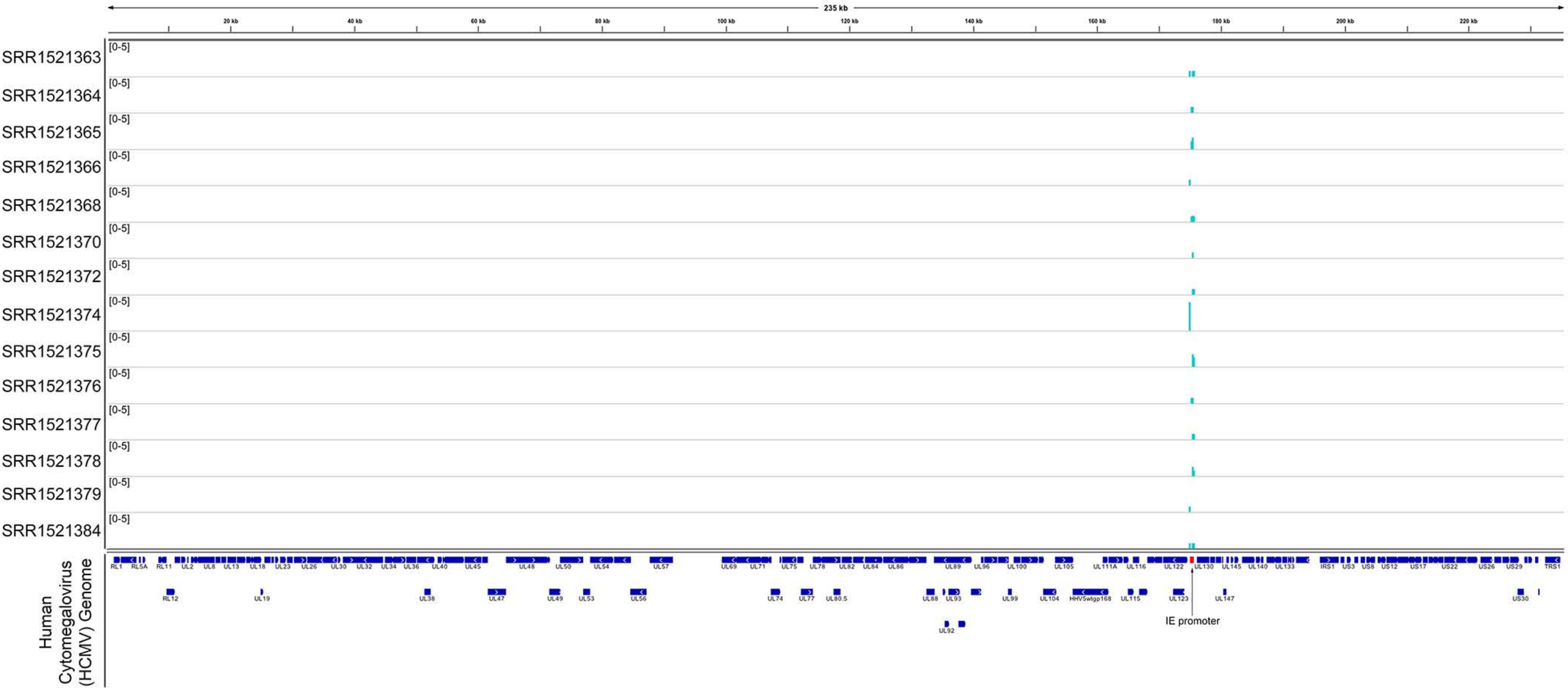
235 kb
20 kb
40 kb
60 kb
80 kb
100 kb
120 kb
140 kb
160 kb
180 kb
200 kb
220 kb
SRR1521363
SRR1521364
SRR1521365
SRR1521366
SRR1521368
SRR1521370
SRR1521372
SRR1521374
SRR1521375
SRR1521376
SRR1521377
SRR1521378
SRR1521379
SRR1521384
[0-5]
Human Cytomegalovirus (HCMV) Genome
RL1 RL5A RL11 UL2 UL8 UL13 UL18 UL23 UL26 UL30 UL32 UL34 UL36 UL40 UL45 UL48 UL50 UL54 UL57 UL69 UL71 UL75 UL78 UL82 UL84 UL86 UL89 UL96 UL100 UL105 UL111A UL116 UL122 UL130 UL145 UL140 UL133 IRS1 US3 US8 US12 US17 US22 US26 US29 TRS1
RL12 UL19 UL38 UL47 UL49 UL53 UL56 UL74 UL77 UL80.5 UL88 UL93 UL99 UL104 HHV5wtgp168 UL115 UL123 UL147 US30
UL92
IE promoter

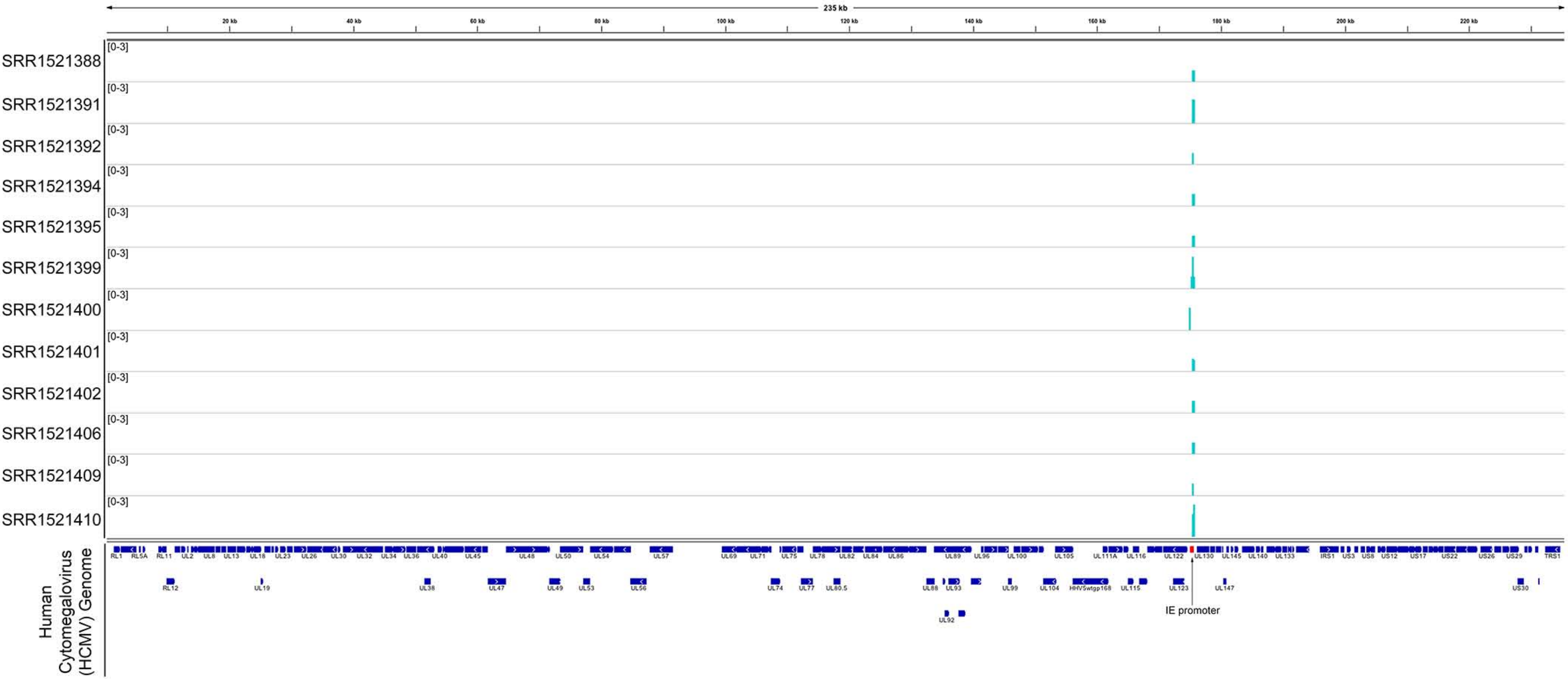
235 kb
20 kb
40 kb
60 kb
80 kb
100 kb
120 kb
140 kb
160 kb
180 kb
200 kb
220 kb
SRR1521388
SRR1521391
SRR1521392
SRR1521394
SRR1521395
SRR1521399
SRR1521400
SRR1521401
SRR1521402
SRR1521406
SRR1521409
SRR1521410
[0-3]
Human Cytomegalovirus (HCMV) Genome
IE promoter

Supplemental Figure S109: SRR608893

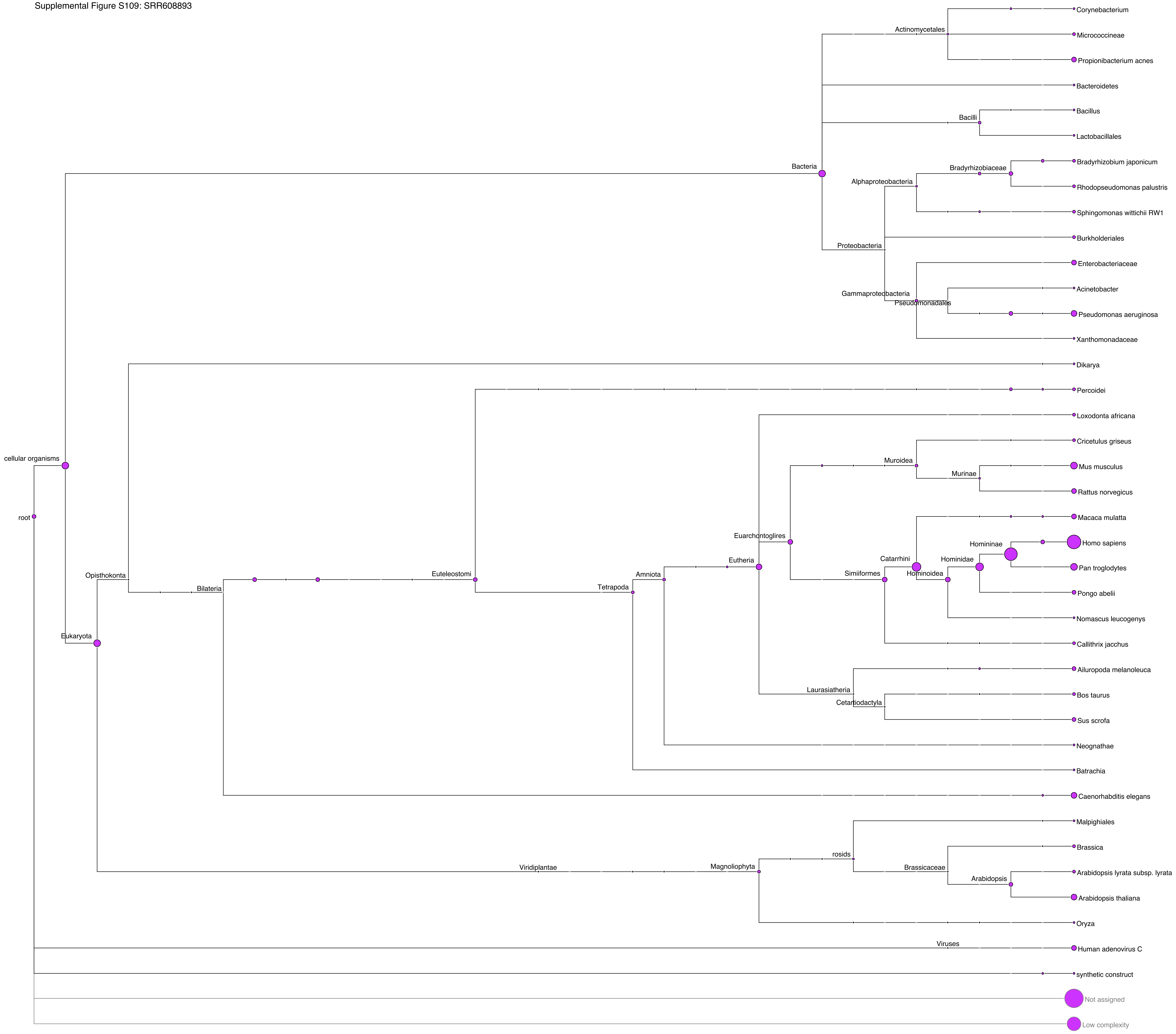

Supplemental Figure S110: SRR608894

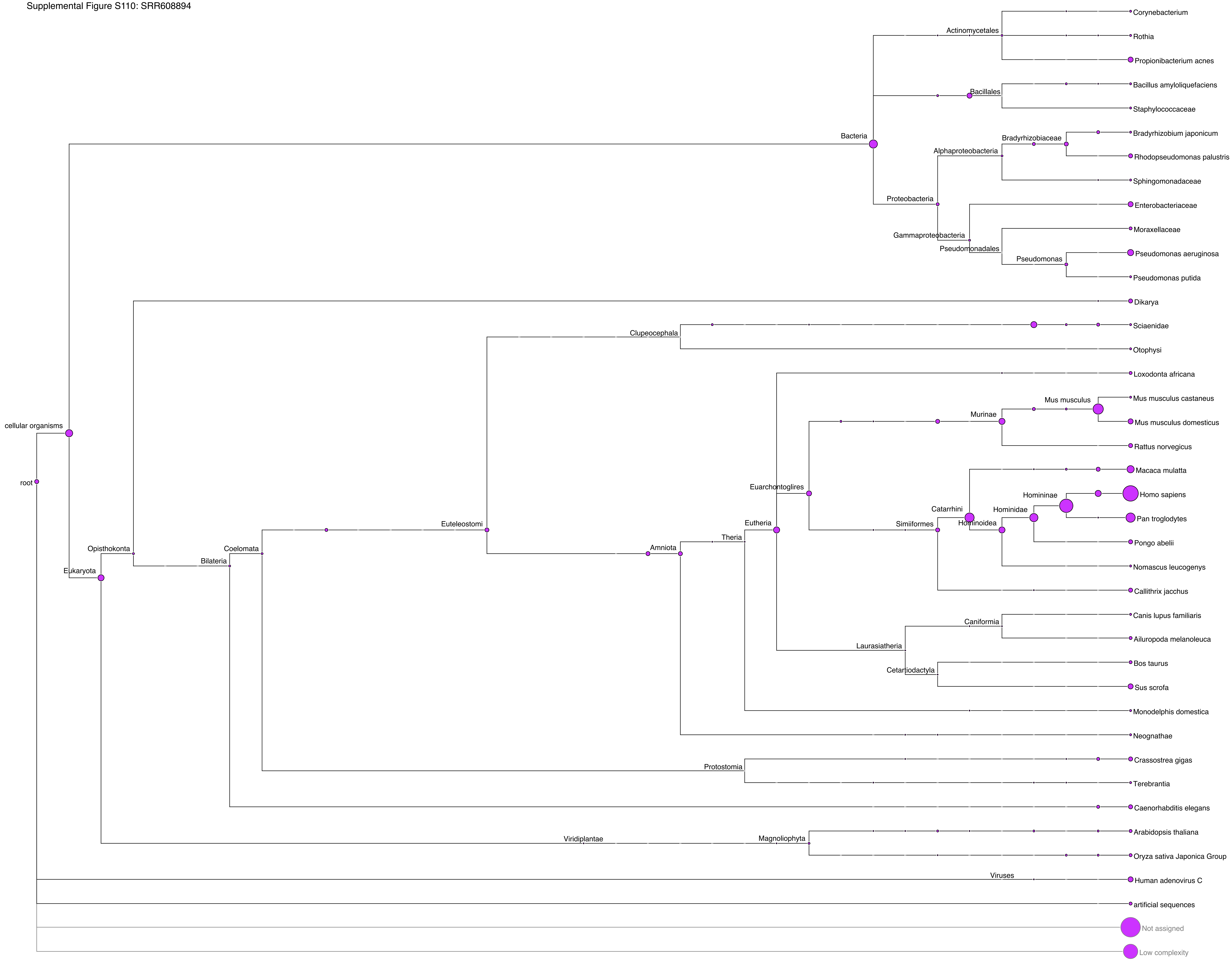

Supplemental Figure S111: SRR608895

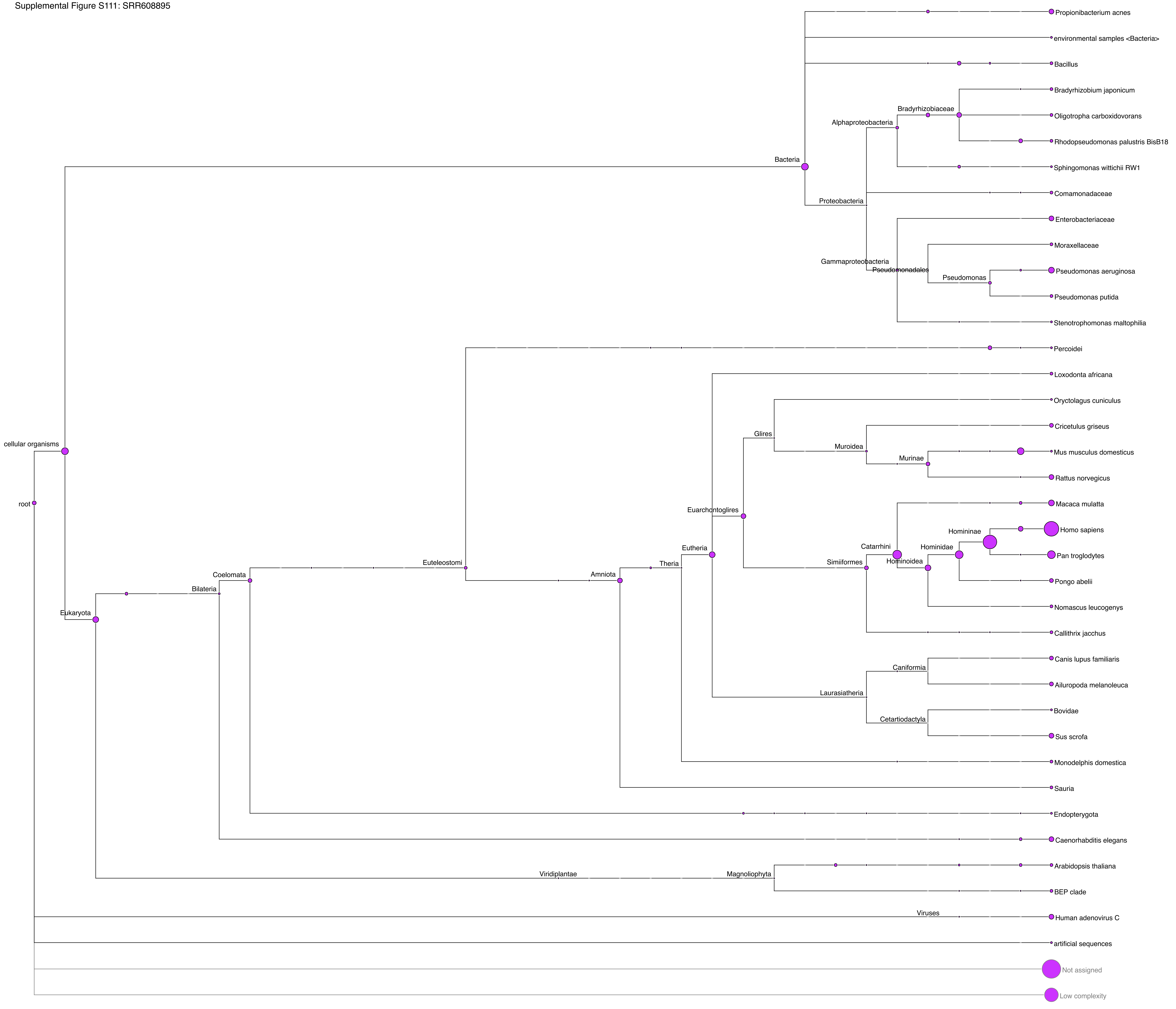

Supplemental Figure S112: SRR608896

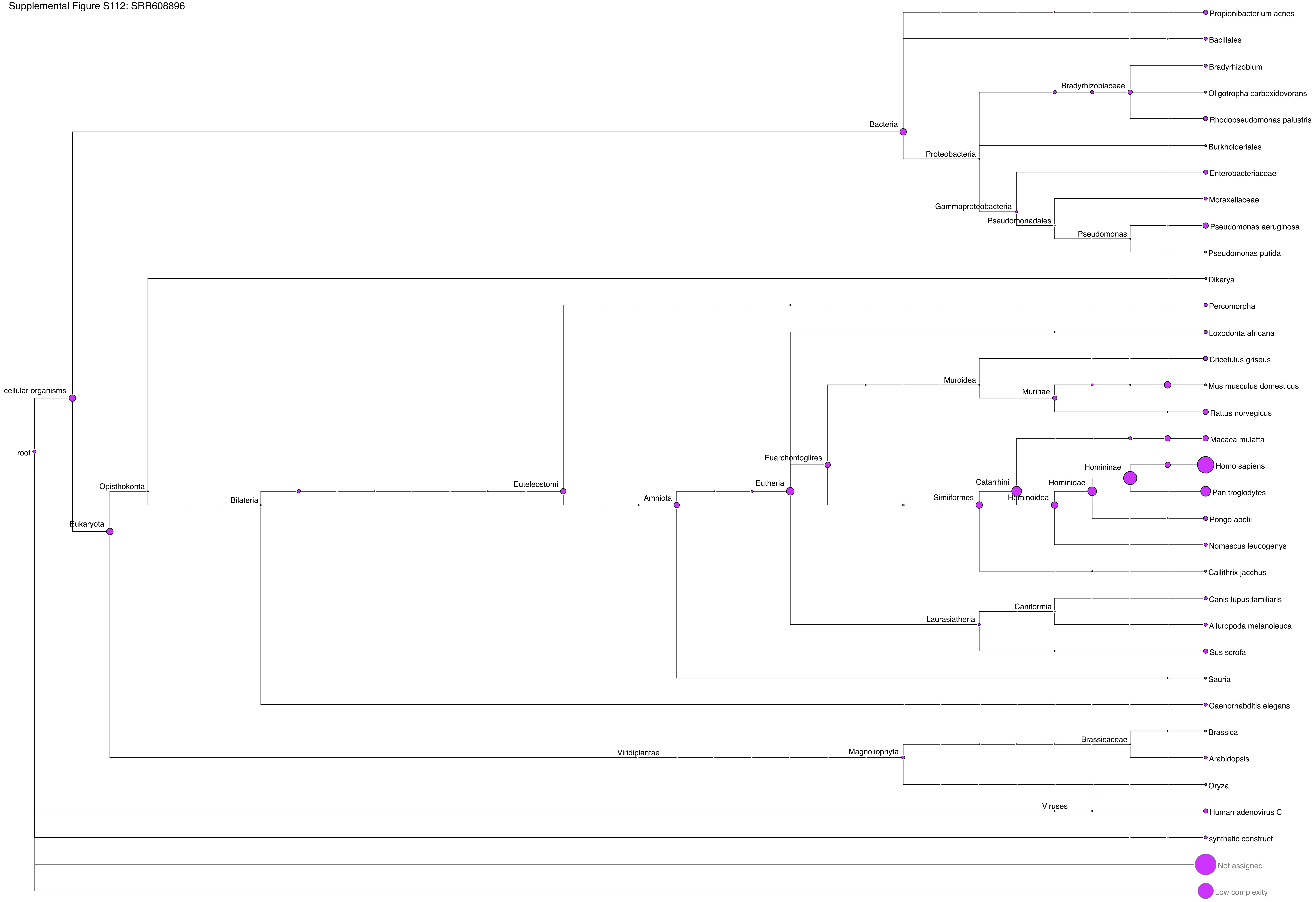

Supplemental Figure S113: SRR608897

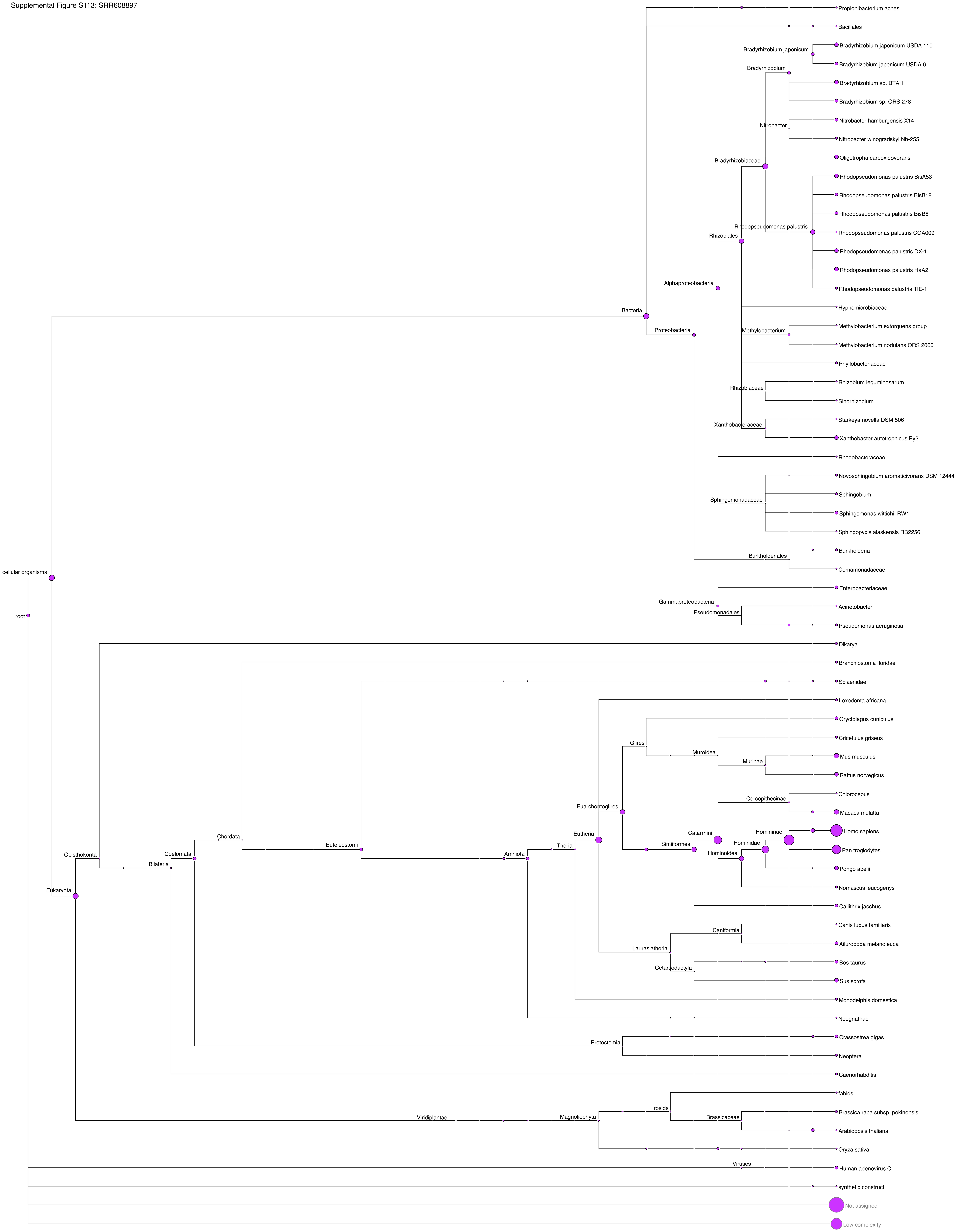

Supplemental Figure S114: SRR608898

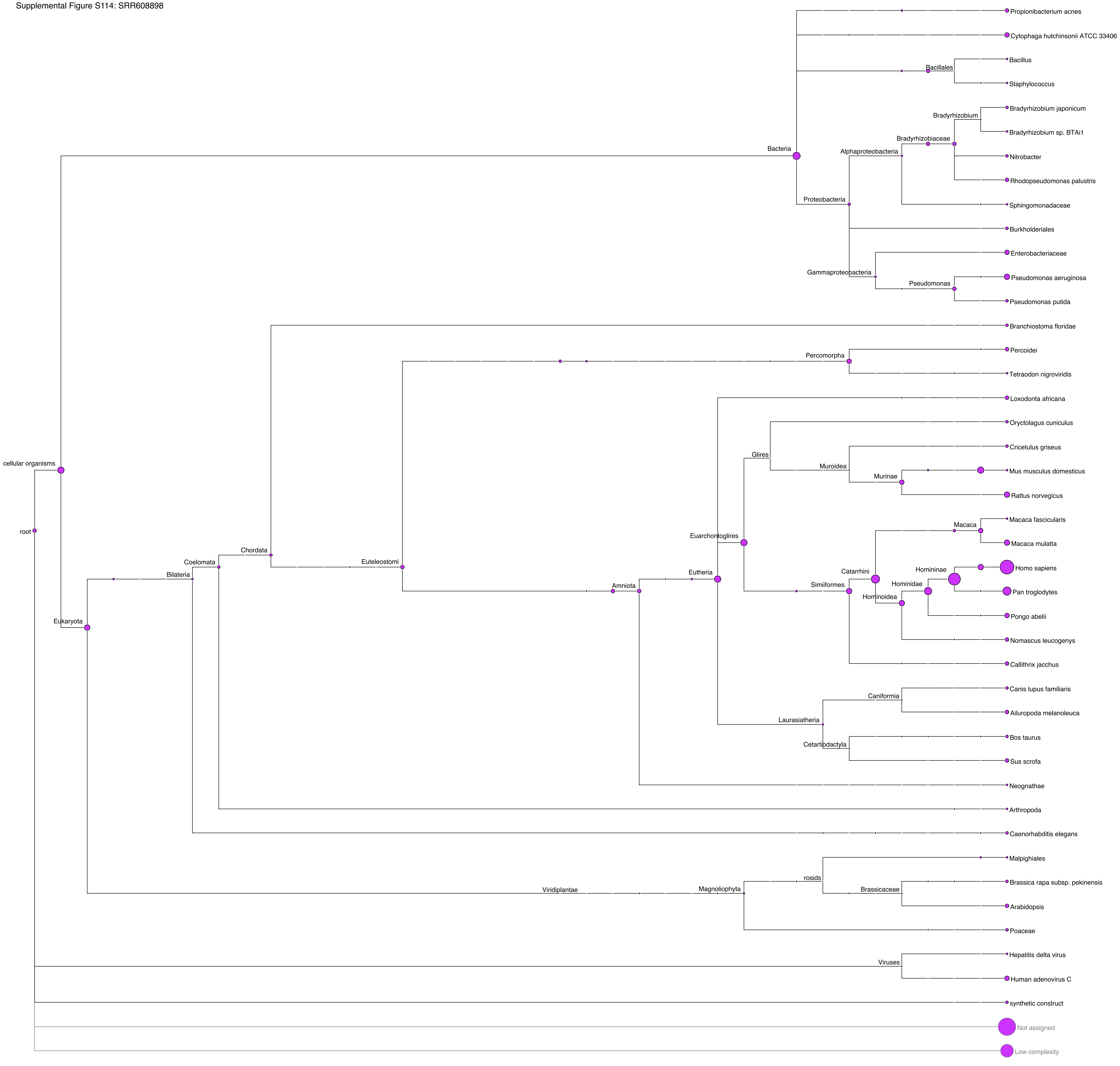

Supplemental Figure S115: SRR608899

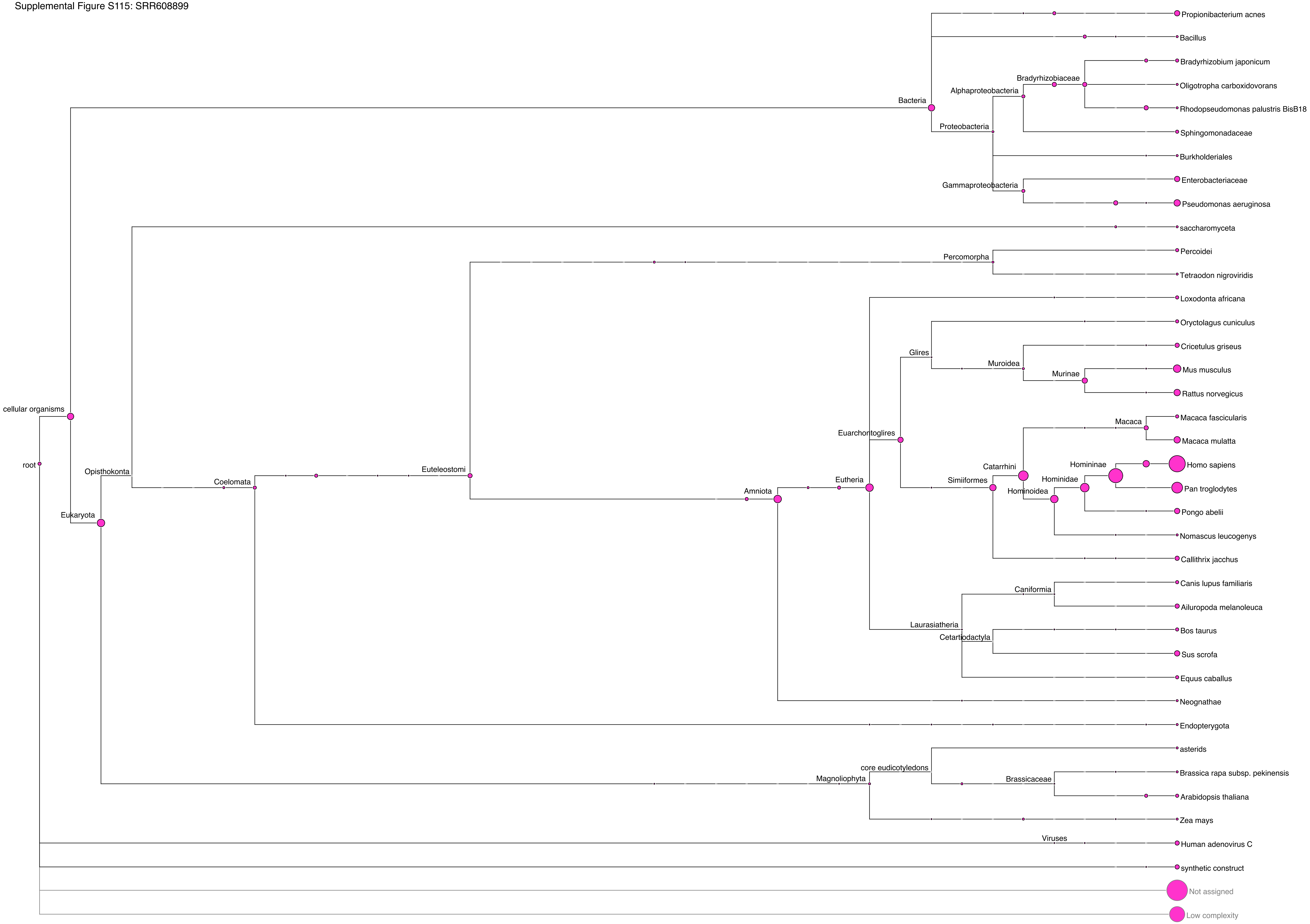

Supplemental Figure S116: SRR608900

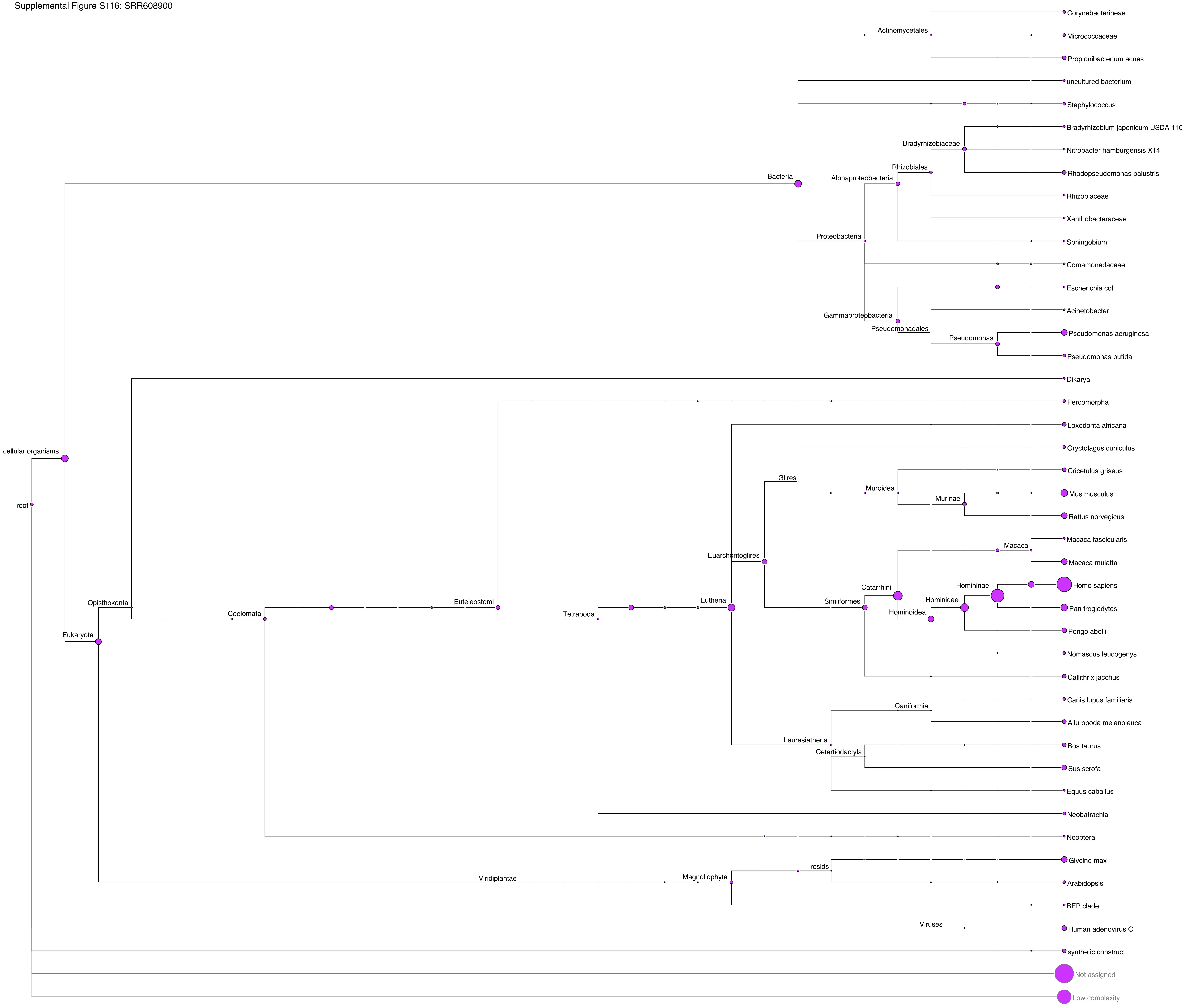

Supplemental Figure S117: SRR608901

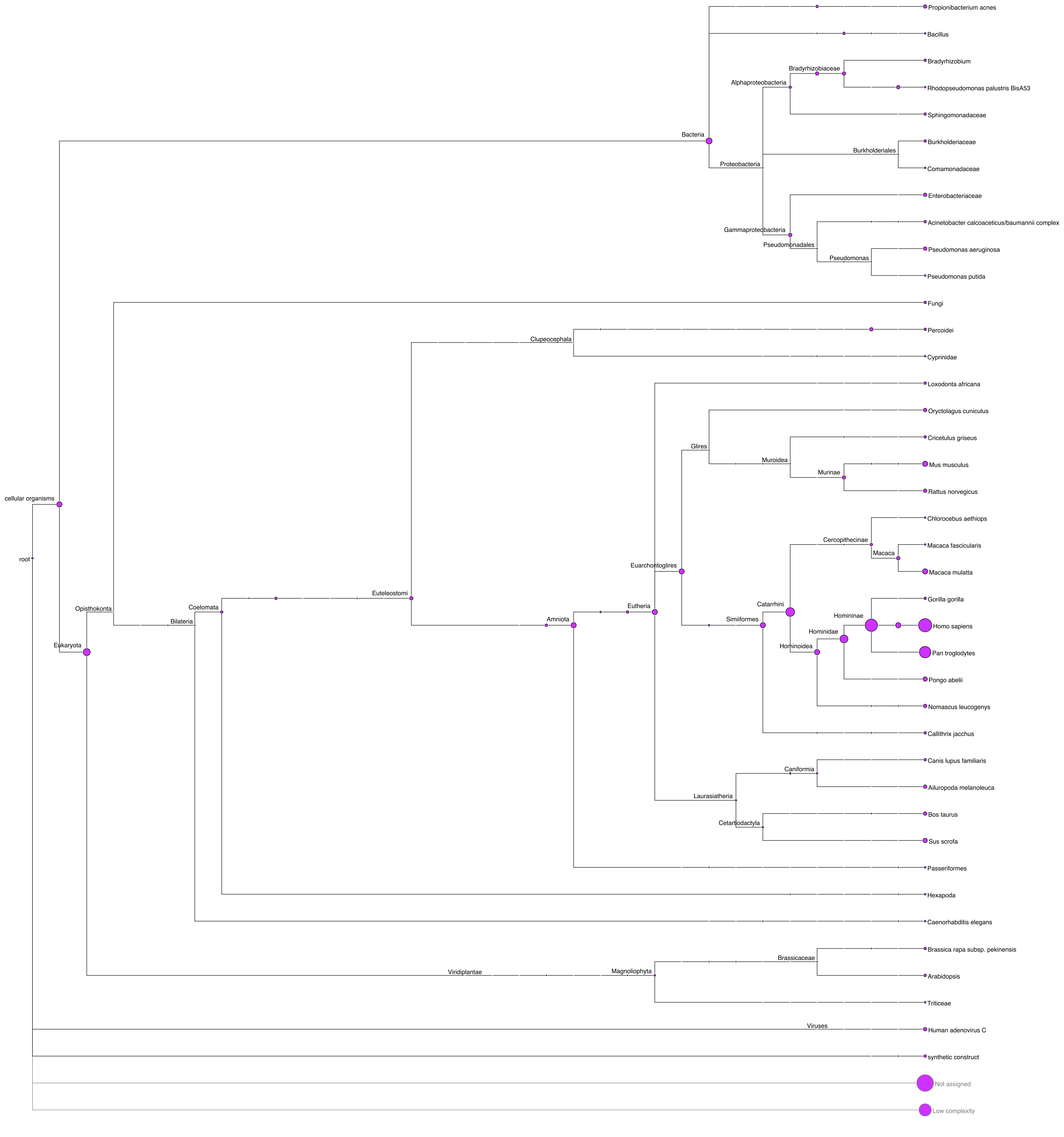

Supplemental Figure S118: SRR942849

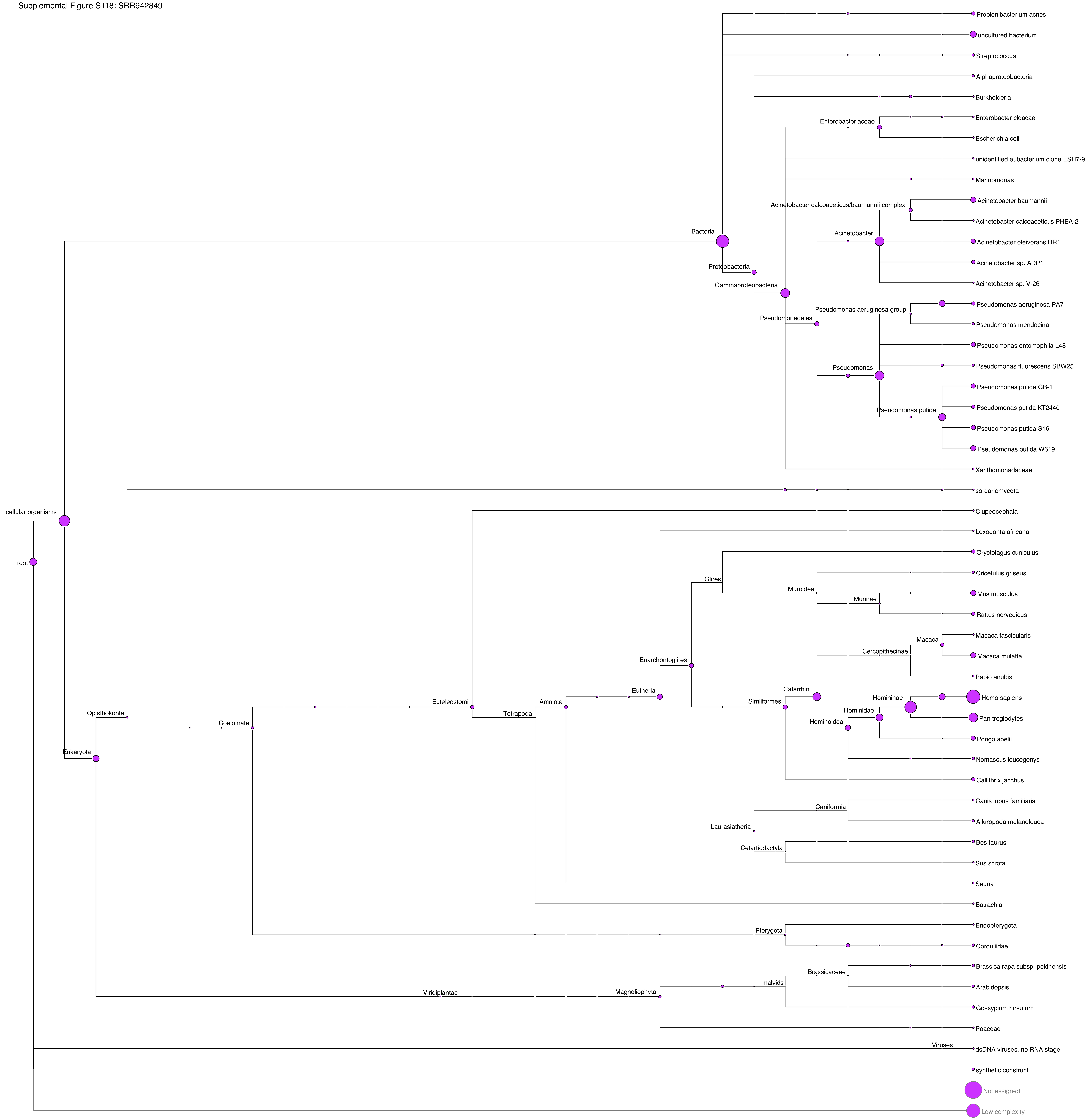

Supplemental Figure S119: SRR942850

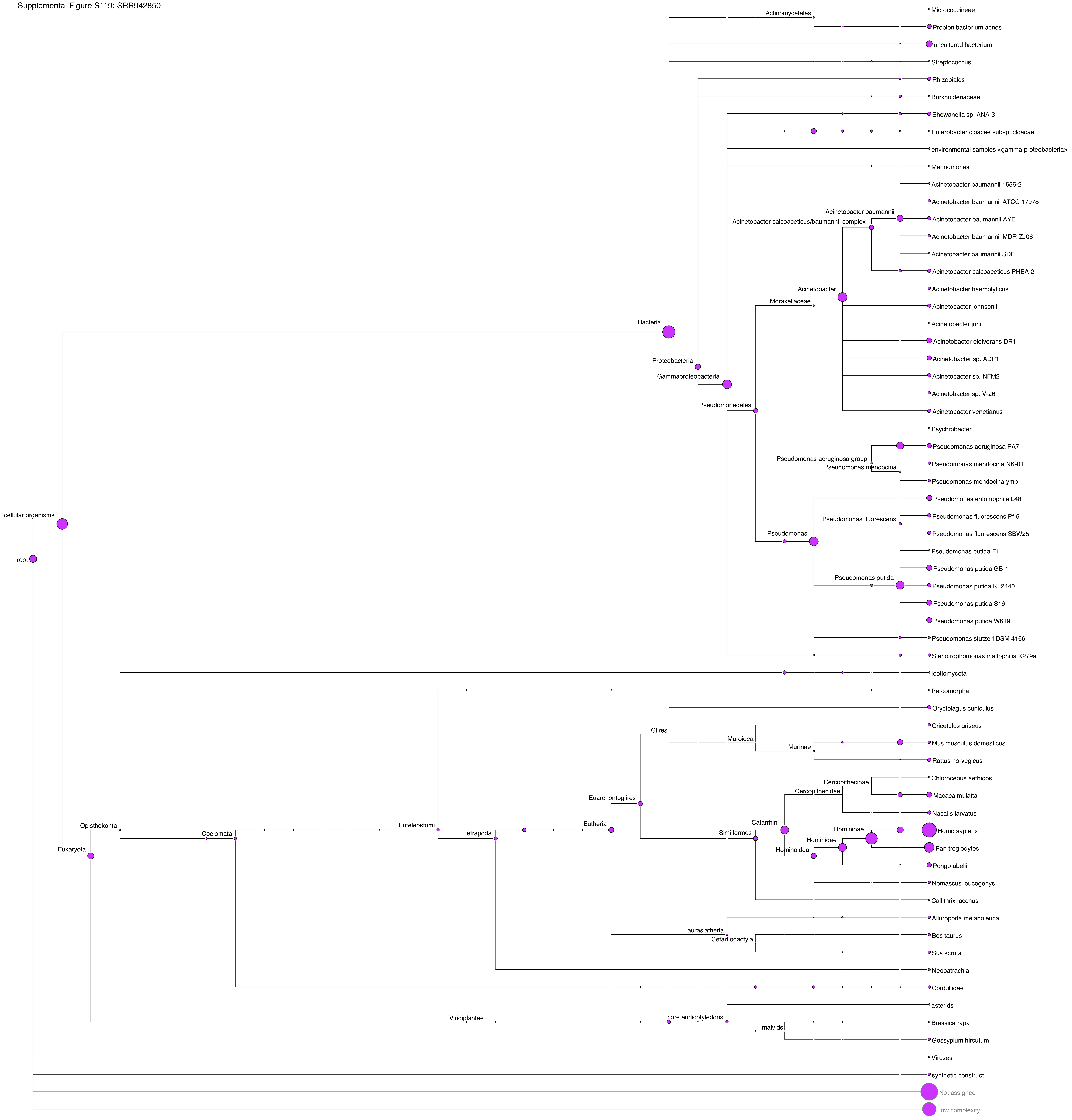

Supplemental Figure S120: SRR942851

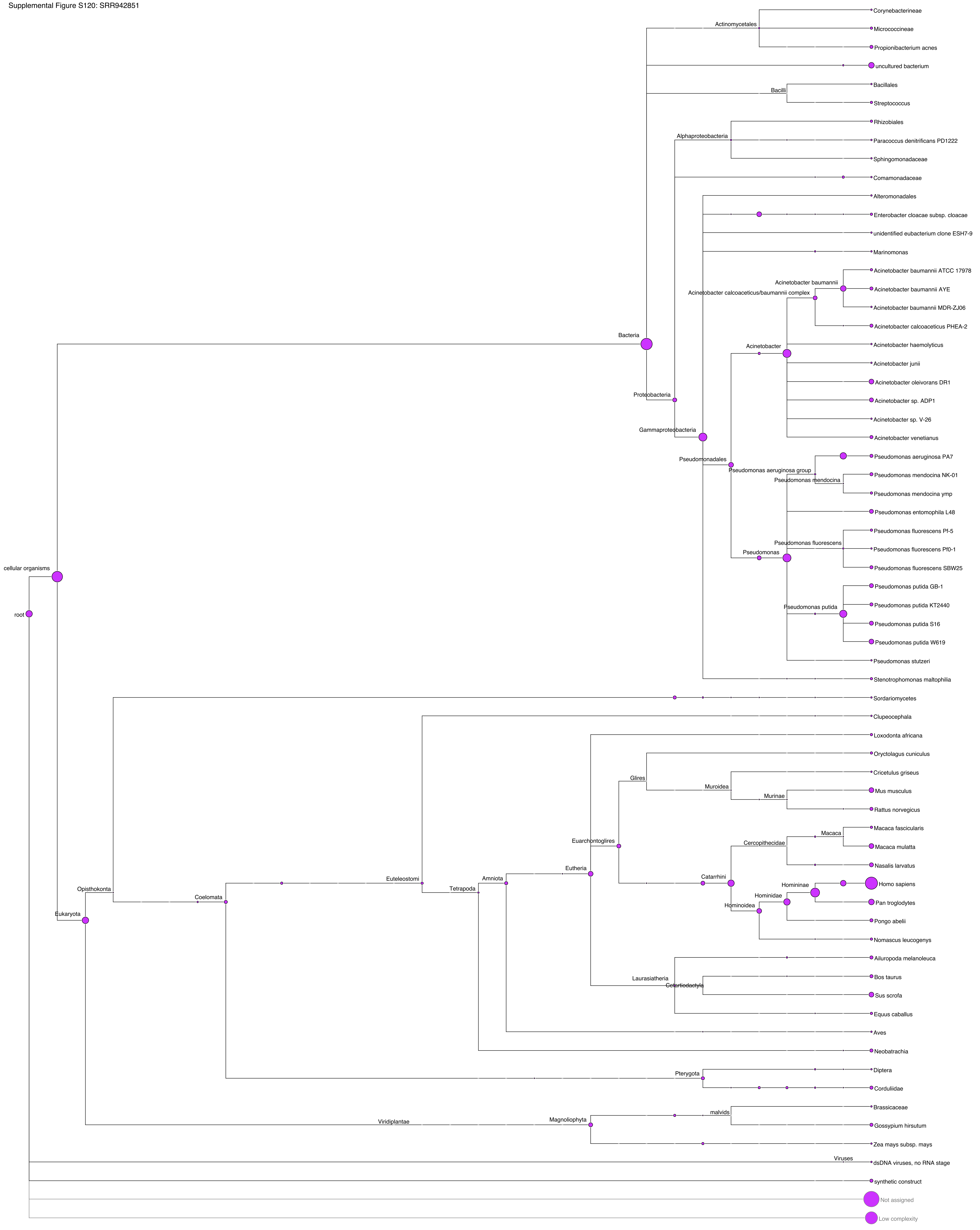

Supplemental Figure S121: SRR942852

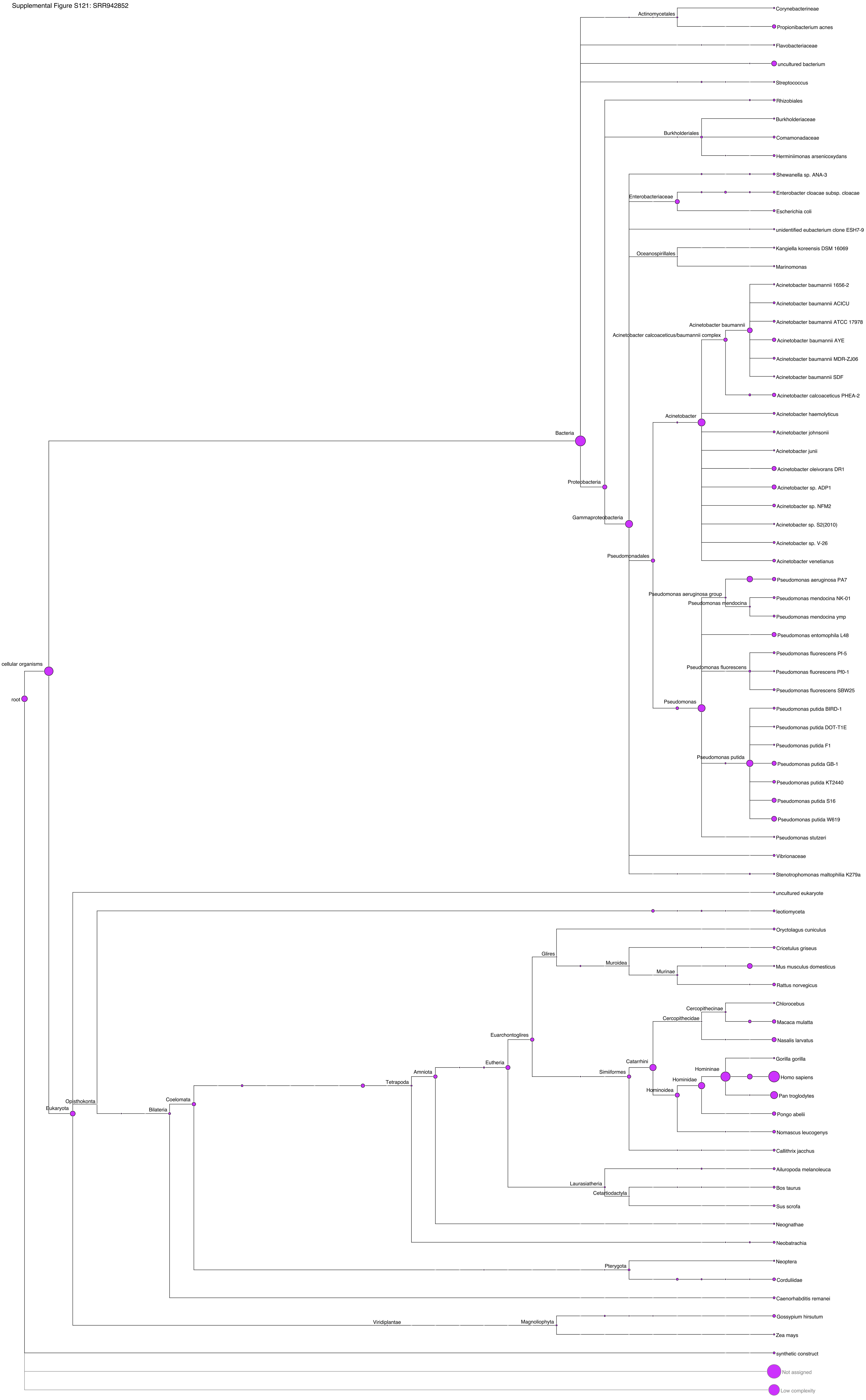

Supplemental Figure S122: SRR942853

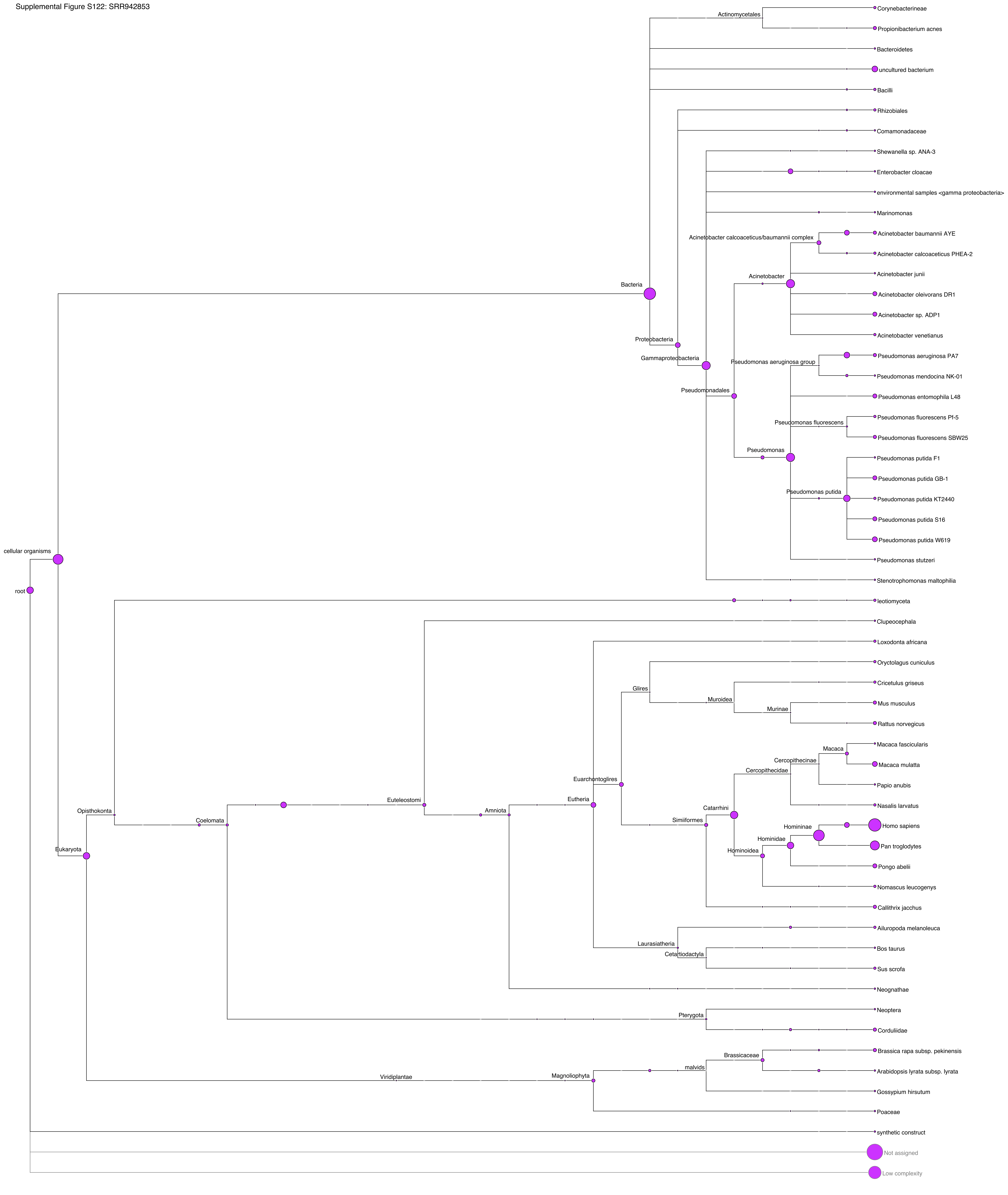

Supplemental Figure S123: SRR942854

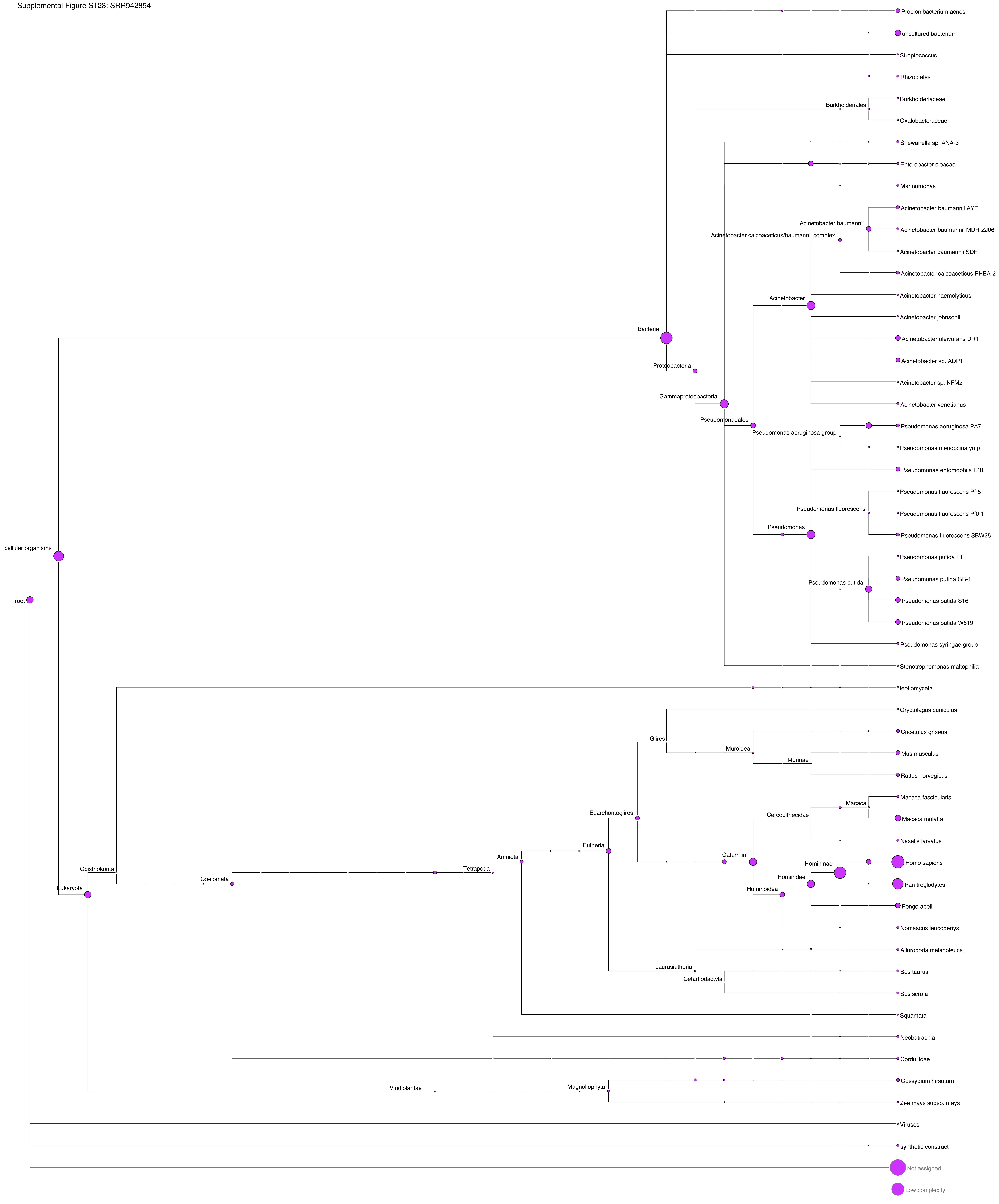

Supplemental Figure S124: SRR942855

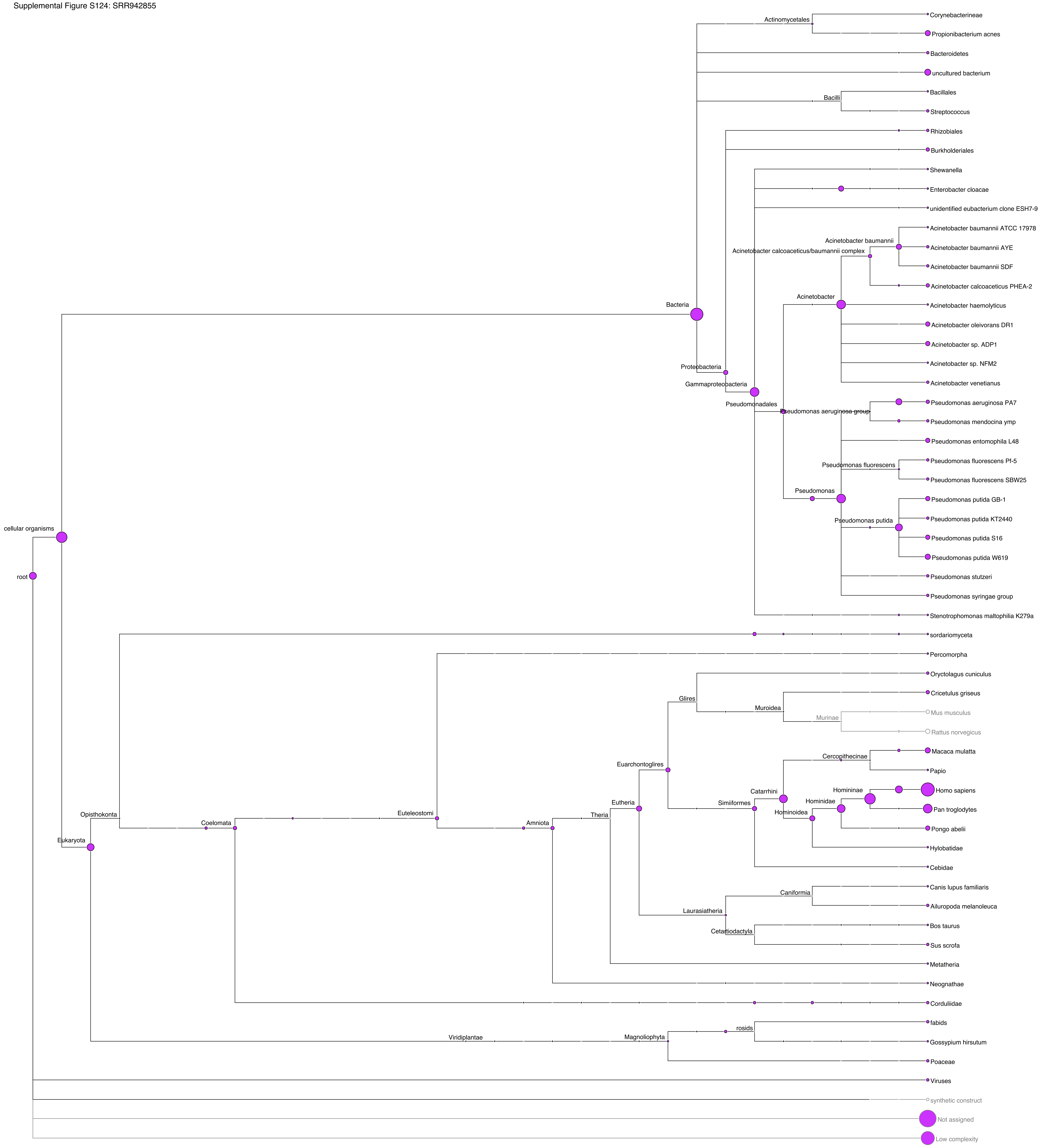

Supplemental Figure S125: SRR942856

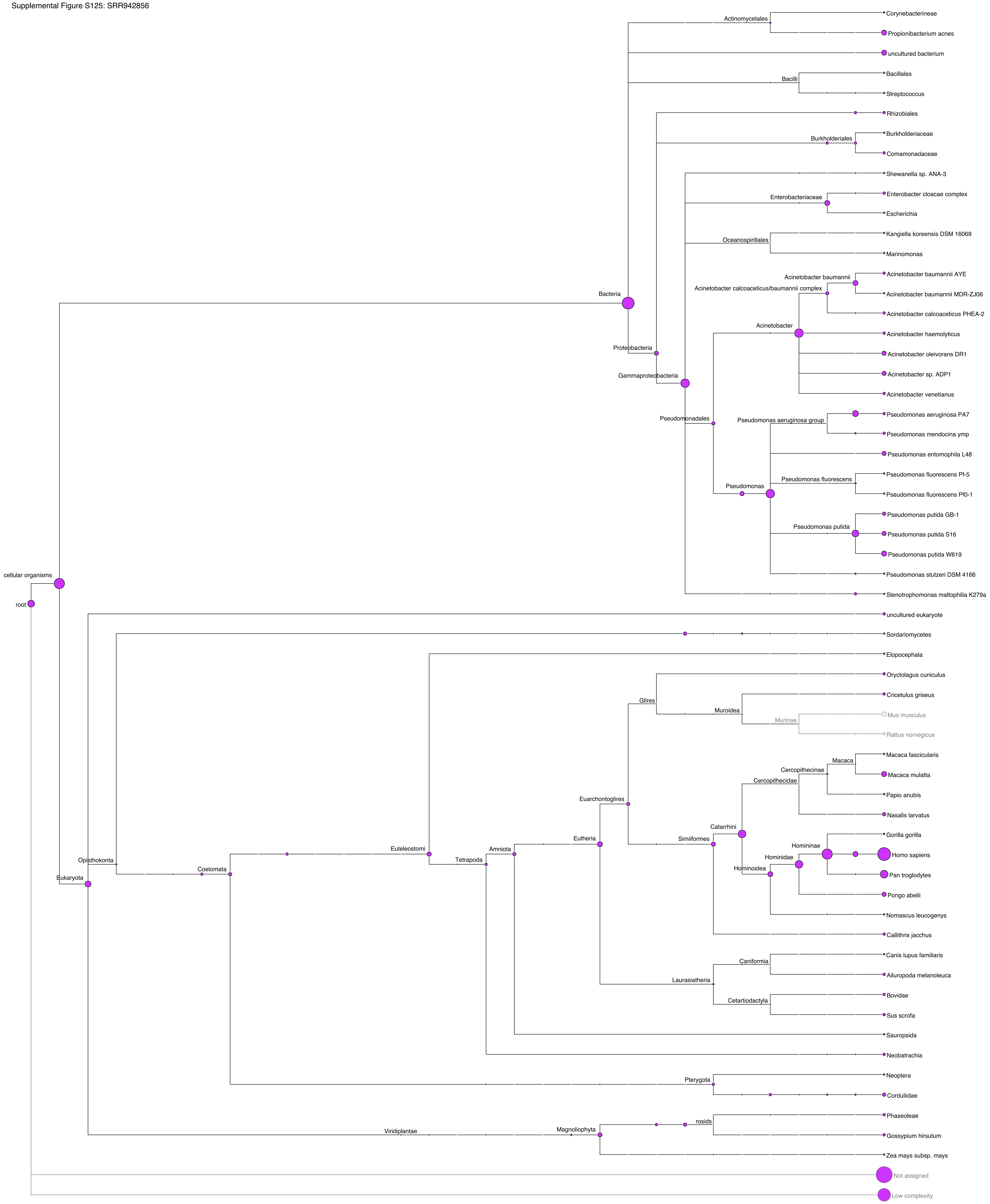

Supplemental Figure S126: SRR942857

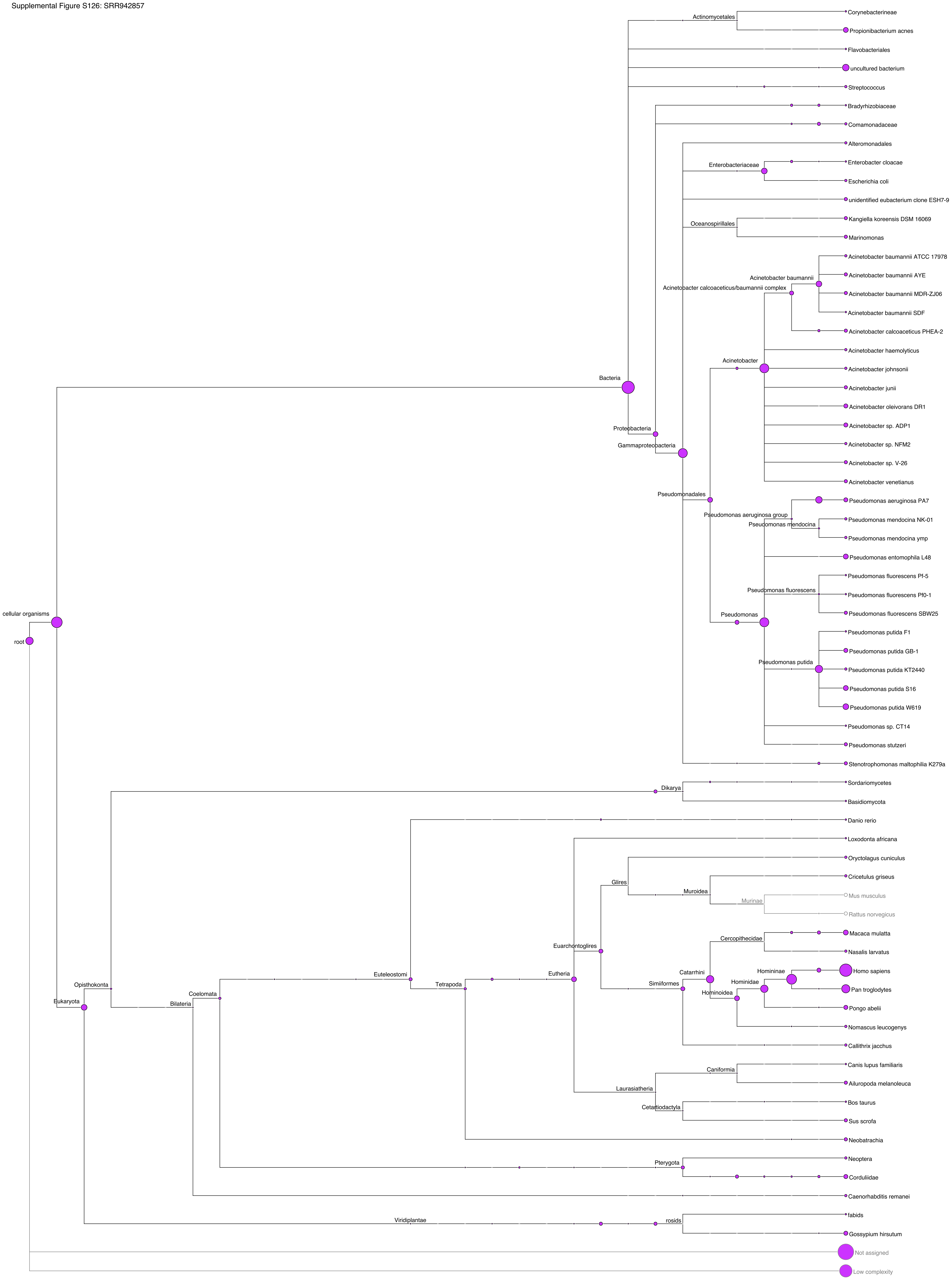

Supplemental Figure S127: SRR942858

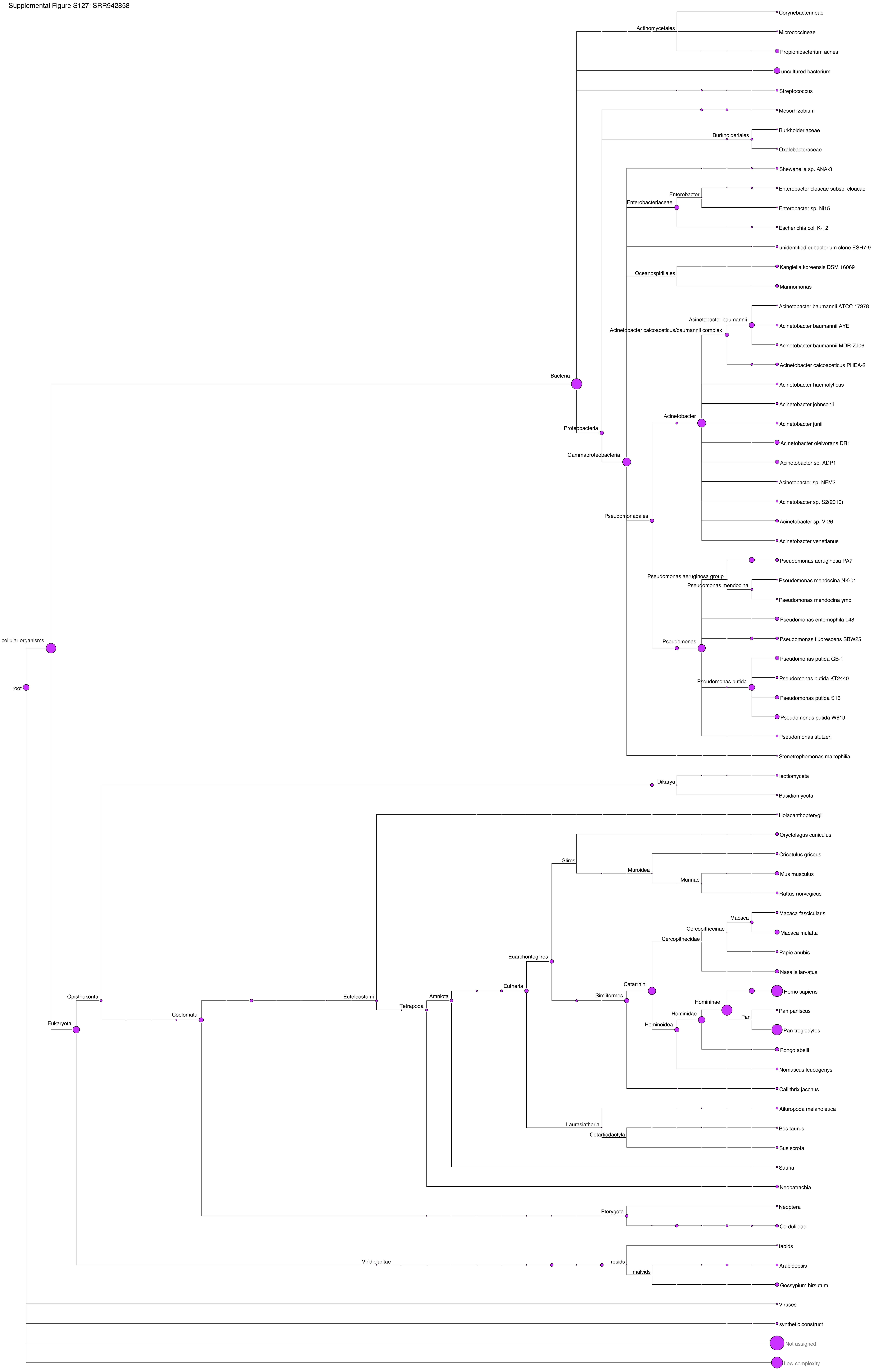

Supplemental Figure S128: SRR942859

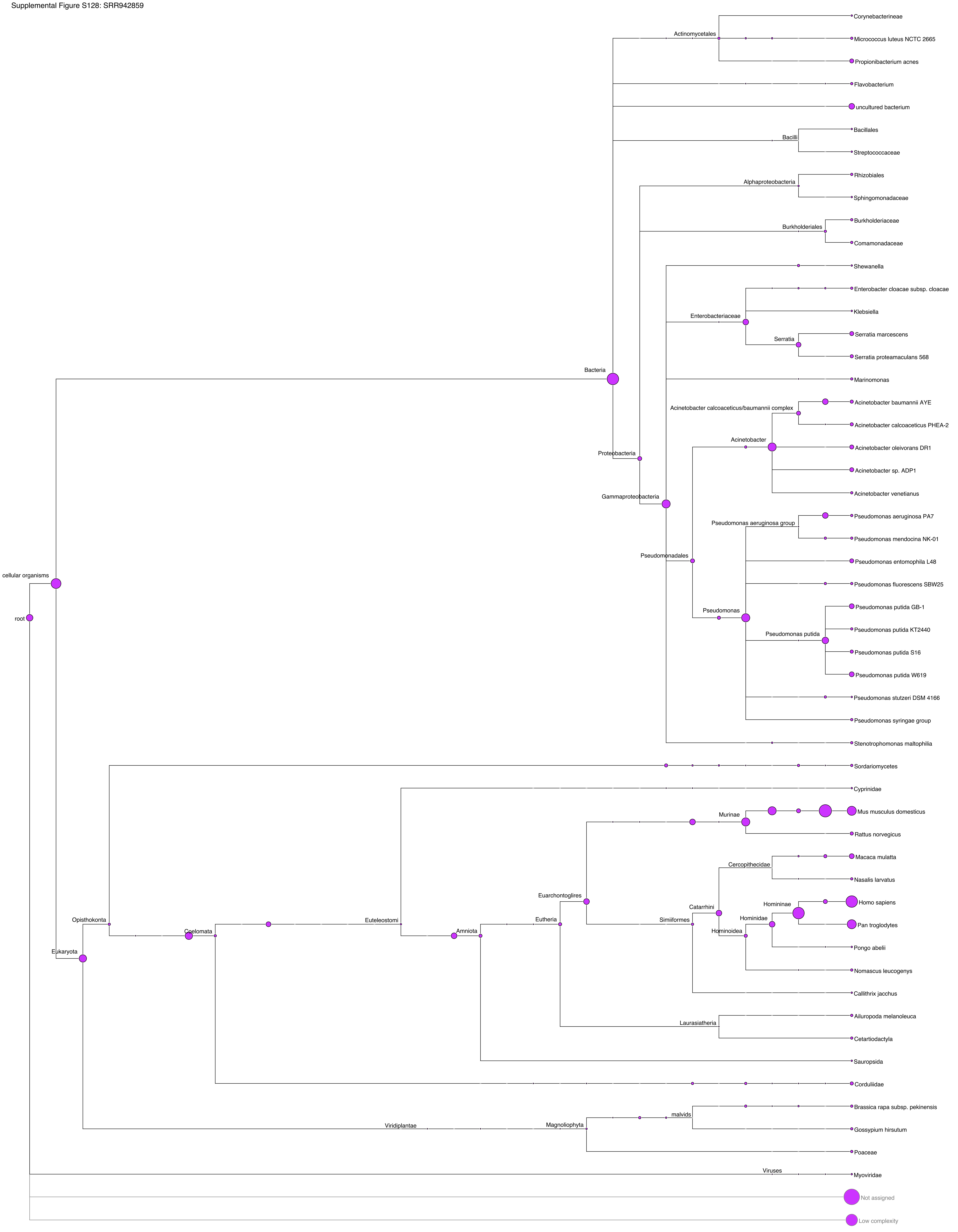

Supplemental Figure S129: SRR942860

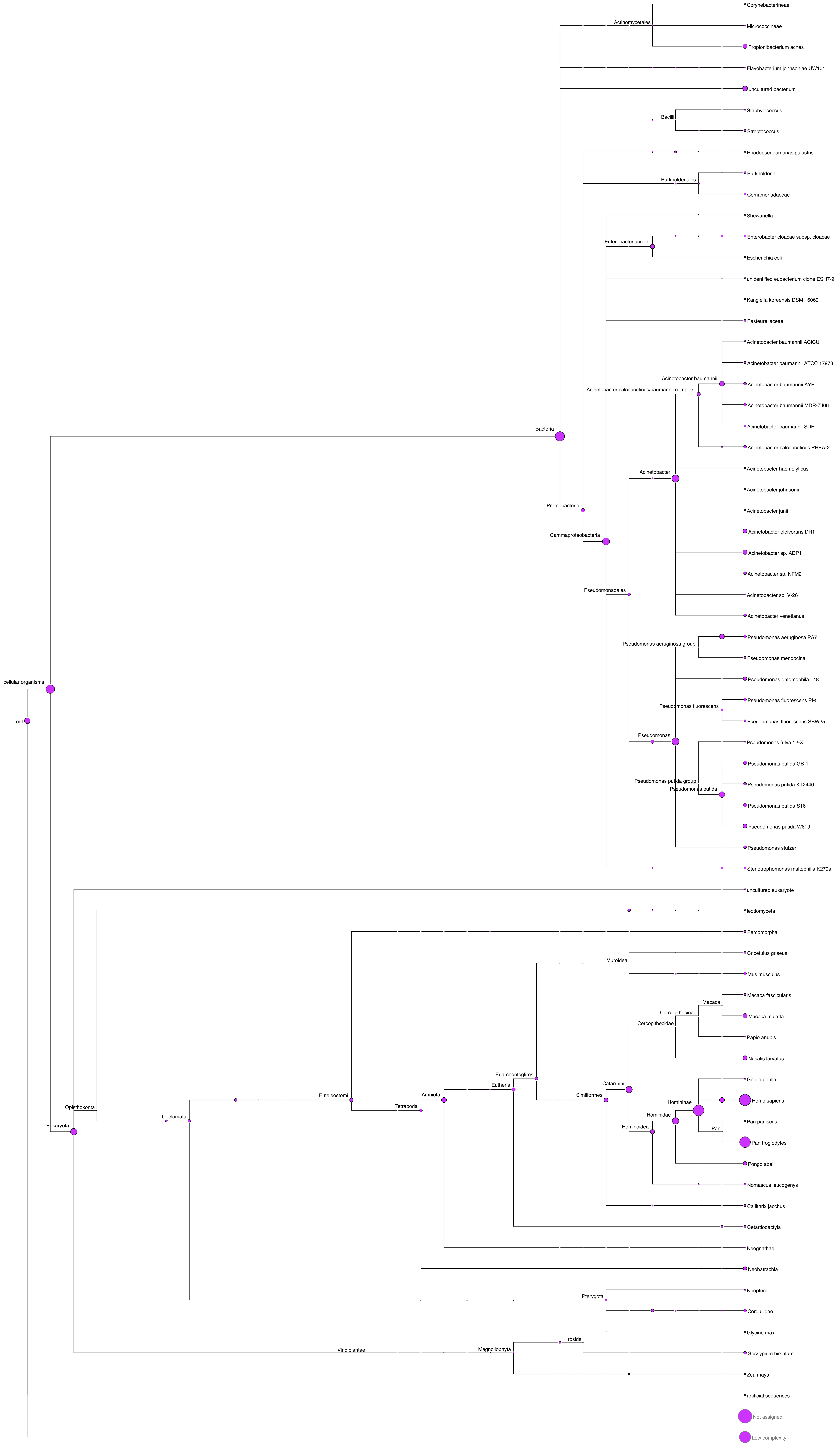

Supplemental Figure S130: SRR942861

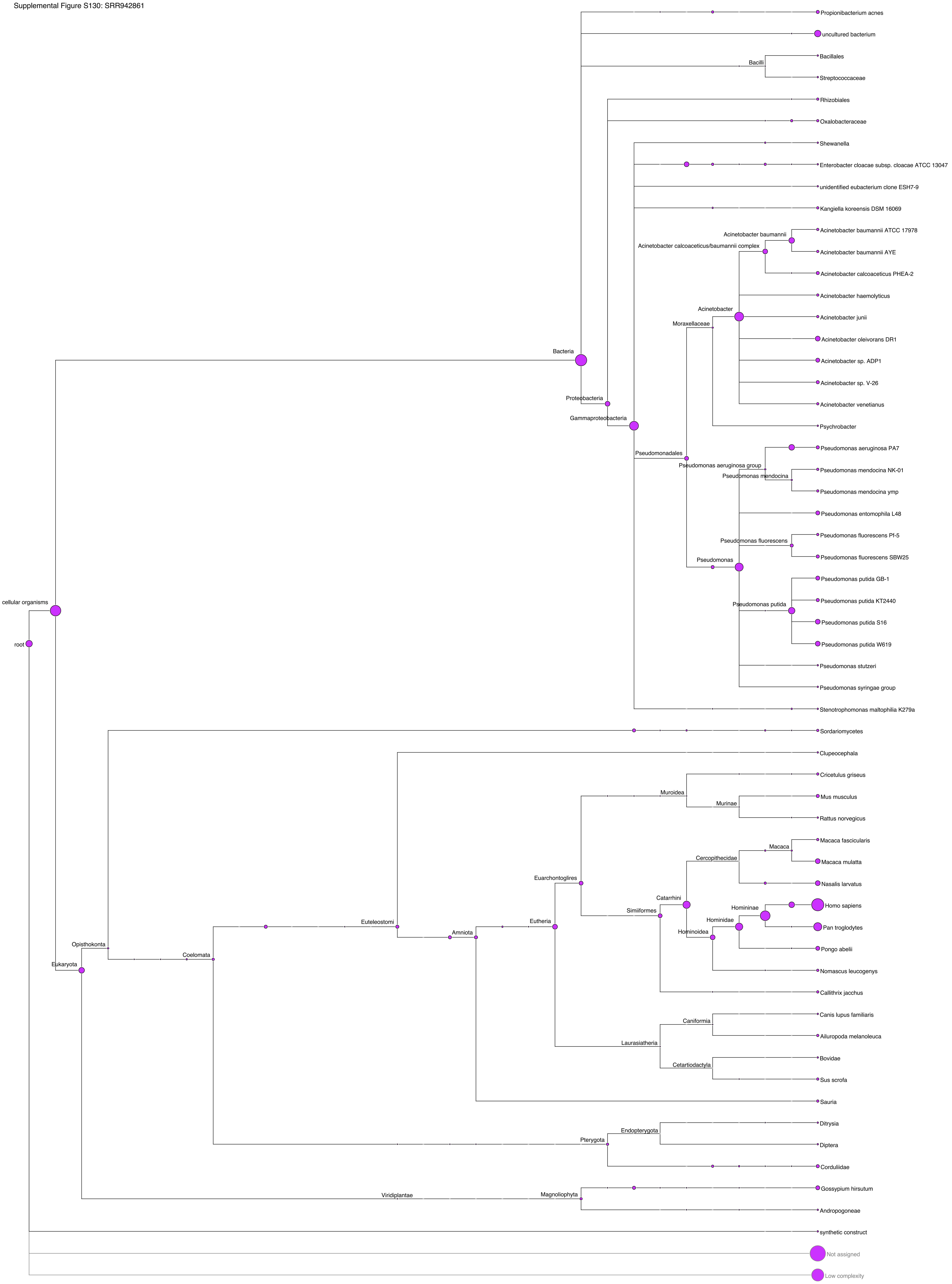

Supplemental Figure S131: SRR942862

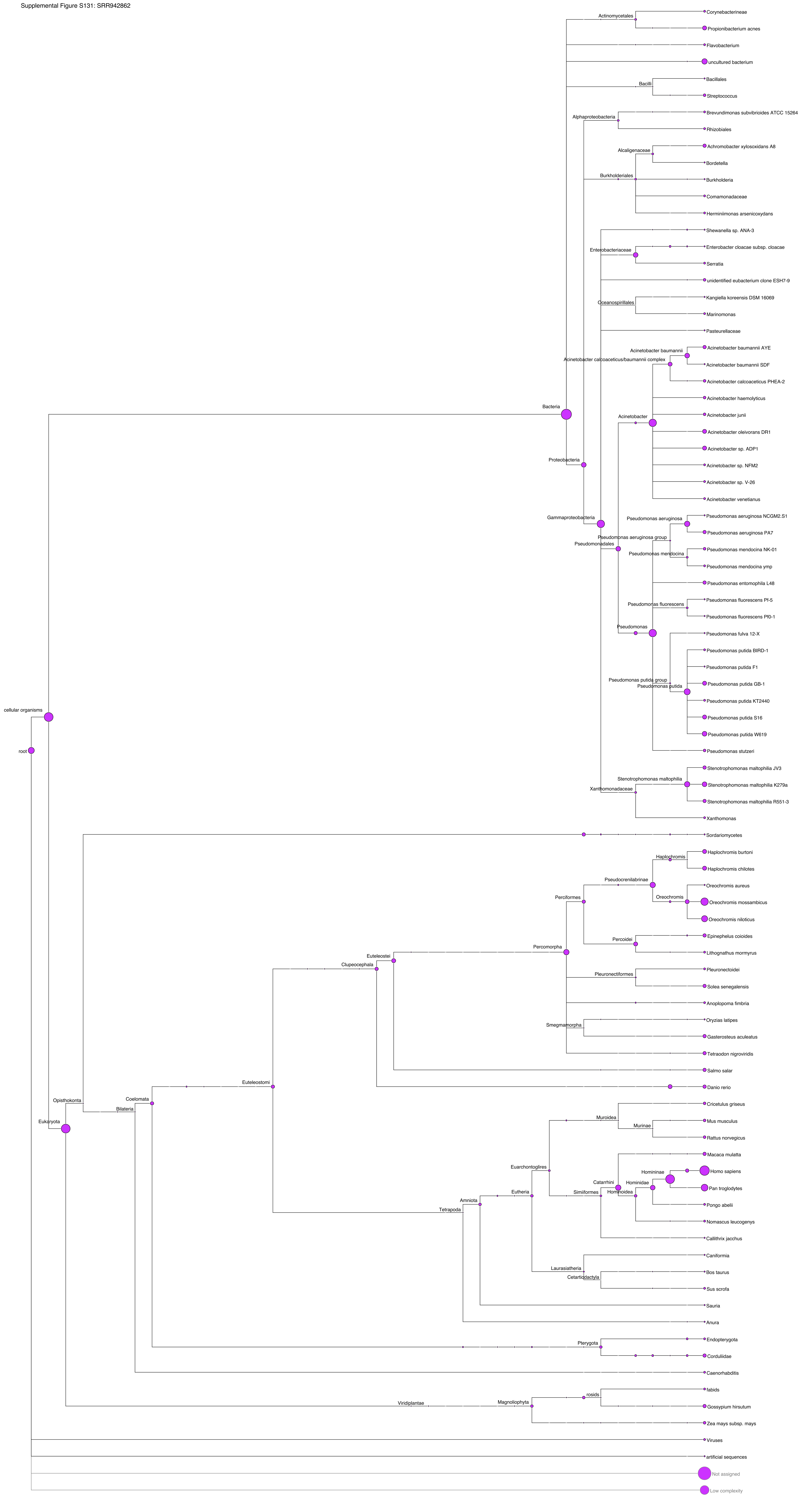

Supplemental Figure S132: SRR942863

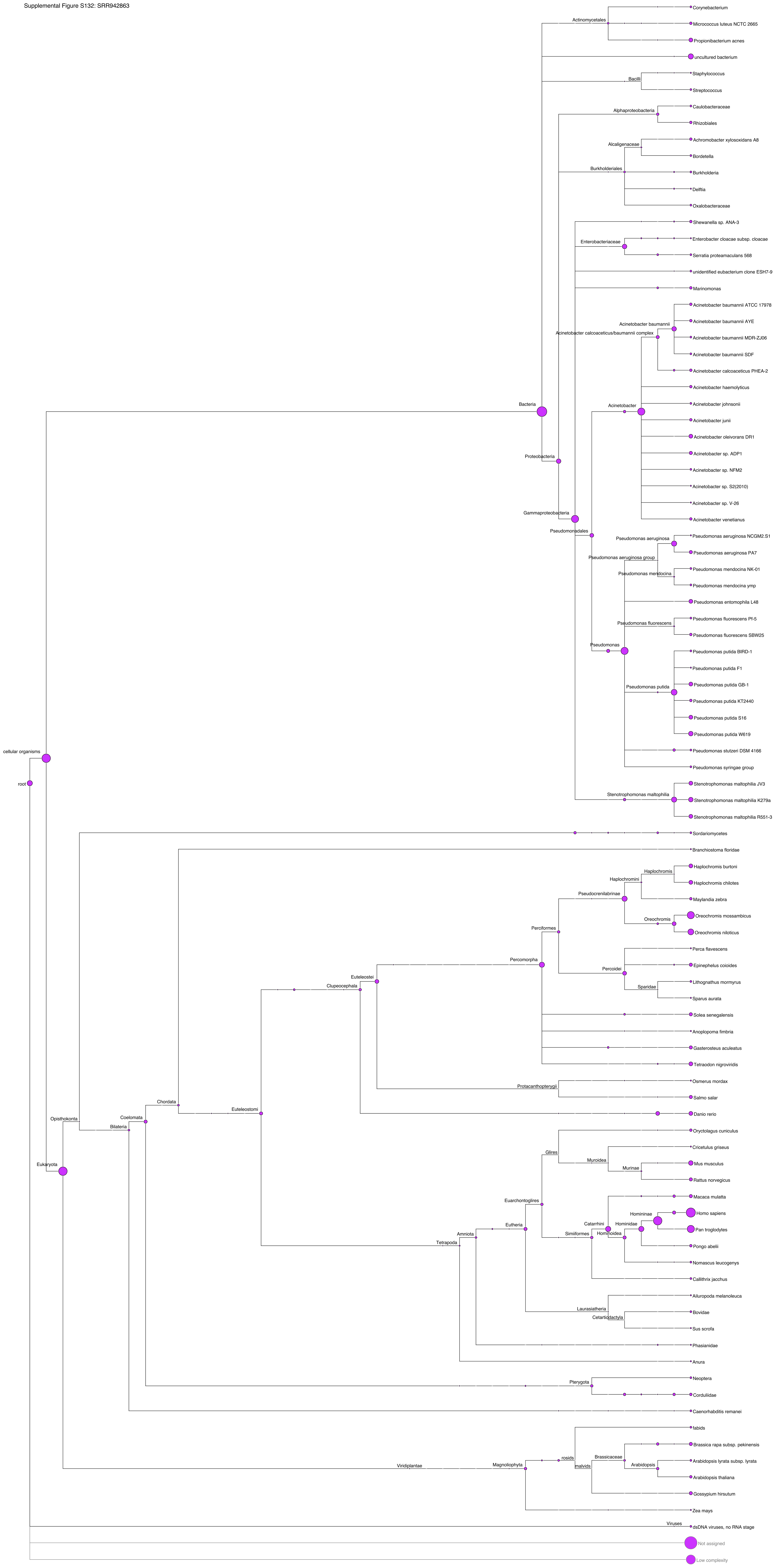

Supplemental Figure S133

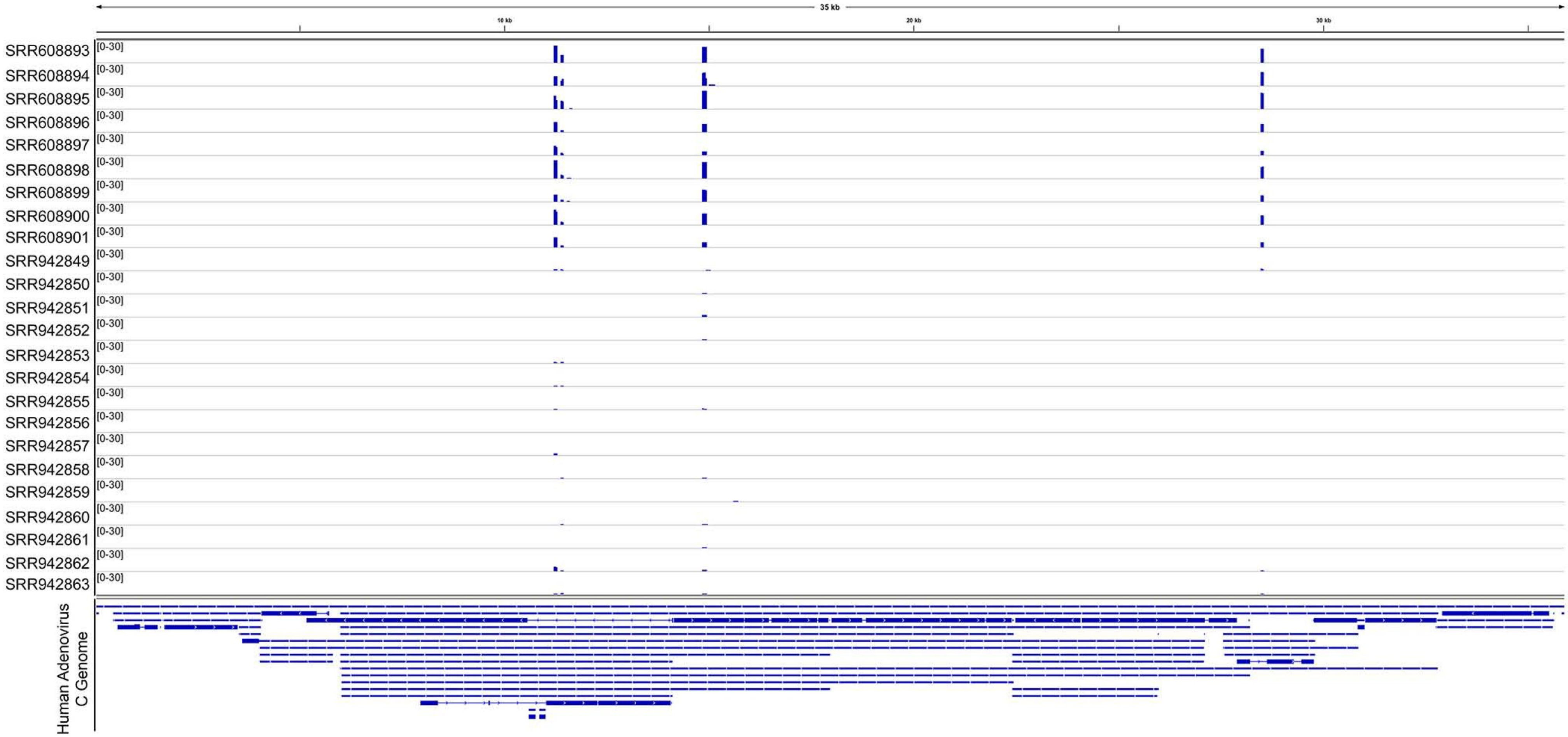

Supplemental Figure S134

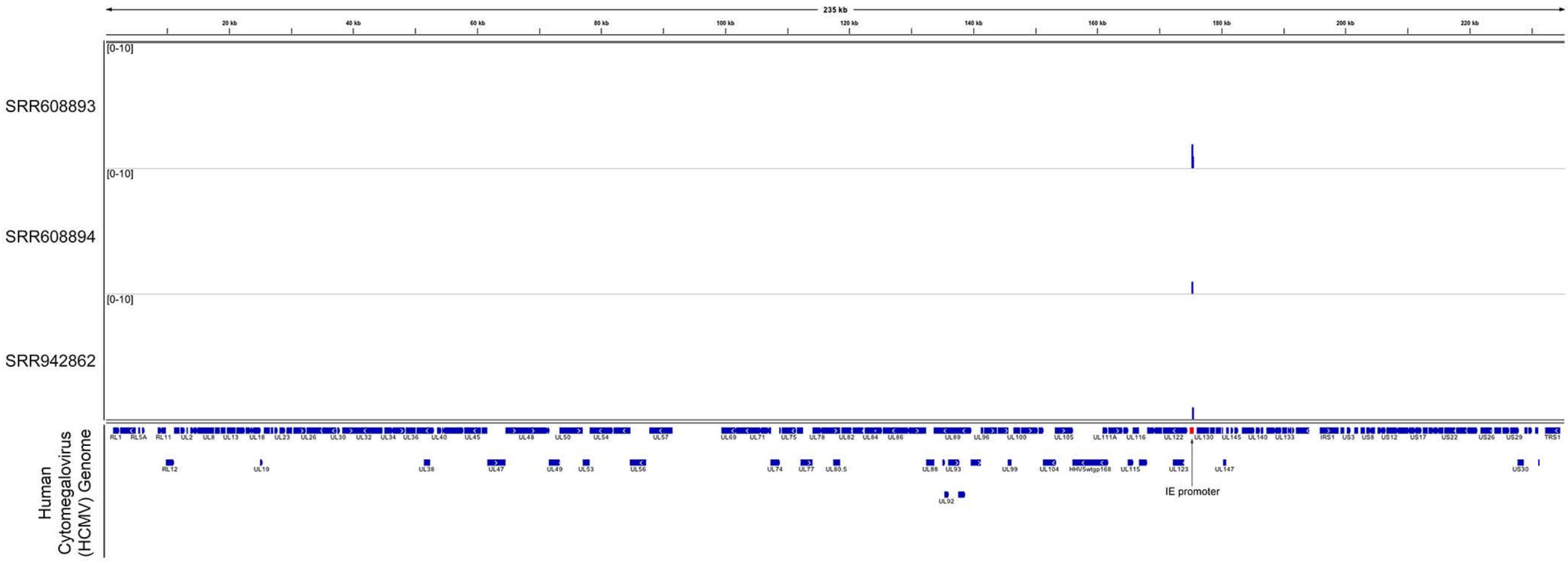

Supplemental Figure S135

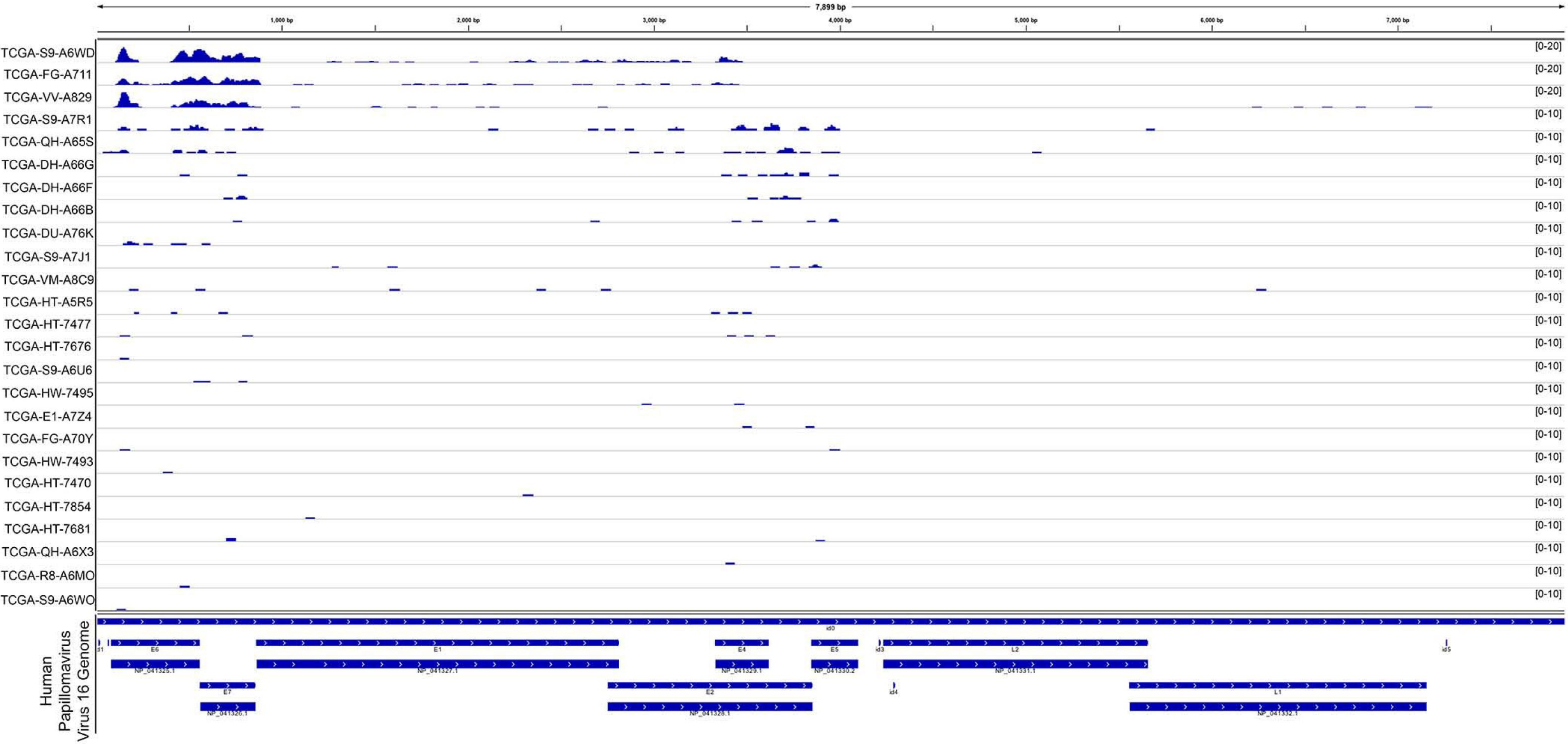

Supplemental Figure S136

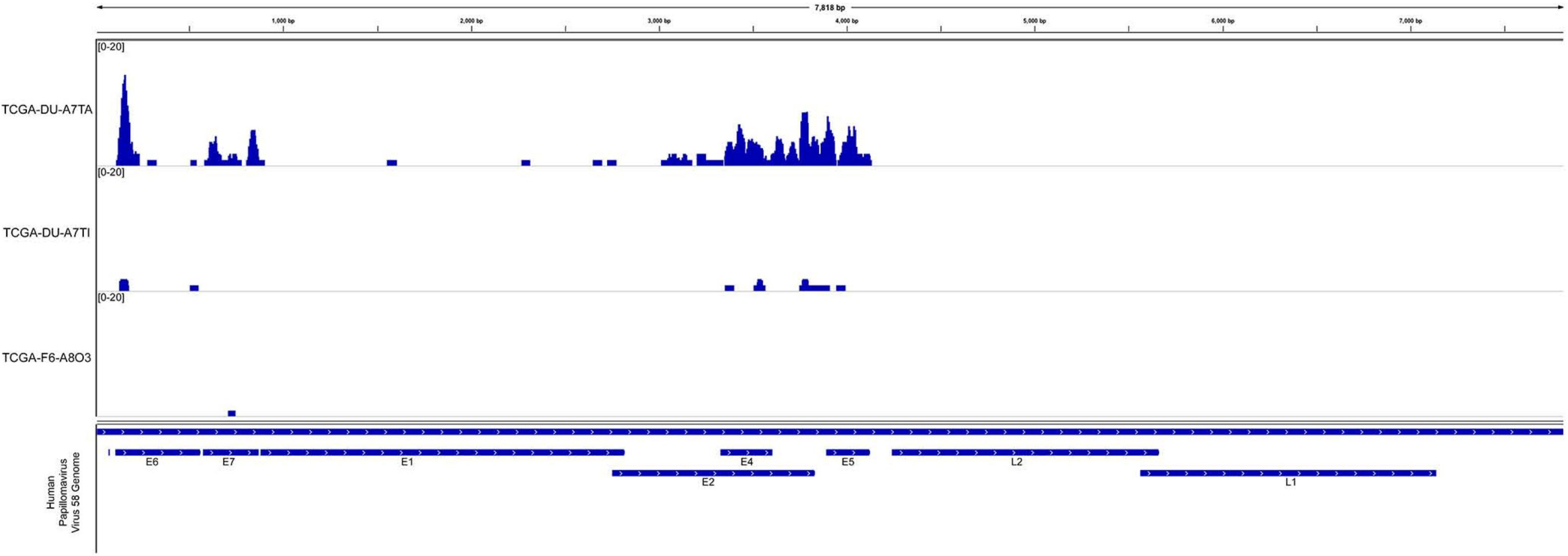

Supplemental Figure S137

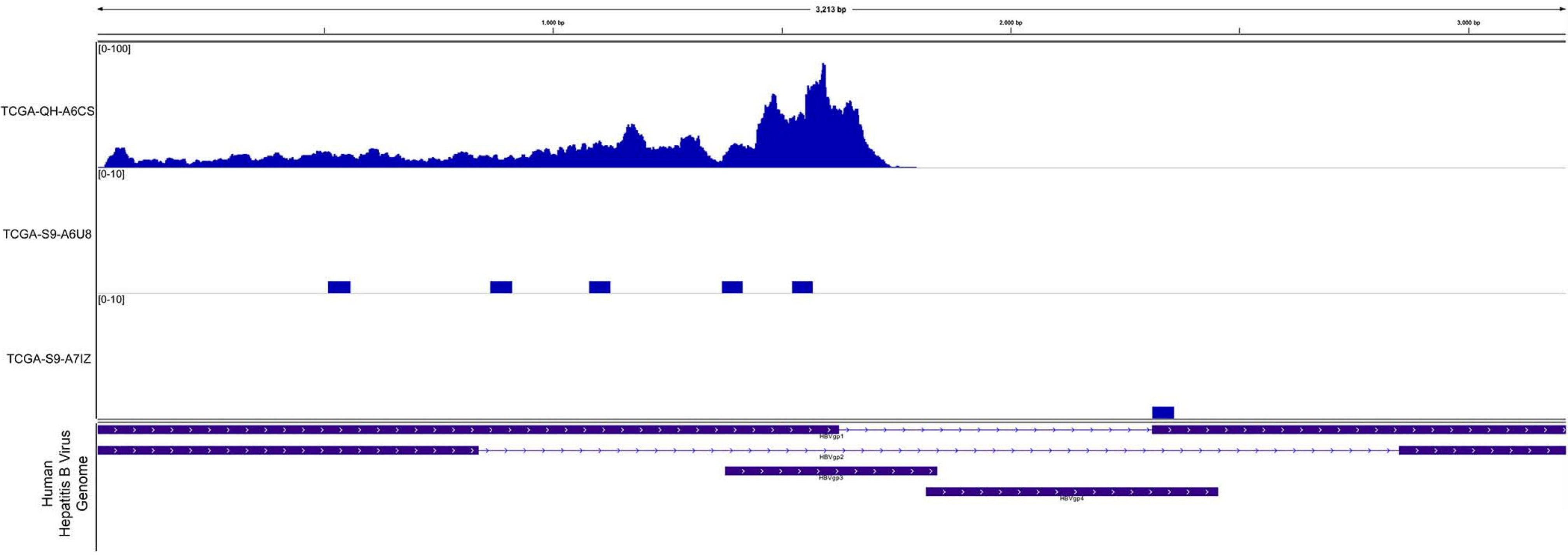

Supplemental Figure S138

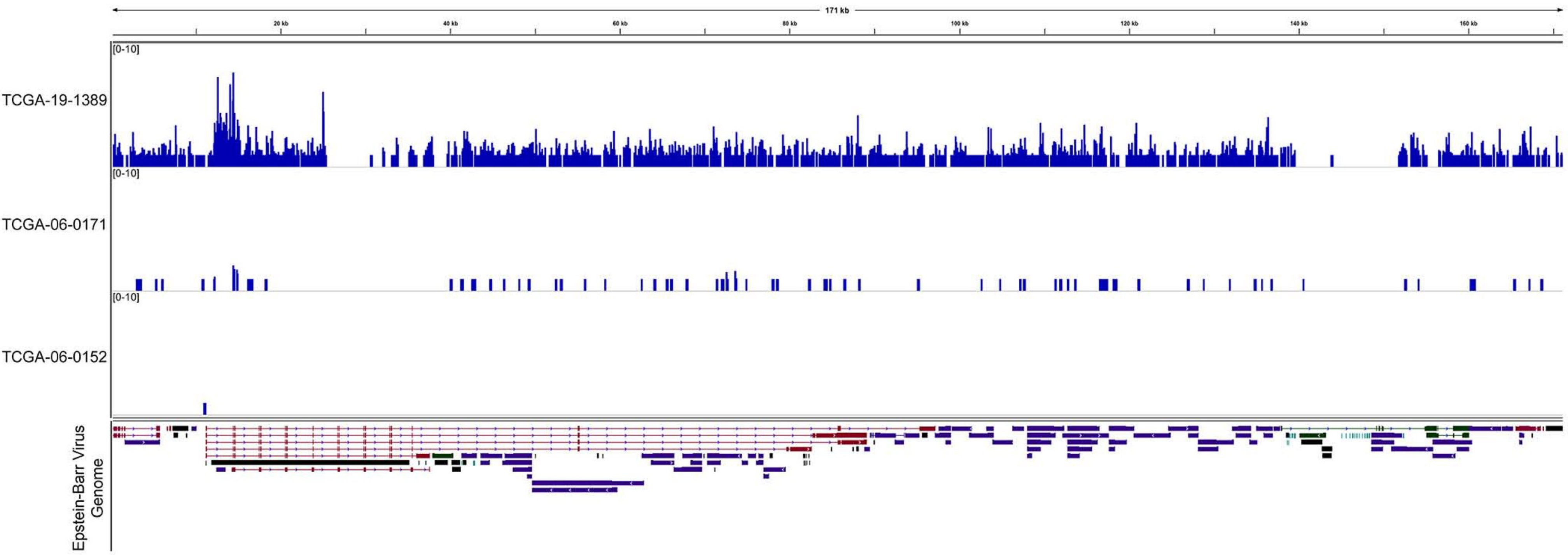

Supplement: Additional file 5: — Supplementary Figures 1-138. (PDF 17.9 mb) [file 40478_2016_338_MOESM5_ESM.pdf]
